# Analysis of nucleotide diphosphate sugar dehydrogenases reveals family and group‐specific relationships

**DOI:** 10.1002/2211-5463.12022

**Published:** 2016-01-11

**Authors:** Nicholas Freas, Peter Newton, John Perozich

**Affiliations:** ^1^ Department of BiologyFranciscan University of SteubenvilleOHUSA

**Keywords:** nucleotide diphosphate sugar dehydrogenase, UDP‐glucose dehydrogenase, UDP‐*N*‐acetyl‐mannosamine dehydrogenase, GDP‐mannose dehydrogenase, multiple sequence alignment

## Abstract

UDP‐glucose dehydrogenase (UDPGDH), UDP‐*N*‐acetyl‐mannosamine dehydrogenase (UDPNAMDH) and GDP‐mannose dehydrogenase (GDPMDH) belong to a family of NAD ^+^‐linked 4‐electron‐transfering oxidoreductases called nucleotide diphosphate sugar dehydrogenases (NDP‐SDHs). UDPGDH is an enzyme responsible for converting UDP‐d‐glucose to UDP‐d‐glucuronic acid, a product that has different roles depending on the organism in which it is found. UDPNAMDH and GDPMDH convert UDP‐*N*‐acetyl‐mannosamine to UDP‐*N*‐acetyl‐mannosaminuronic acid and GDP‐mannose to GDP‐mannuronic acid, respectively, by a similar mechanism to UDPGDH. Their products are used as essential building blocks for the exopolysaccharides found in organisms like *Pseudomonas aeruginosa* and *Staphylococcus aureus*. Few studies have investigated the relationships between these enzymes. This study reveals the relationships between the three enzymes by analysing 229 amino acid sequences. Eighteen invariant and several other highly conserved residues were identified, each serving critical roles in maintaining enzyme structure, coenzyme binding or catalytic function. Also, 10 conserved motifs that included most of the conserved residues were identified and their roles proposed. A phylogenetic tree demonstrated relationships between each group and verified group assignment. Finally, group entropy analysis identified novel conservations unique to each NDP‐SDH group, including residue positions critical to NDP‐sugar substrate interaction, enzyme structure and intersubunit contact. These positions may serve as targets for future research.

**Enzymes:**

UDP‐glucose dehydrogenase (UDPGDH, EC 1.1.1.22).

**Abbreviations**
ADH: alcohol dehydrogenase
ALDH: aldehyde dehydrogenase
GDP‐Man: GDP‐mannose
GDP‐ManUA: GDP‐mannuronic acid
GDPMDH: GDP‐mannose dehydrogenase
hUDPGDH: human UDPGDH
NDP‐SDH: nucleotide diphosphate sugar dehydrogenase
PaGDPMDH: *Pseudomonas aeruginosa* GDPMDH
SaUDPNAMDH: *Staphylococcus aureus* UDPNAMDH
SpUDPGDH: *Streptococcus pyogenes* UDPGDH
UDPGDH: UDP‐glucose dehydrogenase
UDP‐Glc: UDP‐d‐glucose
UDP‐GlcUA: UDP‐d‐glucuronic acid
UDP‐ManNAc: UDP‐*N*‐acetyl‐mannosamine
UDP‐ManNAcUA: UDP‐*N*‐acetyl‐mannosaminuronic acid
UDPNAMDH: UDP‐*N*‐acetyl‐mannosamine dehydrogenase

UDP‐glucose dehydrogenase (UDPGDH, EC 1.1.1.22) and its homologous bacterial and archaeal proteins UDP‐*N*‐acetyl‐mannosamine dehydrogenase (UDPNAMDH) and GDP‐mannose dehydrogenase (GDPMDH) belong to a small group of NAD^+^‐linked 4‐electron‐transfering oxidoreductases termed nucleotide diphosphate sugar dehydrogenases (NDP‐SDHs) 1. UDPGDH was first detected in bovine liver in 1954 2. It was subsequently purified in 1969 3 and sequenced in 1994 4. UDPGDH has since been identified as the rate determining step in the conversion of UDP‐d‐glucose (UDP‐Glc) to UDP‐d‐glucuronic acid (UDP‐GlcUA) by reducing two molecules of NAD^+^ to NADH through two cycles of oxidation 4.

UDPGDH is found in a variety of different organisms from bacteria to plants and animals, and maintains consistency in its mechanism for converting UDP‐Glc to UDP‐GlcUA. Nevertheless, UDPGDH has different quaternary structure in unlike organisms. In the bacteria *Streptococcus pyogenes* UDPGDH (SpUDPGDH) exists as a homodimer 5, whereas studies report its existence in bovines and humans (hUDPGDH) as a homohexamer with ‘half‐of‐the sites’ reactivity, essentially acting as a trimer of dimers 1, 6. Similarly, UDP‐GlcUA has different fates according to the organism in which it is found. In several strains of *Streptococcus* UDP‐GlcUA is the substrate for the production of polysaccharides that comprise the organism's capsule, which aids in surface attachment, increases antibiotic resistance and protects against phagocytosis 7. UDP‐GlcUA is also used by *Burkholdaria cepacia* to synthesize the exopolysaccharide cepacian, a major virulence factor 8. In mammals UDP‐GlcUA serves as precursor to hyaluronan and various glycosaminoglycans. Hyaluronan is found in the extracellular matrix and plays a role in promoting cell growth and migration 9. Interfering with proteoglycan synthesis reduces tumour growth and development 10, 11. Hence, glycosaminoglycans are associated with cancer metastasis 12. Loss of UDPGDH function leads to major problems in embryogenesis, such as heart valve defects in zebrafish and vulval morphogenesis in *Caenorhabditis elegans* 13, 14, while UDPGDH overexpression can lead to chondrogenesis 15. Additionally, UDP‐GlcUA in *Drosophila melanogaster* acts as a modifier for proteins involved in wing formation 16. Another role of UDP‐GlcUA is the glucuronidation of molecules in the liver that targets these compounds for excretion 17. Some lung and colon cancers have actually taken advantage of this activity for drug resistance 18, 19. UDP‐GlcUA also serves as a precursor to UDP‐xylose, a critical component of plant cell wall polysaccharides such as pectin and hemicellulose 20, 21. Amazingly, UDPGDH from *Sphingomonas elodea* has even been shown to also exhibit ribonuclease activity 22.

GDPMDH uses a similar mechanism to convert GDP‐mannose (GDP‐Man) to GDP‐mannuronic acid (GDP‐ManUA) while in turn reducing two molecules of NAD^+^ to NADH. This enzyme in *Pseudomonas aeruginosa* (PaGDPMDH) is the rate‐limiting step in the synthesis of alginate, an exopolysaccharide that protects the organism from antibiotics and host defences and allows *P. aeruginosa* to act as an opportunistic pathogen. There is no equivalent enzyme in humans. PaGDPMDH shares only about 22% identity to SpUDPGDH and, unlike other NDP‐SDHs, has a domain‐swapped dimeric structure 23.

UDPNAMDH converts UDP‐*N*‐acetyl‐mannosamine (UDP‐ManNAc) to UDP‐*N*‐acetyl‐mannosaminuronic acid (UDP‐ManNAcA) while in turn reducing two molecules of NAD^+^ to NADH. The Cap50 enzyme in *Staphylococcus aureus* (SaUDPNAMDH) is a UDPNAMDH responsible for synthesizing UDP‐ManNAcA for incorporation into *S. aureus*'s polysaccharide capsule. SaUDPNAMDH only shares approximately 20% identity to SpUDPGDH and PaGDPMDH. SaUDPNAMDH possess a dimeric organization similar to that of SpUDPGDH. Also, tyrosine phosphorylation, most likely on Tyr89, has been shown to increase the activity of SaUDPNAMDH, similar to what has previously been demonstrated for UDPGDHs from *E. coli* and *Bacillus subtilis* 24, 25, 26.

The mechanism for UDPGDH, which is common to the other NDP‐SDHs, proceeds by a Bi‐Uni‐Uni‐Bi Ping Pong mechanism 27. It begins with an aspartate residue (Asp264 in SpUDPGDH) acting as a general base by activating a water molecule 28. This proceeds to the oxidation of the C6″ hydroxyl of UDP‐Glc to form an aldehyde intermediate and the transfer of the *pro‐R* hydride to NAD^+^ to form NADH 29. Secondly, a cysteine (Cys260 in SpUDPGDH) acts as a nucleophile by attacking the aldehyde, yielding a covalent thiohemiacetal intermediate 30, 31. This is followed by the transfer of the remaining hydride (*pro‐*S) at the C6″ position to a second NAD^+^ to again form NADH. The final, rate‐limiting step of the UDPGDH mechanism is the hydrolysis of the remaining thioester intermediate, which is catalysed by Tyr10 in SpUDPGDH, to yield UDP‐GlcUA 5, 8, 28.

The current era of genomics has yielded a large number of sequences for each type of NDP‐SDH. In addition, tertiary structures are now available for each enzyme. Relationships between each enzyme have scarcely been addressed in previous studies. The goal of this research was to align a large number of protein sequences for each NDP‐SDH homologue and to identify and confirm the structural and functional roles of residues and sequence motifs in UDPGDHs, UDPNAMDHs and GDPMDHs. Group entropy analysis was also performed to identify group‐specific conservations for each NDP‐SDH homolog, yielding new insights into the unique function of each enzyme.

## Results and discussion

### Structure and residue conservations

**Figure 1:**
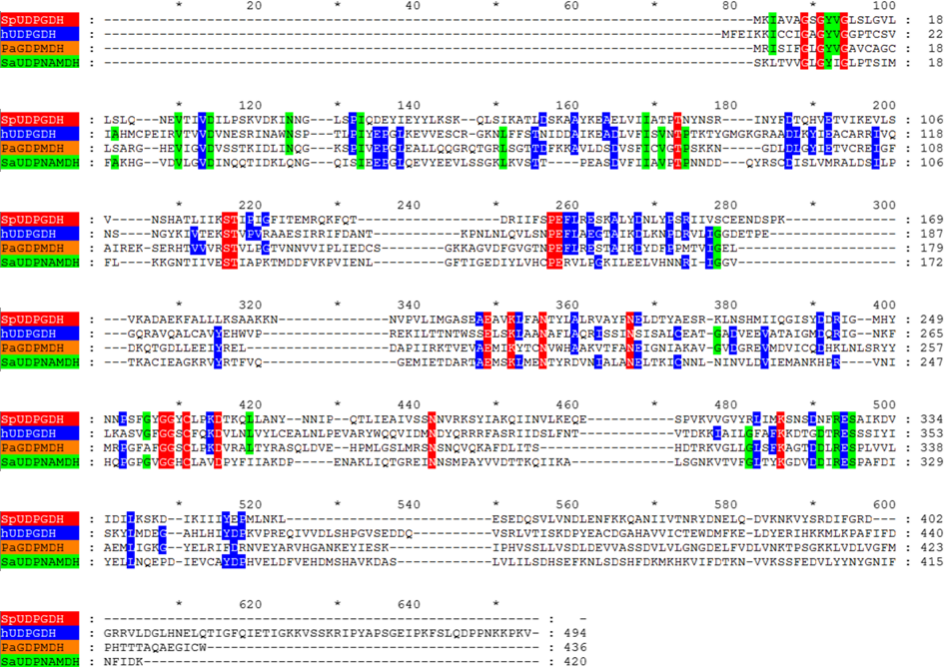
Summary alignment showing a representative sequence for each group of nucleotide diphosphate sugar dehydrogenase (NDP‐SDH) highlighted in the phylogenetic tree (Fig. 3): *Streptococcus pyogenes* UDPGDH (SpUDPGDH) as a Bacterial UDPGDH, human UDPGDH (hUDPGDH) as a Eukaryotic UDPGDH,* Pseudomonas aeruginosa* GDPMDH (PaGDPMDH) as a GDPMDH and *Staphylococcus aureus* UDPNAMDH (SaUDPNAMDH) as a UDPNAMDH. Sequence names (left) are colour‐coded to correspond to the phylogenetic tree. Conserved residue positions are coloured as follows: red = 100% conserved in the entire alignment, green = 80–99% conserved and blue = 60–79% conserved. The entire alignment, which contains 229 protein sequences, is found in Fig. S1. Gap positions from the entire alignment are retained to allow correlation with index position numbers (shown above the alignment) that are noted within the text.

A total of 229 amino acid sequences were aligned (Fig. 1) using tertiary structural alignment as a guide. The full alignment is available in Fig. S1. The sequences used included 92 bacterial and archaeal UDPGDHs, 55 eukaryotic UDPGDHs, 38 UDPNAMDHs and 44 GDPMDHs sequences. Despite only about 20% sequence identity between each enzyme in the family, their tertiary structures are well conserved 5, 6, 23, 24. Eukaryotic UDPGDHs have a slightly longer loop after β‐5 (alignment indices 179–183) as compared to the other NDP‐SDHs. GDPMDHs have an extended loop after α‐5 (alignment indices 202–206) and also after α‐10 (alignment indices 386–387). UDPNAMDHs have an extended loop after α‐6 (alignment indices 236–243).

The sequence alignment demonstrated 18 invariant residues: Gly7{89}, Gly9{91}, Gly12{94}, Thr83{173}, Ser117{216}, Thr118{217}, Pro140{257}, Glu141{258}, Glu201{349}, Lys204{352}, Asn208{356}, Asn219{367}, Gly257{408}, Gly258{409}, Cys260{411}, Asp264{415}, Asn287{442} and Lys320{486} (residue identities are based on SpUDPGDH positions 5 with alignment index position numbers in curled brackets). Most conservations cluster in and around the active site. The overrepresentation of glycines among the invariant residues (5 of 18) is usually due to their critical role in protein structure and has been seen to occur in other enzyme families, such as aldehyde dehydrogenases (ALDH) and alcohol dehydrogenases (ADH) 32, 33, 34. These conserved glycines often lie at critical turns in the enzyme structure or at sites where a side chain would lead to steric issues with protein folding. For example, glycines 7{89}, 9{91} and 12{94} comprise the classic ‘GXGXXG’ pattern of the Rossmann fold which coordinates NAD^+^ in NDP‐SDHs and many other oxidoreductases, including ALDHs and ADHs 32, 33, 34. The first glycine position, Gly7{89}, is also invariant in the Rossmann folds of other dehydrogenase families 32, 35. Gly257{408}, which forms a hydrogen bond to the 3′ hydroxyl of UMP 5 and Gly258{409} are located in the turn preceding the catalytic thiol, Cys260{411}. Cys260{411} is the covalent catalyst for the second oxidation step in the catalytic mechanism 9, 36. Interestingly upon oxidation of SaUDPNAMDH, the catalytic Cys258{411} forms a disulphide with Cys92{186} that has not been seen in other NDP‐SDHs 24.

The side chain hydroxyl of Thr83{173} hydrogen bonds to the 3′ hydroxyl of the nicotinamide ribose of NAD^+^. Thr118{217} interacts with a water molecule which in turn hydrogen bonds to the 2′ hydroxyl of the nicotinamide ribose of NAD^+^. This same water molecule is activated as a nucleophile by Asp264{415}, the general base, to initiate the catalytic mechanism 8. Lys204{352} and Asn208{356} are 3.0 Å from the O6″ carboxylate oxygen of UDP‐GlcUA and are involved in the electrostatic stabilization of the substrate in the first oxidation step 5, 28.

Of the remaining invariant residues, the side chain of Ser117{216} is in the NAD^+^‐binding pocket, but lies nearly 4.0 Å from the nicotinamide ribose. Pro140{257} positions Glu141{258} so that its side chain carbonyl can hydrogen bond with the main chain nitrogen of Leu143{260} (77% conserved). This interaction holds the loop between β‐8 and α‐7 in place. The main chain carbonyl of Glu141{258} also forms an ionic bond with the side chain of Lys204{352}, noted above. The side chain carboxyl of Glu201{349} is 2.7 Å from the main chain nitrogen of Gly122{221} (69% conserved), maintaining enzyme structure 5. Interestingly, an E201D mutation in *Streptococcus pneumoniae* causes the lack of a capsule 7. The amide nitrogen of the side chain of Asn287{442} is 2.9 Å from the main chain nitrogen of Tyr256{407} (not conserved). The side chain carboxyl of Asn219{367} is 2.8 Å from the main chain nitrogen of Ser253{404} (not conserved). Both asparagines (219 & 287) hold the loop between α‐10 and α‐11 in position; α‐11 includes the catalytic residues Cys260{411} and Asp264{415}. Lastly, Lys320{486} coordinates diphosphate bridge of UDP‐glucose 5.

In addition to the 18 invariant residues, 20 residues were conserved in at least 80% of the aligned sequences and 52 additional residues were at least 60% conserved. With 90 residue positions conserved in NDP‐SDH sequences that are roughly 425 amino acids in length, this represents a fairly high degree of conservation despite each different enzyme using a different nucleotide‐sugar substrate. Other highly conserved residues that play functional roles in UDPGDH include the following: Tyr10{92}, Asp29{114}, Asn39{124}, Glu145{262}, Arg244{393}, Asn325{491}, Arg327{493} and Ser329{495}. Tyr10{92} is 98% conserved in our alignment and catalyses the final hydrolysis of the enzymatic thioester intermediate 8. Asp29{114} (99% conserved) coordinates both the 2′ and 3′ hydroxyls of the adenosine ribose of NAD^+^. Asn39{124} (83%), which is in α‐2, hydrogen bonds via its side chain amide nitrogen to the main chain carbonyl oxygen of Ala63{181}, which is not conserved and lies in β‐3. Glu145{262} (76%) is 4.7 Å from the UDP‐glucose diphosphate bridge. Arg244{393} (76%) from subunit b of the dimer is 3.1 Å from 2″ hydroxyl group of glucose in UDP‐glucose bound in subunit a, and *vice versa* 5. This intersubunit contact may participate in the communication that results in half‐sites reactivity in mammalian UDPGDHs 6. Asn325{491} in SpUDPGDH is at a position in the alignment that is 80% aspartate. The side chain amide nitrogen of Asn325{491} is 2.8 Å from the side chain carbonyl oxygen of Glu145{262}. Arg327{493} (99%) forms a salt bridge with the pyrophosphate of NAD^+^. The side chain hydroxyl of Ser329{495} (81%) is 2.8 Å from the main chain carbonyl oxygen of Leu317{483} (64%). In a large alignment, the inclusion of even one or a few sequences with variations can lead to critical residues no longer being invariant, but this does not diminish their critical roles, as was demonstrated in ALDHs 32. Three sequences (MCIThaHYPO, SalPacUGD and UncBacHYPO) lacked tyrosine at index 92, while one sequence (NatGarNSD) lacked an aspartate at index 114 and one sequence (UncBacHYPO) that lacked an arginine at index 493. Of these four sequences, two were from uncultured bacteria from metagenomic studies 37, 38 and the other two lacked a reference. Thus, none had proven enzymatic function.

### Tyrosine phosphorylation

It has been revealed that phosphorylation of a tyrosine at index 157 in the alignment in UDPGDHs from *E. coli* (Tyr71) and *Bacillus subtilis* (Tyr70) causes an increase in enzymatic activity 25, 26. Modelling in *B. subtilis* UDPGDH places this tyrosine at the surface near the NAD^+^‐binding site. It has been suggested that phosphorylation of this tyrosine might make this binding site more accessible 25. Tyrosine is not conserved at index 157, with only 8 out of 92 bacterial and archaeal UDPGDHs having tyrosine. Eukaryotic UDPGDHs, where phosphorylation has not been witnessed, have mostly a hydrophobic isoleucine or valine at index 157, while GDPMDHs have valines or phenylalanines here and UDPNAMDHs have an indel at this index.

SaUDPNAMDH is also activated by phosphorylation on Tyr89{183}. This tyrosine in SaUDPNAMDH lies at the bend in a long loop between β‐d and α‐4 near the enzyme surface. This residue lies before Cys92{186}, which may also be involved in regulation by forming a disulphide with the catalytic Cys258{411} 24. Similar to UDPGDHs, this tyrosine at index 183 is not conserved, with only 3 of the 38 UDPNAMDHs aligned having a tyrosine at this position. Thus, it appears that tyrosine phosphorylation did not evolve at conserved tyrosine positions and therefore may not occur in all organisms or enzymes.

### Conserved motifs

**Figure 2:**
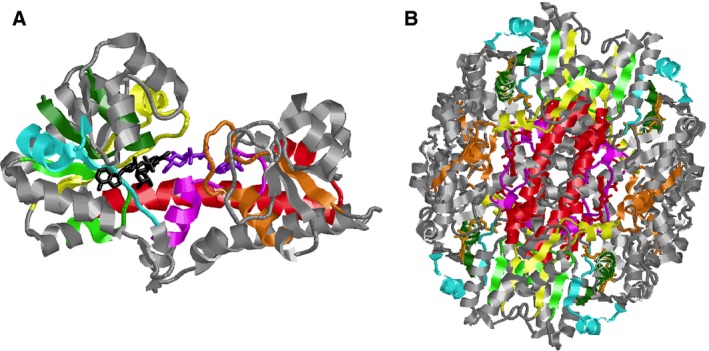
Conserved motifs found in the monomer of SpUDPGDH (A) and a hexamer of PaGDPMDH (B). The colour code for the motifs is identical for both structures. Motifs 4 (yellow), 5 (dark green) and 7 (light blue) make up the NAD ^+^‐binding domain. The Rossmann fold common to dehydrogenases is in Motif 5. 1,4‐dihydro‐NAD ^+^ is shown in black (A). Motifs 1 (red), 2 (pink), 3 (orange) and 4 (yellow) participate in substrate binding. UDP‐GlcUA is shown in purple (A). Motif 1 (red) contains helix α‐9 and functions in domain‐domain interactions (B).

The 10 most well conserved sequence motifs were statistically identified using the meme program. Seventeen of the 18 invariant residues cluster into 7 of the 10 conserved motifs (Table 1). Both the Rossmann fold, found between β‐1 and α‐1 in SpUDPGDH, and Tyr10 are located in Motif 5. The Rossmann fold allows close interaction with the adenosine ribose of NAD^+^ 39. In addition to Motif 5, Motifs 4 and 7 also contribute to the N‐terminal NAD^+^‐binding domain of NDP‐SDHs (Fig. 2A). Motif 4 contains invariant residues Pro140{257} and Glu141{258}. Motif 7 includes the fully conserved Thr83{173}.

**Table 1:** Ten most conserved sequence motifs in nucleotide diphosphate sugar dehydrogenases

Motif Number	Length	Motif	Indices
1	41	[RW][SET][AS]E[MLA][SI]K[LY][AT][AE]N[AT][FY][LR]A[QL][RK][IV][SA][FS][AI]N[ES][LI]SA[IL][CA]EATxG[AIL][DN]VxEV[IA]R	346–387
2	21	K[FY]L[NKQ][PA][GS][FVP]G[FYV]GG[SH]C[LF][PQ]KD[VTP][LK][AN]L	399–419
3	29	[KR]K[IV][AG][IVL][LY]G[LF][AS]FK[PKA][NDG][TS][DG]D[LT]RES[PS][AS][IV]E[IVL][AM][KE]RL	476–504
4	29	F[QN][VI][AL][SF]NPEFL[RA]E[GS]TA[IL]KD[LN]LNP[DS]R[VI][VL][IV]G[GE]	251–280
5	21	MKI[SCA][VCI]IG[LAT]GYVG[LG][PV]T[AC]A[VC][LI]AQ	83–104
6	41	VY[LI]CEAL[NG]LPEVARYWQQVID[MI]NDYQ[RK]RRFAS[RK]I[IV][DE]SLFNT	420–460
7	21	[TS]TDIxEA[IV]K[ED]ADLVFI[ASC]V[PGN]TP	154–174
8	50	[KS]DPYEAC[DR]GAHA[VLI]VI[CL]TEWD[EM]F[KV][ED]xxLDYE[RK]I[HY]K[KS]M[LQ]KPAFIFDGR[RN][VI]LD	558–608
9	15	[KA][ILT]V[VI][EI][KE]STVP[VP][GR][TA][TA]E	210–224
10	21	[KR][IV][DE][LA][IWL]NSGxxxK[SL]PI[YV]EPG[LI][ED]	116–139

Motifs 1–4 are all involved in nucleotide‐sugar substrate binding. Motif 1, which contains four invariant residues (Glu201{349}, Lys204{352}, Asn208{356} and Asn219{367}), is comprised of a long α‐helix (α‐9) that joins the N‐terminal NAD^+^‐binding domain with the C‐terminal domain. This helix also contributes to intersubunit contacts responsible for formation of homodimers, as seen in PaGDPMDH 23 (Fig. 2B) and *Burkholdaria cepacia* UDPGDH 8. Motif 2 contains the fully conserved residues Gly257{408}, Gly258{409}, Cys260{411} and Asp264{415}. Motif 3 contains the invariant residue Lys320{486} and also participates in interactions between subunits b & d, and between subunits a & c in GDPMDH 23.

### Phylogenetic analysis

**Figure 3:**
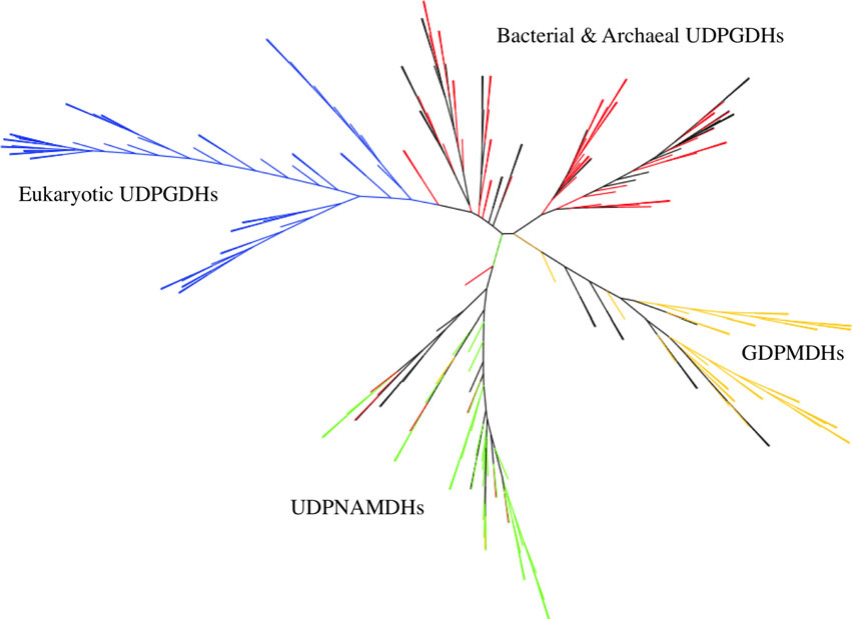
Unrooted bootstrapped phylogenetic tree of nucleotide diphosphate sugar dehydrogenase (NDP‐SDHs). Branches are colour‐coded based on enzyme type: red = Bacterial and Archaeal UDPGDH, blue = Eukaryotic UDPGDH, orange = GDPMDH and green = UDPNAMDH. Uncoloured (black) branches represent sequences solely identified as a NDP‐SDH or a hypothetical protein.

An unrooted bootstrapped phylogenetic tree of NDP‐SDHs (Fig. 3) was generated using the neighbour‐joining method. This method was chosen as maximum likelihood and parsimony methods are computationally prohibitive for larger data sets and as other studies have indicated that the neighbour‐joining method has yielded quality evolutionary relationships in some families 40. In fact, a bootstrapped parsimony tree using only 300 data sets (Fig. S2) was highly comparable to the neighbour‐joining tree using 1000 replicates. The tree was used to support assignment of each NDP‐SDH sequence into an appropriate group for group entropy analysis. The tree indicates that prokaryotic UDPGDHs are the most diverse group of sequences used. Eukaryotic UDPGDHs (823), UDPNAMDHs (995) and GDPMDHs (988) form distinct clades within the phylogenetic tree with high bootstrapping values (in parentheses). The eukaryotic UDPGDHs distinctly cluster within the more diverse prokaryotic UDPGDHs. The UDPNAMDHs and GDPMDHs cluster closely together on the tree, perhaps due to the fact that both substrates involve a mannose sugar (UDP‐ManNAc and GDP‐Man respectively). Among the clade containing 38 UDPNAMDH sequences, there were eight sequences identified as prokaryotic UDPGDHs and two GDPMDHs found in that clade. Literature investigation revealed that all 10 of these sequences resulted from genome sequencing studies, indicating that these outliers could possibly be misidentified without a proven enzymatic function 41, 42, 43, 44, 45, 46.

### Group entropy analysis of GDPMDHs

**Figure 4:**
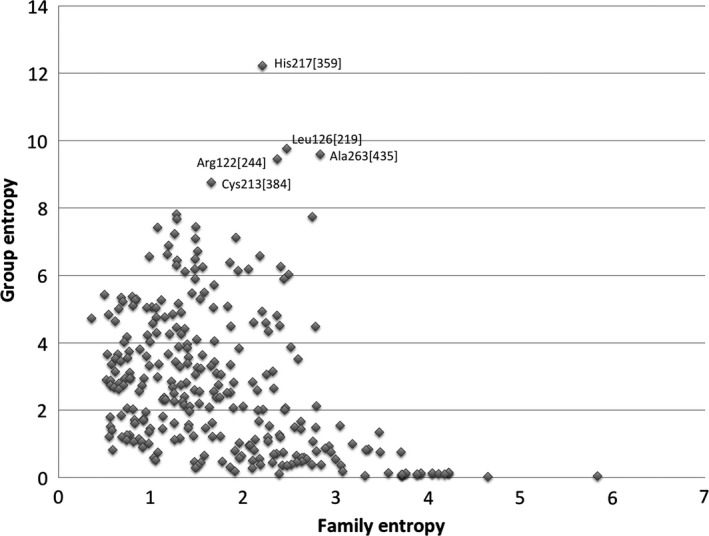
Group entropy analysis plot for GDPMDHs. Residues in the upper left quadrant are unique conservations in GDPMDHs. Residue positions are those in PaGDPMDH with the index position in brackets.

The GEnt program was developed as an algorithm to detect amino acid residues that are characteristic of an individual protein family from an alignment with other related proteins. The program calculates a ‘Group Entropy’ value that represents the degree of residue conservation at that position within the designated group and a ‘Family Entropy’ value that represents the degree of residue conservation at that position within the entire alignment. Residue conservations unique to and critical to the designated group of proteins would have a high Group Entropy value for a specific residue position, indicating it is highly conserved in that group of sequences, while also having a low Family Entropy value, indicating that that position is not as well conserved in the entire alignment. These positions would plot to the upper left quadrant of a Group Entropy vs. Family Entropy plot (Fig. 4). Initial use of the GEnt program was used to identify critical, family‐specific conservations in class 3 ALDHs 47. GEnt was used here to identify novel residue positions important to the unique function each NDP‐SDH homolog.

GEnt analysis of GDPMDHs revealed that residues His217{359}, Leu126{219}, Ala263{406}, Arg122{215} and Cys213{355} (PaGDPMDH residue identities with the alignment index position in curly brackets) have the highest Group Entropy scores (Table 2), indicating that these positions are specifically conserved in the GDPMDHs. The order the residues are listed is descending from highest group entropy. The full GEnt results are available in Table S1. His217{359} was found throughout 43 of the 45 GDPMDH sequences aligned. However, it is possible that these two sequences lacking histidine, GDPMDHs from *Bacillus thuringiensis* and *Vibrio crassostreae*, are misidentified as GDPMDHs, for they both replace histidine at this position with arginine which is invariant in the UDPNAMDHs at this same position. The ND1 position of the His217{359} side chain is 2.9 Å from the 2″‐hydroxyl of the mannose moiety of GDP‐Man, coordinating the substrate 23. Next, Leu126{219} is highly conserved (35 out of 45) in GDPMDHs and is located in a loop following β‐5 which is distant from the active site. With some variations, all other NDP‐SDH groups replace leucine with a proline suggesting that Leu126{219} plays a role in the structure of that loop. With the exception of the same two sequences mentioned above, Ala263{406} is also conserved in 43 of 45 GDPMDHs. All other NDP‐SDH families replace this alanine with a glycine that is 80% conserved in the in the entire alignment. The main chain carbonyl oxygen of Ala263{406} forms a water‐mediated contact to the 2′ hydroxyl of the guanosine ribose of GDP‐Man 23. In SpUDPGDH index 406 is occupied by Gly255{406}, which is 5.6 Å from the oxygen at position 2 of the uridine ring in UDP‐Glc. Thus, Ala263{406} allows for the proper shape of the binding pocket for the nucleotide of GDP‐Man in GDPMDHs. Like His217{359} and Ala263{406}, Arg122{215} was found to be almost fully conserved (43 of 45 sequences) in the GDPMDH group, with the same two previously noted sequences having glutamate at this position. Lysines are found predominantly at index 215 in the rest of the NDP‐SDH alignment. In PaGDPMDH the side chain of Arg122{215} is 2.1 Å from the hydroxyl group of Tyr191{315} (78% conserved in entire alignment and 93% conserved in GDPMDHs) and 2.1 Å from the side chain amide oxygen of Asn155{256}, which lies before the invariant proline‐glutamate sequence (Pro140{257}‐Glu141{258} in SpUDPGDH), aiding in the positioning of that conserved sequence. Lastly, Cys213{355} is partially conserved (25 out of 45) in the GDPMDH group. It is found at the dimer interface region and appears to be associated with subunit contact, as it is located 3.9 Å from Ile245{388} on the neighbouring subunit. Cys213{355} is also 7.3 Å from the 3″ hydroxyl of the bound GDP‐Man.

**Table 2:** Group entropy analysis of GDP‐mannose dehydrogenases (GDPMDHs)

Index	Residue identity^a^	Group entropy	Family entropy	Highest group residue	Common UDPNAMDH residue	Common UDPGDH residue
359	His217	12.231	2.206	His	Arg	Leu
219	Leu126	9.762	2.472	Leu	Pro	Pro
406	Ala263	9.594	2.834	Ala	Gly	Gly
215	Arg122	9.453	2.368	Arg	Lys	Lys
355	Cys213	8.764	1.655	Cys	Glu	Ala

a Residue identity in PaGDPMDH.

### Group entropy analysis of UDPNAMDHs

Group entropy analysis of the UDPNAMDH group (Table 3) found that residues Arg152{259}, Pro155{262}, Arg211{359}, Val261{414}, Val254{407}, His257{410}, Glu117{215}, His242{391} and Phe265{418} (position identities in SaUDPNAMDH with the alignment index position in curly brackets) are unique to the UDPNAMDH group. The full GEnt results are available in Table S2. Arg152{259} is invariant in UDPNAMDHs and is replaced with a phenylalanine in all other groups. Overall, phenylalanine is 78% conserved in the entire alignment at this position. In SaUDPNAMDH the side chain of Arg152{259} is 2.9 Å from the [Eu(DPA)_3_]^3−^ complex bound in the substrate site, which superimposes where the substrate sugar is bound in SpUDPGDH 24. A recent publication of a UDPNAMDH from *Pyrococcus horikoshii* also indicates that Arg152{259} is also found in the substrate‐binding site 46. The main chain carbonyl of Phe142{259} in SpUDPGDH is 4.0 Å from the 4″‐hydroxyl of UDP‐GlcUA in UDPGDH and is located in the glucose‐1‐phosphate‐binding pocket 5. Because of its positioning, Arg152{259} might accommodate for binding a different sugar substrate in UDPNAMDHs. Phe158{259} in PaGDPMDH at this same index position lies at the dimer interface. Next, Pro155{262} is fully conserved within the UDPNAMDH group and in the two possibly misidentified GDPMDH sequences noted above. It is located in a loop following Arg152{259} that makes up the sugar‐binding site, thus possibly providing an altered structure to accommodate a different sugar substrate as well. In all other families, glutamate (76% conserved in the entire alignment) replaces proline at this position. In SpUDPGDH the main chain nitrogen of Glu145{262} at this index position is 2.8 Å from an oxygen atom on the beta phosphate of UDP‐GlcUA. The next residue, Arg211{359}, is also fully conserved in the UDPNAMDH group. The side chain of Arg211{359} is found in the sugar‐binding site of UDPNAMDH. In the *P. horikoshii* UDPNAMDH the NE atom of Arg211{359} hydrogen bonds to the O2A atom of UDP‐ManNAcA 48. In fact two arginines, Arg152{259} and Arg211{359}, identified by GEnt in UDPNAMDHs are both involved in substrate specificity. In support of this observation, a R152F/R211L double mutant of SaUDPNAMDH is unable to oxidize the normal UDP‐ManNAc substrate 24.

**Table 3:** Group entropy analysis of UDPNAMDHs

Index	Residue identity^a^	Group entropy	Family entropy	Highest group residue	Common GDPMDH residue	Common UDPGDH residue
259	Arg152	12.777	3.483	Arg	Phe	Phe
262	Pro155	11.128	2.950	Pro	Glu	Glu
359	Arg211	10.593	2.206	Arg	His	Leu
414	Val261	10.251	2.633	Val	Lys	Lys
407	Val254	10.061	2.55	Val	Phe	Phe, Tyr
410	His257	9.943	2.458	His	Ser	Ser, Tyr
215	Glu117	9.924	2.368	Glu	Arg	Lys
391	His242	9.609	3.001	His	Asp	Asp
418	Phe265	9.595	1.330	Phe	Ala, Gly	Ala, Asn, Gln

a Residue identity in SaUDPNAMDH.

The next residue identified by GEnt in UDPNAMDHs is Val261{414} which is found in 34 out of 38 UDPNAMDH sequences (89% conserved), with the other four UDPNAMDH sequences having either leucine or isoleucine. This index position is mostly replaced by lysine in the entire alignment (78% conserved). This position is adjacent to fully conserved Asp262{415} and is in close proximity to the conserved ‘GGXC’ sequence involving the catalytic thiol. The side chain of Val261{414} does not face the catalytic site and lies about 5.5 Å from the side chain of Ile224{372} from the neighbouring subunit. Hence, this position in UDPNAMDH may play a role in intersubunit contact. In SpUDPGDH Val261{414} is replaced by Lys263{414}, which is 2.9 Å from the 2′ hydroxyl group of the nicotinamide ribose of NAD^+^. Another valine identified by GEnt in SaUDPNAMDH is Val254{407} which is found in 35 out of 38 UDPNAMDH sequences, with the other three UDPNAMDH sequences having leucine. In the *P. horikoshii* UDPNAMDH Val254{407} lines the pocket where the uridine group of UDP‐ManNAcA is located 48. Similarly, the α‐carbons of Tyr256{407} in SpUDPGDH and Phe264{407} in PaGDPMDH are within 5 Å of C1D of the ribose ring of UDP‐xylopyranose and GDP‐mannopyranosyl ester, respectively. Hence, the main chain position of this residue contributes to NDP binding in the substrate. Next, His257{410}, which is invariant in UDPNAMDHs, is located between two invariant glycines and the invariant cysteine in the sequence ‘GGHC’. The side chain of His257{410} is about 4 Å from the nicotinamide ring of NAD^+^ and approximately 5.5 Å from the 2′ and 3′ hydroxyls of the nicotinamide ribose in SaUDPNAMDH. In the *P. horikoshii* UDPNAMDH His257{410} side chain hydrogen bonds to a water molecule that in turn is bonded to the O2B atom of UDP‐ManNAcA 48. Tyr259{410} at the corresponding position in SpUDPGDH is approximately 4.5 Å from the 2′ and 3′ hydroxyl groups of the nicotinamide ribose of NAD^+^.

The next residue identified by GEnt in UDPNAMDH is Glu117{215}, which is invariant in the UDPNAMDH group and is mostly replaced with lysine or arginine in other groups. The side chain carbonyl of Glu117{215} in SaUDPNAMDH is 2.6 Å from the side chain hydroxyl of Tyr184{315}, which is also invariant in UDPNAMDH. The side chain amine of Lys116{215} in SpUDPGDH is 2.8 Å from the main chain carbonyl of invariant Pro140{257}, which is located in the loop between β‐8 and α‐7. Next, His242{391} is found in 35 out of 38 UDPNAMDH sequences (92% conserved) and is replaced in other NDP‐SDH groups by aspartate, which is 78% conserved in the entire alignment. The side chain of His242{391} forms an ion pair with Glu207{355} from the neighbouring subunit 48 and also lies 3.8 Å from the [Eu(DPA)_3_]^3−^ complex bound in the substrate site of that neighbouring subunit in SaUDPNAMDH. It is possible that His242{391} in UDPNAMDH makes a similar intersubunit contact as the conserved Arg244{393} in SpUDPGDH (note that the residues are only two index positions away). In fact, UDPNAMDHs would have two intersubunit contacts with their substrate, His242{391} and Arg244{393}, which lies 3.2 Å from the [Eu(DPA)_3_]^3−^ complex in SaUDPNAMDH and 2.8 Å from O7″ and 2.9 Å from O3″ of UDP‐ManNAcA in *P. horikoshii* UDPNAMDH 48. Interestingly, Arg244{393} in SpUDPGDH interacts with the 2″ hydroxyl of glucose in UDP‐Glc. Glucose and mannose are epimers at the 2″ positions. GDPMDH have Lys250{393} at this index position, but it does not form intersubunit contacts due to the domain‐swapped structure of GDPMDH 23. Lastly, the side chain of Phe265{418} in subunit b of SaUDPNAMDH is 4.3 Å from the side chain of Ile224{372} from subunit a. Thus, in UDPNAMDHs this position plays a role in intersubunit contact. Phenylalanine is found at index 418 in 35 out of 38 UDPNAMDH sequences, with the other three UDPNAMDH sequences having tyrosine. However, Gln267{418} in SpUDPGDH, Ala275{418} in PaGDPMDH and Asn283{418} in hUDPGDH at this index position all lie in the middle of α‐11 and do not form any apparent intermolecular contacts.

### Group entropy analysis of UDPGDHs

The UDPGDH GEnt analysis (Table 4) indicated that the residues Leu211{359}, Phe218{366}, Ile27{112}, Lys116{215}, Ser8{90}, Ala207{355} and Gly238{387} in SpUDPGDH (index positions in curly brackets) are uniquely conserved in the UDPGDH group. The full GEnt results are available in Table S3. Leu211{359}, which is 93% conserved in the UDPGDHs, forms the pocket for the sugar group of UDP‐Glc, making van der Waals contact with the C2″ ring position 23. Second, Phe218{366} in the UDPGDHs lies at a hydrophobic position. The side chain of Phe218{366} is roughly 4.0 Å from Ile245{394} indicating that its function might be hydrophobic packing. Next, Ile27{112} is commonly replaced with alanine, cysteine and valine in the UDPGDH group. It is located 5.0 Å from the Rossmann fold and likely serves in structural positioning. The side chain hydroxyl group of Ser8{90} is 4.5 Å from the gamma carbon of Ile27{112}. This close interaction may lead to a compensatory change in other NDP‐SDHs with index 90 being a hydrophobic amino acid, often leucine, and index 112 being a smaller residue, often glycine, to facilitate packing interactions. For example, PaGDPMDH has a hydrophobic leucine (Leu8) at index 90 and a glycine (Gly28) at index position 112.

**Table 4:** Group entropy analysis of UDPGDHs

Index	Residue identity^a^	Group entropy	Family entropy	Highest group residue	Common GDPMDH residue	Common UDPNAMDH residue
359	Leu211	12.577	2.206	Leu	His	Arg
366	Phe218	10.130	1.683	Phe	Ala	Ala
112	Ile27	10.031	1.679	Cys	Gly	Gly
215	Lys116	9.458	2.368	Lys	Arg	Glu
90	Ser8	9.421	1.298	Ala	Leu	Leu
355	Ala207	8.211	1.655	Ala	Cys	Glu
387	Gly238	7.830	1.277	Gly	Val	Leu

a Residue identity in SpUDPGDH.

The side chain amine of Lys116{215}, which is 92% conserved in the UDPGDH group, is 2.8 Å from the main chain carbonyl of the invariant Pro140{257}. This interaction likely coordinates the position of the critical loop that contains both Pro140{257} and Glu141{258}. Next, the side chain of Ala207{355}, which is 81% conserved in UDPGDHs, is 6.8 Å from the 3″ hydroxyl of the bound UDP‐xylopyranose in SpUDPGDH. This position in PaGDPMDH is occupied by Cys213{355} which lies at the dimer interface region. Lastly, Gly238{387} is mostly glycine and alanine in the bacterial and archaeal UDPGDHs and eukaryotic UDPGDHs, respectively. This index position is exchanged with mostly hydrophobic residues in other NDP‐SDH groups. Val244{387} lies at this position in PaGDPMDH and is involved in subunit interactions.

### Common group entropy positions

Several index positions identified by GEnt demonstrated group‐specific conservations in multiple NDP‐SDH groups, yielding novel insights into the critical differences between each enzyme. First, index position 359 is the highest scoring position for group entropy in GDPMDHs and UDPGDHs, and is also highly scoring in UDPNAMDHs. This position is clearly responsible for substrate specificity, as was initially proposed by Snook and colleagues 23. The side chain of His217{359} in PaGDPMDH is 2.9 Å from the 2″‐hydroxyl of the mannose moiety of GDP‐Man, coordinating the substrate 23. Leu211{359} in SpUDPGDH forms the pocket for the sugar group of UDP‐glucose, making van der Waals contact with the C2″ ring position 23. As previously noted, glucose and mannose are epimers at the 2″ positions, and the *N*‐acetyl group of UDP‐ManNAc is also attached to the 2″ position. Hence, this should be a key location for determining substrate specificity. The side chain of Arg211{359} in SaUDPNAMDH is also found in the sugar‐binding site of UDPNAMDH. However, the specific substrate interaction that Arg211{359} has is not clear, as a [Eu(DPA)_3_]^3−^ complex, instead specific substrate, was crystalized. The recently published *P. horikoshii* UDPNAMDH structure shows that the guanidinium group of Arg211{359} hydrogen bonds to O2A and O1A of the α‐phosphate of UDP‐ManNAcA, while a different arginine, the conserved Arg244{393}, hydrogen bonds to the carbonyl oxygen of the *N*‐acetyl group of UDP‐ManNAcA 48.

Second, index position 215 was also identified by GEnt with high group entropy scores in all three NDP‐SDH groups. This position apparently serves to maintain critical enzyme structure in each group by interacting with a conserved tyrosine. The side chain of Arg122{215} in PaGDPMDH is 2.1 Å from the hydroxyl group of Tyr191{315} (78% conserved in entire alignment) and 2.1 Å from the side chain amide oxygen of Asn155{256}, which lies before the invariant proline{257}‐glutamate{258} sequence, aiding in the positioning of that conserved loop between β‐8 and α‐7. The side chain carbonyl of Glu117{215} in SaUDPNAMDH is 2.6 Å from the side chain hydroxyl of Tyr184{315}, which is also invariant in UDPNAMDH. In SpUDPGDH the side chain of Lys116{215} is 2.8 Å from the main chain carbonyl of invariant Pro140{257}, also holding the same loop in place. Lys116{215} does not interact with the conserved tyrosine at index 315, however, as it is replaced by Leu181{315} in SpUDPGDH. However, in hUDPGDH the side chain Lys129{215} does interact with the side chain of Tyr199{315}, as seen in these other NDP‐SDHs.

Lastly, index 355 is identified in the top six group entropy scores for both GDPMDH and UDPGDH, and is the sixteenth highest group entropy score in UDPNAMDH. Index 355 appears critical for intersubunit contact. The side chain of Ala207{355} in the monomeric SpUDPGDH structure lies 6.8 Å from the 3″ hydroxyl of the bound UDP‐xylopyranose. The side chain of the equivalent residue in hUDPGDH, Ala223{355}, is 7.0 Å from the 3′ hydroxyl of UDP‐Glc, but is 4.0 Å from Ile255{388} in the neighbouring subunit. In PaGDPMDH the side chain of Cys213{355} is located 3.9 Å from Ile245{388} on the neighbouring subunit and is also 7.3 Å from the 3″ hydroxyl of the bound GDP‐Man. In SaUDPNAMDH the side chain carbonyl of Glu207{355} is 4.5 Å from the [Eu(DPA)_3_]^3−^ complex, which sits in the substrate‐binding site, and is 3.9 Å from His242{391}, also identified by GEnt (see above), from the neighbouring subunit. A glutamate at index 355 also lies in the binding site for UDP‐ManNAcA in the *P. horikoshii* UDPNAMDH. The overall and group‐specific conservations identified here could definitely serve as interesting targets for site‐directed mutagenesis by other researchers. The identification of these positions may also aid in drug discovery for bacterial isoforms that assist in capsule formation.

## Materials and methods

The project initially began by obtaining the amino acid sequence of UDPGDH from *Streptococcus pyogenes* (PDB entries 1DLJ and 1DLI) from the RCSB Protein Data Bank. The sequence was then used to perform a psi‐blast 49 search of the nonredundant protein database at the National Center for Biotechnology Information (NCBI). 229 related UDPGDH, GDPMDH and UDPNAMDH amino acid sequences were collected with per cent identities ranging from 99% to 15%. These sequences were initially aligned using T‐Coffee 50. To improve alignment quality, the alignment was manually adjusted using tertiary structure comparison through the RCSB PDB Protein Comparison Tool‐jFATCAT method 51, 52 as a guide, comparing *Streptococcus pyogenes* UDPGDH (SpUDPGDH, PDB entry 1DLJ), *Pseudomonas aeruginosa* GDPMDH (PaGDPMDH, PDB entry 1MV8), human UDPGDH (hUDPGDH, PDB entry 3TDK) and *Staphylococcus aureus* UDPNAMDH (SaUDPNAMDH, PDB entry 3OJL). The alignment editor used was genedoc 53. Conservations within the alignment were analysed for structural or functional significance. Molecular visualization was performed using rasmol 54. Analysis of conserved sequence motifs was facilitated by meme program 55. Group entropy analysis (GEnt) 47 was performed to compare UDPGDH, UDPNAMDH and GDPMDH groups to each other.

The phylip suite of programs was used to generate the phylogenetic tree 56. First, the alignment was trimmed using TrimAl 57. 1000 Bootstrapped data sets of the trimmed alignment were then generated using the seqboot program. Next, distances for the data sets were determined by the protdist program using the Jones‐Taylor‐Thornton matrix. Phylogenetic trees for each data set were generated using the NEIGHBOR program. Lastly, the unrooted consensus tree was generated using the CONSENSE program. The tree graphic was generated using figtree (available at http://tree.bio.ed.ac.uk/software/figtree).

## Author contributions

NF performed the alignment of 229 NDP‐SDH sequences, analysed the alignment for functional, structural and phylogenetic conservations, and drafted the manuscript. PN conceived the initial project and carried out an initial analysis of an NDP‐SDH alignment of 100 NDP‐SDH sequences. JP supervised the project, participated in the analysis of both alignments, helped to write the initial and final drafts of the manuscript and addressed reviewer's comments.

## Supporting information


**Fig. S1.** Complete alignment of 229 NDP‐SDHs sequences (MSF format).

**Fig. S2.** Bootstrapped parsimony tree of NDP‐SDHs.

**Table S1.** Complete GEnt results of GDPMDHs.

**Table S2.** Complete GEnt results of UDPNAMDHs.

**Table S3.** Complete GEnt results of UDPGDHs.
