# Supplementary material for: Analysis of nucleotide diphosphate sugar dehydrogenases reveals family and group‐specific relationships
Source: FEBS Open Bio. 2016 Jan 11;6(1):77–89. doi: 10.1002/2211-5463.12022 (PMC4794789; doi:10.1002/2211-5463.12022)
Supplement: Supplementary file 1 — Fig. S1. Complete alignment of 229 NDP‐SDHs sequences (MSF format). [file FEB4-6-77-s001.docx]

Figure S1. Complete alignment of 229 NDP-SDHs sequences (MSF format).

NSDFINALPS.msf MSF: 655 Type: P January 30, 2015 03:12 Check: 5498 ..

Name: StrPyoUGD Len: 655 Check: 9769 Weight: 1.00

Name: HymAerUGD1 Len: 655 Check: 7527 Weight: 1.00

Name: MetCapUGD1 Len: 655 Check: 8290 Weight: 1.00

Name: ActGloUGD6 Len: 655 Check: 2018 Weight: 1.00

Name: HalZhaHYPO Len: 655 Check: 3220 Weight: 1.00

Name: MCIThaHYPO Len: 655 Check: 6896 Weight: 1.00

Name: SalPacUGD Len: 655 Check: 9401 Weight: 1.00

Name: NatGarNSD2 Len: 655 Check: 143 Weight: 1.00

Name: BacSubUGD2 Len: 655 Check: 1535 Weight: 1.00

Name: AzoTolUGD Len: 655 Check: 1757 Weight: 1.00

Name: LacCreEPS Len: 655 Check: 2669 Weight: 1.00

Name: AlaProUG Len: 655 Check: 6710 Weight: 1.00

Name: StrZooUG Len: 655 Check: 4100 Weight: 1.00

Name: StrHGBNSD Len: 655 Check: 2576 Weight: 1.00

Name: NatGarNSD Len: 655 Check: 6632 Weight: 1.00

Name: TheMelUGD Len: 655 Check: 5982 Weight: 1.00

Name: SulDenUGD Len: 655 Check: 3434 Weight: 1.00

Name: SphAlaUGD Len: 655 Check: 8753 Weight: 1.00

Name: SheOneUGD Len: 655 Check: 2103 Weight: 1.00

Name: SalSerUGD Len: 655 Check: 264 Weight: 1.00

Name: PseHalUGD Len: 655 Check: 4259 Weight: 1.00

Name: ProMirUGD Len: 655 Check: 8896 Weight: 1.00

Name: ParDisUGD Len: 655 Check: 3623 Weight: 1.00

Name: ParDisUGD1 Len: 655 Check: 338 Weight: 1.00

Name: LacSalUGD Len: 655 Check: 3123 Weight: 1.00

Name: CloPerUGD Len: 655 Check: 9977 Weight: 1.00

Name: ArcButUGD Len: 655 Check: 1720 Weight: 1.00

Name: AerHydUGD Len: 655 Check: 3352 Weight: 1.00

Name: LacJohSBP Len: 655 Check: 8861 Weight: 1.00

Name: StrUbeUGD Len: 655 Check: 9472 Weight: 1.00

Name: ReiMEDNSD Len: 655 Check: 8532 Weight: 1.00

Name: ExiSibNSD Len: 655 Check: 3701 Weight: 1.00

Name: RumObeHYPO Len: 655 Check: 1406 Weight: 1.00

Name: RumObeHYP1 Len: 655 Check: 8704 Weight: 1.00

Name: FaePraHYPO Len: 655 Check: 5735 Weight: 1.00

Name: EubDolHYPO Len: 655 Check: 5915 Weight: 1.00

Name: BacPleHYPO Len: 655 Check: 2481 Weight: 1.00

Name: BacCopHYPO Len: 655 Check: 8313 Weight: 1.00

Name: StrTheESP Len: 655 Check: 530 Weight: 1.00

Name: StrIniCPS Len: 655 Check: 9099 Weight: 1.00

Name: SerProUGD Len: 655 Check: 2556 Weight: 1.00

Name: GeoMetUGD Len: 655 Check: 1752 Weight: 1.00

Name: GeoUraUGD Len: 655 Check: 2641 Weight: 1.00

Name: AnoFlaUGD Len: 655 Check: 9455 Weight: 1.00

Name: PorGinNSD Len: 655 Check: 8323 Weight: 1.00

Name: BacSelRPON Len: 655 Check: 4318 Weight: 1.00

Name: PelPhaNSD Len: 655 Check: 4913 Weight: 1.00

Name: MetPopNSD Len: 655 Check: 8089 Weight: 1.00

Name: MetExtNSD Len: 655 Check: 2183 Weight: 1.00

Name: VibVulPRE Len: 655 Check: 628 Weight: 1.00

Name: GeoKauNSD Len: 655 Check: 5886 Weight: 1.00

Name: RhiTriEXO Len: 655 Check: 4051 Weight: 1.00

Name: ParDisUGD2 Len: 655 Check: 2722 Weight: 1.00

Name: XanAxoUGD Len: 655 Check: 1770 Weight: 1.00

Name: XanCamUGD Len: 655 Check: 9659 Weight: 1.00

Name: EntFaeUGD Len: 655 Check: 3378 Weight: 1.00

Name: StrPneUGD2 Len: 655 Check: 5531 Weight: 1.00

Name: StrPneUGD3 Len: 655 Check: 7770 Weight: 1.00

Name: StrPneUG Len: 655 Check: 8824 Weight: 1.00

Name: EscAlbUGD Len: 655 Check: 2022 Weight: 1.00

Name: BacTheUGD Len: 655 Check: 8404 Weight: 1.00

Name: SacSpiUGD Len: 655 Check: 6024 Weight: 1.00

Name: Aer159PBP Len: 655 Check: 9348 Weight: 1.00

Name: ButSynUGD Len: 655 Check: 1238 Weight: 1.00

Name: BraJapUGD Len: 655 Check: 9577 Weight: 1.00

Name: SynCC9UGD Len: 655 Check: 8730 Weight: 1.00

Name: StePneUGD Len: 655 Check: 3039 Weight: 1.00

Name: SalAreUGD Len: 655 Check: 6278 Weight: 1.00

Name: BurYI2NSD Len: 655 Check: 8875 Weight: 1.00

Name: BacCerNSD Len: 655 Check: 6378 Weight: 1.00

Name: PaeSanHYPO Len: 655 Check: 5756 Weight: 1.00

Name: Rhi42MUGD Len: 655 Check: 2645 Weight: 1.00

Name: OpiTAV5NSD Len: 655 Check: 8198 Weight: 1.00

Name: MarHTCUGD8 Len: 655 Check: 5723 Weight: 1.00

Name: BacSubUGD Len: 655 Check: 4155 Weight: 1.00

Name: NocKunUGD Len: 655 Check: 2331 Weight: 1.00

Name: DesHafUGD Len: 655 Check: 2513 Weight: 1.00

Name: BacThuUGD Len: 655 Check: 4581 Weight: 1.00

Name: SphJapUGD Len: 655 Check: 438 Weight: 1.00

Name: RicSibUGD Len: 655 Check: 5072 Weight: 1.00

Name: MetCapUGD Len: 655 Check: 8290 Weight: 1.00

Name: HymAerUGD Len: 655 Check: 7527 Weight: 1.00

Name: BacSubUGD7 Len: 655 Check: 4039 Weight: 1.00

Name: ActGloUDG9 Len: 655 Check: 1457 Weight: 1.00

Name: NatGarNSD5 Len: 655 Check: 6632 Weight: 1.00

Name: UncBacHYPO Len: 655 Check: 8344 Weight: 1.00

Name: HalZhaHYPP Len: 655 Check: 3220 Weight: 1.00

Name: StrPneUGD1 Len: 655 Check: 7788 Weight: 1.00

Name: HoePhoUGD Len: 655 Check: 9886 Weight: 1.00

Name: CapSpuUGD Len: 655 Check: 3943 Weight: 1.00

Name: NovAroUGD Len: 655 Check: 2951 Weight: 1.00

Name: ProMarUGD Len: 655 Check: 6287 Weight: 1.00

Name: HomSapUGD Len: 655 Check: 3990 Weight: 1.00

Name: MusMusUNA Len: 655 Check: 3082 Weight: 1.00

Name: DanRerUGD Len: 655 Check: 5626 Weight: 1.00

Name: XenLaeUGD Len: 655 Check: 2697 Weight: 1.00

Name: RatNorUGD Len: 655 Check: 3918 Weight: 1.00

Name: PonAbeUGD Len: 655 Check: 4710 Weight: 1.00

Name: BosTauUGD3 Len: 655 Check: 3565 Weight: 1.00

Name: BamOldUGD Len: 655 Check: 9141 Weight: 1.00

Name: PedCorUGD Len: 655 Check: 8402 Weight: 1.00

Name: ZeaMayUGD8 Len: 655 Check: 9938 Weight: 1.00

Name: StrPurUGDP Len: 655 Check: 4059 Weight: 1.00

Name: CioIntUGDP Len: 655 Check: 6619 Weight: 1.00

Name: NemVecPPRE Len: 655 Check: 1233 Weight: 1.00

Name: CaeEleUGD Len: 655 Check: 752 Weight: 1.00

Name: PopTomUGD Len: 655 Check: 2202 Weight: 1.00

Name: DroMelUGD Len: 655 Check: 2354 Weight: 1.00

Name: AraThaUGD Len: 655 Check: 4514 Weight: 1.00

Name: DroMelUGD1 Len: 655 Check: 2389 Weight: 1.00

Name: NasVitUDG Len: 655 Check: 1357 Weight: 1.00

Name: CaeBriHYPO Len: 655 Check: 366 Weight: 1.00

Name: DroWilGK3 Len: 655 Check: 3505 Weight: 1.00

Name: NasVitUGDP Len: 655 Check: 2354 Weight: 1.00

Name: DroVirGJ3 Len: 655 Check: 1936 Weight: 1.00

Name: HomSapUGD2 Len: 655 Check: 4489 Weight: 1.00

Name: XenTroUGD Len: 655 Check: 7012 Weight: 1.00

Name: FlaCF1UGD Len: 655 Check: 4204 Weight: 1.00

Name: ZeaMayUGD Len: 655 Check: 9105 Weight: 1.00

Name: MayZebUGD1 Len: 655 Check: 5684 Weight: 1.00

Name: VitvinUGD1 Len: 655 Check: 1373 Weight: 1.00

Name: HomSapUGD1 Len: 655 Check: 178 Weight: 1.00

Name: OviAriUGD1 Len: 655 Check: 3845 Weight: 1.00

Name: FelCatUGD1 Len: 655 Check: 3536 Weight: 1.00

Name: EquCabUGD1 Len: 655 Check: 4332 Weight: 1.00

Name: CanFamUGD1 Len: 655 Check: 4232 Weight: 1.00

Name: DroMojGI7 Len: 655 Check: 1422 Weight: 1.00

Name: DroPseGA34 Len: 655 Check: 2738 Weight: 1.00

Name: HomSapICRA Len: 655 Check: 4489 Weight: 1.00

Name: ColLivUGD Len: 655 Check: 8701 Weight: 1.00

Name: CamFloUGD Len: 655 Check: 7515 Weight: 1.00

Name: BosTauUGD Len: 655 Check: 3565 Weight: 1.00

Name: MelGal1UGD Len: 655 Check: 5231 Weight: 1.00

Name: AilMelUGDP Len: 655 Check: 4012 Weight: 1.00

Name: MusFurUGDP Len: 655 Check: 4121 Weight: 1.00

Name: MusFur1UGD Len: 655 Check: 8482 Weight: 1.00

Name: MelUnd1UGD Len: 655 Check: 3356 Weight: 1.00

Name: FalPer1UGD Len: 655 Check: 4381 Weight: 1.00

Name: ChrBel2UGD Len: 655 Check: 3361 Weight: 1.00

Name: FelCat1UGD Len: 655 Check: 3536 Weight: 1.00

Name: SaiBolUGDP Len: 655 Check: 1285 Weight: 1.00

Name: DroWilGK1 Len: 655 Check: 3505 Weight: 1.00

Name: CaeRemQV40 Len: 655 Check: 2068 Weight: 1.00

Name: CaeBriQV40 Len: 655 Check: 366 Weight: 1.00

Name: ZeaMayUGDI Len: 655 Check: 9938 Weight: 1.00

Name: GalGalUG Len: 655 Check: 4594 Weight: 1.00

Name: CaeEleUG Len: 655 Check: 846 Weight: 1.00

Name: PseAerGMD Len: 655 Check: 4405 Weight: 1.00

Name: CanCloGM Len: 655 Check: 819 Weight: 1.00

Name: VibCraGMD Len: 655 Check: 676 Weight: 1.00

Name: PseSyrGMD Len: 655 Check: 2847 Weight: 1.00

Name: AmyAzuGMD Len: 655 Check: 2305 Weight: 1.00

Name: OxaAB1GMD8 Len: 655 Check: 353 Weight: 1.00

Name: ActGloGDM Len: 655 Check: 5881 Weight: 1.00

Name: AmyAlbGDM7 Len: 655 Check: 5820 Weight: 1.00

Name: AmyBalGDM Len: 655 Check: 5495 Weight: 1.00

Name: PseDC3GMD5 Len: 655 Check: 2847 Weight: 1.00

Name: PseVerGMD1 Len: 655 Check: 3007 Weight: 1.00

Name: PseQDAGMD1 Len: 655 Check: 6045 Weight: 1.00

Name: PseAerGMD1 Len: 655 Check: 4778 Weight: 1.00

Name: PseHYSGMD1 Len: 655 Check: 1924 Weight: 1.00

Name: AmyAlbGMD6 Len: 655 Check: 5820 Weight: 1.00

Name: AmyAzuGMD1 Len: 655 Check: 3935 Weight: 1.00

Name: AlgSagHYPO Len: 655 Check: 6057 Weight: 1.00

Name: AciKBSHYPO Len: 655 Check: 2526 Weight: 1.00

Name: AmyBalGMD Len: 655 Check: 5495 Weight: 1.00

Name: StrCanGMD1 Len: 655 Check: 9221 Weight: 1.00

Name: PseAlcGMD1 Len: 655 Check: 8127 Weight: 1.00

Name: ActGloGMD6 Len: 655 Check: 5881 Weight: 1.00

Name: OxaAB1GMD1 Len: 655 Check: 353 Weight: 1.00

Name: PseAerUNPP Len: 655 Check: 3705 Weight: 1.00

Name: HahGanHYPO Len: 655 Check: 2752 Weight: 1.00

Name: DesSalNSD Len: 655 Check: 2920 Weight: 1.00

Name: PseTomGMD1 Len: 655 Check: 2555 Weight: 1.00

Name: MarAlgGMD Len: 655 Check: 3079 Weight: 1.00

Name: PseSyrGMD3 Len: 655 Check: 2847 Weight: 1.00

Name: NovAroGMD Len: 655 Check: 7190 Weight: 1.00

Name: AzoSpGMD Len: 655 Check: 110 Weight: 1.00

Name: AzoVinGMD Len: 655 Check: 8305 Weight: 1.00

Name: BacThuGMD Len: 655 Check: 6092 Weight: 1.00

Name: HahGanHYPP Len: 655 Check: 2752 Weight: 1.00

Name: AlgSagHYPP Len: 655 Check: 6057 Weight: 1.00

Name: AciKBSHYPP Len: 655 Check: 2526 Weight: 1.00

Name: PseSyrGMD2 Len: 655 Check: 1938 Weight: 1.00

Name: PseSyrGMD1 Len: 655 Check: 1938 Weight: 1.00

Name: StrCanGMD Len: 655 Check: 9221 Weight: 1.00

Name: PseVerGMD Len: 655 Check: 3007 Weight: 1.00

Name: PseQDAGMD Len: 655 Check: 6045 Weight: 1.00

Name: PseHYSGMD Len: 655 Check: 1924 Weight: 1.00

Name: PseAlcGMD Len: 655 Check: 8127 Weight: 1.00

Name: PseAerUPP Len: 655 Check: 3705 Weight: 1.00

Name: StaAurUMD Len: 655 Check: 8187 Weight: 1.00

Name: EscColUMND Len: 655 Check: 1047 Weight: 1.00

Name: LusFleUGD Len: 655 Check: 68 Weight: 1.00

Name: SalEntUMD Len: 655 Check: 7223 Weight: 1.00

Name: ActSerUG Len: 655 Check: 2845 Weight: 1.00

Name: ErwTasUG Len: 655 Check: 1889 Weight: 1.00

Name: FusUlcUMD Len: 655 Check: 2093 Weight: 1.00

Name: CorMarUMD Len: 655 Check: 3641 Weight: 1.00

Name: StaVitUMD Len: 655 Check: 6775 Weight: 1.00

Name: SheVioUMD Len: 655 Check: 8023 Weight: 1.00

Name: SerS4UMD Len: 655 Check: 5202 Weight: 1.00

Name: PseStuUMD Len: 655 Check: 4466 Weight: 1.00

Name: PsePseUMD Len: 655 Check: 5660 Weight: 1.00

Name: PseFluUMD Len: 655 Check: 6975 Weight: 1.00

Name: HalSalUMD Len: 655 Check: 3745 Weight: 1.00

Name: FraPhiUMD Len: 655 Check: 8625 Weight: 1.00

Name: EdwC07UMD Len: 655 Check: 2618 Weight: 1.00

Name: AerAquUMD Len: 655 Check: 5614 Weight: 1.00

Name: GeoWSUUMD Len: 655 Check: 7967 Weight: 1.00

Name: EscMG1UMD Len: 655 Check: 1047 Weight: 1.00

Name: DicZeaUMD Len: 655 Check: 3281 Weight: 1.00

Name: VibOrdUMD Len: 655 Check: 3146 Weight: 1.00

Name: VidVulUMaD Len: 655 Check: 3079 Weight: 1.00

Name: KlePneUMaD Len: 655 Check: 969 Weight: 1.00

Name: GeoTheUMaD Len: 655 Check: 1890 Weight: 1.00

Name: BurXenUMaD Len: 655 Check: 1113 Weight: 1.00

Name: HasParUMaD Len: 655 Check: 8913 Weight: 1.00

Name: RalSolUMaD Len: 655 Check: 9154 Weight: 1.00

Name: PolIrgUMaD Len: 655 Check: 6012 Weight: 1.00

Name: RalSolNGD Len: 655 Check: 9444 Weight: 1.00

Name: YerEntUMaD Len: 655 Check: 9383 Weight: 1.00

Name: EscFerUMaD Len: 655 Check: 7452 Weight: 1.00

Name: OceGraUMaD Len: 655 Check: 1500 Weight: 1.00

Name: BacNRRUMaD Len: 655 Check: 5235 Weight: 1.00

Name: GeoKauUMaD Len: 655 Check: 7894 Weight: 1.00

Name: PsyCNPUMaD Len: 655 Check: 7256 Weight: 1.00

Name: MorPE3UMaD Len: 655 Check: 4340 Weight: 1.00

Name: SerProUMaD Len: 655 Check: 8150 Weight: 1.00

//

1 50

StrPyoUGD .......... .......... .......... .......... ..........

HymAerUGD1 .......... .......... .......... .......... ..........

MetCapUGD1 .......... .......... .......... .......... ..........

ActGloUGD6 .......... .......... .......... .......... ..........

HalZhaHYPO .......... .......... .......... .......... ..........

MCIThaHYPO .......... .......... .......... .......... ..........

SalPacUGD .......... .......... .......... .......... ..........

NatGarNSD2 .......... .......... .......... .......... ..........

BacSubUGD2 .......... .......... .......... .......... ..........

AzoTolUGD .......... .......... .......... .......... ..........

LacCreEPS .......... .......... .......... .......... ..........

AlaProUG .......... .......... .......... .......... ..........

StrZooUG .......... .......... .......... .......... ..........

StrHGBNSD .......... .......... .......... .......... ..........

NatGarNSD .......... .......... .......... .......... ..........

TheMelUGD .......... .......... .......... .......... ..........

SulDenUGD .......... .......... .......... .......... ..........

SphAlaUGD .......... .......... .......... .......... ..........

SheOneUGD .......... .......... .......... .......... ..........

SalSerUGD .......... .......... .......... .......... ..........

PseHalUGD .......... .......... .......... .......... ..........

ProMirUGD .......... .......... .......... .......... ..........

ParDisUGD .......... .......... .......... .......... ..........

ParDisUGD1 .......... .......... .......... .......... ..........

LacSalUGD .......... .......... .......... .......... ..........

CloPerUGD MKKLSLSLLA KKVIESRKEK NITQQQLADL TNINRAMISR LESMDYIPSI

ArcButUGD .......... .......... .......... .......... ..........

AerHydUGD .......... .......... .......... .......... ..........

LacJohSBP .......... .......... .......... .......... ..........

StrUbeUGD .......... .......... .......... .......... ..........

ReiMEDNSD .......... .......... .......... .......... ..........

ExiSibNSD .......... .......... .......... .......... ..........

RumObeHYPO .MKKLSMTRL AELVSGKRKE KSMTQQELAD ATGINRSLLS RLEKKDFIPS

RumObeHYP1 .......... .......... .......... .......... ..........

FaePraHYPO .......... .......... .......... .......... ..........

EubDolHYPO .......... .......... .......... .......... ..........

BacPleHYPO .......... .......... .......... .......... ..........

BacCopHYPO .......... .......... .......... .......... ..........

StrTheESP .......... .......... .......... .......... ..........

StrIniCPS .......... .......... .......... .......... ..........

SerProUGD .......... .......... .......... .......... ..........

GeoMetUGD .......... .......... .......... .......... ..........

GeoUraUGD .......... .......... .......... .......... ..........

AnoFlaUGD .......... .......... .......... .......... ..........

PorGinNSD ....MGQACI GSRPISFLWE VRKCRRFIGS YTIIRRVIVS HVLSGSRHYI

BacSelRPON .......... .......... .......... .......... ..........

PelPhaNSD .......... .......... .......... .......... ..........

MetPopNSD .......... .......... .......... .......... ..........

MetExtNSD .......... .......... .......... .......... ..........

VibVulPRE .......... .......... .......... .......... ..........

GeoKauNSD .......... .......... .......... .......... ..........

RhiTriEXO .......... .......... .......... .......... ..........

ParDisUGD2 .......... .......... .......... .......... ..........

XanAxoUGD .......... .......... .......... .......... ..........

XanCamUGD .......... .......... .......... .......... ..........

EntFaeUGD .......... .......... .......... .......... ..........

StrPneUGD2 .......... .......... .......... .......... ..........

StrPneUGD3 .......... .......... .......... .......... ..........

StrPneUG .......... .......... .......... .......... ..........

EscAlbUGD .......... .......... .......... .......... ..........

BacTheUGD .......... .......... .......... .......... ..........

SacSpiUGD .......... .......... .......... .......... ..........

Aer159PBP .......... .......... .......... .......... ..........

ButSynUGD .......... .......... .......... .......... ..........

BraJapUGD .......... .......... .......... .......... ..........

SynCC9UGD .......... .......... .......... .......... ..........

StePneUGD .......... .......... .......... .......... ..........

SalAreUGD .......... .......... .......... .......... ..........

BurYI2NSD .......... .......... .......... .......... ..........

BacCerNSD .......... .......... .......... .......... ..........

PaeSanHYPO .......... .......... .......... .......... ..........

Rhi42MUGD .......... .......... .......... .......... ..........

OpiTAV5NSD .......... .......... .......... .......... ..........

MarHTCUGD8 .......... .......... .......... .......... ..........

BacSubUGD .......... .......... .......... .......... ..........

NocKunUGD .......... .......... .......... .......... ..........

DesHafUGD .......... .......... .......... .......... ..........

BacThuUGD .......... .......... .......... .......... ..........

SphJapUGD .......... .......... .......... .......... ..........

RicSibUGD .......... .......... .......... .......... ..........

MetCapUGD .......... .......... .......... .......... ..........

HymAerUGD .......... .......... .......... .......... ..........

BacSubUGD7 .......... .......... .......... .......... ..........

ActGloUDG9 .......... .......... .......... .......... ..........

NatGarNSD5 .......... .......... .......... .......... ..........

UncBacHYPO .......... .......... .......... .......... ..........

HalZhaHYPP .......... .......... .......... .......... ..........

StrPneUGD1 .......... .......... .......... .......... ..........

HoePhoUGD .......... .......... .......... .......... ..........

CapSpuUGD .......... .......... .......... .......... ..........

NovAroUGD .......... .......... .......... .......... ..........

ProMarUGD .......... .......... .......... .......... ..........

HomSapUGD .......... .......... .......... .......... ..........

MusMusUNA .......... .......... .......... .......... ..........

DanRerUGD .......... .......... .......... .......... ..........

XenLaeUGD .......... .......... .......... .......... ..........

RatNorUGD .......... .......... .......... .......... ..........

PonAbeUGD .......... .......... .......... .......... ..........

BosTauUGD3 .......... .......... .......... .......... ..........

BamOldUGD .......... .......... .......... .......... ..........

PedCorUGD .......... .......... .......... .......... ..........

ZeaMayUGD8 .......... .......... .......... .......... ..........

StrPurUGDP .......... .......... .......... .......... ..........

CioIntUGDP .......... .......... .......... .......... ..........

NemVecPPRE .......... .......... .......... .......... ..........

CaeEleUGD .......... .......... .......... .......... ..........

PopTomUGD .......... .......... .......... .......... ..........

DroMelUGD .......... .......... .......... .......... ..........

AraThaUGD .......... .......... .......... .......... ..........

DroMelUGD1 .......... .......... .......... .......... ..........

NasVitUDG .......... .......... .......... .......... ..........

CaeBriHYPO .......... .......... .......... .......... ..........

DroWilGK3 .......... .......... .......... .......... ..........

NasVitUGDP .......... .......... .......... .......... ..........

DroVirGJ3 .......... .......... .......... .......... ..........

HomSapUGD2 .......... .......... .......... .......... ..........

XenTroUGD .......... .......... .......... .......... ..........

FlaCF1UGD .......... .......... .......... .......... ..........

ZeaMayUGD .......... .......... .......... .......... ..........

MayZebUGD1 .......... .......... .......... .......... ..........

VitvinUGD1 .......... .......... .......... .......... ..........

HomSapUGD1 .......... .......... .......... .......... ..........

OviAriUGD1 .......... .......... .......... .......... ..........

FelCatUGD1 .......... .......... .......... .......... ..........

EquCabUGD1 .......... .......... .......... .......... ..........

CanFamUGD1 .......... .......... .......... .......... ..........

DroMojGI7 .......... .......... .......... .......... ..........

DroPseGA34 .......... .......... .......... .......... ..........

HomSapICRA .......... .......... .......... .......... ..........

ColLivUGD .......... .......... .......... .......... ..........

CamFloUGD .......... .......... .......... .......... ..........

BosTauUGD .......... .......... .......... .......... ..........

MelGal1UGD .......... .......... .......... .......... ..........

AilMelUGDP .......... .......... .......... .......... ..........

MusFurUGDP .......... .......... .......... .......... ..........

MusFur1UGD .......... .......... .......... .......... ..........

MelUnd1UGD .......... .......... .......... .......... ..........

FalPer1UGD .......... .......... .......... .......... ..........

ChrBel2UGD .......... .......... .......... .......... ..........

FelCat1UGD .......... .......... .......... .......... ..........

SaiBolUGDP .......... .......... .......... .......... ..........

DroWilGK1 .......... .......... .......... .......... ..........

CaeRemQV40 .......... .......... .......... .......... ..........

CaeBriQV40 .......... .......... .......... .......... ..........

ZeaMayUGDI .......... .......... .......... .......... ..........

GalGalUG .......... .......... .......... .......... ..........

CaeEleUG .......... .......... .......... .......... ..........

PseAerGMD .......... .......... .......... .......... ..........

CanCloGM .......... .......... .......... .......... ..........

VibCraGMD .......... .......... .......... .......... ..........

PseSyrGMD .......... .......... .......... .......... ..........

AmyAzuGMD .......... .......... .......... .......... ..........

OxaAB1GMD8 .......... .......... .......... .......... ..........

ActGloGDM .......... .......... .......... .......... ..........

AmyAlbGDM7 .......... .......... .......... .......... ..........

AmyBalGDM .......... .......... .......... .......... ..........

PseDC3GMD5 .......... .......... .......... .......... ..........

PseVerGMD1 .......... .......... .......... .......... ..........

PseQDAGMD1 .......... .......... .......... .......... ..........

PseAerGMD1 .......... .......... .......... .......... ..........

PseHYSGMD1 .......... .......... .......... .......... ..........

AmyAlbGMD6 .......... .......... .......... .......... ..........

AmyAzuGMD1 .......... .......... .......... .......... ..........

AlgSagHYPO .......... .......... .......... .......... ..........

AciKBSHYPO .......... .......... .......... .......... ..........

AmyBalGMD .......... .......... .......... .......... ..........

StrCanGMD1 .......... .......... .......... .......... ..........

PseAlcGMD1 .......... .......... .......... .......... ..........

ActGloGMD6 .......... .......... .......... .......... ..........

OxaAB1GMD1 .......... .......... .......... .......... ..........

PseAerUNPP .......... .......... .......... .......... ..........

HahGanHYPO .......... .......... .......... .......... ..........

DesSalNSD .......... .......... .......... .......... ..........

PseTomGMD1 .......... .......... .......... .......... ..........

MarAlgGMD .......... .......... .......... .......... ..........

PseSyrGMD3 .......... .......... .......... .......... ..........

NovAroGMD .......... .......... .......... .......... ..........

AzoSpGMD .......... .......... .......... .......... ..........

AzoVinGMD .......... .......... .......... .......... ..........

BacThuGMD .......... .......... .......... .......... ..........

HahGanHYPP .......... .......... .......... .......... ..........

AlgSagHYPP .......... .......... .......... .......... ..........

AciKBSHYPP .......... .......... .......... .......... ..........

PseSyrGMD2 .......... .......... .......... .......... ..........

PseSyrGMD1 .......... .......... .......... .......... ..........

StrCanGMD .......... .......... .......... .......... ..........

PseVerGMD .......... .......... .......... .......... ..........

PseQDAGMD .......... .......... .......... .......... ..........

PseHYSGMD .......... .......... .......... .......... ..........

PseAlcGMD .......... .......... .......... .......... ..........

PseAerUPP .......... .......... .......... .......... ..........

StaAurUMD .......... .......... .......... .......... ..........

EscColUMND .......... .......... .......... .......... ..........

LusFleUGD .......... .......... .......... .......... ..........

SalEntUMD .......... .......... .......... .......... ..........

ActSerUG .......... .......... .......... .......... ..........

ErwTasUG .......... .......... .......... .......... ..........

FusUlcUMD .......... .......... .......... .......... ..........

CorMarUMD .......... .......... .......... .......... ..........

StaVitUMD .......... .......... .......... .......... ..........

SheVioUMD .......... .......... .......... .......... ..........

SerS4UMD .......... .......... .......... .......... ..........

PseStuUMD .......... .......... .......... .......... ..........

PsePseUMD .......... .......... .......... .......... ..........

PseFluUMD .......... .......... .......... .......... ..........

HalSalUMD .......... .......... .......... .......... ..........

FraPhiUMD .......... .......... .......... .......... ..........

EdwC07UMD .......... .......... .......... .......... ..........

AerAquUMD .......... .......... .......... .......... ..........

GeoWSUUMD .......... .......... .......... .......... ..........

EscMG1UMD .......... .......... .......... .......... ..........

DicZeaUMD .......... .......... .......... .......... ..........

VibOrdUMD .......... .......... .......... .......... ..........

VidVulUMaD .......... .......... .......... .......... ..........

KlePneUMaD .......... .......... .......... .......... ..........

GeoTheUMaD .......... .......... .......... .......... ..........

BurXenUMaD .......... .......... .......... .......... ..........

HasParUMaD .......... .......... .......... .......... ..........

RalSolUMaD .......... .......... .......... .......... ..........

PolIrgUMaD .......... .......... .......... .......... ..........

RalSolNGD .......... .......... .......... .......... ..........

YerEntUMaD .......... .......... .......... .......... ..........

EscFerUMaD .......... .......... .......... .......... ..........

OceGraUMaD .......... .......... .......... .......... ..........

BacNRRUMaD .......... .......... .......... .......... ..........

GeoKauUMaD .......... .......... .......... .......... ..........

PsyCNPUMaD .......... .......... .......... .......... ..........

MorPE3UMaD .......... .......... .......... .......... ..........

SerProUMaD .......... .......... .......... .......... ..........

51 100

StrPyoUGD .......... .......... .......... ..MKIAVAGS GYVGLSLGVL

HymAerUGD1 .......... .......... .......... ..MKIAVVGT GYVGLVTGTC

MetCapUGD1 .......... .......... .......... ..MKVTIFGS GYVGLVTGAC

ActGloUGD6 .......... .......... .......... ..MRVCVVGT GYVGLTTGVS

HalZhaHYPO .......... .......... .......... ..MNITIFGT GYVGLVTGTC

MCIThaHYPO .......... .......... .......... ..MKIAVIGL GFVGLTLSSV

SalPacUGD .......... .......... .......... ...KVTVIGS GHLGATHAAG

NatGarNSD2 .......... .......... .......... ..ATICVVGL GYVGLPLAVG

BacSubUGD2 .......... .......... .......... ...KICVVGA GYVGLTLSAA

AzoTolUGD .......... .......... .......... ...KIAIAGT GYVGLSNAVL

LacCreEPS .......... .......... .......... MKRKISVAGI GYVGLSLAVL

AlaProUG .......... .......... ........MT NKTKITVVGS GYVGMSLSVL

StrZooUG .......... .......... .......... ..MKISVAGS GYVGLSLSIL

StrHGBNSD .......... .......... .......... ...RVAVLGQ GYVGLPLTMR

NatGarNSD .......... .......... .......... ..MNVSIIGS GYVGTTVAAC

TheMelUGD .......... .......... .......... .MHKISVAGA GYVGLSIATM

SulDenUGD .......... .......... .......... ..MKIAIAGT GYVGLSNGIL

SphAlaUGD .......... .......... ....MTSAKD SPVKIAVVGT GYVGISNAVL

SheOneUGD .......... .......... .......... ..MNIVVVGM GYVGLSNAVL

SalSerUGD .......... .......... .......... ..MKITISGT GYVGLSNGLL

PseHalUGD .......... .......... .......... ..MKVTVVGT GYVGLSNAML

ProMirUGD .......... .......... .......... ..MKITISGT GYVGLSNGIL

ParDisUGD .......... .......... ........MN TDIKIAVAGT GYVGMSIATL

ParDisUGD1 .......... .......... .........M KKYKIAVAGT GYVGLSIATL

LacSalUGD .......... .......... ........ME NRLNIAVAGV GYVGMSLAVL

CloPerUGD PQLEKLGEVL EFEPISLFLN DEDKASPLKC SPLNIAVAGT GYVGLSIATL

ArcButUGD .......... .......... .......... ..MKIAIAGT GYVGLSNGLL

AerHydUGD .......... .......... .......... ..MKIAICGT GYVGLSNAVL

LacJohSBP .......... .......... .........M NSYKIAVAGT GYVGLSLATL

StrUbeUGD .......... .......... .......... ..MKIAVAGS GYVGLSLSVL

ReiMEDNSD .......... .......... .......... ..MKIAVAGT GYVGLSNAML

ExiSibNSD .......... .......... .......... ..MKITVAGT GYVGLSMAVL

RumObeHYPO IPQLESLGEC LGFDPDEAFT EQSASRLPSP SPLNIAVAGT GYVGLSIATL

RumObeHYP1 .......... .......... .......... ..MKIAVAGT GYVGLSIATL

FaePraHYPO .......... .......... .......... ..MKIAVAGT GYVGLSIATL

EubDolHYPO .......... .......... .......... ..MKIAVAGT GYVGLSIAIL

BacPleHYPO .......... .......... .........M KQLNIAVAGT GYVGLSIATL

BacCopHYPO .......... .......... ........MM KNYTIAVAGT GYVGLSIATL

StrTheESP .......... .......... ......MREF KDLKIAVAGT GYVGLSIATL

StrIniCPS .......... .......... .......... .MKKIAVAGT GYVGLSMAVL

SerProUGD .......... .......... .......... ..MKVTVFGI GYVGLVQAAV

GeoMetUGD .......... .......... .......... ..MKICVIGS GYVGLVAGTC

GeoUraUGD .......... .......... .......... ..MKICIIGT GYVGLVAGTC

AnoFlaUGD .......... .......... .......... ..MNIVVVGT GYVGLVTGVA

PorGinNSD PATGRGNSRL SLDKASPFYL TFVPKNSISY YNMDIAVVGI GYVGLVSATC

BacSelRPON .......... .......MRK TKVKCIERGF YMYKIAVAGT GYVGLVAGVC

PelPhaNSD .......... .......... .......... ..MKITIFGS GYVGLVTGAC

MetPopNSD .......... .......... .......... ..MRIAMIGS GYVGLVSGAC

MetExtNSD .......... .......... .......... ..MRIAMIGS GYVGLVSGAC

VibVulPRE .......... .......... .......... ..MRITVVGA GYVGLANAAL

GeoKauNSD .......... .......... .......... ..MKITIAGA GYVGLVTAAC

RhiTriEXO .......... .......... .......... ..MRITMIGS GYVGLVSGVC

ParDisUGD2 .......... .......... ......MKSF QELKIAVAGT GYVGLSIATL

XanAxoUGD .......... .......... .......... ..MKIAVAGT GYVGLSNGVL

XanCamUGD .......... .......... .......... ..MKIAVAGT GYVGLSNGVL

EntFaeUGD .......... .......... ......MREY KGLKIAVAGT GYVGLSIATL

StrPneUGD2 .......... .......... .......... ..MKIAVAGT GYVGLSIAVL

StrPneUGD3 .......... .......... .......... ..MKIAVTGT GYVGLSIAVL

StrPneUG .......... .......... .......... ..MKIAVAGT GYVGLSIAVL

EscAlbUGD .......... .......... .......... ..MKITISGT GYVGLSNGLL

BacTheUGD .......... .......... .......MDT KELKIAVAGT GYVGLSIATL

SacSpiUGD .......... .......... .........M PGARVVIVGQ GYVGLPVAMR

Aer159PBP .......... .......... .......... ...KIGIIGL GYVGLPLAVE

ButSynUGD .......... .......... .......... ...KISVAGT GYVGLSIATL

BraJapUGD .......... .......... .......... ...RIAMIGT GYVGLVSGAC

SynCC9UGD .......... .......... .........M TIQRICCIGA GYVGGPTMAV

StePneUGD .......... .......... .......... ...KIAIAGS GYVGLSLAVL

SalAreUGD .......... .......... .......... ........GA GYVGTCLGVA

BurYI2NSD .......... .......... .......... ...KIGVVGL GYVGLPLAVE

BacCerNSD .......... .......... .......... .MYKIAVAGT GYVGLVAGVC

PaeSanHYPO .......... .......... .......... ..RTIAIVGL GYVGLPLAET

Rhi42MUGD .......... .......... .......... ...RIVMIGS GYVGLVSGAC

OpiTAV5NSD .......... .......... .......... ..MTICCIGA GYVGGPTMAM

MarHTCUGD8 .......... .......... ........MH KITKICCIGA GYVGGPTMSV

BacSubUGD .......... .......... .......... ..MNITVIGT GYVGLVTGVS

NocKunUGD .......... .......... .......... ......VLGL GYVGLPLAAE

DesHafUGD .......... .......... .......... ..ATVGVIGL GYVGLPLAVE

BacThuUGD .......... .......... .......... ....ICVVGL GYIGLPTASL

SphJapUGD .......... .......... .......... ....VSVIGL GYIGLPTAAL

RicSibUGD .......... .......... .......... ...NITFIGS GYVGLVSGII

MetCapUGD .......... .......... .......... ..MKVTIFGS GYVGLVTGAC

HymAerUGD .......... .......... .......... ..MKIAVVGT GYVGLVTGTC

BacSubUGD7 .......... .......... .......... ..MNITVIGT GYVGLVTGVS

ActGloUDG9 .......... .......... .......... ..MRVCVVGT GYVGLTTGVS

NatGarNSD5 .......... .......... .......... ..MNVSIIGS GYVGTTVAAC

UncBacHYPO .......... .......... .......... ........GL GKLGACTAAC

HalZhaHYPP .......... .......... .......... ..MNITIFGT GYVGLVTGTC

StrPneUGD1 .......... .......... .......... ..MKIAVTGT GYVGLSIAVL

HoePhoUGD .......... .......... .......... ..MRISIVGA GYVGLVSGVC

CapSpuUGD .......... .......... .......... ..MKIAVIGT GYVGLVSGTC

NovAroUGD .......... .......... .......... ..MKIAMVGS GYVGLVSGAC

ProMarUGD .......... .......... ........MY LIKKICCIGA GYVGGPTMAV

HomSapUGD .......... .......... ........MF EIKKICCIGA GYVGGPTCSV

MusMusUNA .......... .......... ........MV EIKKICCIGA GYVGGPTCSV

DanRerUGD .......... .......... ........MF QIKKICCIGA GYVGGPTCSV

XenLaeUGD .......... .......... ........MF QIKKICCIGA GYVGGPTCSV

RatNorUGD .......... .......... ........MV EIKKICCIGA GYVGGPTCSV

PonAbeUGD .......... .......... ........MF EIKKICCIGA GYVGGPTCSV

BosTauUGD3 .......... .......... ........MF EIKKICCIGA GYVGGPTCSV

BamOldUGD .......... .......... .......... .MVKICCIGA GYVGGPTMAV

PedCorUGD .......... .......... .........M SVKKICCIGA GYVGGPTCSI

ZeaMayUGD8 .......... .......... .......... .MVKICCIGA GYVGGPTMAV

StrPurUGDP .......... .......... .........M VIKKICCMGA GYVGGPTCSV

CioIntUGDP .......... .......... .........M KVKKICCIGA GYVGGPTCSV

NemVecPPRE .......... .......... ........MF NIKNICCIGA GYVGGPSCSV

CaeEleUGD .......... .......... ...MTDQVFG KVSKVVCVGA GYVGGPTCAM

PopTomUGD .......... .......... .......... .MVKICCIGA GYVGGPTMAV

DroMelUGD .......... .......... .......... ..MKVCCIGA GYVGGPTCAV

AraThaUGD .......... .......... .......... .MVKICCIGA GYVGGPTMAV

DroMelUGD1 .......... .......... .......... ..MKVCCIGA GYVGGPTCAV

NasVitUDG .......... .......... .........M PITKICCIGA GYVGGPTCSV

CaeBriHYPO .......... .......... ...MTDQVFG KVSKVVCVGA GYVGGPTCAM

DroWilGK3 .......... .......... .......... ..MKVCCIGA GYVGGPTCAV

NasVitUGDP .......... .......... .......... ..MKVCCIGA GYVGGPTCAV

DroVirGJ3 .......... .......... .......... ..MKVCCIGA GYVGGPTCAV

HomSapUGD2 .......... .......... ........MF EIKKICCIGA GYVGGPTCSV

XenTroUGD .......... .......... ........MV QVKNICCIGA GYVGGPTCTV

FlaCF1UGD .......... .......... .........M KITKICCIGA GYVGGPTMAV

ZeaMayUGD .......... .......... .......... .MVKICCIGA GYVGGPTMAV

MayZebUGD1 .......... .......... ........MF QIKRICCIGA GYVGGPTCSV

VitvinUGD1 .......... .......... .......... .MVKICCIGA GYVGGPTMAV

HomSapUGD1 .......... .....MWSSG LLIQDSTIMF EIKKICCIGA GYVGGPTCSV

OviAriUGD1 .......... .......... ........MF EIKKICCIGA GYVGGPTCSV

FelCatUGD1 .......... .......... ........MF EIKKICCIGA GYVGGPTCSV

EquCabUGD1 .......... .......... ........MF EIKKICCIGA GYVGGPTCSV

CanFamUGD1 .......... .......... ........MF EIKKICCIGA GYVGGPTCSV

DroMojGI7 .......... .......... .......... ..MKVCCIGA GYVGGPTCAV

DroPseGA34 .......... .......... .......... ..MKVCCIGA GYVGGPTCAV

HomSapICRA .......... .......... ........MF EIKKICCIGA GYVGGPTCSV

ColLivUGD .......... .......... ........MF EIKKICCIGA GYVGGPTCSV

CamFloUGD .......... .......... ........MT RIRKICCIGA GYVGGPTCSV

BosTauUGD .......... .......... ........MF EIKKICCIGA GYVGGPTCSV

MelGal1UGD .......... .......... ........MF EIKKICCIGA GYVGGPTCSV

AilMelUGDP .......... .......... ........MF EIKKICCIGA GYVGGPTCSV

MusFurUGDP .......... .......... ........MF EIKKICCIGA GYVGGPTCSV

MusFur1UGD ...MFNVISL NFLFSIVEQS YCFPSSLIMF EIKKICCIGA GYVGGPTCSV

MelUnd1UGD .......... .......... ........MF DIKKICCIGA GYVGGPTCSV

FalPer1UGD .......... .......... ........MF EIKKICCIGA GYVGGPTCSV

ChrBel2UGD .......... .......... ........MF EIKKICCIGA GYVGGPTCSV

FelCat1UGD .......... .......... ........MF EIKKICCIGA GYVGGPTCSV

SaiBolUGDP .........M RNSASSRQEA PLSQKRTIMF EIKKICCIGA GYVGGPTCSV

DroWilGK1 .......... .......... .......... ..MKVCCIGA GYVGGPTCAV

CaeRemQV40 .......... .......... ...MTDQVFG KVSKVVCVGA GYVGGPTCAM

CaeBriQV40 .......... .......... ...MTDQVFG KVSKVVCVGA GYVGGPTCAM

ZeaMayUGDI .......... .......... .......... .MVKICCIGA GYVGGPTMAV

GalGalUG .......... .......... ........MF EIKKICCIGA GYVGGPTCSV

CaeEleUG .......... .......... ...MTDQVFG KVSKVVCVGA GYVGGPTCAM

PseAerGMD .......... .......... .......... ..MRISIFGL GYVGAVCAGC

CanCloGM .......... .......... .......... .MVKISIFGL GYVGCVSLGC

VibCraGMD .......... .......... .......... .......VGL GYIGLPTAAM

PseSyrGMD .......... .......... .......... ..MRISIFGL GYVGAVCAGC

AmyAzuGMD .......... .......... .......... ..MKISVFGL GYVGCVSAAC

OxaAB1GMD8 .......... .......... .......... ..MKISIFGL GYVGAVSAGC

ActGloGDM .......... .......... .......... ..MRISVFGL GYVGSVSAAC

AmyAlbGDM7 .......... .......... .......... ..MKISVFGL GYVGCVSAAC

AmyBalGDM .......... .......... .......... ..MKISVFGL GYVGCVSAAC

PseDC3GMD5 .......... .......... .......... ..MRISIFGL GYVGAVCAGC

PseVerGMD1 .......... .......... .......... ..MRISIFGL GYVGAVCAGC

PseQDAGMD1 .......... .......... .......... ..MRISIFGL GYVGAVCAGC

PseAerGMD1 .......... .......... .......... ..MRISIFGL GYVGAVCAGC

PseHYSGMD1 .......... .......... .......... ..MNISIFGL GYVGAVCAGC

AmyAlbGMD6 .......... .......... .......... ..MKISVFGL GYVGCVSAAC

AmyAzuGMD1 .......... .......... .......... ..MKISVFGL GYVGCVSAAC

AlgSagHYPO .......... .......... .........M TTPIISIFGM GYVGVVSAVS

AciKBSHYPO .......... .......... .......... MSSRIAVLGL GYVGCVSAAC

AmyBalGMD .......... .......... .......... ..MKISVFGL GYVGCVSAAC

StrCanGMD1 .......... .......... .......... ..MRISVFGL GYVGCVSAAC

PseAlcGMD1 .......... .......... .......... ..MRISIFGL GYVGAVCAGC

ActGloGMD6 .......... .......... .......... ..MRISVFGL GYVGSVSAAC

OxaAB1GMD1 .......... .......... .......... ..MKISIFGL GYVGAVSAGC

PseAerUNPP .......... .......... .......... ..MRISIFGL GYVGAVCAGC

HahGanHYPO .......... .......... .......... ..MRISIFGL GYVGAVCAAS

DesSalNSD .......... .......... .......... ..MKVSVFGL GYVGTVSAGC

PseTomGMD1 .......... .......... .......... ..MRISIFGL GYVGAVCAGC

MarAlgGMD .......... .......... .......... ..MRVSIFGL GYVGAVCTAS

PseSyrGMD3 .......... .......... .......... ..MRISIFGL GYVGAVCAGC

NovAroGMD .......... .......... .......... ..MKIAILGL GYVGCTAAGC

AzoSpGMD .......... .......... .......... ..MKISIFGL GYVGAVSLAC

AzoVinGMD .......... .......... .......... ..MRISIFGL GYVGAVCAGC

BacThuGMD .......... .......... .......... ....ICVVGL GYIGLPTCAM

HahGanHYPP .......... .......... .......... ..MRISIFGL GYVGAVCAAS

AlgSagHYPP .......... .......... .........M TTPIISIFGM GYVGVVSAVS

AciKBSHYPP .......... .......... .......... MSSRIAVLGL GYVGCVSAAC

PseSyrGMD2 .......... .......... .......... ..MRISIFGL GYVGAVCAGC

PseSyrGMD1 .......... .......... .......... ..MRISIFGL GYVGAVCAGC

StrCanGMD .......... .......... .......... ..MRISVFGL GYVGCVSAAC

PseVerGMD .......... .......... .......... ..MRISIFGL GYVGAVCAGC

PseQDAGMD .......... .......... .......... ..MRISIFGL GYVGAVCAGC

PseHYSGMD .......... .......... .......... ..MNISIFGL GYVGAVCAGC

PseAlcGMD .......... .......... .......... ..MRISIFGL GYVGAVCAGC

PseAerUPP .......... .......... .......... ..MRISIFGL GYVGAVCAGC

StaAurUMD .......... .......... .......... ..SKLTVVGL GYIGLPTSIM

EscColUMND .......... .......... .........M SFATISVIGL GYIGLPTRAF

LusFleUGD .......... .......... .......... ....INVIGL GYIGLPTSVV

SalEntUMD .......... .......... .........M SFTTISVIGL GYIGLPTAAA

ActSerUG .......... .......... ........MN TFNRIAIIGL GYIGLPAAVA

ErwTasUG .......... .......... .........M IFKTISVIGL GYIGLPTAAA

FusUlcUMD .......... .......... .......... ....ITILGM GYIGLPTAIA

CorMarUMD .......... .......... .........M SDYQIAFVGL GYIGLPTAVV

StaVitUMD .......... .......... .......... ....LTTIGL GYIGLPTSIM

SheVioUMD .......... .......... .........M KFNTISIVGL GYIGLPTAAV

SerS4UMD .......... .......... .........M SFNTISVIGL GYIGLPTAAA

PseStuUMD .......... .......... .........M PFQTISVIGL GYIGLPTAAV

PsePseUMD .......... .......... .........M QFKTISVVGL GYIGLPTAAV

PseFluUMD .......... .......... .........M SLQTISVIGL GYIGLPTAAV

HalSalUMD .......... .......... .......... ...TVAVVGL GYIGLPTALL

FraPhiUMD .......... .......... .........M KFNSVCMVGL GYIGLPTAAV

EdwC07UMD .......... .......... .........M SFDTISVIGL GYIGLPTAVA

AerAquUMD .......... .......... .........M KFETISVVGL GYIGLPTAAV

GeoWSUUMD .......... .......... .......... ..AVIGVVGL GYVGLPLAVE

EscMG1UMD .......... .......... .........M SFATISVIGL GYIGLPTRAF

DicZeaUMD .......... .......... .........M SFNRICVLGL GYIGLPTAAV

VibOrdUMD .......... .......... .......... ....VSVVGL GYIGLPTAAM

VidVulUMaD .......... .......... .........M SFETISVIGL GYIGLPTAAM

KlePneUMaD .......... .......... .........M SFSTISVIGL GYIGLPTAAA

GeoTheUMaD .......... .......... .......... .MKKVCVVGL GYIGLPTSAI

BurXenUMaD .......... .......... .........M PFDVVSIIGL GYIGLPTAAA

HasParUMaD .......... .......... ........MA VFEKIVVVGL GYIGLPTAAA

RalSolUMaD .......... .......... ...MDRAIDI DFRTISVVGL GYIGLPTATV

PolIrgUMaD .......... .......... .......... ..MKAVFMGL GYIGLPTAAV

RalSolNGD .......... .......... ...MDRAIEI DFRTISVVGL GYIGLPTATV

YerEntUMaD .......... .......... .........M SFETISVIGL GYIGLPTAAA

EscFerUMaD .......... .......... .........M SFATISVIGL GYIGLPTAAA

OceGraUMaD .......... ........MT IQESAKTSAG PFSRVCVIGL GYIGLPTAAM

BacNRRUMaD .......... ....MKSTIL QNDLKEKLLN KTATLGVIGL GYVGLPLAVE

GeoKauUMaD .......... .......MNY AERLLQKFEK RDAVIGVVGL GYVGLPLAVE

PsyCNPUMaD .......... .......... .........M NINTISVVGL GYIGLPTAAV

MorPE3UMaD .......... .......... .........M SFETISVVGL GYIGLPTAAM

SerProUMaD .......... .......... .........M SFNTISVIGL GYIGLPTAAA

101 150

StrPyoUGD LSLQ...NEV TIVDILPSKV DKINNG...L SPIQDEYIEY YLKSK..QLS

HymAerUGD1 FAEVG..IDV TCIDIDQRKI DNLRQG...I LPIYEPGLEE MVTRNVEAGR

MetCapUGD1 LAEVG..NDV VCIDIDAGKI ERLERG...E VPIYEPGLDT IIKRNMVAGR

ActGloUGD6 LAFLG..HQV TCVDLDQSKV DMLNGG...R CPIYEPGMDD LLAEA..ATN

HalZhaHYPO LADAG..HHV MCVDVDKSKI DRLNEG...I IPIFEPGLEQ MVRQNVESAR

MCIThaHYPO LASKG..VIT VGIDLDKEKR SKIAKG...T PTFFEPNLKK TLKKALK.KN

SalPacUGD MADIG..HEV IGVDIDPEKV ETLNAG...R GWFHEPELDV LLARNLDTGR

NatGarNSD2 FARSN..YRV IGYDVDDVTV SRLQEG...I DTTGDLS.DD AIQ....DGD

BacSubUGD2 LASIG..HDM ICTDKDVKKI GQLKKG...V IPFYEPGLSD AILR...CGN

AzoTolUGD LAQS...NEV VALDIASERV EMLNRR...Q SPIVDSEIEE YLQRR..SLD

LacCreEPS LAQK...NHV TAVDIIQSKV DLINNK...K SPIQDDYIEK YLAEK..ELD

AlaProUG LAQH...NDV VVLDVDASRV EKIINR...Q STVADAEIEA FLADK..ELS

StrZooUG LAQH...NDV TVVDIIDEKV RLINQG...I SPIKDADIEE YLKNA..PLN

StrHGBNSD AVEAG..FDV VGFDVDKARV DLLARG...E SFIEDIDPAA VAEAVA.TGR

NatGarNSD LAELG..HDV VNVEIDEEIV EMINAG...E APIHESGLAE RIAAHA.GTT

TheMelUGD LSQN...HNV TILDVVDKKV KMINER...I CPIKDKEIEE FFKTK..KLN

SulDenUGD LSQH...NEV VALDIIPQKV EMLNKK...I SPIEDKEIEE YLVRD..DLN

SphAlaUGD LAQH...NAV VALDIDTRKV ELVNAR...Q SPIVDPEIED YLANK..QLN

SheOneUGD LAQQ...NQV TAIDLMPERV KLVNDR...K STVADNLIEE YLRNS..ALN

SalSerUGD IAQH...HDV VALDIVPSRV ELLNDR...I SPIVDKEIQQ FLKED..NIR

PseHalUGD LAQH...NEV IALDIDEQKI ALLNNK...Q SPIVDFHISE YLQRD..DIS

ProMirUGD LSQH...NKV VALDINKEKV ELLNKK...I SPISDKEIEE FLATK..ELD

ParDisUGD LAQH...HQV IAVDVIPEKV EKINHQ...V SPIQDEYIEK YLAEK..PLN

ParDisUGD1 LAQH...NQV FAVDVIQSKV DAINQR...K SPIQDEYIEK YLTEK..PLD

LacSalUGD LAQH...NHV SAVDTVPEKV ELINKR...Q SPIQDEYIEK YLAEK..ELD

CloPerUGD LAQN...NHV TAVDIIPEKV EMINNK...K SPIQDDYIEK YLAEK..DLD

ArcButUGD LSQY...NEV IALDIVPQKV EMLNNK...M SPIEDKEIEE YLKKD..HIN

AerHydUGD LAQH...HEV VAVDVIASKV EMINEW...K SPIVDKEIER YLASR..DLN

LacJohSBP LSQH...NEV TAVDIVPEKV DLINNH...K SPIVDKEIED FLANK..DLN

StrUbeUGD LAQK...NPV TVVDIIEKKV NLINQK...Q SPIQDVDIEN YLKEK..KLQ

ReiMEDNSD LAQN...HEV VAVDIVPEKV EQLNQG...Q SPIEDTEIED FLANK..TLN

ExiSibNSD LAQH...NEV TSIDIIKERV ELVNDR...K SPIVDAEIEN FLQNK..ELN

RumObeHYPO LAQH...NHV TAVDIIPEKV DLINKR...K SPIQDEYIEK YLAEK..ELD

RumObeHYP1 LSQN...HEV MAVDIVPEKV EKINKR...I SPIQDEYIEK YLKEK..ELN

FaePraHYPO LSQH...HEV MAVDIIPEKV DLINHK...K SPIQDEYIEK YLAEK..DLN

EubDolHYPO LAQH...HEV VTVDIIPEKV ELINNK...K SPIQDDYIEK YLAEK..ELN

BacPleHYPO LAQH...HRV TAVDVIPEKV EKINKR...I SPIQDEYIEK YLTEK..ELN

BacCopHYPO LAQH...HRV VAVDVIPEKV EKINNR...I SPIQDEYIEK YLAEK..ELN

StrTheESP LSQH...HKV TAVDIIPEKV ELINNK...K SPIQDEYIEK YLAEK..ELD

StrIniCPS LAQH...HQV IAVDIVPEKV DLINAK...K SPIQDKEIEE FLASK..ELH

SerProUGD LAEVG..HDV MCIDIDERKV ENLKKG...N IPIFEPGLTP LVQQNYEAGR

GeoMetUGD FAESG..NTV ICVDVNQEKI EGLKQG...I LPIYEPGLKE LVLRNSAEGR

GeoUraUGD FAESG..NDV ICVDVDEAKI EGLKNG...V IPIYEPGLKE LVLRNCEEGR

AnoFlaUGD LAHVG..HRV TCVDVDEEKV ERMRQG...I SPIYESGIEP LMKENMEANR

PorGinNSD FAELG..ANV RCIDTDRNKI EQLNSG...T IPIYEPGLEK MIARNVKAGR

BacSelRPON FAEVG..HQV TCVDIDENKV NLMKSG...V SPIYEAGLEE LMQKNYAAGR

PelPhaNSD FAEVG..NDV LCVDIDQRKI DALNSG...K IPIHEPGLDE IVAENIKEGR

MetPopNSD LADFG..HEV VCIDKDPAKI AALNEG...R MPIYEPGLDA LVAENVRQKR

MetExtNSD LADFG..HEV VCIDNDPGKI AALNEG...R MPIYEPGLDA LVAENVRAKR

VibVulPRE LAQY...HSI VLLDTDSKRV EQINAK...S SPIVDVDIER FLSSG..ALQ

GeoKauNSD LADKG..HDV ICVDVNEEKI RRLNEG...I VPIYEPGLEP LIQRN..SAR

RhiTriEXO FADLG..HDV ICVDKDLSKI EALREG...R IPIYEPGLEQ LVAENTSTGR

ParDisUGD2 LSQH...HEV VAIDIVPEKV DLINNK...K SPIQDDYIEE YLASH..KLN

XanAxoUGD LARH...HEV VALDIVESKV KLLNEG...R SPIVDKELEA ALAQG..GLN

XanCamUGD LAKH...HEV VALDIVESKV KLLNEG...R SPIVDKELEA ALAQG..GLN

EntFaeUGD LAQS...HTV TAVDIIPEKV ELINNK...K SPIQDDYIEM YLAKK..ELD

StrPneUGD2 LAQH...HEI KAVDIVAEKV ELINQK...K SPIQDDYIEK YLVEK..DLN

StrPneUGD3 LAQH...HQV IAVDIIPEKV ELINQR...K SPIQDDYIEK YLAEK..DLN

StrPneUG LSQQ...HQV MAVDIIPEKV ELINQR...K SPIQDDYIEK YLAKK..DLN

EscAlbUGD IAQN...HEV VALDILPSRV AMLNDR...I SPVVDKEIQN FLQSD..EIR

BacTheUGD LSQH...HQV TAVDVIPEKV DMLNRK...Q SPIQDEYIEK YLSEK..ALN

SacSpiUGD ACEAG..FDV VGLDVAEDRI DLLQRG...K SFIDDVDDER LAAALA.TGR

Aer159PBP FGKK...FET LGFDIKTDRV NELRSG...Q DSTLECSAEE LLE....ATH

ButSynUGD LAQH...NEV TAVDIVPERV ELVNNR...K SPIQDDYIEE YLATK..PLN

BraJapUGD FADFG..HDV TCVDKDEKKI AALHRG...E IPIYEPGLDE LVATNVKAKR

SynCC9UGD IADHCPQIQV QVVDINQARI DAWNDADLSK LPVYEPGLDR VVERAR.GRN

StePneUGD LAQH...HEV KVIDVIKDKV ESINNR...K SPIKDEAIEK YLVEK..ELN

SalAreUGD LAERG..AEV VAVDSDPDTV EDLRAG...R CRLPEPGLAA MVRRLAATGR

BurYI2NSD FGKR...HPV VGFDINRARI GALREG...R DVTLEVDDEE LAS....ATL

BacCerNSD FAEVG..HQV TCVDIDEEKV KLMESG...V SPIYEADLEE LMQKNYTAGR

PaeSanHYPO YMDQG..FHV IGVDKDESKL KLLSEG...R SYIADVPESK I.RHYVQTRQ

Rhi42MUGD LADFG..HHV TCVDKSAAKI DALEAG...K VPIFEPGLDT IIDHNRTAGR

OpiTAV5NSD IALKAHDITV NVVDMNASRI AAWNS...DN LPVYEPGLDE IVKERR.GKN

MarHTCUGD8 IANQCPNIQV TVVDINQVRI DQWNDSNLDN LPIYEPGLKE IVRDTR.GKN

BacSubUGD LSEIG..HHV TCIDIDAHKI DEMRKG...I SPIFEPGLEE LMRKNTADGR

NocKunUGD AAAAG..LRV VGLDVSTSVV ESLNQG...I SHIDDLSPDD ISMMLD..QG

DesHafUGD KGKVG..FSV IGFDINAARV AKVNAG...D NYIADVKDEE LLELTQ.KGM

BacThuUGD LATKG..FQV YGVDVNESAV EMINSG...K VHIYEPDLDI MVKAAVQSGN

SphJapUGD IARGG..CQV VGVDVSAHVV ETVNSG...R VHIEEVDLDG LVQGVVARGN

RicSibUGD MGYLG..HNV TCLDNDEVKI SKLNKQ...I LPIYEVKLDE YLKQALESDR

MetCapUGD LAEVG..NDV VCIDIDAGKI ERLERG...E VPIYEPGLDT IIKRNMVAGR

HymAerUGD FAEVG..IDV TCIDIDQRKI DNLRQG...I LPIYEPGLEE MVTRNVEAGR

BacSubUGD7 LSEIG..HHV TCIDIDAHKI DEMRKG...I SPIFEPGLEE LMRKNTADGR

ActGloUDG9 LAFLG..HQV TCVDLDQSKV DMLNGG...R CPIYEPGMDD LLAEA..ATN

NatGarNSD5 LAELG..HDV VNVEIDEEIV EMINAG...E APIHESGLAE RIAAHA.GTT

UncBacHYPO FALRG..FDV IGIDVNPESV KAINAG...H APVWEPMLSE TIQKT..KSK

HalZhaHYPP LADAG..HHV MCVDVDKSKI DRLNEG...I IPIFEPGLEQ MVRQNVESAR

StrPneUGD1 LAQH...HQV IAVDIIPEKV ELINQR...K SPIQDDYIEK YLAEK..DLN

HoePhoUGD LSSFG..HDV TCIDNSSEKI DMLNSG...K VPIYEPGLEE LMAENVALGR

CapSpuUGD FAEMG..NKV TCVDVNTEKI NKLKQG...I IPIYEPGLEE MVLSNVAHQT

NovAroUGD FADFG..HDV VCIDKDEGKI ESLRQG...V MPIYEPGLAE LVAANVKAGR

ProMarUGD FAKNCPDIEI EVVDINQIRI NAWNSDDLTK LPVFEPGLPQ IIKKVR.GRN

HomSapUGD IAHMCPEIRV TVVDVNESRI NAWNSP...T LPIYEPGLKE VVESCR.GKN

MusMusUNA IAHMCPEIRV TVVDVNEARI NAWNSP...T LPIYEPGLKE VVESCR.GKN

DanRerUGD IASMCPEITV TVVDVNESRI KAWNSD...T LPIYEPGLNE VVLSCR.GKN

XenLaeUGD IAQMCPDIKV TVVDVNQARI NAWNSD...T LPIYEPGLKE VVESCR.GKN

RatNorUGD IARMCPEIRV TVVDVNEARI NAWNSP...T LPIYEPGLKE VVESCR.GKN

PonAbeUGD IAHMRPEIRV TVVDVNESRI NAWNSP...T LPIYEPGLKE VVESCR.GKN

BosTauUGD3 IAHMCPEIRV TVVDINESRI NAWNSP...T LPIYEPGLKE VVESCR.GKN

BamOldUGD IALKCPAIEV CVVDISVARI TAWNSD...Q LPIYEPGLDD VVKQCR.GRN

PedCorUGD IALKCPEITV TVIDKNEERI KQWNSD...K LPIYEPGLDE IVKNCR.NKN

ZeaMayUGD8 IALKCPDIEV VVVDISKPRI EAWNSD...T LPIYEPGLDD VVKQCR.GRN

StrPurUGDP IALKCPEVTV TVVDLSQARI DAWNSSD.FK LPIYEPSLEK VVRECR.GRN

CioIntUGDP MACKCPDIQV TVVDLNKARI NAWNSD...Q LPIYEPGLDE IVQKHR.GKN

NemVecPPRE IALKCPRIKV TVVDLSQHRI DAWNSD...N LPIFEPGLSE VVRECR.GRN

CaeEleUGD IAHKCPHITV TVVDMNTAKI AEWNSD...K LPIYEPGLDE IVFAAR.GRN

PopTomUGD IALKCPSIEV VVVDIWEPRI AAWNSD...Q LPIYEPGLYD VVKECR.GRN

DroMelUGD MALKCPDIVI TLVDKSSERI AQWNSD...K LPIYEPGLDE VVKRCR.NVN

AraThaUGD IALKCPDVEV AVVDISVPRI NAWNSD...T LPIYEPGLDD VVKQCR.GKN

DroMelUGD1 MALKCPDIVI TLVDKSSERI AQWNSD...K LPIYEPGLDE VVKRCR.NVN

NasVitUDG IALKCPHIRV TVVDKSYERI AQWNSS...K LPIYEPGLDD VVQQRR.NKN

CaeBriHYPO IAHKCPHVTV TVVDMNKDKI AEWNSD...K LPIYEPGLDE IVFAAR.GRN

DroWilGK3 MALKCPDIVI TLVDKSSERI AQWNSD...K LPIYEPGLDE VVKKCR.NVN

NasVitUGDP MALKCPDIVI TLVDKSSERI AQWNSD...K LPIYEPGLDE VVKRCR.NVN

DroVirGJ3 MALKCPDITI TLVDKSAERI AQWNSE...K LPIYEPGLDE VVKKCR.NVN

HomSapUGD2 IAHMCPEIRV TVVDVNESRI NAWNSP...T LPIYEPGLKE VVESCR.GKN

XenTroUGD IAQMCPEVTV TVVDINEERI RAWNSD...Q LPIYEPGLQD VVDFCR.GKN

FlaCF1UGD IAQKCPHIQV TVVDLNEQRI KDWNDPNTDN IPIYEPGLSE IVAEAR.GRN

ZeaMayUGD IALKCPDIEV VVVDISKPRI EAWNSD...T LPIYEPGLDD VVKQCR.GRN

MayZebUGD1 IAQMCPEITV TVVDVNESRI KAWNSD...T LPIYEPGLKE VVESCR.GRN

VitvinUGD1 IALKCPDIEV AVVDISVGRI NAWNSD...Q LPIYEPGLDE VVKQCR.GKN

HomSapUGD1 IAHMCPEIRV TVVDVNESRI NAWNSP...T LPIYEPGLKE VVESCR.GKN

OviAriUGD1 IAHMCPEIRV TVVDINESRI NAWNSP...T LPIYEPGLKE VVESCR.GKN

FelCatUGD1 IAHMCPEIRV TVVDVNESRI NAWNSP...T LPIYEPGLKE VVESCR.GKN

EquCabUGD1 IAQMCPEIRV TVVDVNESRI NAWNSP...T LPIYEPGLKE VVESCR.GKN

CanFamUGD1 IAHMCPEIRV TVVDVNESRI NAWNSP...T LPIYEPGLKE VVESCR.GKN

DroMojGI7 MALKCPDISI TLVDKSAERI AQWNSE...K LPIYEPGLDE VVKKCR.NVN

DroPseGA34 MALKCPDIVI TLVDKSVERI AQWNSD...K LPIYEPGLDE VVKKCR.NVN

HomSapICRA IAHMCPEIRV TVVDVNESRI NAWNSP...T LPIYEPGLKE VVESCR.GKN

ColLivUGD IAQMCPNIKV TVVDVNEARI NAWNSD...A LPIYEPGLKE VVESCR.GRN

CamFloUGD IALKCPEIQV TVVDKSKERI AQWNSQ...K LPIYEPGLDE VVQKCR.GTN

BosTauUGD IAHMCPEIRV TVVDINESRI NAWNSP...T LPIYEPGLKE VVESCR.GKN

MelGal1UGD IAQMCPKIQV TVVDVNEARI NAWNSD...T LPIYEPGLKE VVESCR.GRN

AilMelUGDP IAHMCPEIRV TVVDVNESRI NAWNSP...T LPIYEPGLKE VVESCR.GKN

MusFurUGDP IAHMCPEIRV TVVDVNESRI NAWNSS...T LPIYEPGLKE VVESCR.GKN

MusFur1UGD IAHMCPEIRV TVVDVNESRI NAWNSS...T LPIYEPGLKE VVESCR.GKN

MelUnd1UGD IAQMCPNIKV TVVDVNEARI NAWNSD...T LPIYEPGLKE VVESCR.GRN

FalPer1UGD IAQMCPSIKV TVVDVNEARI NAWNSD...T LPIYEPGLKE VVESCR.GRN

ChrBel2UGD IAHMCPKIKV TVVDVNEARI NAWNSD...T LPIYEPGLKE VVESCR.GKN

FelCat1UGD IAHMCPEIRV TVVDVNESRI NAWNSP...T LPIYEPGLKE VVESCR.GKN

SaiBolUGDP IAHMCPEIRV TVVDVNESRI NAWNSP...T LPIYEPGLKE VVESCR.GKN

DroWilGK1 MALKCPDIVI TLVDKSSERI AQWNSD...K LPIYEPGLDE VVKKCR.NVN

CaeRemQV40 IAHKCPHITV TVVDMNTAKI AEWNSD...K LPIYEPGLDE IVFAAR.DRN

CaeBriQV40 IAHKCPHVTV TVVDMNKDKI AEWNSD...K LPIYEPGLDE IVFAAR.GRN

ZeaMayUGDI IALKCPDIEV VVVDISKPRI EAWNSD...T LPIYEPGLDD VVKQCR.GRN

GalGalUG IAQMCPKIQV TVVDVNEARI NAWNSD...T LPIYEPGLKE VVESCR.GRN

CaeEleUG IAHKCPHITV TVVDMNTAKI AEWNSD...K LPIYEPGLDE IVFAAR.GRN

PseAerGMD LSARG..HEV IGVDVSSTKI DLINQG...K SPIVEPGLEA LLQQGRQTGR

CanCloGM LAQNG..FEV IGIDVNKVKV DLINRG...L PTIIEKDIDI ILKEQHKAGR

VibCraGMD FASRK..KQV VGVDVNQHAV DTINQG...K IHIVEPELDM IVNAAVTEGY

PseSyrGMD LSARG..HDV VGVDISSTKI DLINNG...K SPIVEPGLEE LLQKGLATGK

AmyAzuGMD LAGQG..HEV VGVDVNPVKI DLISGG...K APVVEERIGE LTAEVVAQGR

OxaAB1GMD8 LATDG..HEV VGVDPNLTKV DLINQG...V TPIIEKDIGE MIAATVKSGH

ActGloGDM LAERG..HEV VGVDVNPTKV ELLSRG...Q APVVEERIGE LTAEVVASGA

AmyAlbGDM7 LAGRG..HEV VGVDVNPVKI DLISGG...K APVVEERIGE LTAEVVAQGK

AmyBalGDM LAGQG..HRV VGVDVNPVKI DLITGG...N APVVEERIGE LTAEVVASGA

PseDC3GMD5 LSARG..HDV VGVDISSTKI DLINNG...K SPIVEPGLEE LLQKGLATGK

PseVerGMD1 LSARG..HEV VGVDISKEKI DLINAG...K SPIVEPGLGE LLSQGIETGR

PseQDAGMD1 LTARG..HEV IGVDVSSTKI DLINQG...K SPIVEPGLEA LLQQGIANGR

PseAerGMD1 LSARG..HEV IGVDVSSTKI DLINQG...K SPIVEPGLEA LLQQGRQTGR

PseHYSGMD1 LSARG..HQV LGVDISPAKI DMINQG...K SPIVEPGLEQ LLLDGVRHGR

AmyAlbGMD6 LAGRG..HEV VGVDVNPVKI DLISGG...K APVVEERIGE LTAEVVAQGK

AmyAzuGMD1 LAGQG..HEV VGVDVNPVKI DLISGG...K APVVEERIGE LTAEVVAQGR

AlgSagHYPO FAKLG..YTV IGVDKFLHKV EGLNSA...K LNFVEPNLQQ SLREQLDSGR

AciKBSHYPO LARLG..YRV TGVDRDEFKV RSVNEG...C APFYEPGLEE IVRDTVARGR

AmyBalGMD LAGQG..HRV VGVDVNPVKI DLITGG...N APVVEERIGE LTAEVVASGA

StrCanGMD1 LAGMG..HEV IGVDVNQVKV DLVNDG...K APVVEERIGE LTAEVVRKGA

PseAlcGMD1 LSARG..HEV IGVDVSQTKI DLINQG...K SPIVEPGLAE LLEAGVNSGL

ActGloGMD6 LAERG..HEV VGVDVNPTKV ELLSRG...Q APVVEERIGE LTAEVVASGA

OxaAB1GMD1 LATDG..HEV VGVDPNLTKV DLINQG...V TPIIEKDIGE MIAATVKSGH

PseAerUNPP LSARG..HEV IGVDVSSTKI DLINQG...K SPIVEPGLEA LLQQGRQTGR

HahGanHYPO MAARG..HQV IGVDVSEDKV ALINSG...K SPIVEPGLEE LLSKAVSEKL

DesSalNSD LAKEG..HEV VGVDPNQVKV DMINSG...Q TPIIEDLIGD ILQQAVQDGS

PseTomGMD1 LSARG..HEV VGVDISSTKI DLINNG...K SPIVEPGLEE LLQKGITTGK

MarAlgGMD LAQRG..HHV VGVDVSPIKI DLINSG...R SPIVEPGLEE LLRSGRENGF

PseSyrGMD3 LSARG..HDV VGVDISSTKI DLINNG...K SPIVEPGLEE LLQKGLATGK

NovAroGMD IASQG..HHV VGIDVNLGKV ETLNTG...K SPVYEPGLDD LIAKARAEGR

AzoSpGMD LARDG..HQV VGVDIDATKL ELIRSG...T TPVVEEGMVE LMANVAASGR

AzoVinGMD LSGRG..HEV VGVDISAAKI DMINQG...K SPIVEPGLGE LLAEGVKTGR

BacThuGMD FAKAG..FKV TGVDVNPLII DTVNDG...K IHIEEVGLGE LVKEEVSKGN

HahGanHYPP MAARG..HQV IGVDVSEDKV ALINSG...K SPIVEPGLEE LLSKAVSEKL

AlgSagHYPP FAKLG..YTV IGVDKFLHKV EGLNSA...K LNFVEPNLQQ SLREQLDSGR

AciKBSHYPP LARLG..YRV TGVDRDEFKV RSVNEG...C APFYEPGLEE IVRDTVARGR

PseSyrGMD2 LSARG..HEV VGVDISSTKI DLINNG...K SPIVEPGLEE LLQKGISTGK

PseSyrGMD1 LSARG..HEV VGVDISSTKI DLINNG...K SPIVEPGLEE LLQKGISTGK

StrCanGMD LAGMG..HEV IGVDVNQVKV DLVNDG...K APVVEERIGE LTAEVVRKGA

PseVerGMD LSARG..HEV VGVDISKEKI DLINAG...K SPIVEPGLGE LLSQGIETGR

PseQDAGMD LTARG..HEV IGVDVSSTKI DLINQG...K SPIVEPGLEA LLQQGIANGR

PseHYSGMD LSARG..HQV LGVDISPAKI DMINQG...K SPIVEPGLEQ LLLDGVRHGR

PseAlcGMD LSARG..HEV IGVDVSQTKI DLINQG...K SPIVEPGLAE LLEAGVNSGL

PseAerUPP LSARG..HEV IGVDVSSTKI DLINQG...K SPIVEPGLEA LLQQGRQTGR

StaAurUMD FAKHG..VDV LGVDINQQTI DKLQNG...Q ISIEEPGLQE VYEEVLSSGK

EscColUMND ASRQ...KQV IGVDINQHAV DTINRG...E IHIVEPDLAS VVKTAVEGGF

LusFleUGD FASHG..VEV RGIDTKKEVV DKLNTG...Q IHIEEEGLQS LFEEVVNNGM

SalEntUMD FASRQ..KQV IGVDINQHAV DTINRG...E IHIVEPALGN VVKMAVEGGF

ActSerUG FARCG..KSV LGVDINPDVV TKINQG...R IHFVEPQLEE AVKQAVENGL

ErwTasUG FASCQ..RQV IGIDIKEHAV ATINRG...E VHFAEPDLDR AVKAAVEGGF

FusUlcUMD FARAG..FTV NGFDVNKKVI ETLKNG...H IHIVEPDLQE AFEEALGSGR

CorMarUMD MANSG..LSV SGVDVVAEKV EKINRG...E VTIVEPGLEE QLKKALHSGR

StaVitUMD FAKHG..VDV VGVDINKEAV DKLNNG...Q IHIEEPGLQE AYEEVLSQNK

SheVioUMD IAGNG..IRV KGVDVSEYVV NCINQG...K IHIVEPGLEL LVKTSVEAGF

SerS4UMD FASRK..KKV VGVDVNQHAV DTINRG...A IHIVEPDLDK VVKDAVDGGY

PseStuUMD FASRK..KQV IGVDVNEKAV ETINRG...E IYIVEPDLDM VVHAAVTEGH

PsePseUMD FASRK..LEV IGVDVNQHAV DTINRG...E IHIVEPDLDM VVHAAVTEGY

PseFluUMD FASRK..KQV IGVDVNSDTV AIINKG...S IHIVEPDLDM VVHAAVTSGY

HalSalUMD LART...RDV VGVDIDEDVV AAVNAG...E LPFEEPGLSD LFADVE.A.N

FraPhiUMD FARGG..MHI KGVDVNQHAV DTINDG...R IHIEEPGLAE LIKDVVGKGL

EdwC07UMD FAGCR..KQV IGVDVSQHAV ETINRG...E IHIVEPDLDR AVKRAVEGGF

AerAquUMD IASNG..IRV IGVDVNQHAV DTINQG...K IHIVEPGLED LVKQAVQDGH

GeoWSUUMD KAKAG..FHV IGFDIQQSRV DQVNNG...I NYIGDVVDED LHEMVK.QGR

EscMG1UMD ASRQ...KQV IGVDINQHAV DTINRG...E IHIVEPDLAS VVKTAVEGGF

DicZeaUMD LAARQ..QSV FGVDVNPHVV ETVRQG...H THIAEPGLAQ TLQQAVAQGY

VibOrdUMD FASRK..KKV IGVDVNQHAV DTINEG...K IHIVEPELDM IVHAAVKEGY

VidVulUMaD FASRK..KKV IGVDVNQHAV DTINQG...K IHIVEPDLDM MVHAAVSGGY

KlePneUMaD FASRQ..KRV VGVDVNQHAV ETINRG...E IHIVEPDLAS VVKTAVEQGY

GeoTheUMaD FAAAG..FDV VGVDVSETVV ATINSG...N IHIEKTGLAE LVNRVVKEGK

BurXenUMaD FAARR..KKV IGVDVSQRAV DTINKG...E IHIVEPELDM LVHAAVTQGY

HasParUMaD FALAG..QQV IGVDCNPHVI EQINQG...R SHLHEPELAQ AVLQAVSSGH

RalSolUMaD LASRQ..REV IGVDINQHAV DTINQG...R IHIVEPDLDM LVRAAVSQGY

PolIrgUMaD AASKG..IEV IGVDVNLEVV DTINKG...K IHIVEPDLDK VVKEVVEKGL

RalSolNGD LASRQ..REV IGVDINQHAV DTINQG...R IHIVEPDLDM LVRAAVSQGY

YerEntUMaD FASRK..KKV IGVDVNAHAV ETINRG...A IHIVEPDLDK VVKIAVEGGY

EscFerUMaD FASRQ..KQV IGVDINQHAV DTINRG...E IHIVEPDLAS VVKTAVEGGF

OceGraUMaD FASRR..ISV TGVDVDRSVV ERVNKG...Q IHIVEPDLDI VVQAVVSQGH

BacNRRUMaD KAKAG..YKT IGFDVQKSKV EMVNEG...K NYIGDVVNED L.ESLVNSGY

GeoKauUMaD KAKAG..FHV IGFDIQQSRV DQVNNG...I NYIGDVVDED L.HEMVKQGR

PsyCNPUMaD IASNG..IRV KGIDVNQHVI DIINQG...K IHIVEPGLEE LVKSVVAEGY

MorPE3UMaD FASRK..KQV IGVDVNQHAV DTINRG...E IHIVEPELDM IVHATVTEGY

SerProUMaD FASRK..KKV VGVDVNQHAV DTINRG...A IHIVEPDLDK VVKDAVDGGY

151 200

StrPyoUGD IKATLDSKAA YKEAELVIIA TPTNYNSR.. ..INYFDTQH VETVIKEVLS

HymAerUGD1 LHFSTDLGAA IKDCDVAFIA VGTPPGE... ..DGSADLKY VLAVARGIGE

MetCapUGD1 LQFTTDIDLG VSHGLFQFIA VGTPPDE... ..DGSADLQY VLAVARSIGE

ActGloUGD6 LTFTTSYAEA VPGADVVFVA VQTPSAD... ..DGSPDLRY LRSAAESVAQ

HalZhaHYPO LSFTTQSEEA VRFGTLQFIA VGTPPGE... ..DGSADLQY VLQVAETIGR

MCIThaHYPO LF.ITDKLSS INNCDFIFIT VGTPQKKN.. ...GEIDLSF IKTVVRSVGK

SalPacUGD LRFTTDFAAA ATFGDVHFLA VGTPSGL... ..DGTYDLTQ ISAAIERLAP

NatGarNSD2 ISYTTD.ATD IGAADYVIIA VPTPIDD... ..DDRPDLGY VESAATTVGS

BacSubUGD2 LSFSSEVKSS MEECPVIFIA VGTPPRS... ..DGSADTKA LQSVIGDLSE

AzoTolUGD LRASLDKRDA YLGAKYVIIA TPTDYD.... PETNYFNTES VESVIRDVMT

LacCreEPS LTATVDGEKA YKEAEYVIIA APTNYD.... SASHHFDTSA VEAVIELVMK

AlaProUG LTATLDKRVA YSGADFVVVA TPTDYD.... PSTNRFDTST VDSVVSDAME

StrZooUG LTATLDGASA YSNADLIIIA TPTNYD.... SERNYFDTRH VEEVIEQVLD

StrHGBNSD YRPTCD.PGH LQGFDVAVVC VPTPLR.... ..DGAPDLTH VEQAARLLSG

NatGarNSD LRATTEY.DA VRETEVTFLC LPTPQTDD.. ...GSLDLAI MEAGAESLGR

TheMelUGD LKATLDKYEA YKNSEYVIVA TPTNYD.... PEKNYFDTSS VEQVIQDVLE

SulDenUGD FRATLDKQEA YIGATYVIIA TPTDYD.... TQTNYFNTKS IEYVIADVLS

SphAlaUGD LVATTDKQAA YADANFVIVA TPTDYD.... PDTNYFNTNS VESVIADALK

SheOneUGD LRATMDK.NA VKDADFVIIA TPTDYD.... PQTNYFNTQS VENVAKEVLS

SalSerUGD FRATLDKFDA YQNADYVIIA TPTDYD.... PKTNYFNTSS VESVIQDVIS

PseHalUGD FTATTNKSMA YCNADFIIIA TPTDYD.... THTHNFNTSS VEAVINDALA

ProMirUGD FTATLDKEFA YKDAEFVIIA TPTDYD.... PKTNYFNTSS VESVIRDVLE

ParDisUGD LTATLNGATA YRDADFVVIA APTNYD.... PVKNYFDTSH VEEVIELVKS

ParDisUGD1 LTATLDGSEA YKNADFVVIA TPTNYD.... SAKNYFDTSH VEEVIKLVKQ

LacSalUGD LQATTDGKEA YCQADIVIIA TPTNYD.... VVKNYFDTSS VESVIEMVLS

CloPerUGD LIATIDGESA YKNADFVVIA APTNYD.... SKKNYFDTSA VESVIELVLK

ArcButUGD FKATLDKNEA YKDADFIIIA TPTDYD.... EKTNYFNTKS VEAVIKDVLE

AerHydUGD LRATLNKEEA YQGADFVIIA TPTDYD.... PETNYFNTST VESVIKDVMA

LacJohSBP LKATTDAESA YKDADFVIIA TPTNYD.... SEKNFFDTSA VEAVIKLVKQ

StrUbeUGD LRATLDADQA FRDADILIIA TPTNYD.... VEKNFFDTSH VETVIEKALA

ReiMEDNSD FSATLDAEAA YQGAEFVIIA TPTDYD.... PDTNYFNTGS VEAVIKQVMA

ExiSibNSD LHATTDNFEA YKDAEFVIIA TPTDYD.... PTRNYFNTRT VESVIATVLA

RumObeHYPO LTATLDGEAA YTNADYIVIA APTNYD.... SQKNYFDTSA VEAVIELVLK

RumObeHYP1 LTATLDAESA YKDADFVVIA APTNYD.... SKKNFFDTSA VEAVIKLVIE

FaePraHYPO LTATLDAEAA YKDADFVVIA APTNYD.... SKKNFFDTSA VEAVIKLVIQ

EubDolHYPO LTATLDANEA YKDAKFVVIA APTNYD.... SKKNFFDTSA VESVIKLVME

BacPleHYPO LIATLDGASA YKNADFVVIA APTNYD.... PVKNYFDTSH VEEVIDLVLE

BacCopHYPO LTATLDGASA YREADFVVIA APTNYD.... PVKNYFDTSH VEEVIDLVLE

StrTheESP LTATLDAKEA YSDADFVVIA APTNYD.... SKKNFFDTSA VEAVIKLVIE

StrIniCPS LKATLDAESA YQDADFVIVA APTNYD.... SKRDFFDTKA VEAVIEAVLA

SerProUGD LQFTTDAKAG VEHGTIQFIA VGTPPDE... ..DGSADLKY VTAVARTIAE

GeoMetUGD LSFTTDLASA VKESLICFIA VGTPPGE... ..DGSADLQH VLAVAREIGR

GeoUraUGD LSFTTDLASA VKASLVNFIA VGTPPGE... ..DGSADLQY VLDVARTIGR

AnoFlaUGD LFFTTDGARA YRESDVIYIA VGTPENE... ..DGSANLTY LKQAVRDIAS

PorGinNSD LRFGTEIEQA VPEADIVFIA VGTPAGE... ..DGSADMGY VLDAARSIGR

BacSelRPON INYTTNYKSD YKDVDAIFIG VGTPEQP... ..DGSANLSY IATVARQIAE

PelPhaNSD LRFTTDLREG VEFGLYQFIA VGTPPDE... ..DGSADLRH VLSVAESIGT

MetPopNSD LAFSTDLKPA VAGAQAVFIA VGTPSRR... .GDGFADLSY VYAAAREIAE

MetExtNSD LTFSTDLKPA VAQAQAVFIA VGTPSRR... .GDGFADLSY VYAAAREIAE

VibVulPRE LTATTSPQIA YHDAQLIIVA TPTNYD.... PITNGFDTVS VEQVIQDAIQ

GeoKauNSD LRFTTDDAEA YRWAEVIMVA VGTPPLP... ..DGSVKLDD VWETLRRIAA

RhiTriEXO LSFSTDVGES VRSADVVFIA VGTPSRR... .GDGHADLSY VYAAAREIAT

ParDisUGD2 LKATLDAVMA YKDADFVVIA APTNYD.... SQRNFFDTSA VEAVIEQVLR

XanAxoUGD FKATLDANEA FAGAEFVIIA TPTDYD.... PDTNYFNTGS IEVVIAKVLE

XanCamUGD FKATLDAKEA FAGAEFVIIA TPTDYD.... PDTNYFNTGS IEVVIAKVLE

EntFaeUGD LMATVDGVSA YKDADFVVIA APTNYD.... SKKNFFDTSA VEAVIKLVME

StrPneUGD2 LEATMDAKYA YEDAEFVVVA APTNYD.... SKKNFFDTSA VEAVIQQVKK

StrPneUGD3 LVATLDAKEA YKDADFVVIA APTNYD.... PKKNFFDTSA VESVIETVLK

StrPneUG LVATLDAKEA YQDADFVVIA APTNYD.... PKKNFFDTSA VESVIETALK

EscAlbUGD FNATLDKNEA YRDADYVIIA TPTDYD.... PKTNYFNTFS VESVIQDVIE

BacTheUGD LTATLDGAKA YSDADFVIIA APTNYD.... PVKNYFDTHH IEDVIDLVLS

SacSpiUGD YLPTTD.QAE LCGFDFASVC VPTPLH.... ..DGAPDLRY VEDAARALST

Aer159PBP LSYTDC.LDD IKHCNFYIIT VPTPITE... ..ENTPDLTP LEKASEALGK

ButSynUGD LRATTDWEAG YRDAEFVVIA APTNYD.... SQKNFFDTSA VEDVIGKVKR

BraJapUGD LDFTTDLSKP VADADAVFIA VGTPSRR... .GDGHADLSY VYAAAKEIAQ

SynCC9UGD LHFSTDVAES IAAADMVFIS VNTPTKTKGL GAGQASDLRW VEACAREVAQ

StePneUGD LEASLDPAHV YKDVEYAIIA TPTNYD.... VDLNQFDTSS VEAAIKTCME

SalAreUGD LTASTSF.DP VQVADVVLVA VGTPIDPK.. .GVLVADQLV AACQQIAPRL

BurYI2NSD MQYSAD.VED LRGCTVFIAT VPTPIDH... ..YKRPDLSP LISASTTIGS

BacCerNSD INYTTDYQSA YKDADAIFIG VGTPEQA... ..DGSANLSY IATVAKQIAE

PaeSanHYPO LEVSPK.YSS IATAEAIIMC VPTPLNP... ..QHEPDMDY LIEASRDISG

Rhi42MUGD LDFSKELAPS VADADVVFIA VGTPSRR... .GDGHADLSY VYAAAREIAA

OpiTAV5NSD LFFSTDIHAS IAKADIIFVS VNTPTKTYGV GAGRAADLRY IESVARTIAE

MarHTCUGD8 LFFSTDVDKA IDESQLIFIS VNTPTKTYGK GKGQAADLKY IELCARNIAK

BacSubUGD LNFETSYENG LAQADIIFIA VGTPQKS... ..DGHANLEH ITDAAKRIAR

NocKunUGD FAATDD.PAC LASARTIVIC VPTPLSP... ..EGGPDLSA VTSAARSIAG

DesHafUGD ITATTD.YAK LAECDVVVIC VPTPLTI... ..TRDPDISY IQASSEQIAK

BacThuUGD LKAGIV.... PETSDIFILA VPTPFKG... ..DHKPDLTY VEQATKTIAP

SphJapUGD LRASLT.... VEDSDVFIIA VPTPVSE... ..DRAPDISY VLKAARTIAP

RicSibUGD LKFTNIYNNE LQNADAIFIT VGTPSKG... ..LGEADLKY VYDAIDKVFE

MetCapUGD LQFTTDIDLG VSHGLFQFIA VGTPPDE... ..DGSADLQY VLAVARSIGE

HymAerUGD LHFSTDLGAA IKDCDVAFIA VGTPPGE... ..DGSADLKY VLAVARGIGE

BacSubUGD7 LNFETSYENG LAQADIIFIA VGTPQKS... ..DGHANLEH ITDAAKRIAR

ActGloUDG9 LTFTTSYAEA VPGADVVFVA VQTPSAD... ..DGSPDLRY LRSAAESVAQ

NatGarNSD5 LRATTEY.DA VRETEVTFLC LPTPQTDD.. ...GSLDLAI MEAGAESLGR

UncBacHYPO LTATLDFAEA IKKTDVSFLI VPTPSQT... ..DGHFSTRY LREALTKLAS

HalZhaHYPP LSFTTQSEEA VRFGTLQFIA VGTPPGE... ..DGSADLQY VLQVAETIGR

StrPneUGD1 LVATLDAKEA YKDADFVVIA APTNYD.... PKKNFFDTSA VESVIETVLK

HoePhoUGD LHFTSDLPAS VSNADAIFIA VGTPSRN... .GDGHADLQY VNAVVADVAK

CapSpuUGD LFFTTDIAEA IKDAEVAFIA VGTPMGD... ..DGSADLQY VLSVAQAIGE

NovAroUGD LSFSTDLAAS IEDAQAIFIA VGTPSRR... .GDGHADLSY VYAVAQELAE

ProMarUGD LNFSTNVSNS IREADMVFIS VNTPIKEKGV GAGETSDLRW VESCARQISE

HomSapUGD LFFSTNIDDA IKEADLVFIS VNTPTKTYGM GKGRAADLKY IEACARRIVQ

MusMusUNA LFFSTNIDDA IREADLVFIS VNTPTKTYGM GKGRAADLKY IEACARRIVQ

DanRerUGD LFFSTDIDSA IKEADLVFIS VNTPTKTYGM GKGRAADLKF IEACARRIVE

XenLaeUGD LFYSTDIDGA IQEADLVFIS VNTPTKTYGM GKGRAADLKY IEACARRIVQ

RatNorUGD LFFSTNIDDA IREADLVFIS VNTPTKTYGM GKGRAADLKY IEACARRIVQ

PonAbeUGD LFFSTNIDDA IKEADLVFIS VNTPTKTYGM GKGRAADLKY IEACARRIVQ

BosTauUGD3 LFFSTNIDDA IKEADLVFIS VNTPTKTYGM GKGRAADLKY IEACARRIVQ

BamOldUGD LFFSNDIEKH VAEADITFVS VNTPTKTRGL GAGKVADLTY WESAARMIAD

PedCorUGD LFFSTDVYAA IKEADLIFIS VNTPTKTYGI GKGRAADLKF VESCARMIAD

ZeaMayUGD8 LFFSTDVEKH VAEADIIFVS VNTPTKTRGL GAGKAADLTY WESAARMIAD

StrPurUGDP LFFSTDVDSA IQEADLIFIS VNTPTKTFGL GKGRAADLKY IEAAARRIAD

CioIntUGDP LFFSTNIDDA IKSADLIFIS VNTPTKTYGI GKGRAADLKY VESCARHIAA

NemVecPPRE LFFSTDIDTA IKDADLIFIC VNTPTKTYGL GKGRAPDLKY IESAARHIAD

CaeEleUGD LFFSSDIPKA IAEADLIFIS VNTPTKMYGR GKGMAPDLKY VESVSRTIAQ

PopTomUGD LFFSKDVEKH VAEADIVFVS VNTPTKTQGL GAGKAADLTY WESAARTIAD

DroMelUGD LFFSTDIETA IKEADLIFIS VNTPTKTCGN GKGRAADLKY VESAARMIAE

AraThaUGD LFFSTDVEKH VREADIVFVS VNTPTKTRGL GAGKAADLTY WESAARMIAD

DroMelUGD1 LFFSTDIETA IKEADLIFIS VNTPTKTCGN GKGRAADLKY VESAARMIAE

NasVitUDG LFFSTDIAQA IEEAELIFIS VNTPTKTFGN GKGRAADLKY VESAARMIAD

CaeBriHYPO LFFSSDIPKA IAEADLIFIS VNTPTKMYGR GKGMAPDLKY VESVSRTIAQ

DroWilGK3 LFFSTDIETA IKDADLIFIS VNTPTKISGN GKGRAADLKY VESAARMIAE

NasVitUGDP LFFSTDIETA IKEADLIFIS VNTPTKTCGN GKGRAADLKY VESAARMIAE

DroVirGJ3 LFFSTDITTA IKDADLIFIS VNTPTKTCGS GKGRAADLKY VESAARMIAE

HomSapUGD2 LFFSTNIDDA IKEADLVFIS VNTPTKTYGM GKGRAADLKY IEACARRIVQ

XenTroUGD LFFSTNINEA IKKADIIFIS VNTPTKTFGM GKGRAADLKY VEACARQIAD

FlaCF1UGD LFFSTNVEKA IDDAQVVFIS VNTPTKTYGK GKGMAADLKY IELCARQIAK

ZeaMayUGD LFFSTDVEKH VAEADIIFVS VNTPTKTRGL GAGKAADLTY WESAARMIAD

MayZebUGD1 LFFSTDIDSA IRDADLVFIS VNTPTKTYGM GKGRAADLKF IEACARRIVE

VitvinUGD1 LFFSTDVEKH VSEADIVFVS VNTPTKTQGL GAGKAADLTY WESAARMIAD

HomSapUGD1 LFFSTNIDDA IKEADLVFIS VNTPTKTYGM GKGRAADLKY IEACARRIVQ

OviAriUGD1 LFFSTNIDDA IKEADLVFIS VNTPTKTYGM GKGRAADLKY IEACARRIVQ

FelCatUGD1 LFFSTDIDDA IKEADLVFIS VNTPTKTYGM GKGRAADLKY IEACARRIVQ

EquCabUGD1 LFFSTNIDDA INEADLVFIS VNTPTKTYGM GKGRAADLKY IEACARRIVQ

CanFamUGD1 LFFSTNIDDA IKEADLVFIS VNTPTKTYGM GKGRAADLKY IEACARRIVQ

DroMojGI7 LFFSTDITTA IKEADLIFIS VNTPTKTCGS GKGRAADLKY VESAARMIAE

DroPseGA34 LFFSTDIETA IKEADLIFIS VNTPTKVSGN GKGRAADLKY VESAARMIAE

HomSapICRA LFFSTNIDDA IKEADLVFIS VNTPTKTYGM GKGRAADLKY IEACARRIVQ

ColLivUGD LFFSTSIDDA IREADLVFIS VNTPTKTYGM GKGRAADLKY IEACARRIVQ

CamFloUGD LFFSTDIDTA IKEADLIFIS VNTPTKTFGN GKGRAADLKY VESAARMIAE

BosTauUGD LFFSTNIDDA IKEADLVFIS VNTPTKTYGM GKGRAADLKY IEACARRIVQ

MelGal1UGD LFFSTSIDDA IREADLVFIS VNTPTKTYGM GKGRAADLKY IEACARRIVQ

AilMelUGDP LFFSTNIDDA IKEADLVFIS VNTPTKTYGM GKGRAADLKY IEACARRIVQ

MusFurUGDP LFFSTNIDDA IKEADLVFIS VNTPTKTYGM GKGRAADLKY IEACARRIVQ

MusFur1UGD LFFSTNIDDA IKEADLVFIS VNTPTKTYGM GKGRAADLKY IEACARRIVQ

MelUnd1UGD LFFSTSIDDA IREADLVFIS VNTPTKTYGM GKGRAADLKY IEACARRIVQ

FalPer1UGD LFFSTSIDDA IREADLVFIS VNTPTKTYGM GKGRAADLKY IEACARRIVQ

ChrBel2UGD LLFSTSIDDA IREADLVFIS VNTPTKTYGM GKGRAADLKY IEACARRIVQ

FelCat1UGD LFFSTDIDDA IKEADLVFIS VNTPTKTYGM GKGRAADLKY IEACARRIVQ

SaiBolUGDP LFFSTNIDDA IKEADLVFIS VNTPTKTYGM GKGRAADLKY IEACARRIVQ

DroWilGK1 LFFSTDIETA IKDADLIFIS VNTPTKISGN GKGRAADLKY VESAARMIAE

CaeRemQV40 LFFSSDIPKA IAEADLIFIS VNTPTKMYGR GKGMAPDLKY VESVSRTIAQ

CaeBriQV40 LFFSSDIPKA IAEADLIFIS VNTPTKMYGR GKGMAPDLKY VESVSRTIAQ

ZeaMayUGDI LFFSTDVEKH VAEADIIFVS VNTPTKTRGL GAGKAADLTY WESAARMIAD

GalGalUG LFFSTSIDDA IREADLVFIS VNTPTKTYGM GKGRAADLKY IEACARRIVQ

CaeEleUG LFFSSDIPKA IAEADLIFIS VNTPTKMYGR GKGMAPDLKY VESVSRTIAQ

PseAerGMD LSGTTDFKKA VLDSDVSFIC VGTPSKKN.. ...GDLDLGY IETVCREIGF

CanCloGM IRATTDYMEA VSNSEVSIIC VGTPSTEK.. ...GHLNLDY IFETAKQIGE

VibCraGMD LKAVIT.... PEAADAFLIA VPTPFLPCKE GEVPAPDLSY IKAASEAIAP

PseSyrGMD LRGTTDFAEA IRATDLSMIC VGTPSKKN.. ...GDLELDY IESVCREIGY

AmyAzuGMD LRATTDVRQA IADSEVSLIC VGTPSAPN.. ...GSLSTAF LERVAEEIGE

OxaAB1GMD8 LRATVDVRDA VMGTDMSLIC VGTPSQLN.. ...GNLDLSH VRKVCEQIGA

ActGloGDM LRATTDVAEA IRATEISLIC VGTPSAAN.. ...GSLSTAY LERVAEEIGA

AmyAlbGDM7 LRATTDVRQA IADSEVSLIC VGTPSAPN.. ...GSLSTAY LERVAEEIGE

AmyBalGDM LRATTDVAEA VADSEISLIC VGTPSAPN.. ...GSLSTVY LERVAEEIGE

PseDC3GMD5 LRGTTDFAEA IRATDLSMIC VGTPSKKN.. ...GDLELDY IESVCREIGY

PseVerGMD1 LRGTTDFAEA IRATDLSMIC VGTPSKKN.. ...GDLELNY IESVCREIGF

PseQDAGMD1 LRGTTDFAEA IRASDVSMIC VGTPSKKN.. ...GDLGLEY IESVCREIGY

PseAerGMD1 LSGTTDFKKA VLDSDVSFIC VGTPSKKN.. ...GDLDLGY IETVCREIGF

PseHYSGMD1 LRGTTDVQAA VLATEMSLLC VGTPSKKN.. ...GDLDLVY MEAVCREIGT

AmyAlbGMD6 LRATTDVRQA IADSEVSLIC VGTPSAPN.. ...GSLSTAY LERVAEEIGE

AmyAzuGMD1 LRATTDVRQA IADSEVSLIC VGTPSAPN.. ...GSLSTAF LERVAEEIGE

AlgSagHYPO LSFTTSVEKA IRDTDMSLIS VGTPSLST.. ...GAVDTTD VCHVLTQIAQ

AciKBSHYPO FDAAISLAEG MADADVAFIC VGTPSEKN.. ...GSLGLEQ LRRVVGEIEA

AmyBalGMD LRATTDVAEA VADSEISLIC VGTPSAPN.. ...GSLSTVY LERVAEEIGE

StrCanGMD1 LRATTDVREA IMDSEVSLIC VGTPSEPN.. ...GSLCTTY LERVTEEIGA

PseAlcGMD1 LRGTTDVGAA VLASELSFIA VGTPSKRN.. ...GDLDLGY MESVCKQIGA

ActGloGMD6 LRATTDVAEA IRATEISLIC VGTPSAAN.. ...GSLSTAY LERVAEEIGA

OxaAB1GMD1 LRATVDVRDA VMGTDMSLIC VGTPSQLN.. ...GNLDLSH VRKVCEQIGA

PseAerUNPP LSGTTDFKKA VLDSDVSFIC VGTPSKKN.. ...GDLDLGY IETVCREIGF

HahGanHYPO LHGTTDVQSA IENSDISMIC VGTPSKRN.. ...GDLDLRY VESVCRDIGR

DesSalNSD LTASTSAEEA ILSSEISFVC VGTPSQFN.. ...GSLDLSY VRRVCEEIGV

PseTomGMD1 LRGTTDFAEA IRATDLSMIC VGTPSKKN.. ...GDLELDY IESVCREIGY

MarAlgGMD INGVTDGHIA VQESELSMIC VGTPSKPN.. ...GDLDLQF VEKVCGDIGN

PseSyrGMD3 LRGTTDFAEA IRATDLSMIC VGTPSKKN.. ...GDLELDY IESVCREIGY

NovAroGMD LHAVTEIGSA LDDCDIAIVC VGTPSGVD.. ...GAHNMSF IVQVSRAIAA

AzoSpGMD VSVTTDVRQA VLDTEISLIC VGTPSAPN.. ...GSQDQGA VLRLARDLGR

AzoVinGMD LRGTTNVTEA VLATELSMLC VGTPSKLN.. ...GDLELDY IEEVCRQMGS

BacThuGMD LNASLK.... PVLADVYIIA VPTPHNYD.. ...HTADLKY VQSAVESIKP

HahGanHYPP LHGTTDVQSA IENSDISMIC VGTPSKRN.. ...GDLDLRY VESVCRDIGR

AlgSagHYPP LSFTTSVEKA IRDTDMSLIS VGTPSLST.. ...GAVDTTD VCHVLTQIAQ

AciKBSHYPP FDAAISLAEG MADADVAFIC VGTPSEKN.. ...GSLGLEQ LRRVVGEIEA

PseSyrGMD2 LRGTTDFAEA IRATDLSMIC VGTPSKKN.. ...GDLELDY IESVCREIGY

PseSyrGMD1 LRGTTDFAEA IRATDLSMIC VGTPSKKN.. ...GDLELDY IESVCREIGY

StrCanGMD LRATTDVREA IMDSEVSLIC VGTPSEPN.. ...GSLCTTY LERVTEEIGA

PseVerGMD LRGTTDFAEA IRATDLSMIC VGTPSKKN.. ...GDLELNY IESVCREIGF

PseQDAGMD LRGTTDFAEA IRASDVSMIC VGTPSKKN.. ...GDLGLEY IESVCREIGY

PseHYSGMD LRGTTDVQAA VLATEMSLLC VGTPSKKN.. ...GDLDLVY MEAVCREIGT

PseAlcGMD LRGTTDVGAA VLASELSFIA VGTPSKRN.. ...GDLDLGY MESVCKQIGA

PseAerUPP LSGTTDFKKA VLDSDVSFIC VGTPSKKN.. ...GDLDLGY IETVCREIGF

StaAurUMD LKVSTT.... PEASDVFIIA VPTPNNDD.. .QYRSCDISL VMRALDSILP

EscColUMND LRASTT.... PVEADRWLIA VPTPFKG... ..DHEPDMTY VESAARSIAP

LusFleUGD FQAQLE.... PEEADAFIIA VPTPNKDD.. .EYKSCDTKY VISALHAILP

SalEntUMD LRATTT.... PVEADAYLIA VPTPFKG... ..DHDPDMAY VEAAAKSIAP

ActSerUG LFATQI.... PDPADVFVIA VPTPLTY... ..NHQPDLQY IKQAVKAISP

ErwTasUG LRAATR.... PQPADAFLIA VPTPFRG... ..DHLPDMAF VQEAAESVAT

FusUlcUMD LLPTDK.... LEKSDVFIIA VPTPFKKE.. HEEKIADLSY VGSGAKEVAE

CorMarUMD FRASTD.... TPRADVYIIA VPTPFTE... ..MRDVDMKY IYSAVEAIAP

StaVitUMD FKASLT.... PEEADAFIIA VPTPNNDD.. .QYESCDISL VMGAVESIVP

SheVioUMD LTAHTE.... SQPADAFLIA VPTPFKG... .DDYEPDLKY IEAAAKALAT

SerS4UMD LQAVTQ.... PLAADAFLIA VPTPFKG... ..DHEPDLAY VEAAAKSLAP

PseStuUMD LRATTK.... PEPADAFLIA VPTPFKD... ..DHEPDLAY IESASKAIAP

PsePseUMD LRASTK.... PESADAFLIA VPTPFKG... .DNHEPDLSY IESASKAIAP

PseFluUMD LRATTI.... PEPADAFLIA VPTPFND... ..DYTPDLGY IESASKAIAP

HalSalUMD VSAQTT...V PSDADAYLVA TPTPLDEV.. ..TDVANLQY VGAAVESVAP

FraPhiUMD LKAYTE.... PQEADAFIIA VPTPFKG... .DDYEPNLDY IESAAKSLAK

EdwC07UMD LRAVMR.... PEPADAFLIA VPTPFKG... ..DHEPDLTY VEAAARSIAP

AerAquUMD LSAHTN.... PQKADAFLIA VPTPFKG... .EEHEPDLKY IESASRALAP

GeoWSUUMD LVATTD.YAR IAEVDAVAIA VPTPLDE... ..HHQPDTSY VENSAKEIAK

EscMG1UMD LRASTT.... PVEADRWLIA VPTPFKG... ..DHEPDMTY VESAARSIAP

DicZeaUMD LQVGSR.... PVPAQAFIIA VPTPLKA... ..DHQPDVSL VQAAALSLAP

VibOrdUMD LKAVVT.... PEAADAFLIA VPTPFLPCRE GEVPAPDLSY IEAASKAIAP

VidVulUMaD LKATTT.... PEAADAFLIA VPTPFLPCAE GDIPPPDLSY IEAASKAIAP

KlePneUMaD LSATTT.... PVEADAYLIA VPTPFKD... ..RHEPDMVF VESAAKSIAP

GeoTheUMaD FRAQLA.... PEEANVFIIA VPTPIHDD.. ...YTANIDY VIEATKSVAP

BurXenUMaD LRATQT.... PEPADAFLIA VPTPFCD... ..GNKPDLSY IEAASRSIAP

HasParUMaD FYASTE.... PTFADAFIIA VPTPLQA... ..NRQPDLTA IQSAVKSIAP

RalSolUMaD LRATTE.... PEPADAFLIA VPTPFLD... ..NKQPDLSY IEAAARAIAP

PolIrgUMaD LKASLM.... PEEADAFFIV VPTPFKQ... ..NKRADISY VESATRMVIP

RalSolNGD LRATTE.... PEPADAFLIA VPTPFLE... ..DKQPDLTY IEAAAKAIAP

YerEntUMaD LQAVTK.... PLAADAFLIA VPTPFKG... ..DHEPDMVY VESAAKSIAP

EscFerUMaD LRASTT.... PVEADAWLIA VPTPFKG... ..DHEPDMTY VESAARSIAP

OceGraUMaD LRASLT.... PEPAEAFLVA VPTPFKG... .DNYEPDLSF IKAAADAIAP

BacNRRUMaD LSATTN.FEK VAQADCVSIC VPTPLDK... ..YQQPDISY VKTSAESIVP

GeoKauUMaD LMATTD.YAR IAEVDAVAIA VPTPLDE... ..HHQPDTSY VENSANEVAK

PsyCNPUMaD LTAHTE.... PQSADAFLIA VPTPFKG... .ADHEPDLKY IEAASKALAS

MorPE3UMaD LKAVTS.... PEPADAFLIA VPTPFKVCAE GEIPEPDLRY IEAASKAIAL

SerProUMaD LQAVTQ.... PLAADAFLIA VPTPFKG... ..DHEPDLAY VEAAAKSLAP

201 250

StrPyoUGD V.....NSHA TLIIKSTIPI GFITEMRQKF QT........ ..........

HymAerUGD1 NMS....SYG VIVTKSTVPV GTAAKVRHEI EQALE..... ...KRGADID

MetCapUGD1 RMQ....EYR VVVNKSTVPV GTADKVRGAV SEVLA..... ...RRGVAIE

ActGloUGD6 HLD...HTFT VVVNKSTVPI GSGNWVDAIL RESFADRA.. ...DRPANCE

HalZhaHYPO HMD....DYK VVINKSTVPV GTAEKVQVKI AKTLQ..... ...ERGIELD

MCIThaHYPO IISKN.KKKP VILIKSTVIP GTMKDVILPI LEKNS..... ...KKKAGKD

SalPacUGD HLQ....RPC LIVGKSTVSP GTAEVVEAQA RKL....... ....SPAGDD

NatGarNSD2 KM....GPGT TVVLESTVYP GTTRETLVPA LEDAS..... ...GLTAGTD

BacSubUGD2 SIR....SYK TIITKSTVPP GTNENIAKQL IAS....... ....GVSRNL

AzoTolUGD I.....TPSA VMVIKSTVPV GFTVKMKAKF GC........ ..........

LacCreEPS V.....NPNA WMIIKSTIPV GYTESVRKKY GT........ ..........

AlaProUG L.....NTDA LVIIKSTIPV GHTKSLQENH KT........ ..........

StrZooUG L.....NASA TIIIKSTIPL GFIKHVREKY QT........ ..........

StrHGBNSD HL....RPGC LVVLESTTYP GTTEEVFRPL LEGGG..... ...ALRAGRD

NatGarNSD ALAEK.DDEH LVVVKSTVLP GTTEDVVGPV LERES..... ...GTEIGDG

TheMelUGD I.....NSNA IIIIKSTIPV GFTKEMRKKY KT........ ..........

SulDenUGD I.....NPDA VMVIKSTVPV GYVNSLKEKF KT........ ..........

SphAlaUGD L.....APGA LVVVKSTVPV GFTERMRAEH GS........ ..........

SheOneUGD L.....NPDT TIIIKSTVPV GFTASLRTRL NS........ ..........

SalSerUGD I.....NPAA VMIIKSTVPV GFTTAMRQKF AT........ ..........

PseHalUGD Y.....NPHA VIIVKSTVPV RFTQRMKAQL GV........ ..........

ProMirUGD Y.....NPNT TMIIKSTIPV GFTKQMREKY HT........ ..........

ParDisUGD V.....NPNA IMVIKSTIPV GYTESVRKKL DT........ ..........

ParDisUGD1 V.....NPTA IMIIKSTIPV GFTEKIRKNL NT........ ..........

LacSalUGD V.....NTHA TIVIKSTIPV GYTESVREKY QT........ ..........

CloPerUGD V.....NPDA IMVIKSTIPV GYTDSVRKKY NT........ ..........

ArcButUGD I.....NPNA TMVIKSTVPV GYTKEVRERF ET........ ..........

AerHydUGD I.....NPDA TMVIKSTIPV GFTARLREEL GC........ ..........

LacJohSBP Y.....NPQA IMIIKSTIPV GYTNHVRKEF ET........ ..........

StrUbeUGD L.....NSQA LLVIKSTIPL GFIKKMRQKY QT........ ..........

ReiMEDNSD V.....NPEA VMVIKSTIPV GYTAKLKAEM GT........ ..........

ExiSibNSD I.....NPDA TMVIKSTVPV GYTQELKEKF DT........ ..........

RumObeHYPO V.....NPHA IMVIKSTIPV GYTESVRKKY NT........ ..........

RumObeHYP1 Y.....NPDA IMVIKSTIPV GYTASIREKF HC........ ..........

FaePraHYPO Y.....NPDA IMVIKSTIPV GFTASVREKY HC........ ..........

EubDolHYPO I.....NPEA IMVIKSTIPV GYTKSIREKT GS........ ..........

BacPleHYPO V.....NPDA VMVIKSTIPV GYCRSLYVKY AEKFRSV... ...PALAGKK

BacCopHYPO V.....NPDA VMVIKSTIPV GYCRSLYVKY AEKFRTS... ...PALAGKK

StrTheESP Y.....NPEA VMVIKSTIPV GYTASVREKF HC........ ..........

StrIniCPS V.....NTQA IIIIKSTIPV GYTDSIRQKF NT........ ..........

SerProUGD HMT....DHK VVIDKSTVPV GTADKVRQVM EETLR..... ...KRGSTVP

GeoMetUGD NME....GFK IIVDKSTVPV GTADKVRRAA QEELD..... ...RRGAAYE

GeoUraUGD NME....SFK ILVDKSTVPV GTADKVRAVV NEELE..... ...KRHATIE

AnoFlaUGD AVM....KDV IVVTKSTVPV GTNHEIQRMF QQ........ ....LAPHVR

PorGinNSD AMS....RYI LIVTKSTVPV GSYRLIRKVI QEELD..... ...KREVLID

BacSelRPON SVE....KDC LVVVKSTVPV GTNDKVEQFI QDF....... ....LINDVK

PelPhaNSD YMQ....EYR IVINKSTVPV GTADLVREKI GSVLV..... ...ERNVDID

MetPopNSD ALT....GYA VVVTKSTVPV GTGDEVERIL REA....... .....RPDLD

MetExtNSD ALT....GYT VVVTKSTVPV GTGDEVERIL REA....... .....RPDID

VibVulPRE I.....SPNA TILIRSTVPV GFTERMRTQV ER........ ..........

GeoKauNSD SAE....RDC LVAIKSTVPV GTGDEAARFL AEH....... ....GRGGVR

RhiTriEXO YVE....GFT VIVTKSTVPV GTGDEVERIM RET....... .....NPAAD

ParDisUGD2 F.....NPNA FMVIKSTIPV GYTISVRERF HT........ ..........

XanAxoUGD I.....NPQA TMVIKSTIPV GYVAKVRAQF GT........ ..........

XanCamUGD I.....NPQA TMVIKSTIPV GYVAKLRAQF GT........ ..........

EntFaeUGD V.....NPEA IMVIKSTIPV GYTASIREKT GS........ ..........

StrPneUGD2 I.....NPKA VIIIKSTIPV GFTESVRKKY QY........ ..........

StrPneUGD3 Y.....NPDA IMVIKSTVPV GYTESARKKF QT........ ..........

StrPneUG Y.....NPDA IMVIKSTVPV GYTESARKKF QT........ ..........

EscAlbUGD I.....NPLA VMVIKSTVPV GFTAAMQTKY HT........ ..........

BacTheUGD V.....NPDA VMVIKSTIPV GYCRGLYLKY ARK....... ......EVKK

SacSpiUGD HI....TPGC CVVLESTTYP GTTEEIFVPI LETGS..... ...GLRAGND

Aer159PBP II....KQGD IVVFESTVYP GATEEVCLPI IEKIS..... ...GLKFNND

ButSynUGD V.....NPGA VMVIKSTIPV GYTRSVREKF ST........ ..........

BraJapUGD SLQ....GFT VVVTKSTVPV GTGDEVERII RET....... .....NPKAD

SynCC9UGD AA....TEHT IVVEKSTLPV RTAAAIKTIL QAAS...... ...NGEGQRT

StePneUGD Y.....NDTC TIVIKSTIPE GYTKEVRKKF NT........ ..........

SalAreUGD R......PRH LVILKSTVAP GTTRTLVAPL LERG...... ...GLVHERD

BurYI2NSD VL....KKGD VVIYESTVYP GATEEECVPV LEKMS..... ...GLKFNED

BacCerNSD SVE....KDC LVVVKSTVPI GTNDKVEQFI QDF....... ....LVNDVK

PaeSanHYPO HLK...AGQL VALESSTYPG TTREVILPLL EQS....... ...GLRIGEE

Rhi42MUGD AVS....GFT VVVTKSTVPV GTGDEIERIF REE....... .....FPEKD

OpiTAV5NSD AA....TTPK IIVEKSTIPV RTAETIQAIL KAN....... .....GNGSS

MarHTCUGD8 VA....KDDK IVVEKSTLPV RTASAIKSIL QNT....... .....GNGVN

BacSubUGD HV....KRDA IVVTKSTVPV GTNDLIEGLI SKH....... ....LAEPVS

NocKunUGD NL....TPGT LVILESTTYP GTTDEVVRPI LEES...... ...GLVAGAD

DesHafUGD YL....KPGQ LVTLESTTYP GTTEEVILPM LEQS...... ...GLKVGKD

BacThuUGD YI....KPGD LVILESTSPV GTTEKVTEWI LEEREELRCD RT.INSNKGV

SphJapUGD VL....KAGD TVILESTSPV GTTEAMRDVI GTMRPDLKMP ...GKGVQGD

RicSibUGD HIN....KDC LIVIKSTVPP GSCSNIIAYL K......... .....SRGFS

MetCapUGD RMQ....EYR VVVNKSTVPV GTADKVRGAV SEVLA..... ...RRGVAIE

HymAerUGD NMS....SYG VIVTKSTVPV GTAAKVRHEI EQALE..... ...KRGADID

BacSubUGD7 HVK....RDA IVVTKSTVPV GTNDLIEGLI SKH....... ....LAEPVS

ActGloUDG9 HLD...HTFT VVVNKSTVPI GSGNWVDAIL RESFADRA.. ...DRPANCE

NatGarNSD5 ALAEK.DDEH LVVVKSTVLP GTTEDVVGPV LERES..... ...GTEIGDG

UncBacHYPO LLSESSKKYH LFVITSTVSP KSIEKDLIPL IEKYS..... ...GRKLNEG

HalZhaHYPP HMD....DYK VVINKSTVPV GTAEKVQVKI AKTLQ..... ...ERGIELD

StrPneUGD1 Y.....NPDA IMVIKSTVPV GYTESARKKF QT........ ..........

HoePhoUGD ALN....GYT VIITKSTVPV GTGDEIERIV QQA....... .....NPDAD

CapSpuUGD TMQ....GEL IVVDKSTVPV GTADKVRATV QTALD..... ...KRGVNYK

NovAroUGD NLK....TPA VIVTKSTVPV GTGDEVERII RES....... .....GTAVR

ProMarUGD IA....EGHT IVVEKSTLPV KTAQTIKDIL FSSNYQ.... .....TSEKS

HomSapUGD NS....NGYK IVTEKSTVPV RAAESIRRIF DANT...... .....KPNLN

MusMusUNA NS....NGYK IVTEKSTVPV RAAESIRRIF DANT...... .....KPNLN

DanRerUGD VS....DGYK IVTEKSTVPV RAAESIRRIF DANT...... .....KPSLN

XenLaeUGD NS....NGYK IVTEKSTVPV RAAESIRRIF DANT...... .....KPDLN

RatNorUGD NS....NGYK IVTEKSTVPV RAAESIRRIF DANT...... .....KPNLN

PonAbeUGD NS....NGYK IVTEKSTVPV RAAESIRRIF DANT...... .....KPNLN

BosTauUGD3 NS....HGYK IVTEKSTVPV RAAESIRRIF DANT...... .....KPNLN

BamOldUGD VS....KSDK IVVEKSTVPV KTAEAIEKIL THN....... .....SKGIN

PedCorUGD IA....EENK IVVEKSTVPV RAAESIMTVL KANQ...... .....RQGVS

ZeaMayUGD8 VS....KSDK IVVEKSTVPV KTAEAIEKIL THN....... .....SKGIN

StrPurUGDP VA....TSDK IVVEKSTVPV KAAQSIQRIL TANT...... .....KPGCR

CioIntUGDP VS....TGFK IVVEKSTVPV RAAASISSIL SSNK...... .....QSSTS

NemVecPPRE VA....EGGK IIVEKSTVPV RAAESITRIL SANA...... .......DKK

CaeEleUGD YA....GGPK IVVEKSTVPV KAAESIGCIL REAQ...... ...KNNENLK

PopTomUGD VS....KSDK IVVGKSTVPV KTAEAIEKIL THN....... .....SKGIK

DroMelUGD IA....QSNK IVVEKSTVPV RAAESIMHIL RANQ...... .....KPGIH

AraThaUGD VS....VSDK IVVEKSTVPV KTAEAIEKIL THN....... .....SKGIK

DroMelUGD1 IA....QSNK IVVEKSTVPV RAAESIMHIL RAN.Q..... .....KPGIH

NasVitUDG IA....TGDK IVVEKSTVPV RAAESIMNIL RANH...... .....KPGVS

CaeBriHYPO YA....VGPK IVVEKSTVPV KAAESIGCIL REAQ...... ...KNNANLK

DroWilGK3 IA....QSNK IVVEKSTVPV RAAESIMHIL RANQ...... .....KPGIH

NasVitUGDP IA....QSNK IVVEKSTVPV RAAESIMHIL RANQ...... .....KPGIH

DroVirGJ3 IA....QSNK IVVEKSTVPV RAAESIMHIL RANQ...... .....KPGIH

HomSapUGD2 NS....NGYK IVTEKSTVPV RAAESIRRIF DANT...... .....KPNLN

XenTroUGD VA....DGYK IVVEKSTVPV RAAEIIRRIF KTST...... .....KPNLS

FlaCF1UGD VS....RDNK IVVEKSTLPV RTAEAIKSIL DNT....... .....GNGVQ

ZeaMayUGD VS....KSDK IVVEKSTVPV KTAEAIEKIL THN....... .....SKGIN

MayZebUGD1 MS....DGYK IVTEKSTVPV RAAESIRRIF DANT...... .....KPSLN

VitvinUGD1 VS....KSDK IVVEKSTVPV KTAEAIEKIL THN....... .....SKGIN

HomSapUGD1 NS....NGYK IVTEKSTVPV RAAESIRRIF DANT...... .....KPNLN

OviAriUGD1 NS....HGYK IVTEKSTVPV RAAESIRRIF DANT...... .....KPNLN

FelCatUGD1 NS....HGYK IVTEKSTVPV RAAESIRRIF DANT...... .....KPNLN

EquCabUGD1 NS....HGYK IVTEKSTVPV RAAESIRRIF DANT...... .....KPNLN

CanFamUGD1 NS....HGYK IVTEKSTVPV RAAESIRRIF DANT...... .....KPNLN

DroMojGI7 IA....QSNK IVVEKSTVPV RAAESIMHIL RANQ...... .....KPGIH

DroPseGA34 IA....QSNK IVVEKSTVPV RAAESIMHIL RANQ...... .....KPGIH

HomSapICRA NS....NGYK IVTEKSTVPV RAAESIRRIF DANT...... .....KPNLN

ColLivUGD NS....NGYK IVTEKSTVPV RAAESIRRIF DANT...... .....KPNLD

CamFloUGD IA....TGNK IVVEKSTVPV RAAESIMNIL RANH...... .....KPGVS

BosTauUGD NS....HGYK IVTEKSTVPV RAAESIRRIF DANT...... .....KPNLN

MelGal1UGD NS....NGYK IVTEKSTVPV RAAESIRRIF DANT...... .....KPNLD

AilMelUGDP NS....HGYK IVTEKSTVPV RAAESIRRIF DANT...... .....KPNLD

MusFurUGDP NS....HGYK IVTEKSTVPV RAAESIRRIF DANT...... .....KPNLN

MusFur1UGD NS....HGYK IVTEKSTVPV RAAESIRRIF DANT...... .....KPNLN

MelUnd1UGD NS....NGYK IVTEKSTVPV RAAESIRRIF DANT...... .....KPNLD

FalPer1UGD NS....NGYK IVTEKSTVPV RAAESIRRIF DANT...... .....KPDLD

ChrBel2UGD NS....NGYK IVTEKSTVPV RAAESIRRIF DANT...... .....KPNLN

FelCat1UGD NS....HGYK IVTEKSTVPV RAAESIRRIF DANT...... .....KPNLN

SaiBolUGDP NS....NGYK IVTEKSTVPV RAAESIRRIF DANT...... .....KPNLN

DroWilGK1 IA....QSNK IVVEKSTVPV RAAESIMHIL RANQ...... .....KPGIH

CaeRemQV40 YA....VGPK IVVEKSTVPV KAAESIGCIL REAQ...... ...KNNENLK

CaeBriQV40 YA....VGPK IVVEKSTVPV KAAESIGCIL REAQ...... ...KNNANLK

ZeaMayUGDI VS....KSDK IVVEKSTVPV KTAEAIEKIL THN....... .....SKGIN

GalGalUG NS....NGYK IVTEKSTVPV RAAESIRRIF DANT...... .....KPNLD

CaeEleUG YA....GGPK IVVEKSTVPV KAAESIGCIL REAQ...... ...KNNENLK

PseAerGMD AIREK.SERH TVVVRSTVLP GTVNNVVIPL IEDCS..... ...GKKAGVD

CanCloGM ALKQK.KSYH TIVIRSTVLP GTNCQVGEII AQIS...... ...NKIRNRD

VibCraGMD VL....KKGD LVILESTSPV GATEQMADWL AQARSDLTFP Q..THGEDAD

PseSyrGMD VLRDK.NTRH TIVVRSTVLP GTVANVVIPI LEDCS..... ...GKKAGVD

AmyAzuGMD ALRDK.AERH TVVFRSTMLP GTCLDLLVPI LEKSS..... ...GRTAGVD

OxaAB1GMD8 AIKEK.DSFH VVVARSTMLP GSMSSVVIPT LEAAS..... ...GKKAGVD

ActGloGDM VLAGL.DRWH TVVFRSTMLP GTCTDLLIPI LEKES..... ...GKTAGVE

AmyAlbGDM7 ALRDK.AERH TVVFRSTMLP GTCLDLLVPI LEKSS..... ...GLTAGVD

AmyBalGDM ALAKK.SERH TVVFRSTMLP GTCLDLLIPI LEKAS..... ...GRTAGVD

PseDC3GMD5 VLRDK.NTRH TIVVRSTVLP GTVANVVIPI LEDCS..... ...GKKAGVD

PseVerGMD1 VLRDK.PTRH TIVVRSTVLP GTVANVVIPI LEDCS..... ...GKKAGVD

PseQDAGMD1 VLRDT.TRRH TIVVRSTVLP GTVKNVVIPI LEDCS..... ...GKQAGVD

PseAerGMD1 AIREK.SERH TVVVRSTVLP GTVNNVVIPL IEDCS..... ...GKKAGVD

PseHYSGMD1 AMRDK.ASRH TVVVRSTVLP GTVKNVVIPI LEKYA..... ...GKRAGID

AmyAlbGMD6 ALRDK.AERH TVVFRSTMLP GTCLDLLVPI LEKSS..... ...GLTAGVD

AmyAzuGMD1 ALRDK.AERH TVVFRSTMLP GTCLDLLVPI LEKSS..... ...GRTAGVD

AlgSagHYPO SLRDK.ESTH LIVMRSTVAP GHGSASFIPL IEAIS..... ...DKTEGEG

AciKBSHYPO LLPHR.DKPL LLVVRSTVFP GTCEEHIIRP LRRF...... ........PG

AmyBalGMD ALAKK.SERH TVVFRSTMLP GTCLDLLIPI LEKAS..... ...GRTAGVD

StrCanGMD1 ALAER.GGRH TVVFRSTMLP GTCLNLLVPI LEKNI..... ...GGTAGVD

PseAlcGMD1 ALRDK.QERH TVVVRSTVLP GTVKNVVIPL IEAAS..... ...GKKAGVD

ActGloGMD6 VLAGL.DRWH TVVFRSTMLP GTCTDLLIPI LEKES..... ...GKTAGVE

OxaAB1GMD1 AIKEK.DSFH VVVARSTMLP GSMSSVVIPT LEAAS..... ...GKKAGVD

PseAerUNPP AIREK.SERH TVVVRSTVLP GTVNNVVIPL IEDCS..... ...GKKAGVD

HahGanHYPO ALRGK.DERH TIVVRSTVLP GTVKNTVIPI LEDCA..... ...QKQAGKD

DesSalNSD VLKNK.DEFH VVVIRSTILP GSMRSLVIPA LEAAS..... ...GKVAGVD

PseTomGMD1 VLRDKA.TRH TIVVRSTVLP GTVANVVIPI LEDCS..... ...GKKAGVD

MarAlgGMD ALKDK.GDWH LVAVRSTVLP GTVREVVIPA LEKAS..... ...GKRAGVD

PseSyrGMD3 VLRDK.NTRH TIVVRSTVLP GTVANVVIPI LEDCS..... ...GKKAGVD

NovAroGMD SVRPDRKTPL TVVYRSTMRP GTTEQMILPI FRSVI..... ...GEDCEKL

AzoSpGMD ALAEK.SAPH VLVFRSTLVP GTVEGSLKPI IETES..... ...GKRDGED

AzoVinGMD ALRDK.TERH TVVVRSTVLP GTVHNVVIPI LEEFS..... ...GKKAGVD

BacThuGMD FL....QKGN ILIVESTIPP RTINDIVAPA IEEA...... ...GWKVGEE

HahGanHYPP ALRGK.DERH TIVVRSTVLP GTVKNTVIPI LEDCA..... ...QKQAGKD

AlgSagHYPP SLRDK.ESTH LIVMRSTVAP GHGSASFIPL IEAIS..... ...DKTEGEG

AciKBSHYPP LLPHR.DKPL LLVVRSTVFP GTCEEHIIRP LRRF...... ........PG

PseSyrGMD2 VLRDK.ATRH TIVVRSTVLP GTVANVVIPI LEDCS..... ...GKKAGVD

PseSyrGMD1 VLRDK.ATRH TIVVRSTVLP GTVANVVIPI LEDCS..... ...GKKAGVD

StrCanGMD ALAER.GGRH TVVFRSTMLP GTCLNLLVPI LEKNI..... ...GGTAGVD

PseVerGMD VLRDK.PTRH TIVVRSTVLP GTVANVVIPI LEDCS..... ...GKKAGVD

PseQDAGMD VLRDT.TRRH TIVVRSTVLP GTVKNVVIPI LEDCS..... ...GKQAGVD

PseHYSGMD AMRDK.ASRH TVVVRSTVLP GTVKNVVIPI LEKYA..... ...GKRAGID

PseAlcGMD ALRDK.QERH TVVVRSTVLP GTVKNVVIPL IEAAS..... ...GKKAGVD

PseAerUPP AIREK.SERH TVVVRSTVLP GTVNNVVIPL IEDCS..... ...GKKAGVD

StaAurUMD FL....KKGN TIIVESTIAP KTMDDFVKPV IENL...... ...GFTIGED

EscColUMND VL....KKGA LVILESTSPV GSTEKMAEWL AEMRPDLTFP Q..QVGEQAD

LusFleUGD YL....KKGN VVIVESTIAP RTMED.VVLP IIEKA..... ...GFSVGED

SalEntUMD VL....KKGA LVILESTSPV GATEQMAGWL AGMRPDLTFP Q..QAGEQAD

ActSerUG YL....VKGN LIVLESTSPV GTTEKLAKWL QKLRPDLSFP T..QGNDNAD

ErwTasUG VL....KKGD LVILESTSPV GSTEQMAQWM AAVRPDLTFP Q..QAGDAAD

FusUlcUMD VL....EENN LVILESTVPP MTTKLMTDIL ERES...... ....GISRDK

CorMarUMD QL....QGDE LIVLESTSPP LTTDKMAQRV LELRPDLAAD EEENPENKPI

StaVitUMD FL....EKGN TVIVESTIAP RTTDDHVKPY LESQ...... ...GFEIGKD

SheVioUMD KL....VKGN LVVLESTSPI GATEKMAAWL HEVRPDLSFP QTVGEGEIAD

SerS4UMD VL....KKGD LVILESTSPV GATEQMAQWL AEARSDLSFP Q..QAGEAAD

PseStuUMD VL....KKGD LVILESTSPV GATEQMAAWL AEARPELSFP Q..THGEDSD

PsePseUMD VL....KQGD LVILESTSPV GATEQMAAWL AEARPDLSFP Q..THGEASD

PseFluUMD VL....KKGD LVILESTSPV GATEQMSAWL ALERPDLSFP Q..DCGEDSD

HalSalUMD HL....DPGD LVVLESTVPP GTSERLVRPI LEREG..... ....PED.GG

FraPhiUMD VL....KKGD LVILESTSPV GATEKMSAWI AEQRPDLTFP Q..THGEDSD

EdwC07UMD VL....KKGD LVILESTSPV GATEQMCAWL AECRTDLRFP H..QDGEQAD

AerAquUMD ML....EKGN LVVLESTSPV GATEQMAKWL AEERSDLTFP HTVAEGQTPD

GeoWSUUMD YA....HEGM LVVLESTTYP GTTEEIVKPA LEKK...... ...GLVVGET

EscMG1UMD VL....KKGA LVILESTSPV GSTEKMAEWL AEMRPDLTFP Q..QVGEQAD

DicZeaUMD VL....KPGD VVILESTSPV GTTEHMAEWL AQARPDLSFP Q..QAGVASD

VibOrdUMD VL....KKGD LVILESTSPV GATEKMADWL AKARSDLTFP Q..THGEDAD

VidVulUMaD VL....KKGD LVILESTSPV GATEKMAAWL AEARSDLTFP Q..TYGEEAD

KlePneUMaD TL....KKGS LVILESTSPV GSTEQMAEWL AEMRPDLSFP Q..QVGEAAD

GeoTheUMaD YL....RKGN VVIVESTIPP RTMDDVVAPI IREH...... ...GFDPEQD

BurXenUMaD AL....KKGD IVVLESTSPV GATEKMSAWL AECRPDLTFP Q..HSGEQSD

HasParUMaD YL....RQGN LVVLESTSPV GTTEQLAQWI EKERPDL... .....SIPTD

RalSolUMaD VL....KRGD LVVLESTSPV GATEQLSDWL SAQRPDLSFP H..QQGEESD

PolIrgUMaD FL....KEGD LYVIESTSPV MTTEKMAELI FTERP..... .....ELKDK

RalSolNGD VL....KRGD LVVLESTSPV GATEQLSAWL SEQRSDLSFP H..QLGEESD

YerEntUMaD VL....KKGD LVILESTSPV GATEQMAQWL AEARPDLSFP Q..NVGEEAD

EscFerUMaD VL....KKGA LVILESTSPV GSTEKMAEWL AEMRPDLTFP Q..QVGEQAD

OceGraUMaD VL....SQGN LVVLESTSPV GATEQLAGWL AAARPDLTFP Q..AHGEDSD

BacNRRUMaD YM....HKNM LIVLESTTYP GTTEELLKPI LEES...... ...GLKCGED

GeoKauUMaD YA....HEGM LVVLESTTYP GTTEEIVKPA LEKK...... ...GLVVGET

PsyCNPUMaD QL....VKGN LVILESTSPV GATEKMAAWL AEARPELSFP HTVKEGQVAD

MorPE3UMaD VL....KKGD LVILESTSPV GATEQMAEWL AEIRPDLSFP Q..QSGENAD

SerProUMaD VL....KKGD LVILESTSPV GATEQMAQWL AEARSDLSFP Q..QAGEAAD

251 300

StrPyoUGD DRIIFSPEFL RESKALYDNL YPSRIIVSCE ENDSPK.... ..........

HymAerUGD1 FDVASNPEFL KEGAAIDDFL KPDRIVVGVA .......... ..........

MetCapUGD1 FDVVSNPEFL KEGAAIEDFM KPDRIVVGTD .......... ..........

ActGloUGD6 FAVASNPEFL REGNAIHDTL FPDRIVIGSD .......... ..........

HalZhaHYPO YDVCSNPEFL KEGAAIEDFT RGARIVVGTR .......... ..........

MCIThaHYPO FGLISNPEFL QESNAIRDTI KPHVIVLGGY .......... ..........

SalPacUGD VQVAWNPEFL REGTAVADTL TPDRIVLGVA .......... ..........

NatGarNSD2 FFVGYSPERA TPGDADHGLE DVVKV.VSAQ .......... ..........

BacSubUGD2 FNVVSNPEFL REGNALYDML HPDKTVIGVQ EE........ ..........

AzoTolUGD ENLIFSPEFL REGRALHDNL YPSRIVVGER .......... ..........

LacCreEPS NKILFSPEFL RESKALYDNL YPSRIIVSCD EE........ ..........

AlaProUG NRVIFSPEFL REGQALKDNL YPSRIIVGSQ .......... ..........

StrZooUG DRIIFSPEFL RESKALYDNL YPSRIIVSYE KDDSPR.... ..........

StrHGBNSD FRLGYSPERI DPGNPSWHLA NTPKI.VSGV .......... ..........

NatGarNSD LALAMNPEFL RMGTAVRDFL EPDKVVFGTA .......... ..........

TheMelUGD DNIIFSPEFL REGKALYDNL YPSRIVVGEK .......... ..........

SulDenUGD QNIIFSPEFL REGLALHDNL YPSRIIVGEQ .......... ..........

SphAlaUGD NDIVFSPEFL REGRALHDNL HPSRIIVGNT .......... ..........

SheOneUGD DNILFSPEFL REGQALYDNL HPSRIIVGER .......... ..........

SalSerUGD ENIIFSPEFL REGKALYDNL YPSRIVIGEQ .......... ..........

PseHalUGD DNIIFSPEFL REGKALYDNL HPSRIVVGEQ .......... ..........

ProMirUGD DNIIFSPEFL REGRALYDNL YPSRIVIGEQ .......... ..........

ParDisUGD ENVIFSPEFL RESKALYDNL YPSRIIVGRP EGDKR..... ..........

ParDisUGD1 ENVLFSPEFL RESKALYDNL YPSRIIVGRP ENDTR..... ..........

LacSalUGD DRIIFSPEFL RESKALYDNL YPSRIIVATD KSDEK..... ..........

CloPerUGD ENIIFSPEFL RESKALYDNL YPSRIIVSTD INNER..... ..........

ArcButUGD SNIIFSPEFL REGKALYDNL YPSRIIVGEQ .......... ..........

AerHydUGD DNLIFSPEFL REGRALYDNL HPSRIVVGER .......... ..........

LacJohSBP ENIIFSPEFL REGHALYDNL HPSRIVVGTD KENTK..... ..........

StrUbeUGD DRIIFSPEFL RESKALKDNL YPSRIIVSFE DDDSME.... ..........

ReiMEDNSD DNLIFSPEFL REGKALYDNL YPSRIIVGER .......... ..........

ExiSibNSD PNIIFSPEFL REGQALHDNL YPSRIVVGEQ .......... ..........

RumObeHYPO ANIIFSPEFL RESKALYDNL YPSRIIVATD PDDPK..... ..........

RumObeHYP1 DNIIFSPEFL RESKALYDNL YPSRIIVGTD VENAR..... ..........

FaePraHYPO DNIIFSPEFL RESKALYDNL YPSRIIVGTD VDNAR..... ..........

EubDolHYPO KNIIFSPEFL RESKALYDNL YPSRIIVGTD MNDER..... ..........

BacPleHYPO FNLLFSPEFL RESKALYDNL YPSRIIVGYP KLIKGL..DE ENKAIKTIAG

BacCopHYPO FNLLFSPEFL RESKALYDNL YPSRIIVGFP KLIAGL..DE ENRAIKEIAG

StrTheESP DNIIFSPEFL RESKALYDNL YPSRIIVGTD VDNAR..... ..........

StrIniCPS TNIIFSPEFL RESRALYDNL YPSRIIVGTD LSNQR..... ..........

SerProUGD FDVVSNPEFL KEGAAVADCM RPERIVIGTD .......... ..........

GeoMetUGD FDVVSNPEFL KEGAAIDDFM KPDRVVIGAD .......... ..........

GeoUraUGD FDVVSNPEFL KEGAAIDDFM KPDRVVIGTD .......... ..........

AnoFlaUGD IDVVSNPEFL REGSAIHDTF HGDRIVIGAD .......... ..........

PorGinNSD FDIASNPEFL KEGNAIDDFM KPDRVVVGVD .......... ..........

BacSelRPON VEVASNPEFL AQGSAVHDTL HAERIIIGTE .......... ..........

PelPhaNSD YDVVSNPEFL KEGDAVNDFM KPERIVVGVD .......... ..........

MetPopNSD IGVASNPEFL REGAAIGDFK RPDRIVIGAE .......... ..........

MetExtNSD VGVASNPEFL REGAAIGDFK RPDRIVIGAE .......... ..........

VibVulPRE RNILFAPEFL REGKALYDNL FPSRIVIGEQ .......... ..........

GeoKauNSD IDVVSNPEFL SQGTAVRDTL QAPRIVLGVE .......... ..........

RhiTriEXO VAVVSNPEFL REGAAIEDFK RPDRIVVGLN .......... ..........

ParDisUGD2 KRILFSPEFL RESKALYDNL YPSRIIVGTD LSDPE..... ..........

XanAxoUGD DNIIFSPEFL REGRALYDNL YPSRIIVGER .......... ..........

XanCamUGD DNIIFSPEFL REGRALYDNL YPSRIIVGER .......... ..........

EntFaeUGD KNIIFSPEFL RESKALYDNL YPSRIIVGTD MEDER..... ..........

StrPneUGD2 KNILFSPEFL RESKALYDNL YPSRIIVGTD LEDTY..... ..........

StrPneUGD3 ENILFSPEFL RESKALYDNL YPSRIILGTD KNNER..... ..........

StrPneUG ENILFSPEFL RESKALYDNL YPSRIILGTD KNNER..... ..........

EscAlbUGD ENIIFSPEFL REGKALYDNL HPSRIVIGER .......... ..........

BacTheUGD LNLLFSPEFL RESMALYDNL YPSRIIVGYP KLIDGEQFDE ENEAIKAIAD

SacSpiUGD FHVGYSPERI DPSNPTWKFH NTPKI.VSGV .......... ..........

Aer159PBP FYAGYSPERI NPGDKVNRLT TIVKI.TSGS .......... ..........

ButSynUGD RNIIFAPEFL RESKALYDNL YPSRIIVGYD QSDKE..... ..........

BraJapUGD VVVASNPEFL REGAAIRDFK FPDRVVVGTS .......... ..........

SynCC9UGD FSVLSNPEFL AEGTAIRDLE APDRVLIGGD .......... ..........

StePneUGD DRIIFSPEFL RESKALYDNL YPSRIVVGTD LDDSE..... ..........

SalAreUGD FGLAVCPERL AEGVALTQVR TLPV.VVGGC .......... ..........

BurYI2NSD FFVGYSPERI NPGDKSHRLP DIKKV.TSGS .......... ..........

BacCerNSD VEVASNPEFL AQGSAVHDTL YAERIVIGTE .......... ..........

PaeSanHYPO LFVGFSPERV NPGSRRYTLQ EIPKI.VSGI .......... ..........

Rhi42MUGD ISVVSNPEFL REGAAITDFK RPDRIVLGTE .......... ..........

OpiTAV5NSD HQVLSNPEFL AEGTAVADLQ NPDRVLIGGE RTPE...... ..........

MarHTCUGD8 FEILSNPEFL AEGTAIDDLL NADRVLIGGD DTPS...... ..........

BacSubUGD ISVASNPEFL REGSAIYDTF HGDRIVIGTA .......... ..........

NocKunUGD FHLAFSPERI DPGNTAFGVA NTPKV.VGGL .......... ..........

DesHafUGD FFLAFSPERV DPGNKRFTTN NTSKV.VGGM .......... ..........

BacThuUGD FFVAHCPERV LPGHILRELV ENDRI.IGGI .......... ..........

SphJapUGD IAIAYCPERV LPGRILVELI DNDRC.IGGI .......... ..........

RicSibUGD FNVASNPEFL REGSAVEDFL YPDRIVIGVN .......... ..........

MetCapUGD FDVVSNPEFL KEGAAIEDFM KPDRIVVGTD .......... ..........

HymAerUGD FDVASNPEFL KEGAAIDDFL KPDRIVVGVA .......... ..........

BacSubUGD7 ISVASNPEFL REGSAIYDTF HGDRIVIGTA .......... ..........

ActGloUDG9 FAVASNPEFL REGNAIHDTL FPDRIVIGSD .......... ..........

NatGarNSD5 LALAMNPEFL RMGTAVRDFL EPDKVVFGTA .......... ..........

UncBacHYPO FGVAYNPEFI ALGSVIRDFL NPDMILIGES .......... ..........

HalZhaHYPP YDVCSNPEFL KEGAAIEDFT RGARIVVGTR .......... ..........

StrPneUGD1 ENILFSPEFL RESKALYDNL YPSRIILGTD KNNER..... ..........

HoePhoUGD FSVVSNPEFL REGAAIRDFL EPDRVLIGAE .......... ..........

CapSpuUGD FHVVSNPEFL KEGKAIEDFM KPDRVVIGAD .......... ..........

NovAroUGD FEVVSNPEFL REGAAIGDFK RPDRIVIGAE .......... ..........

ProMarUGD FSVLSNPEFL SEGTAIRDLE EPDRVLIGGD .......... ..........

HomSapUGD LQVLSNPEFL AEGTAIKDLK NPDRVLIGGD ETPE...... ..........

MusMusUNA LQVLSNPEFL AEGTAIKDLK NPDRVLIGGD ETPE...... ..........

DanRerUGD LQVLSNPEFL AEGTAVKDLK EPDRVLIGGD ETPE...... ..........

XenLaeUGD LQVLSNPEFL AEGTAIKDLK NPDRVLIGGD ETPE...... ..........

RatNorUGD LQVLSNPEFL AEGTAIKDLK NPDRVLIGGD ETPE...... ..........

PonAbeUGD LQVLSNPEFL AEGTAIKDLK NPDRVLIGGD ETPE...... ..........

BosTauUGD3 LQVLSNPEFL AEGTAIKDLK NPDRVLIGGD ETPE...... ..........

BamOldUGD FQILSNPEFL AEGTAIQDLF NPDRVLIGGR ETPE...... ..........

PedCorUGD YQVLSNPEFL AEGTAVKDLL NPDRVLIGGE NTPE...... ..........

ZeaMayUGD8 YQILSNPEFL AEGTAIEDLF KPDRVLIGGR ETPE...... ..........

StrPurUGDP YEVLSNPEFL AEGSAIKDLL EPDRVLIGGD TSHS...... ..........

CioIntUGDP YQVLSNPEFL AEGTAVENLV NPDRVLIGGE KSPE...... ..........

NemVecPPRE FHVLSNPEFL AEGTAIKDLM EPDRVLIGGE QTKE...... ..........

CaeEleUGD FQVLSNPEFL AEGTAMKDLA NPDRVLIGGE SSPE...... ..........

PopTomUGD FQILSNPEFL AEGTAIGDLF QPDRVLIGGR ETPE...... ..........

DroMelUGD YDILSNPEFL AEGTAINDLL NADRVLIGGE ETPE...... ..........

AraThaUGD FQILSNPEFL AEGTAIKDLF NPDRVLIGGR ETPE...... ..........

DroMelUGD1 YDILSNPEFL AEGTAINDLL NADRVLIGGE ETPE...... ..........

NasVitUDG YQILSNPEFL AEGTAIEDLL NADRVLIGGE ESPE...... ..........

CaeBriHYPO FQVLSNPEFL AEGTAMKDLA NPDRVLIGGE SSTE...... ..........

DroWilGK3 YDILSNPEFL AEGTAINDLL NADRVLIGGE ETPE...... ..........

NasVitUGDP YDILSNPEFL AEGTAINDLL NADRVLIGGE ETPE...... ..........

DroVirGJ3 YDILSNPEFL AEGTAINDLL NADRVLIGGE ETPE...... ..........

HomSapUGD2 LQVLSNPEFL AEGTAIKDLK NPDRVLIGGD ETPE...... ..........

XenTroUGD FQVLSNPEFL AEGTAIENLK NPDRILIGGD ETPA...... ..........

FlaCF1UGD FQILSNPEFL AEGTAVTDLL NPDRILIGGD STPD...... ..........

ZeaMayUGD YQILSNPEFL AEGTAIEDLF KPDRVLIGGR ETPE...... ..........

MayZebUGD1 LQVLSNPEFL AEGTAVRDLK EPDRVLIGGD ETAE...... ..........

VitvinUGD1 FQILSNPEFL AEGTAIQDLF NPDRVLIGGR ETPE...... ..........

HomSapUGD1 LQVLSNPEFL AEGTAIKDLK NPDRVLIGGD ETPE...... ..........

OviAriUGD1 LQVLSNPEFL AEGTAIKDLK NPDRVLIGGD ETPE...... ..........

FelCatUGD1 LQVLSNPEFL AEGTAIKDLK NPDRVLIGGD ETPE...... ..........

EquCabUGD1 LQVLSNPEFL AEGTAIKDLK NPDRVLIGGD ETPE...... ..........

CanFamUGD1 LQVLSNPEFL AEGTAIKDLK NPDRVLIGGD ETPE...... ..........

DroMojGI7 YDILSNPEFL AEGTAINDLL NADRVLIGGE ETAE...... ..........

DroPseGA34 YDILSNPEFL AEGTAINDLL NADRVLIGGE ETTE...... ..........

HomSapICRA LQVLSNPEFL AEGTAIKDLK NPDRVLIGGD ETPE...... ..........

ColLivUGD LQVLSNPEFL AEGTAIKDLK NPDRVLIGGD DSPE...... ..........

CamFloUGD YQILSNPEFL AEGTAIEDLV NADRVLIGGE DSPE...... ..........

BosTauUGD LQVLSNPEFL AEGTAIKDLK NPDRVLIGGD ETPE...... ..........

MelGal1UGD LQVLSNPEFL AEGTAIKDLK NPDRVLIGGD DSPE...... ..........

AilMelUGDP LQVLSNPEFL AEGTAIKDLK NPDRVLIGGD ETPE...... ..........

MusFurUGDP LQVLSNPEFL AEGTAIKDLK NPDRVLIGGD ETPE...... ..........

MusFur1UGD LQVLSNPEFL AEGTAIKDLK NPDRVLIGGD ETPE...... ..........

MelUnd1UGD LQVLSNPEFL AEGTAIKDLK NPDRVLIGGD DSPE...... ..........

FalPer1UGD LQVLSNPEFL AEGTAIKDLK NPDRVLIGGD DSPE...... ..........

ChrBel2UGD LQVLSNPEFL AEGTAIKDLK NPDRVLIGGD ETPE...... ..........

FelCat1UGD LQVLSNPEFL AEGTAIKDLK NPDRVLIGGD ETPE...... ..........

SaiBolUGDP LQVLSNPEFL AEGTAIKDLK NPDRVLIGGD ETPE...... ..........

DroWilGK1 YDILSNPEFL AEGTAINDLL NADRVLIGGE ETPE...... ..........

CaeRemQV40 FQVLSNPEFL AEGTAMKDLA NPDRVLIGGE TSPE...... ..........

CaeBriQV40 FQVLSNPEFL AEGTAMKDLA NPDRVLIGGE SSTE...... ..........

ZeaMayUGDI YQILSNPEFL AEGTAIEDLF KPDRVLIGGR ETPE...... ..........

GalGalUG LQVLSNPEFL AEGTAIKDLK NPDRVLIGGD DSPE...... ..........

CaeEleUG FQVLSNPEFL AEGTAMKDLA NPDRVLIGGE SSPE...... ..........

PseAerGMD FGVGTNPEFL RESTAIKDYD FPPMTVIGEL .......... ..........

CanCloGM FSVVSNPEFL REGSAVQDYY NPPYTLIGSD .......... ..........

VibCraGMD INIAHCPERV LPGHVVRELV ENDRV.IGGL .......... ..........

PseSyrGMD FGVAVNPEFL RESTAIKDYD LPPMTVIGEF .......... ..........

AmyAzuGMD FGVAVNPEFL REGSSVKDFF DPPKTVIGEL .......... ..........

OxaAB1GMD8 FGVCNNPEFL REGTAVYDYY HPPKTVIGES .......... ..........

ActGloGDM FGVAVNPEFL REGSSVKDFF DPPKTVIGEL .......... ..........

AmyAlbGDM7 FGVAVNPEFL REGSSVKDFF DPPKTVIGEL .......... ..........

AmyBalGDM FGVAVNPEFL REGTSVRDFF DPPKTVIGEI .......... ..........

PseDC3GMD5 FGVAVNPEFL RESTAIKDYD LPPMTVIGEF .......... ..........

PseVerGMD1 FGVAVNPEFL RESTAIADYD LPPMTVIGEF .......... ..........

PseQDAGMD1 FGVAVNPEFL RESTAIKDYD QPPMTVIGEL .......... ..........

PseAerGMD1 FGVGTNPEFL RESTAIKDYD FPPMTVIGEL .......... ..........

PseHYSGMD1 FGVAVNPEFL RESTAIQDYD FPAMTVIGEL .......... ..........

AmyAlbGMD6 FGVAVNPEFL REGSSVKDFF DPPKTVIGEL .......... ..........

AmyAzuGMD1 FGVAVNPEFL REGSSVKDFF DPPKTVIGEL .......... ..........

AlgSagHYPO FFYAHNPEFL REGSALDDFL HPSETLIGCN .......... ..........

AciKBSHYPO IRVVANPEFL REGSAIADFM EPSLLVAGSD .......... ..........

AmyBalGMD FGVAVNPEFL REGTSVRDFF DPPKTVIGEI .......... ..........

StrCanGMD1 VGVAVNPEFL REGTSVRDFF DPPKTVIGEL .......... ..........

PseAlcGMD1 FGVATNPEFL RESTAIKDYD FPAMTVIGEL .......... ..........

ActGloGMD6 FGVAVNPEFL REGSSVKDFF DPPKTVIGEL .......... ..........

OxaAB1GMD1 FGVCNNPEFL REGTAVYDYY HPPKTVIGES .......... ..........

PseAerUNPP FGVGTNPEFL RESTAIKDYD FPPMTVIGEL .......... ..........

HahGanHYPO FGLATNPEFL RESTAIQDYD YPPMTVIGEL .......... ..........

DesSalNSD FGVCNNPEFL REGSSVHDFY NPPKTVIGES .......... ..........

PseTomGMD1 FGVAVNPEFL RESTAIKDYD LPPMTVIGEF .......... ..........

MarAlgGMD FGVCVNPEFL RESTAIKDYD HPPMTVIGEL .......... ..........

PseSyrGMD3 FGVAVNPEFL RESTAIKDYD LPPMTVIGEF .......... ..........

NovAroGMD IELVYNPEFL REATAIEDYF HPPKIVLGTI .......... ..........

AzoSpGMD FHLCFQPEFL REGSSIRDYD KPPFTVVGAN .......... ..........

AzoVinGMD FGVAVNPEFL RESTAIKDYN FPPMTVIGEL .......... ..........

BacThuGMD IYLAHCPERV LPGRILIELV ENARI.VGGI .......... ..........

HahGanHYPP FGLATNPEFL RESTAIQDYD YPPMTVIGEL .......... ..........

AlgSagHYPP FFYAHNPEFL REGSALDDFL HPSETLIGCN .......... ..........

AciKBSHYPP IRVVANPEFL REGSAIADFM EPSLLVAGSD .......... ..........

PseSyrGMD2 FGVAVNPEFL RESTAIKDYD LPPMTVIGEF .......... ..........

PseSyrGMD1 FGVAVNPEFL RESTAIKDYD LPPMTVIGEF .......... ..........

StrCanGMD VGVAVNPEFL REGTSVRDFF DPPKTVIGEL .......... ..........

PseVerGMD FGVAVNPEFL RESTAIADYD LPPMTVIGEF .......... ..........

PseQDAGMD FGVAVNPEFL RESTAIKDYD QPPMTVIGEL .......... ..........

PseHYSGMD FGVAVNPEFL RESTAIQDYD FPAMTVIGEL .......... ..........

PseAlcGMD FGVATNPEFL RESTAIKDYD FPAMTVIGEL .......... ..........

PseAerUPP FGVGTNPEFL RESTAIKDYD FPPMTVIGEL .......... ..........

StaAurUMD IYLVHCPERV LPGKILEELV HNNRI.IGGV .......... ..........

EscColUMND VNIAYCPERV LPGQVMVELI KNDRV.IGGM .......... ..........

LusFleUGD IFLAHCPERV LPGNIVYEMV NNPRI.IGGI .......... ..........

SalEntUMD VNIAYCPERV LPGQVMVELI KNDRV.IGGM .......... ..........

ActSerUG IYIAYCPERV LPGRVMIELF ENDRV.IGGL .......... ..........

ErwTasUG IQVAYCPERV LPGQVMAELR KNDRV.IGGM .......... ..........

FusUlcUMD FMTVHCPERV LPGKILYELE HNDRI.IGAE .......... ..........

CorMarUMD IYFAHCPERI LPGNAMEELL TNDRI.IGGM .......... ..........

StaVitUMD IFLVHCPERV LPGKILHELI HNNRI.IGGI .......... ..........

SheVioUMD ILVAHCPERV LPGQVIRELV ENDRV.IGGM .......... ..........

SerS4UMD VNIAYCPERV LPGQVMVELI QNDRV.IGGM .......... ..........

PseStuUMD IRIAHCPERV LPGHVLRELV QNDRV.IGGM .......... ..........

PsePseUMD IRIAHCPERV LPGHVLRELI QNDRV.VGGM .......... ..........

PseFluUMD IRIAHCPERV LPGHVLRELV QNDRV.IGGM .......... ..........

HalSalUMD VHYAHCPERA SPGATLDEMV HNDRL.VGGV .......... ..........

FraPhiUMD IRVAHCPERI LPGQMIRELV ENDRI.IGGM .......... ..........

EdwC07UMD IRVAYCPERV LPGKIMVELL RNDRV.IGGM .......... ..........

AerAquUMD IFVAHCPERV LPGQVIRELV ENDRI.IGGM .......... ..........

GeoWSUUMD VFVAYSPERV DPGNKQFKTK NTPKV.VGGV .......... ..........

EscMG1UMD VNIAYCPERV LPGQVMVELI KNDRV.IGGM .......... ..........

DicZeaUMD IRIAYCPERV LPGRIMEELL SHERV.IGGM .......... ..........

VibOrdUMD ISIAHCPERV LPGHVVRELV ENDRV.IGGL .......... ..........

VidVulUMaD VNIAHCPERV LPGHVVRELV ENDRV.IGGL .......... ..........

KlePneUMaD VNIAYCPERV LPGQVMVELI KNDRV.IGGM .......... ..........

GeoTheUMaD VYLAHCPERV LPGRILIELV ENTRI.VGGV .......... ..........

BurXenUMaD IRIAHCPERV LPGHVIRELV QNDRV.IGGM .......... ..........

HasParUMaD VAVAYCPERV LPSNIWHELY HNDRV.IGGL .......... ..........

RalSolUMaD IRVAHCPERV LPGHVLRELV ENDRI.IGGM .......... ..........

PolIrgUMaD IYIAYCPERV LPGNVLFELE NNDRV.IGGI .......... ..........

RalSolNGD IRVAHCPERV LPGHVLRELV ENDRI.IGGM .......... ..........

YerEntUMaD INIAYCPERV LPGQVMVELI QNDRV.IGGM .......... ..........

EscFerUMaD VNIAYCPERV LPGQVMVELI KNDRV.IGGM .......... ..........

OceGraUMaD IRVAHCPERV LPGKVMQELI SNDRL.IGGL .......... ..........

BacNRRUMaD FFLAYSPERV DPGNFTFKTK NTPKV.VGGI .......... ..........

GeoKauUMaD VFVAYSPERV DPGNKQFKTK NTPKV.VGGV .......... ..........

PsyCNPUMaD ILVAYCPERV LPGQVIRELV ENDRV.IGGM .......... ..........

MorPE3UMaD VNVAHCPERV LPGHVVRELV ENDRV.IGGM .......... ..........

SerProUMaD VNIAYCPERV LPGQVMVELI QNDRV.IGGM .......... ..........

301 350

StrPyoUGD ...VKADAEK FALLLKSAAK KN........ ......NVPV LIMGASEAEA

HymAerUGD1 ...SERAEDV MSRLYKPFLL N......... ......GHPI IFMDIPSAEM

MetCapUGD1 ...NPRTTEL LRALYAPFNR S......... ......HDRM VCMDIRSAEL

ActGloUGD6 ...NPRSLEV LNHLYRPIIN QTFTAPTFLP RPDDIGAVPL VSTDLASAEL

HalZhaHYPO ...SEKAIKL MRECYAPYNR N......... ......REKI MIMDVRSAEL

MCIThaHYPO ...RTKFMNN TRKFFSRFNP .......... ......NTPI IITNHQTAEM

SalPacUGD ...STEAEET LRLLYAPITS GH........ ......GCPL VVTDRITAEM

NatGarNSD2 ...TETVLED VATLYESIVD .......... ......AGVH RAPSIEVAEA

BacSubUGD2 ...DHVSAVI VKSIYKHI.. .......... ......DTPF IVTSLAGAEL

AzoTolUGD ....SQRAHV FAELLKRGAL KK........ ......DIPT LFTDPTEAEA

LacCreEPS ...SREAAET FAGLLQEGAL KE........ ......DIDT LFMGFTEAEA

AlaProUG ....LTAGIA FADLLVEGAE KK........ ......DIET LFIRSTEAEA

StrZooUG ...VIQAAKA FAGLLKEGAK SK........ ......DTPV LFMGSQEAEA

StrHGBNSD ...DEDSLRA VGEFYGRLVD .......... .......VVV PVPGPREAEL

NatGarNSD ...DDEAAAT LRELYAPILE RA........ ......ETDL VETEIREAEL

TheMelUGD ....SERAKR FADLLLEGAI KK........ ......DVPI LLTDSTEAEA

SulDenUGD ....SQRAKI FAELLAQGAI KK........ ......DIPI LFTDSTEAEA

SphAlaUGD ....HPRAAD FARLLLEGSL KP........ ......DTAV LQTGNTEAEA

SheOneUGD ....SARAQT FATLLVNAAI KQ........ ......DIPI LLTDSTEAEA

SalSerUGD ....SERARE FAALLQEGAI KQ........ ......EIPT LFTDSTEAEA

PseHalUGD ....SERAAV FANLLKQGAI KQ........ ......DIAV LFTESTEAEA

ProMirUGD ....SARAEK FAELLQQGAL KK........ ......DIDV LFTDSTEAEA

ParDisUGD ...LGEAART FAALLQEGAM KK........ ......NIDT LFMGLTEAEA

ParDisUGD1 ...LDDAAHT FATLLQEGAI KE........ ......NIDT LFMGLTEAEA

LacSalUGD ...QLKASQQ FAELLQEGAI KE........ ......DIPV LFPGFSEAEA

CloPerUGD ...LVKAAHT FASLLQEGAI EE........ ......NIPT LFMGFTEAEA

ArcButUGD ....SKRAET FAKLLEQGAI KK........ ......DIPV LFTDSTEAEA

AerHydUGD ....SDRAQV FANLLQEGAI KQ........ ......NIPT LLTDSTEAEA

LacJohSBP ...LVEAAHT FASLLQEGAE DK........ ......DIAT LFMGFTEAEA

StrUbeUGD ...VIEAAKT FAQLLKDGSL DK........ ......DVPV LFMGSAEAEA

ReiMEDNSD ....SERAER FAALLQEGAV KK........ ......DVPT LFVDATEAEA

ExiSibNSD ....SERAQQ FADLLLEGAI KK........ ......DVPI LLTDSTEAEA

RumObeHYPO ...LVQASHN FAVLLQEGAL KE........ ......NIDT LFMGFTEAEA

RumObeHYP1 ...LVKAANT FAGLLQEGAI KE........ ......NIDT LIMGFTEAEA

FaePraHYPO ...LVKAANT FAGLLQEGAI KE........ ......NIDT LIMGFTEAEA

EubDolHYPO ...LLEVAHV FAELLQEGAI KE........ ......DIDT LFMGFTEAEA

BacPleHYPO V.NLKEKAQE FAALLQEGAL KP........ ......DVDT LFMGLKEAEA

BacCopHYPO E.QLEEKAHE FAALLQEGAI KP........ ......DVDT LFMGMKEAEA

StrTheESP ...LVKAAHT FAELLQEGAI KE........ ......NIDT LFMGFTEAEA

StrIniCPS ...LTQKANE FARLLQEGAI KE........ ......PIET LIMGFTEAEA

SerProUGD ...NKDVIEP IRELYEPFNR N......... ......HDRM IMMDIRSAEL

GeoMetUGD ...NVRTAEI MKELYSPFMR K......... ......TNRL IVMDVHSAEM

GeoUraUGD ...NVRTAEI MKELYAPFMR K......... ......TNRL IVMDIRSAEM

AnoFlaUGD ...DERAAAV VADIHRPF.. .......... ......GIPI FQTDIRSAEM

PorGinNSD ...SDRAREL ITSLYKPMLL N......... ......NFRV LFMDIASAEM

BacSelRPON ...SKWAEEL LTKLYEPFN. .......... .......LPI VSVNRRSAEM

PelPhaNSD ...NPRTREL LRFLYSPFNR S......... ......HERF IAMDVRSAEL

MetPopNSD ...DARAAAV MQEVYRPLYL N......... ......QAPI LFTGRRTAEL

MetExtNSD ...DNRAAAV MQEVYRPLYL N......... ......AAPI LLTGRRTAEL

VibVulPRE ....SERGQQ IAELFANAAE KR........ ......DVPI LLTNPTEAEA

GeoKauNSD ...SERAERI MKDLYAPFA. .......... .......LPY VVTDRKSAEM

RhiTriEXO ...DDRARET MTEVYRPLYL N......... ......QAPL VFTTRRTSEL

ParDisUGD2 ...LVDAAHI FAELLQEGAI KE........ ......NIDT LFMGLTEAEA

XanAxoUGD ....SARAET FARLLQQGAL KP........ ......DVPV LFTEPTEAEA

XanCamUGD ....SARAET FARLLQEGAL KP........ ......DVPV LFTEPTEAEA

EntFaeUGD ...LVEAAQT FAKLLQEGAI KE........ ......NVDT LFMGFTEAEA

StrPneUGD2 ...LKDKANI FASLLKEGAI KE........ ......DIDT LFMGVTEAEA

StrPneUGD3 ...LVSVAET FAGLLQEGAL KE........ ......DIDT LYMGFTEAEA

StrPneUG ...LVSAAET FARLLQEGAL KE........ ......NIDT LYMGFTEAEA

EscAlbUGD ....SERAER FAALLQEGAI KQ........ ......NIPT LFTDSTEAEA

BacTheUGD VLTLEKAAHA FAALLQEGAI KK........ ......DIPT LFMGIKEAEA

SacSpiUGD ...DPESLRV VRDFYDQLVD .......... ......VTV. PVPGTREAEL

Aer159PBP ...TPAVANF VDEVYRQIVT .......... ......VGTH KASSIRVAEA

ButSynUGD ...LEAKAMR FAGVIKEGAI EQ........ ......NVPV LYMGSTEAEA

BraJapUGD ...DERGRKV MGDIYRPLSL N......... ......QAPL MFTARRTAEM

SynCC9UGD ...DPASIDA LAAIYAHWVP .......... ......QEQI LRTNLWSSEL

StePneUGD ...LTKRAWQ FADLLKGGAI KE........ ......EVPI LVVAFNEAEV

SalAreUGD ...GPHSAIA AERFWRDALG V......... .......DVR RVPTAEAAEV

BurYI2NSD ...TPEVADF VDELYRGIIT .......... ......AGTH KASSIRVAEA

BacCerNSD ...SKWAEEV LMNLYKPFH. .......... .......LPI VSVNRRSAEM

PaeSanHYPO ...TSACADR TEELYQAAFH .......... ......KV.V RVSSTETAEM

Rhi42MUGD ...EPRAIEI MREVYRPLYL N......... ......EAPL YFCERRTSEL

OpiTAV5NSD ...GQQAVET LVSVYARWVP .......... ......RERI ITTNLWSSEL

MarHTCUGD8 ...GQAAKDA LSAVYLNWLP .......... ......KDRV LQTNVWSSEL

BacSubUGD ...DQTTAKT LEELFRPF.. .......... ......QIPI YQTDIRSAEM

NocKunUGD ...TDVCGER AAHFYSSFVH .......... .......TVV RARGTREAEM

DesHafUGD ...TPVCLEV AYTFYAQTIV .......... .......NVV PVSSPAAAEL

BacThuUGD ...NQKSTKK TVDFYKKFVK .......... .......GKI LETNARTAEM

SphJapUGD ...TPRCARK ALGFYRQFVR .......... .......GAC ITTTARAAEM

RicSibUGD ...NKESEEI LRKIYAPLQ. .......... ......GVKF VVTDLVTSEL

MetCapUGD ...NPRTTEL LRALYAPFNR S......... ......HDRM VCMDIRSAEL

HymAerUGD ...SERAEDV MSRLYKPFLL N......... ......GHPI IFMDIPSAEM

BacSubUGD7 ...DQTTAKT LEELFRPF.. .......... ......QIPI YQTDIRSAEM

ActGloUDG9 ...NPRSLEV LNHLYRPIIN QTFTAPTFLP RPDDIGAVPL VSTDLASAEL

NatGarNSD5 ...DDEAAAT LRELYAPILE RA........ ......ETDL VETEIREAEL

UncBacHYPO ...APSVGDT LEFIYKKTCD N......... ......EPKI SRMSLVSAEI

HalZhaHYPP ...SEKAIKL MRECYAPYNR N......... ......REKI MIMDVRSAEL

StrPneUGD1 ...LVSVAET FAGLLQEGAL KE........ ......DIDT LYMGFTEAEA

HoePhoUGD ...DPRAIEV VQDIYKPLDP D......... ......LHPL VITSRRTAEL

CapSpuUGD ...NEHAFKK MKELYSPFYM Q......... ......NERM IIMDIRSAEM

NovAroUGD ...DEWAQGV MKEVYRPLFL N......... ......RAPI LFTSRRSSEL

ProMarUGD ...DDEAIKA LNDIYLNWIP .......... ......QEKL IFTNLWSSEL

HomSapUGD ...GQRAVQA LCAVYEHWVP .......... ......REKI LTTNTWSSEL

MusMusUNA ...GQKAVRA LCAVYEHWVP .......... ......KEKI LTTNTWSSEL

DanRerUGD ...GQRAISA LCAVYEHWVP .......... ......KTRI ITTNTWSSEL

XenLaeUGD ...GQKAVRA LCDVYEHWVP .......... ......SEKI ITTNTWSSEL

RatNorUGD ...GQRAVQA LCAVYEHWVP .......... ......KEKI LTTNTWSSEL

PonAbeUGD ...GQRAVQA LCAVYEHWVP .......... ......REKI LTTNTWSSEL

BosTauUGD3 ...GQRAVQA LCAVYEHWVP .......... ......REKI LTTNTWSSEL

BamOldUGD ...GRKAVQA LKDVYAHWVP .......... ......EDRI LTTNLWSAEL

PedCorUGD ...GIIAIEA LCAIYEHWIP .......... ......RKNI ITTNTWSSEL

ZeaMayUGD8 ...GRKAVQA LKDVYAHWVP .......... ......EDRI LTTNLWSAEL

StrPurUGDP ...GLKAIDE LASIYVNWVP .......... ......KDRI IKTNTWSSEL

CioIntUGDP ...GNAAVDA LAEIYANWVD .......... ......PEKI IKTNTWSSEL

NemVecPPRE ...GLLSIDA LAWVYQHWIP .......... ......RDKI IKTNTWSSEL

CaeEleUGD ...GLQAVAE LVRIYENWVP .......... ......RNRI ITTNTWSSEL

PopTomUGD ...GQKAIQA LKDVYAHWVP .......... ......EDRI LTTNLWSAEL

DroMelUGD ...GHQAVEK LSWIYEHWIP .......... ......KQNI LTTNTWSSEL

AraThaUGD ...GFKAVQT LKNVYAHWVP .......... ......EGQI ITTNLWSAEL

DroMelUGD1 ...GHQAVEK LSWIYEHWIP .......... ......KQNI LTTNTWSSEL

NasVitUDG ...GQAAIEQ LCQVYEHWIP .......... ......RKNI ITTNTWSSEL

CaeBriHYPO ...GLQAVAE LVRIYENWVP .......... ......RERI ITTNTWSSEL

DroWilGK3 ...GHQAVEK LSWIYEHWIP .......... ......KKHI LTTNTWSSEL

NasVitUGDP ...GHQAVEK LSWIYEHWIP .......... ......KQNI LTTNTWSSEL

DroVirGJ3 ...GHQAVEK LSWIYEHWIP .......... ......KKNI LTTNTWSSEL

HomSapUGD2 ...GQRAVQA LCAVYEHWVP .......... ......REKI LTTNTWSSEL

XenTroUGD ...GQNAIAA LSEVYEHWV. P......... ......KSKI ITTNTWSSEL

FlaCF1UGD ...GETAIKA LVDVYANWVS .......... ......KDKI LTTNVWSSEL

ZeaMayUGD ...GRKAVQA LKDVYAHWVP .......... ......EDRI LTTNLWSAEL

MayZebUGD1 ...GQKAIRA LCAVYEHWVP .......... ......KERI ITTNTWSSEL

VitvinUGD1 ...GQKAIKA LRDVYAHWVP .......... ......VERI ICTNLWSAEL

HomSapUGD1 ...GQRAVQA LCAVYEHWVP .......... ......REKI LTTNTWSSEL

OviAriUGD1 ...GQRAVQA LCAVYEHWVP .......... ......REKI LTTNTWSSEL

FelCatUGD1 ...GQRAVQA LCAVYEHWVP .......... ......REKI LTTNTWSSEL

EquCabUGD1 ...GQRAVQA LCAVYEHWVP .......... ......REKI LTTNTWSSEL

CanFamUGD1 ...GQRAVQA LCAVYEHWVP .......... ......REKI LTTNTWSSEL

DroMojGI7 ...GHQAVEK LSWIYEHWIP .......... ......KKHI LTTNTWSSEL

DroPseGA34 ...GHEAVAK LSWIYEHWIP .......... ......KQHI LTTNTWSSEL

HomSapICRA ...GQRAVQA LCAVYEHWVP .......... ......REKI LTTNTWSSEL

ColLivUGD ...GQKAVRA LCAVYEHWVP .......... ......KEKI LTTNTWSSEL

CamFloUGD ...GQEAIEE LCKVYEHWIP .......... ......RENI LTTNTWSSEL

BosTauUGD ...GQRAVQA LCAVYEHWVP .......... ......REKI LTTNTWSSEL

MelGal1UGD ...GQKAVRA LCAVYEHWVP .......... ......KEKI LTTNTWSSEL

AilMelUGDP ...GQRAVQA LCAVYEHWVP .......... ......REKI LTTNTWSSEL

MusFurUGDP ...GQKAVQA LCAVYEHWVP .......... ......REKI LTTNTWSSEL

MusFur1UGD ...GQKAVQA LCAVYEHWVP .......... ......REKI LTTNTWSSEL

MelUnd1UGD ...GQKAVRA LCAVYEHWVP .......... ......KEKI LTTNTWSSEL

FalPer1UGD ...GQKAVRA LCAVYEHWVP .......... ......KEKI LTTNTWSSEL

ChrBel2UGD ...GQKAVRA LCAVYEHWVP .......... ......KEKI LTTNTWSSEL

FelCat1UGD ...GQRAVQA LCAVYEHWVP .......... ......REKI LTTNTWSSEL

SaiBolUGDP ...GQRAVQA LCAVYEHWVP .......... ......REKI LTTNTWSSEL

DroWilGK1 ...GHQAVEK LSWIYEHWIP .......... ......KKHI LTTNTWSSEL

CaeRemQV40 ...GLQAVAE LVRIYENWVP .......... ......RDRI ITTNTWSSEL

CaeBriQV40 ...GLQAVAE LVRIYENWVP .......... ......RERI ITTNTWSSEL

ZeaMayUGDI ...GRKAVQA LKDVYAHWVP .......... ......EDRI LTTNLWSAEL

GalGalUG ...GQKAVRA LCAVYEHWVP .......... ......KEKI LTTNTWSSEL

CaeEleUG ...GLQAVAE LVRIYENWVP .......... ......RNRI ITTNTWSSEL

PseAerGMD ...DKQTGDL LEEIYREL.. .......... ......DAPI IRKTVEVAEM

CanCloGM ...DNKAVDI MRELYSKV.. .......... ......NGEF FTVDIKTAEI

VibCraGMD ...TPKCSQR SVELYQTFVQ .......... .......GEC VITNARTAEM

PseSyrGMD ...DKASGDV LQSLYEEL.. .......... ......DAPI IRKDIAVAEM

AmyAzuGMD ...DPASGDV VAALYEGL.. .......... ......PGEV FRVAIPVAEM

OxaAB1GMD8 ...DEKAGAM LVELYAKM.. .......... ......EAPL VRTNVETAEM

ActGloGDM ...DSRSADA VAAMYVGL.. .......... ......PGDV FRVPIAVAEM

AmyAlbGDM7 ...DPASGDV VAALYEGL.. .......... ......PGEV FRVAIPVAEM

AmyBalGDM ...DTASGDA VAALYEGL.. .......... ......PGPV FRVPIPVAEM

PseDC3GMD5 ...DKASGDV LQSLYEEL.. .......... ......DAPI IRKDIAVAEM

PseVerGMD1 ...DKASGDV LQSLYEEL.. .......... ......DAPI IRKDIAVAEM

PseQDAGMD1 ...DSASGDI LQALYEEL.. .......... ......DAPV IRKPIEVAEM

PseAerGMD1 ...DKQTGDL LEEIYREL.. .......... ......DAPI IRKTVEVAEM

PseHYSGMD1 ...DTQSGDL LQSLYQGL.. .......... ......DAPV IRKSIEVAEM

AmyAlbGMD6 ...DPASGDV VAALYEGL.. .......... ......PGEV FRVAIPVAEM

AmyAzuGMD1 ...DPASGDV VAALYEGL.. .......... ......PGEV FRVAIPVAEM

AlgSagHYPO ...HSQAFER LSSLYQDI.. .......... ......DAPI TQTSITVSEM

AciKBSHYPO ...DAAAAEC VAALYRPL.. .......... ......NVPP CIVSLRAAEM

AmyBalGMD ...DTASGDA VAALYEGL.. .......... ......PGPV FRVPIPVAEM

StrCanGMD1 ...DPASGDV VAALYEGL.. .......... ......PGEV FRVPVPTAEA

PseAlcGMD1 ...DEQSGDL LQELYSEL.. .......... ......DAPI IRKSIEVAEM

ActGloGMD6 ...DSRSADA VAAMYVGL.. .......... ......PGDV FRVPIAVAEM

OxaAB1GMD1 ...DEKAGAM LVELYAKM.. .......... ......EAPL VRTNVETAEM

PseAerUNPP ...DKQTGDL LEEIYREL.. .......... ......DAPI IRKTVEVAEM

HahGanHYPO ...DKDAGDV LQELYKDL.. .......... ......PAPI IRKPVEVAEM

DesSalNSD ...DSKSGDI LASLYENI.. .......... ......DAPL IRTEMEVSEM

PseTomGMD1 ...DKASGDV LQSLYEEL.. .......... ......DAPI IRKDIAVAEM

MarAlgGMD ...DERSGEF LARIYQDL.. .......... ......DAPI IRKPIEVAEM

PseSyrGMD3 ...DKASGDV LQSLYEEL.. .......... ......DAPI IRKDIAVAEM

NovAroGMD ...DGAPSEK MVALHEGI.. .......... ......EAPI FHVGIREAEI

AzoSpGMD ...HAYPAER LRELFGHL.. .......... ......PCKF LETSVRAAEM

AzoVinGMD ...DKASGDA LASIYAEL.. .......... ......DAPI VRKDIAVAEM

BacThuGMD ...NEISANK AAEIYKSFVK .......... .......GNV ITTTAVTAEM

HahGanHYPP ...DKDAGDV LQELYKDL.. .......... ......PAPI IRKPVEVAEM

AlgSagHYPP ...HSQAFER LSSLYQDI.. .......... ......DAPI TQTSITVSEM

AciKBSHYPP ...DAAAAEC VAALYRPL.. .......... ......NVPP CIVSLRAAEM

PseSyrGMD2 ...DTASGDV LQSLYEEL.. .......... ......DAPI IRKDIAVAEM

PseSyrGMD1 ...DTASGDV LQSLYEEL.. .......... ......DAPI IRKDIAVAEM

StrCanGMD ...DPASGDV VAALYEGL.. .......... ......PGEV FRVPVPTAEA

PseVerGMD ...DKASGDV LQSLYEEL.. .......... ......DAPI IRKDIAVAEM

PseQDAGMD ...DSASGDI LQALYEEL.. .......... ......DAPV IRKPIEVAEM

PseHYSGMD ...DTQSGDL LQSLYQGL.. .......... ......DAPV IRKSIEVAEM

PseAlcGMD ...DEQSGDL LQELYSEL.. .......... ......DAPI IRKSIEVAEM

PseAerUPP ...DKQTGDL LEEIYREL.. .......... ......DAPI IRKTVEVAEM

StaAurUMD ...TKACIEA GKRVYRTFVQ .......... .......GEM IETDARTAEM

EscColUMND ...TPVCSAR ASELYKIFLE .......... .......GEC VVTNSRTAEM

LusFleUGD ...TERCTIE AVKVYQTFVK .......... .......GEL IRSKAGEAEL

SalEntUMD ...TPVCSAR ASALYKIFLE .......... .......GEC VVTNSRTAEM

ActSerUG ...TAESTRQ AVDLYRIFVK .......... .......GEC IATDARTAEM

ErwTasUG ...TDTCSQR ASELYRLFVE .......... .......GEC VVTNSRTAEM

FusUlcUMD ...RKEAAEY TKVIYEAMVK .......... ......EGTC YITDDITAEM

CorMarUMD ...SEEATRR ATKIYASFCT .......... .......GEL LGTNARTAEL

StaVitUMD ...TPACVEA GKEVYGTFVQ .......... .......GEM IETNAKTAEM

SheVioUMD ...DQASTDA ATAVYKMFVH .......... ......KGNC IATNARTAEM

SerS4UMD ...TPKCSER ASALYKIFLE .......... .......GEC VITNSRTAEM

PseStuUMD ...TAKCSEA AVTLYKTFVE .......... .......GEC VITNARTAEM

PsePseUMD ...TARCSAA ACALYKIFVE .......... .......GEC VVTNARTAEM

PseFluUMD ...TPKCSAA AASLYQIFVE .......... .......GEC VITNARTAEM

HalSalUMD ...DDASRAR TVDLYDFAE. .......... .......GKI HRTNPTTAEF

FraPhiUMD ...TPKCSEY AATLYKMVVQ .......... .......GEC SITTARTAEM

EdwC07UMD ...TPTCSAQ ASALYKLFLE .......... .......GEC VETNARTAEM

AerAquUMD ...DKASTAA ATQVYKMFVK .......... ......KGEC IATEARTAEM

GeoWSUUMD ...TKTCTKV AAAMYRAVLE .......... ......GDVH EVSSPAVAEM

EscMG1UMD ...TPVCSAR ASELYKIFLE .......... .......GEC VVTNSRTAEM

DicZeaUMD ...TPRCSAR ASELYRLFLE .......... .......GEC VMTDSRTAEM

VibOrdUMD ...TPRCSQR SVELYQTFVQ .......... .......GEC VITNARTAEM

VidVulUMaD ...TPKCSAR AVELYKTFVQ .......... .......GEC VITNARTAEM

KlePneUMaD ...SPVCSAR ASELYKIFLE .......... .......GEC VMTNSRTAEM

GeoTheUMaD ...TKEAAKQ AADVYRAIVK .......... .......GEV IETEAVTAEM

BurXenUMaD ...TRRCGEL ARELYQIFVQ .......... .......GDC ILTDARTAEM

HasParUMaD ...TPNCTQQ AVALYQLFAK .......... .......GEC ITTDSRLAEM

RalSolUMaD ...TPKCSEA AQRLYELFVR .......... .......GRC IVTDARTAEM

PolIrgUMaD ...NDASSEK AILFYKQFVN .......... .......GDL HKTNARTAEM

RalSolNGD ...TPRCSQA AQRLYELFVR .......... .......GRC IVTDARTAEM

YerEntUMaD ...TPKCSAR ASELYKIFLE .......... .......GEC VVTNSRTAEM

EscFerUMaD ...TPVCSAR ASELYKIFLE .......... .......GEC VVTNSRTAEM

OceGraUMaD ...TPKCSEL AAQLYKLFVT .......... .......GDC VLTNARTAEM

BacNRRUMaD ...TPKCTDV AAALYESILE .......... ......APIH RVSSPAVAEM

GeoKauUMaD ...TKTCTKV AAAMYRAVLE .......... ......GDVH EVSSPAVAEM

PsyCNPUMaD ...DEASTKA ATLVYKSFVH .......... ......KGNC IATTARTAEM

MorPE3UMaD ...SKRCSER SVELYKIFVQ .......... .......GDC VITNARTAEM

SerProUMaD ...TPKCSER ASALYKIFLE .......... .......GEC VITNSRTAEM

351 400

StrPyoUGD VKLFANTYLA LRVAYFNELD TYAESR.KLN SHMIIQGISY DDRIG..MHY

HymAerUGD1 TKYAANSMLA TKISFMNDIA NLCEIM.GAD VNMVRRGIGS DARIG..PKF

MetCapUGD1 TKYAANAMLA TKISFMNELA NLAERL.GAD IEKVRVGIGS DPRIG..YHF

ActGloUGD6 IKYAANAFLA LKISFVNEIG QLAGKV.GAD ITEVARGIGL DQRIG..SRF

HalZhaHYPO TKYAANAMLA TKISFMNEMA NLAERL.GAD IEQVRQGIGS DPRIG..YHF

MCIThaHYPO IKYANNSFLA TKISFINQLA SICQKIPDTN IDDIAQTIGL DPRIG..NLF

SalPacUGD VKATANAYLA TRISFVNAVA EMCAVV.GAN VAELADAIGH DARIG..HQY

NatGarNSD2 CKVVENAQRD LNIAFVNELT MALDTM.DID GRAVLEAAGT KWN....FHE

BacSubUGD2 IKYANNFFLA AKISFINEMA RICEAY.QSD ISDISRAIGL DPRIG..KHF

AzoTolUGD IKLFANTYLA MRVAFFNELD TYAASH.GLD TRQIIEGAEL DPRIG..KGY

LacCreEPS VKLFANTYLA LRVSYFNELD TYAEMK.GLD TEAIIKGVGL DPRIG..SHY

AlaProUG VKLYANTYLA MRVSFFNELD SYALAH.ELD AKGIINGVCL DERIG..KGY

StrZooUG VKLFANTFLA MRVSYFNELD TYSESK.GLD AQRVIEGVCH DQRIG..NHY

StrHGBNSD AKLLENTFRH VNVALVNELA MYCRAA.GVD VWSVLDAAGT KPF.G..FMR

NatGarNSD IKYANNAFLA SKVSLVNELG NIAREY.GAD AYEVLEAVGL DERI..SERF

TheMelUGD IKLFANTYLA MRVAFFNELD TYCEIK.GLN TRQVIEGVCL DPRIG..MYY

SulDenUGD IKLFANTYLA MRVAYFNELD SYAQSH.GLD TKQIIEGVGL DPRIG..THY

SphAlaUGD IKLFANTYLA MRVAFFNELD TYAALH.GVD SRQVIEGVCL DPRIG..SFY

SheOneUGD IKLFANTFLA MRVAYFNELD TYAESR.GLN TRQIIEGVCL DPRIG..DYY

SalSerUGD IKLFANTYLA MRVAYFNELD SYAETL.GLN TRQIIEGVCL DPRIG..NHY

PseHalUGD IKLFSNTYLA MRVAYFNELD TYAEAH.GLN SKQIIQGVGL DPRIG..NHY

ProMirUGD IKLFANTYLA LRVAYFNELD SYAQAY.NLN ARQIIEGVCL DPRIG..NHY

ParDisUGD VKLFANTYLA LRVSYFNELD TYAEMK.GLD TQAIINGVSL DPRIG..THY

ParDisUGD1 VKLFANTYLA LRVSYFNELD TYAEMK.GLD TKAIIDGISL DPRIG..THY

LacSalUGD IKLFANTYLA LRISFFNELD TYAESR.NLN TQDIIKGVSL DERIG..DHY

CloPerUGD VKLFANTYLA LRVSYFNELD TYAEMK.RLD TKAIIDGVCL DPRIG..THY

ArcButUGD IKLFSNTYLA MRVAYFNELD SYASAH.NLD TKQIIDGVGL DPRIG..THY

AerHydUGD IKLFANTYLA MRVAFFNELD TYAQSH.GLD SRQIIDGVGL DPRIG..EHY

LacJohSBP VKLFANTYLA LRVSYFNELD TYAESK.GLN TQEIIDGVGL DPRIG..SHY

StrUbeUGD VKLFANTYLA MRVSYFNELD TYAEKN.GLR VDNIIEGVCH DRRIG..IHY

ReiMEDNSD IKLFANTYLA MRVAYFNELD TYAETH.DLN TRQIIEGVGL DPRIG..NHY

ExiSibNSD IKLFSNTYLA MRVAFFNELD TYAEVR.NLD TKQIIEGVGL DSRIG..NHY

RumObeHYPO VKLFANTYLA LRVSYFNELD TYAEMK.GLN TQNIIQGVCL DPRIG..THY

RumObeHYP1 VKLFANTYLA LRVSYFNELD TYAEMK.GLN TQQIINGVCL DPRIG..SHY

FaePraHYPO VKLFANTYLA LRVSYFNELD TYAEMK.GLN TQQIIKGVCL DPRIG..DQY

EubDolHYPO VKLFANTYLA LRVSYFNELD TYAEMK.GLD TQQIINGVCL DPRIG..THY

BacPleHYPO VKLFANTYLA LRVSYFNELD TYAEVK.GLD TQAIIQGICL DPRIG..THY

BacCopHYPO VKLFANTYLA LRVSYFNELD TYAEVK.GLD TQAIIQGICL DPRIG..SHY

StrTheESP VKLFANTYLA LRVSYFNELD TYAEMK.GLN TQQIINGVCL DPRID..THY

StrIniCPS VKLFSNTYLA LRVSYFNELD TYAETK.GLD TKAIIDGVGL DPRIG..DHY

SerProUGD TKYAANCMLA TKISFMNEMS NLAEML.GAD IEKVRQGIGS DSRIG..YHF

GeoMetUGD TKYAANAMLA TRISFMNQIA NLCERM.GAD VSAVREGIGS DSRIG..YDF

GeoUraUGD TKYAANAMLA TKISFMNQIA NLCERM.GAD VSAVREGIGS DSRIG..YDF

AnoFlaUGD IKYASNAFLA TKISFINEIA NICEKV.GAD VEQVAAGMGM DQRIG..SAF

PorGinNSD TKYAANAMLA TRISFMNDVA NLCERV.GAD VSMVRLGIGS DSRIG..SKF

BacSelRPON IKYASNDYLA LKISYMNDIA NLCELV.GAD VQDVAKGMSY DARIG..SKF

PelPhaNSD TKYAANAMLA TKISFMNEIA NIAERV.GAD VEAVRKGIGS DSRIG..FSF

MetPopNSD TKYAANAFLA TKITFINEIA DLCEQV.GAN VQEVARGIGL DNRIG..SKF

MetExtNSD TKYAANAFLA TKITFINEIA DLCEQV.GAN VQEVARGIGL DNRIG..GKF

VibVulPRE IKLFSNTYLA MRIAYFNELD SYAEAH.SLG ARQIIEGVGL DPRIG..NHY

GeoKauNSD IKYAANVFLA LKISYINEIA NVCELV.GAD IQAVAEGIGM DPRIG..RRF

RhiTriEXO IKYAANAFLA MKITFINEIA DLCERV.DAN VQDVSRGIGL DGRIG..SKF

ParDisUGD2 VKLFANTYLA LRVSYFNELD TYAEMK.GLD TQSIINGVCL DPRIG..NHY

XanAxoUGD IKLFANTYLA MRVSYFNELD TYALTH.GLD SRQIIEGICL DPRIG..SHY

XanCamUGD IKLFANTYLA MRVSYFNELD TYALTH.GLD SRQIIEGICL DPRIG..SHY

EntFaeUGD VKLFANTYLA LRVSYFNELD TYAEMK.GLN TQQIINGVCR DPRIG..THY

StrPneUGD2 VKLFANTYLA LRVSYFNELD TYAEVK.GLD AKAIIDGIGL DPRIG..NHY

StrPneUGD3 VKLFANTYLA LRVSYFNELD TYSEMK.GLD TQAIIDGVGL DPRIG..SHY

StrPneUG VKLFANTYLA LRVSYFNELD TYSEMK.GLD TQAIIDGVGL DPRIG..SHY

EscAlbUGD IKLFANTYLA MRVAYFNELD SYAESL.GLN TRQIIEGVCL DPRIG..NHY

BacTheUGD VKLFANTYLA LRVSYFNELD TYAEMK.GLD SQSIIQGVGL DPRIG..THY

SacSpiUGD TKLLENTFRH INIALVNELA VFAHGL.GVD MWSVLDAAAS KPFG...FMR

Aer159PBP AKVIENTQRD LNIAVINEFA KIFNRL.DID TEEVLKAAGT KW.N...FLP

ButSynUGD VKLFANTYLA LRVSYFNELD TYAELK.ELN TRQIIEGVCL DPRIG..DHY

BraJapUGD IKYAANAFLA TKITFINEIA DLSEKV.GAN VQEVARGIGL DNRIG..TKF

SynCC9UGD SKLTANAFLA QRISSINSIA AFCEAS.GAD VREVARAIGT DSRIG..PKF

StePneUGD AKLFSNTYLA TRVAYFNEID TYSEVK.GLN PKTIIDIVCY DPRIG..SYY

SalAreUGD VKLATNWWID VNVAIANELA RYCAVL.DVD VLDVIDAANT LPKGTSRVNL

BurYI2NSD AKVIENTQRD VNIALINELS IIFNKM.NID TESVLLAAGT KW..N..FIP

BacCerNSD IKYASNDFLA LKISYMNDIA NLCELV.GAD IQDVARGMSF DERIG..SKF

PaeSanHYPO AKLLENTFRF VNISFMNEFA MLCDRL.GIN VWEVVEAAST KPF.G..FTA

Rhi42MUGD IKYAANAFLA MKITFINEIA DLCEQI.GAD VQKVAKGIGM DKRIG..DKF

OpiTAV5NSD SKLVANAFLA QRISSINAIS ALCEAT.GAN VDEVAHAIGR DSRIG..PKF

MarHTCUGD8 SKLVANAFLA QRVSSINSIS ALCEKT.DAN IAEVARAIGY DSRIG..SKF

BacSubUGD IKYASNAFLA TKISFINEIS NICEKV.GAD IEAVAYGMGQ DKRIG..SQF

NocKunUGD AKLLENTYRH VNIALVNEMA VFCQEL.GID LWDSISAAAT KPFG...FQA

DesHafUGD TKVFENTYRA VNIALVNELM LLCDRM.GID IWEVVEAAGT KPFG...IQT

BacThuUGD AKLTENSFRD VNIAFANELS IICDEL.HIN VWELISLANR HPR....VNI

SphJapUGD VKLVENSFRD VNIAFANELS VIAENM.DID VWEVIRLANR HPR....VNI

RicSibUGD IKYASNSFLA TKIAFINEMA DLCEKI.GGN IKDLSKGVGL DQRIG..QNF

MetCapUGD TKYAANAMLA TKISFMNELA NLAERL.GAD IEKVRVGIGS DPRIG..YHF

HymAerUGD TKYAANSMLA TKISFMNDIA NLCEIM.GAD VNMVRRGIGS DARIG..PKF

BacSubUGD7 IKYASNAFLA TKISFINEIS NICEKV.GAD IEAVAYGMGQ DKRIG..SQF

ActGloUDG9 IKYAANAFLA LKISFVNEIG QLAGKV.GAD ITEVARGIGL DQRIG..SRF

NatGarNSD5 IKYANNAFLA SKVSLVNELG NIAREY.GAD AYEVLEAVGL DERI..SERF

UncBacHYPO AKLSLNAYIT MKISFVNSLS NICQAIPNAN IDNVTEALGA DRRIA..PYY

HalZhaHYPP TKYAANAMLA TKISFMNEMA NLAERL.GAD IEQVRQGIGS DPRIG..YHF

StrPneUGD1 VKLFANTYLA LRVSYFNELD TYSEMK.GLD TQAIIDGVGL DPRIG..SHY

HoePhoUGD TKYAANAFLA VKLAYINEIA DLCEQV.GAD VQHVSLGIGL DSRIG..KKF

CapSpuUGD TKYAANTMLA TKISFMNEIA NICERV.GAD VNKVRIGIGS DSRIG..YSF

NovAroUGD IKYAANAFLA TKITFINEMA DLCEKV.GAD VQDVSRGIGL DNRIG..AKF

ProMarUGD SKLTANAFLA QRISSINSIS AICEVT.GAD IKDVSFAIGK DNRIG..SKF

HomSapUGD SKLAANAFLA QRISSINSIS ALCEAT.GAD VEEVATAIGM DQRIG..NKF

MusMusUNA SKLAANAFLA QRISSINSIS ALCEAT.GAD VEEVATAIGM DQRIG..NKF

DanRerUGD SKLAANAFLA QRISSINSIS ALCEST.GAD VEEVARAIGM DQRIG..SKF

XenLaeUGD SKLAANAFLA QRISSINSIS ALCEAT.GAD VEEVARAIGM DQRIG..NKF

RatNorUGD SKLAANAFLA QRISSINSIS ALCEST.GAD VEEVATAIGM DQRIG..NKF

PonAbeUGD SKLAANAFLA QRISSINSIS ALCEAT.GAD VEEVATAIGM DQRIG..NKF

BosTauUGD3 SKLTANAFLA QRISSINSIS ALCEAT.GAD VEEVATAIGM DQRIG..NKF

BamOldUGD SKLAANAFLA QRISSVNAIS ALCEAT.GAN VAEVAYAVGK DSRIG..PRF

PedCorUGD SKLAANAFLA QRISSINSLS AVCEVT.GGD VSEVAAAIGM DSRIG..SKF

ZeaMayUGD8 SKLAANAFLA QRISSVNAIS ALCEAT.GAN VTEVAYAVGK DTRIG..PKF

StrPurUGDP SKLAANAFLA QRISSINSIS AVCEAT.GAD VSEVAHAIGM DSRLG..PKF

CioIntUGDP SKLAANAFLA QRISSINSMS AICEAT.GAD VGEVANAVGK DSRIG..NKF

NemVecPPRE SKLAANAFLA QRISSINSMS AICEAT.GAD VSEVAHAIGM DSRIG..SQF

CaeEleUGD SKLVANAFLA QRISSINSIS AVCEAT.GAE ISEVAHAVGY DTRIG..SKF

PopTomUGD SKLAANAFLA QRISSVNAMS ALCEAT.GAD VAEVSYAVGK DSRIG..PKF

DroMelUGD SKLAANAFLA QRISSINSLS AVCEAT.GAD VSEVARAVGL DSRIG..SKF

AraThaUGD SKLAANAFLA QRISSVNAMS ALCEAT.GAD VTQVSYAVGT DSRIG..PKF

DroMelUGD1 SKLAANAFLA QRISSINSLS AVCEAT.GAD VSEVARAVGL DSRIG..SKF

NasVitUDG SKLAANAILA QRISSINSLS AVCEAT.GAD VSEVARAVGL DSRIG..PKF

CaeBriHYPO SKLVANAFLA QRISSINSIS AVCEAT.GAE ISEVAHAVGF DTRIG..NKF

DroWilGK3 SKLAANAFLA QRISSINSLS AVCEAT.GAD VSEVARAVGL DSRIG..SKF

NasVitUGDP SKLAANAFLA QRISSINSLS AVCEAT.GAD VSEVARAVGL DSRIG..SKF

DroVirGJ3 SKLAANAFLA QRISSINSLS AVCEAT.GAD VSEVARAVGL DSRIG..SKF

HomSapUGD2 SKLVTNAFLA QRISSINSIS ALCEAT.GAD VEEVATAIGM DQRIG..NKF

XenTroUGD SKLAANAFLA QRISSINSIS ALCEVT.GAD VGEVAHAIGT DQRIG..RPF

FlaCF1UGD SKLTANAFLA QRISSINAMS ELCEKT.GAD VNEVARAIGM DSRIG..SKF

ZeaMayUGD SKLAANAFLA QRISSVNAIS ALCEAT.GAN VSEVAYAVGK DTRIG..PKF

MayZebUGD1 SKLAANAFLA QRISSINSIS ALCEAT.GAD VEEVAKAIGM DQRIG..NKF

VitvinUGD1 SKLAANAFLA QRISSVNAMS ALCEAT.GAD VTEVSHAVGK DTRIG..PKF

HomSapUGD1 SKLAANAFLA QRISSINSIS ALCEAT.GAD VEEVATAIGM DQRIG..NKF

OviAriUGD1 SKLTANAFLA QRISSINSIS ALCEAT.GAD VEEVATAIGM DQRIG..SKF

FelCatUGD1 SKLAANAFLA QRISSINSIS ALCEAT.GAD VEEVATAIGM DQRIG..NKF

EquCabUGD1 SKLAANAFLA QRISSINSIS ALCEAT.GAD VEEVATAIGM DQRIG..NKF

CanFamUGD1 SKLAANAFLA QRISSINSIS ALCEAT.GAD VEEVATAIGM DQRIG..NKF

DroMojGI7 SKLAANAFLA QRISSINSLS AVCEST.GAD VSEVARAVGL DSRIG..SKF

DroPseGA34 SKLAANAFLA QRISSINSLS AVCEAT.GAD VSEVARAVGL DSRIG..SKF

HomSapICRA SKLVTNAFLA QRISSINSIS ALCEAT.GAD VEEVATAIGM DQRIG..NKF

ColLivUGD SKLAANAFLA QRISSINSIS ALCEAT.GAD VEEVARAIGT DQRIG..NKF

CamFloUGD SKLAANAFLA QRISSINSLS AVCEAT.GAD VSEVARAVGL DSRIG..SKF

BosTauUGD SKLTANAFLA QRISSINSIS ALCEAT.GAD VEEVATAIGM DQRIG..NKF

MelGal1UGD SKLAANAFLA QRISSINSIS ALCEAT.GAD VEEVARAIGT DQRIG..NKF

AilMelUGDP SKLAANAFLA QRISSINSIS ALCEAT.GAD VEEVATAIGM DQRIG..NKF

MusFurUGDP SKLAANAFLA QRISSINSIS ALCEAT.GAD VEEVATAIGM DQRIG..NKF

MusFur1UGD SKLAANAFLA QRISSINSIS ALCEAT.GAD VEEVATAIGM DQRIG..NKF

MelUnd1UGD SKLAANAFLA QRISSINSIS ALCEAT.GAD VEEVARAIGT DQRIG..NKF

FalPer1UGD SKLAANAFLA QRISSINSIS ALCEAT.GAD VEEVARAIGT DQRIG..NKF

ChrBel2UGD SKLAANAFLA QRISSINSIS ALCEAT.GAD VEEVARAIGM DQRIG..NKF

FelCat1UGD SKLAANAFLA QRISSINSIS ALCEAT.GAD VEEVATAIGM DQRIG..NKF

SaiBolUGDP SKLAANAFLA QRISSINSIS ALCEAT.GAD VEEVATAIGM DQRIG..NKF

DroWilGK1 SKLAANAFLA QRISSINSLS AVCEAT.GAD VSEVARAVGL DSRIG..SKF

CaeRemQV40 SKLVANAFLA QRISSINSIS AVCEAT.GAE ISEVAHAVGF DTRIG..SKF

CaeBriQV40 SKLVANAFLA QRISSINSIS AVCEAT.GAE ISEVAHAVGF DTRIG..NKF

ZeaMayUGDI SKLAANAFLA QRISSVNAIS ALCEAT.GAN VTEVAYAVGK DTRIG..PKF

GalGalUG SKLAANAFLA QRISSINSIS ALCEAT.GAD VEEVARAIGT DQRIG..NKF

CaeEleUG SKLVANAFLA QRISSINSIS AVCEAT.GAE ISEVAHAVGY DTRIG..SKF

PseAerGMD IKYTCNVWHA AKVTFANEIG NIAKAV.GVD GREVMDVICQ DHKLNLSRYY

CanCloGM IKYVNNSYHA LKVTFANEIG RICKTL.NID SFKVMQLFCL DTRLNISPYY

VibCraGMD AKLTENSSRD VQIAFANELS VICDKL.DIN VWELIALANR HPR....VNI

PseSyrGMD IKYTCNVWHA TKVTFANEIG NIAKAV.GVD GREVMDVVCQ DKALNLSQYY

AmyAzuGMD TKYADNSFHG LKIGFANELG AICRAL.GLD SHQVIDVFLA DRKLNISPAY

OxaAB1GMD8 VKYTDNTWHA VKVAFANEIG NICKAV.GID GHKVMEIFCQ DTKLNLSSYY

ActGloGDM AKYADNCFHG LKIAFANELG AISRAL.GVD SHKMIEVFLA DTKLNISPAY

AmyAlbGDM7 TKYADNSFHG LKIGFANELG AICRAL.GLD SHQVIDVFLS DRKLNISPAY

AmyBalGDM TKYADNSFHG LKIGFANELG SVCRAL.GLD SHQVIDVFLA DTKLNISPAY

PseDC3GMD5 IKYTCNVWHA TKVTFANEIG NIAKAV.GVD GREVMDVVCQ DKALNLSQYY

PseVerGMD1 IKYTCNVWHA TKVTFANEIG NIAKAV.GVD GREVMEVVCQ DKTLNLSQYY

PseQDAGMD1 IKYTCNVWHA TKVTFANEIG NIAKAV.GVD GREVMDVVCQ DTVLNLSQYY

PseAerGMD1 IKYTCNVWHA AKVTFANEIG NIAKAV.GVD GREVMDVICQ DHKLNLSRYY

PseHYSGMD1 IKYTCNVWHA TKVSFANEIG NIAKAS.GVD GREVMDVVCQ DYKLNLSRYY

AmyAlbGMD6 TKYADNSFHG LKIGFANELG AICRAL.GLD SHQVIDVFLS DRKLNISPAY

AmyAzuGMD1 TKYADNSFHG LKIGFANELG AICRAL.GLD SHQVIDVFLA DRKLNISPAY

AlgSagHYPO VKYVDNSWHA LKVSFANEIG RVCKSL.GID GHEVMEIFCH DRKLNLSEYY

AciKBSHYPO IKYACNAFHA MKIAFANEIG TVCESL.EMD AAEVMSALCE DRKLNISPAY

AmyBalGMD TKYADNSFHG LKIGFANELG SVCRAL.GLD SHQVIDVFLA DTKLNISPAY

StrCanGMD1 IKYADNAFHG LKIGFANELG AVCQAL.GVD SHQVMDVFLA DRKLNISPAY

PseAlcGMD1 IKYTCNVWHA AKVTFANEIG NIAKAA.GVD GREVMDVVCQ DHKLNLSKYY

ActGloGMD6 AKYADNCFHG LKIAFANELG AISRAL.GVD SHKMIEVFLA DTKLNISPAY

OxaAB1GMD1 VKYTDNTWHA VKVAFANEIG NICKAV.GID GHKVMEIFCQ DTKLNLSSYY

PseAerUNPP IKYTCNVWHA AKVTFANEIG NIAKAV.GVD GREVMDVICQ DHKLNLSRYY

HahGanHYPO IKYTCNVWHA TKVTFANEIG NIAKAL.GVD GRDVMDVVCK DSKLNISKYY

DesSalNSD VKYADNNWHA VKVAFANEIG AICKEV.GID SHKVMNIFCK DTKLNISSYY

PseTomGMD1 IKYTCNVWHA TKVTFANEIG NIAKAV.GVD GREVMDVVCQ DKALNLSQYY

MarAlgGMD IKYTCNIWHA TKVSFANEIG NIAKSM.GVD GRDVMDVVCQ DKKLNISRYY

PseSyrGMD3 IKYTCNVWHA TKVTFANEIG NIAKAV.GVD GREVMDVVCQ DKALNLSQYY

NovAroGMD TKFVDNTWHA VKVAFANEVG RVCQNL.GIS AKQVHEIFVS DTKLNISAYY

AzoSpGMD MKYCCNNFHA LKITFANETA RLCSAL.GVN PFEVMELVCQ DTQLNISRAY

AzoVinGMD IKYTCNVWHA TKVTFANEIG NIAKAA.GVD GREVMEVVCM DNKLNLSQYY

BacThuGMD AKLMENTFRD VNIALANELA KISANL.GIN ALDVISLANL HPR....VNL

HahGanHYPP IKYTCNVWHA TKVTFANEIG NIAKAL.GVD GRDVMDVVCK DSKLNISKYY

AlgSagHYPP VKYVDNSWHA LKVSFANEIG RVCKSL.GID GHEVMEIFCH DRKLNLSEYY

AciKBSHYPP IKYACNAFHA MKIAFANEIG TVCESL.EMD AAEVMSALCE DRKLNISPAY

PseSyrGMD2 IKYTCNVWHA TKVTFANEIG NIAKAV.GVD GREVMDVVCQ DKALNLSQYY

PseSyrGMD1 IKYTCNVWHA TKVTFANEIG NIAKAV.GVD GREVMDVVCQ DKALNLSQYY

StrCanGMD IKYADNAFHG LKIGFANELG AVCQAL.GVD SHQVMDVFLA DRKLNISPAY

PseVerGMD IKYTCNVWHA TKVTFANEIG NIAKAV.GVD GREVMEVVCQ DKTLNLSQYY

PseQDAGMD IKYTCNVWHA TKVTFANEIG NIAKAV.GVD GREVMDVVCQ DTVLNLSQYY

PseHYSGMD IKYTCNVWHA TKVSFANEIG NIAKAS.GVD GREVMDVVCQ DYKLNLSRYY

PseAlcGMD IKYTCNVWHA AKVTFANEIG NIAKAA.GVD GREVMDVVCQ DHKLNLSKYY

PseAerUPP IKYTCNVWHA AKVTFANEIG NIAKAV.GVD GREVMDVICQ DHKLNLSRYY

StaAurUMD SKLMENTYRD VNIALANELT KICNNL.NIN VLDVIEMANK HPR....VNI

EscColUMND CKLTENSFRD VNIAFANELS LICADQ.GIN VWELIRLANR HPR....VNI

LusFleUGD SKLMENTFRD VNIALANELV KVSEKL.NID ALKVIEMANL HPR....VNL

SalEntUMD CKLTENSFRD VNIAFANELS LICAEQ.GIN VWELIRLANR HPR....VNI

ActSerUG CKLTENSFRD VNLAFANELS MICDKL.NIN VWELIRLANR HPR....VNI

ErwTasUG CKLTENSFRD VNIAFANELS LICTQQ.GIN VWELIRLANR HPR....VNI

FusUlcUMD CKLVENTFRD VNIAFANELS VICDKL.NIN VFELIKLANK HPR....VNI

CorMarUMD AKLTENSFRD VNIAFANELS LICDKI.GID VWELIDLANH HPR....VNI

StaVitUMD SKLMENTYRD LNIALANELA MVCNQL.DIN VLEVIKMANK HPR....VNI

SheVioUMD AKLTENASRD VQIAFANELS MICDKQ.GID VWELIELANL HPR....VNI

SerS4UMD CKLTENSFRD VNIAFANELS LICADQ.GIN VWELIRLANR HPR....VNI

PseStuUMD CKLTENSFRD VNIAFANELS IICDKF.GVD VWELIKLANR HPR....VNI

PsePseUMD AKLTENSFRD VNIAFANELS IICDKI.GIS VWELIRLANR HPR....VNI

PseFluUMD CKLTENSFRD TNIAFANELS MICDKL.DIN VWELIRLANR HPR....VNI

HalSalUMD VKLMENTYRD VNIALANEFA MLGEEY.AID TRRAIELANE HPR....VDV

FraPhiUMD CKLTENSFRD INIAFANELS MLCDKM.GIN VWELITMANK HPR....VNV

EdwC07UMD CKLTENSFRD VNIAFANELS LICDAQ.GID VWQLIALANR HPR....VNI

AerAquUMD SKLTENAFRD VNIAFANELS LICDQQ.GIN VWELISLANR HPR....VNI

GeoWSUUMD EKIFENTFRH INIALANEMA ILCERM.GID VWEVIDAAKT KPYG...FMA

EscMG1UMD CKLTENSFRD VNIAFANELS LICADQ.GIN VWELIRLANR HPR....VNI

DicZeaUMD CKLTENSFRD VNIAFANELS RICAEQ.QIN VWELIRLANR HPR....VTI

VibOrdUMD AKLTENSSRD VQIAFANELS VICDKL.DIN VWELITLANR HPR....VNI

VidVulUMaD AKLTENSSRD VQIAFANELS IICDKL.DIN VWELIALANR HPR....VNI

KlePneUMaD CKLTENSFRD VNIAFANELS LICADQ.GIN VWELIRLANR HPR....VNI

GeoTheUMaD AKLMENTFRD VNIALANELV KIAQRV.GVN AHKVIELANK HPR....VNI

BurXenUMaD CKLSENAFRD INIAYANELS MICDRL.DIN VWELIRLANR HPR....VSI

HasParUMaD CKLTENSFRD VNIAFANELS MICDQL.NVN VWELIRIANR HPR....VSI

RalSolUMaD CKLTENAFRD VNIAFANELS MICDEI.GVN VWELISVANR HPR....VNI

PolIrgUMaD CKLTENSSRD AQIAFANELS MICDTA.GIN VWELISLANK HPR....VNI

RalSolNGD CKLTENAFRD VNIAFANELS MICDEI.GVN VWELISVANR HPR....VNI

YerEntUMaD CKLTENSFRD VNIAFANELS LICDEQ.GIN VWELIRLANR HPR....VNI

EscFerUMaD CKLTENSFRD VNIAFANELS LICADQ.GIN VWELIRLANR HPR....VNI

OceGraUMaD SKLTENSFRD VNIAFANELS LICDSL.QIN VWEVIALANR HPR....VRI

BacNRRUMaD EKILENIYRN INIGLVNELA ILSEKM.GIN IWEVIEAAKS KPYG...FQA

GeoKauUMaD EKIFENTFRH INIALANEMA ILCERM.GID VWEVIEAAKT KPYG...FMA

PsyCNPUMaD VKLTENASRD SQIAFANELS MICDKQ.GIN VWELIELANL HPR....VNI

MorPE3UMaD AKLTENSCRD VQIAFANELS MICDKL.DID VWELISLANR HPR....INI

SerProUMaD CKLTENSFRD VNIAFANELS LICADQ.GIN VWELIRLANR HPR....VNI

401 450

StrPyoUGD NNPSFGYGGY CLPKDTKQLL ANY..NNIP. .QTLIEAIVS SNNVRKSYIA

HymAerUGD1 IYPGIGYGGS CFPKDVKALI KTAQENGYQ. .MQVLQAVES VNEGQKEVLF

MetCapUGD1 IYPGCGYGGS CFPKDVKALE RTARDAGYP. .AQLLQAVEA VNDRQKEKLF

ActGloUGD6 LQPGVGWGGS CFGKDTKALI ATASEYNYD. .MPIVKAARE VNERQRAIAV

HalZhaHYPO IYPGCGYGGS CFPKDVQALS RTAQQVGYK. .AEMLEAVEA INERQKHTLF

MCIThaHYPO LDAGPGYGGS CLPKDMKAII NLSSTVGVK. .PTMLNAVEK TNKQQINNII

SalPacUGD LQPGLGFGGG CLPKDLHAFT AQAERGGVTA AAEFLRGVDG VNLHMRTRAV

NatGarNSD2 YRPGL.VGGH CIPVDPYFFA YRSAQEGFD. .PELMQTGRQ VNESVPDHVA

BacSubUGD2 LQAGIGYGGS CFPKDLQALQ FAAQEKNTE. .TFLLRAVQH INDTQLGLYI

AzoTolUGD NNPSFGYGGY CLPKDTKQLL ANY..RDVP. .QNLIHAIVE ANTTRKDFIA

LacCreEPS NNPSFGYGGY CLPKDTKQLL ANY..ADVP. .QNMMSAIVE SNRTRKDFIA

AlaProUG NNPSFGYGGY CLPKDTKQLL ANY..ENVP. .QALIQAIVS SNSTRKDVIA

StrZooUG NNPSFGYGGY CLPKDSKQLL ANY..RGIP. .QSLMSAIVE SNKIRKSYLA

StrHGBNSD FTPGPGVGGH CLPIDPSYLS WQARRSLGS. GFRFVELAND VNDRMPDYVV

NatGarNSD MRSGLGWGGS CFPKDVNALR AGAREQGYD. .PELLDAVVA VNDEQPRRLV

TheMelUGD NNPSFGYGGY CLPKDTKQLL ANYEKDKVP. .QKLIKAIVE SNQVRKQHIA

SulDenUGD NNPSFGYGGY CLPKDTKQLL ANY..SNVP. .SNMIEAIVK SNSTRKDFIA

SphAlaUGD NNPSFGYGGY CLPKDTKQML ANY..KDVP. .QNLIQAIVS SNTTRKDFVA

SheOneUGD NNPSFGYGGY CLPKDTKQLL ANY..NDVP. .NNLISAIVD SNTTRKDFIA

SalSerUGD NNPSFGYGGY CLPKDTKQLL ANY..QSVP. .NNIISAIVE ANRTRKDFIA

PseHalUGD NNPSFGYGGY CLPKDTKQLL ANY..KDIP. .NNMICAIVD ANTTRKNFIA

ProMirUGD NNPSFGYGGY CLPKDTKQLL ANY..ESVP. .NNIIGAIVE ANRTRKDFIA

ParDisUGD NNPSFGYGGY CLPKDTKQLL ANY..ANVP. .ENLIQAIVE SNRTRKDFIA

ParDisUGD1 NNPSFGYGGY CLPKDTKQLL ANY..VDVP. .ENLIGAIVE SNRTRKDFIA

LacSalUGD NNPSFGYGGY CLPKDTKQLL ANY..QDIP. .EDLIEAIVK SNSTRKSFIA

CloPerUGD NNPSFGYGGY CLPKDTKQLL ANY..NEVP. .QNMMSAIVE SNRTRKDFIA

ArcButUGD NNPSFGYGGY CLPKDTKQLL ANY..SEVP. .SNLIEAIVK SNSTRKDFIA

AerHydUGD NNPSFGYGGY CLPKDTKQLL ANY..NNVP. .NNLIRAIVD SNTTRKDFVA

LacJohSBP NNPSFGYGGY CLPKDTKQLL ANY..KDVP. .ENLIEAIVK SNDTRKDFIA

StrUbeUGD NNPSFGYGGY CLPKDTKQLL AGY..DGIP. .QSLIKAIVD SNKIRKEYIA

ReiMEDNSD NNPSFGYGGY CLPKDTKQLL ANF..EEVP. .NNLIKAIVD ANTTRKDFIA

ExiSibNSD NNPSFGYGGY CLPKDTKQLL ANY..DEVP. .NNIIGAIVE ANRTRKDYIA

RumObeHYPO NNPSFGYGGY CLPKDTKQLL ANY..NDVP. .QNMMSAIVE SNRTRKDFIA

RumObeHYP1 NNPSFGYGGY CLPKDTKQLL ANY..ADVP. .QNMMSAIVE SNRTRKDFIA

FaePraHYPO NNPSFGYGGY CLPKDTKQLL ANY..ADVP. .ENLIEAIVE SNRTRKDYIA

EubDolHYPO NNPSFGYGGY CLPKDTKQLL ANY..EDVP. .QNMMSAIVE SNRTRKDFIA

BacPleHYPO NNPSFGYGGY CLPKDTKQLL ANY..EDVP. .ENLIQAIVE SNRTRKDFIA

BacCopHYPO NNPSFGYGGY CLPKDTKQLL ANY..QDVP. .ENLIQAIVE SNRTRKDFIA

StrTheESP NNPSFGYGGY CLPKDTKQLL ANY..ADVP. .ENLIEAIVE SNRTRKDFIA

StrIniCPS NNPSFGYGGY CLPKDTKQLL ANY..RDVP. .QNMMTAIVE SNRTRKDYIA

SerProUGD IYPGCGYGGS CFPKDVQALI RTAEHIGYQ. .PKLLQAVEQ VNYQQKYKLT

GeoMetUGD LFPGVGYGGS CFPKDVKALI KTAEECEYD. .FVLLKSVEE VNERQKAILI

GeoUraUGD LFPGVGYGGS CFPKDVKALV KTAEECDYE. .FILLKAVEE VNELQKLVLT

AnoFlaUGD LRAGIGYGGS CFPKDTKALA KIAANIDHD. .FELLKAVIE VNNKQQRKLI

PorGinNSD LYPGCGYGGS CFPKDVKALI RTAEDNGYR. .MEVLEAVER VNEKQKSILF

BacSelRPON LNAGIGYGGS CFPKDTKALE NIAKQHGYE. .LRTVKAAID VNIDQKTRLY

PelPhaNSD IYPGIGYGGS CFPKDVQALE RTAHKHGYH. .SRILQAVEA VNHDQKSSIV

MetPopNSD LHAGPGYGGS CFPKDTLALV KTAQDYGTP. .VRIVETVVA VNDQRKRAMA

MetExtNSD LHAGPGYGGS CFPKDTLALV KTAQDYGTP. .VRIVETVVA VNDQRKRAMA

VibVulPRE NNPSFGYGGY CLPKDTKQLL ANY..QDIP. .NNIIGAIVD ANRTRKDFVA

GeoKauNSD LRAGVGYGGS CLPKDTKALY ALAASHGYS. .LKTVWAAMD VNEKQKWKLF

RhiTriEXO LHAGPGYGGS CFPKDTLALA KTAQDYDAP. .MRLIETTIS INDNRKRAMG

ParDisUGD2 NNPSFGYGGY CLPKDTKQLL ANY..EDVP. .ENLIEAIVE SNRTRKDFIA

XanAxoUGD NNPSFGYGGY CLPKDTKQLL ANY..QSVP. .QTIIRAIVD SNTTRKDFIA

XanCamUGD NNPSFGYGGY CLPKDTKQLL ANY..QSVP. .QNIIRAIVD SNTTRKDFIA

EntFaeUGD NNPSFGYGGY CLPKDTKQLL ANY..EDVP. .ENLIEAIVE SNRTRKNFIA

StrPneUGD2 NNPSFGYGGY CLPKDSKQLL ANY..FDVP. .QNMMTATVE SNRTRKDFIA

StrPneUGD3 NNPSFGYGGY CLPKDTKQLL ANY..EDVP. .EELIGAIVR SNSTRKDFIA

StrPneUG NNPSFGYGGY CLPKDTKQLL ANY..EDIP. .EELIGAIVR SNSTRKDFIA

EscAlbUGD NNPSFGYGGY CLPKDTKQLL ANY..QSVP. .NNLISAIVD ANRTRKDFIA

BacTheUGD NNPSFGYGGY CLPKDTKQLL ANY..QDVP. .QNMMTAIVE SNRTRKDFIA

SacSpiUGD FSPGPGVGGH CLPIDASYLS WQVRRSLG.Q EFRLVDVAND INKHMPDHVV

Aer159PBP FKPGL.VGGH CISVDPYYLT HKAQEVG..Y RPEVILAGRR INDGMGEYVA

ButSynUGD NNPSFGYGGY CLPKDTKQLL ANY..ADVP. .QDMMSAIVE SNRTRKDFIA

BraJapUGD LHAGPGFGGS CFPKDTKALI KIAQDYDVS. .LRIVESVLA VNENRKRAMA

SynCC9UGD LNAGPGFGGS CFQKDILNLV YLCRHFGLPE VADYWESVVA LNTWQQHRIA

StePneUGD NNPSFGYGGY CLPKDTKQLK ASF..RDVP. .ENLITAVVQ SNKTRKDYIA

SalAreUGD LVPGVGVGGS CLTKDPWMAW RDGQDRGVR. .LRTVETARA VNDDMPGHVA

BurYI2NSD FRPGL.VGGH CIGVDPYYLT HKAQAIG..Y HPEIILAGRR LNDSMGNYVV

BacCerNSD LNAGIGFGGS CFPKDTKALE YLARQNGYE. .LRTIKAAID VNKDQKTLLY

PaeSanHYPO FYPGPGVGGH CIPVDPLYLQ WKARQLE..A DSTFIEASTR INRSMPAYVV

Rhi42MUGD LHAGPGYGGS CFPKDTLALV KTAQDHDSP. .VRLIETTVA INDNRKRAMG

OpiTAV5NSD LKASVGFGGS CFQKDILNLV YLCEYFGLPE VASYWDQVIK INEFQKHRFA

MarHTCUGD8 LNSSVGFGGS CFQKDILNLV YIARSFGLNE VADYWEQVIL MNDYQKKRFA

BacSubUGD LKAGIGYGGS CFPKDTNALV QIAGNVEHD. .FELLKSVIK VNNNQQAMLV

NocKunUGD FYPGPGVGGH CIPIDPNYLS YKVKTLG..Y PFRFVELAQE INGRMPAYVT

DesHafUGD FWPGPGVGGH CIPIDPFYLT WKAREYD..F HTRFIELAGE INVEVSYHVI

BacThuUGD LQPGPGVGGH CIAVDPWFIV DAVP.....E QAKLIHAARK VNDYKPGYVV

SphJapUGD LSPGPGVGGH CIAVDPWFIV HGDP.....E NARIIRTARE VNDGKTDYVV

RicSibUGD LNAGPGFGGS CFPKDILALN NLVENHHID. .CRILEAVIK SNKQRPSNMV

MetCapUGD IYPGCGYGGS CFPKDVKALE RTARDAGYP. .AQLLQAVEA VNDRQKEKLF

HymAerUGD IYPGIGYGGS CFPKDVKALI KTAQENGYQ. .MQVLQAVES VNEGQKEVLF

BacSubUGD7 LKAGIGYGGS CFPKDTNALV QIAGNVEHD. .FELLKSVIK VNNNQQAMLV

ActGloUDG9 LQPGVGWGGS CFGKDTKALI ATASEYNYD. .MPIVKAARE VNERQRAIAV

NatGarNSD5 MRSGLGWGGS CFPKDVNALR AGAREQGYD. .PELLDAVVA VNDEQPRRLV

UncBacHYPO LKGGVGYGGP CFPRDNKAFV AFAAEQGVD. .AKLARAVEK INDEQIYLLY

HalZhaHYPP IYPGCGYGGS CFPKDVQALS RTAQQVGYK. .AEMLEAVEA INERQKHTLF

StrPneUGD1 NNPSFGYGGY CLPKDTKQLL ANY..EDVP. .EELIGAIVR SNSTRKDFIA

HoePhoUGD LNAGPGYGGS CFPKDTLALL RTAQDHDSP. .IRVIETIVG INETRKRAMA

CapSpuUGD IYPGCGYGGS CFPKDVLALK KLAEEVDYK. .AELIESVDK VNNRQKYVIA

NovAroUGD LHAGPGYGGS CFPKDTLALL KTAEDYNSP. .VRLVEAVVK VNDSRKRAMG

ProMarUGD LKSGPGFGGS CFKKDILNLV YLAKFYGLNE VAGYWRKVIE INSWQQKRIY

HomSapUGD LKASVGFGGS CFQKDVLNLV YLCEALNLPE VARYWQQVID MNDYQRRRFA

MusMusUNA LKASVGFGGS CFQKDVLNLV YLCEALNLPE VARYWQQVID MNDYQRRRFA

DanRerUGD LKASVGFGGS CFQKDVLNLV YLCEALNLPE VASYWQQVID MNEYQRKRFA

XenLaeUGD LKASVGFGGS CFQKDVLNLV YLCEVLNLHE VAKYWQQVID MNDYQRRRFT

RatNorUGD LKASVGFGGG CFQKDVLNLV YLCEALNLPE VARYWQQVID MNDYQRRRFA

PonAbeUGD LKASVGFGGS CFQKDVLNLV YLCEALNLPE VARYWQQVID MNDYQRRRFA

BosTauUGD3 LKASVGFGGS CFQKDVLNLV YLCEALNLPE VARYWQQVID MNDYQRRRFA

BamOldUGD LSASVGFGGS CFQKDILNLV YICECNGLPE VANYWKQVIK INDYQKSRFV

PedCorUGD LQASIGFGGS CFQKDLLNLV YMCECLNLPH VANYWQQVLD INQYQKTRFT

ZeaMayUGD8 LNASVGFGGS CFQKDILNLV YICECNGLPE VANYWKQVIK INDYQKSRFV

StrPurUGDP LQASLGFGGS CFQKDVLNLV YLCEACNIPE VAQYWQQVID MNDYQRRRFA

CioIntUGDP LQASIGFGGS CFQKDVLNLV YLCEALNLPE VANYWQQVIE INNYQRRRFA

NemVecPPRE LQASVGFGGS CFQKDVLNLV YLCEALNLPE VANYWYQVIS MNEYQRRRFT

CaeEleUGD LQASVGFGGS CFQKDVLSLV YLCESLNLPQ VADYWQGVIN INNWQRRRFA

PopTomUGD LNASVGFGGS CFQKDILNLV YICECNGLPE VAEYWKQVIK INDYQKSRFV

DroMelUGD LQASVGFGGS CFQKDILNLI YICENLNLPE VAAYWQQVID MNEYQKRRFS

AraThaUGD LNSSVGFGGS CFQKDILNLV YICECNGLPE VAEYWKQVIK INDYQKSRFV

DroMelUGD1 LQASVGFGGS CFQKDILNLI YICENLNLPE VAAYWQQVID MNEYQKRRFS

NasVitUDG MQASVGFGGS CFQKDILNLV YICECLNLPE VAAYWQQVID MNEYQKSRFS

CaeBriHYPO LKASVGFGGS CFQKDVLSLV YLCESLNLPQ VAEYWQGVIN VNNWQRRRFA

DroWilGK3 LQASVGFGGS CFQKDILNLI YICENLNLPE VAAYWQQVID MNDYQKRRFS

NasVitUGDP LQASVGFGGS CFQKDILNLI YICENLNLPE VAAYWQQVID MNEYQKRRFS

DroVirGJ3 LQASVGFGGS CFQKDILNLI YICENLNLPE VAAYWQQVID MNDYQKRRFS

HomSapUGD2 LKASVGFGGS CFQKDVLNLV YLCEALNLPE VARYWQQVID MNDYQRRRFA

XenTroUGD LQASIGFGGS CFQKDILNLI YICETLSLHE VALYWQQVLD INEYQRRRFA

FlaCF1UGD LKASVGFGGS CFQKDILNLV YIAKSYGLNE VADYWEQVII MNDYQKRRFS

ZeaMayUGD LNASVGFGGS CFQKDILNLV YICECNGLPE VANYWKQVIK INDYQKSRFV

MayZebUGD1 LKASVGFGGS CFQKDVLNLV YLCEALNLPE VASYWQQVID MNEYQRRRFA

VitvinUGD1 LNASVGFGGS CFQKDILNLV YICECNGLPE VANYWKQVIK VNDYQKTRFV

HomSapUGD1 LKASVGFGGS CFQKDVLNLV YLCEALNLPE VARYWQQVID MNDYQRRRFA

OviAriUGD1 LKASVGFGGS CFQKDVLNLV YLCEALNLPE VARYWQQVID MNDYQRRRFA

FelCatUGD1 LKASVGFGGS CFQKDVLNLV YLCEALNLPE VARYWQQVID MNDYQRRRFA

EquCabUGD1 LKASVGFGGS CFQKDVLNLV YLCEALNLPE VARYWQQVID MNDYQRRRFA

CanFamUGD1 LKASVGFGGS CFQKDVLNLV YLCEALNLPE VARYWQQVID MNDYQRRRFA

DroMojGI7 LQASVGFGGS CFQKDILNLI YICENLNLPE VAAYWQQVID MNEYQKRRFS

DroPseGA34 LQASVGFGGS CFQKDILNLI YICENLNLPE VATYWQQVID MNDYQKRRFS

HomSapICRA LKASVGFGGS CFQKDVLNLV YLCEALNLPE VARYWQQVID MNDYQRRRFA

ColLivUGD LKASVGFGGS CFQKDVLNLV YLCEALNLPE VARYWQQVID MNDYQRRRFA

CamFloUGD LHASVGFGGS CFQKDILNLV YICECLNLPE VAAYWQQVID MNEYQKSRFS

BosTauUGD LKASVGFGGS CFQKDVLNLV YLCEALNLPE VARYWQQVID MNDYQRRRFA

MelGal1UGD LKASVGFGGS CFQKDVLNLV YLCEALNLPE VARYWQQVID MNDYQRRRFA

AilMelUGDP LKASVGFGGS CFQKDVLNLV YLCEALNLPE VARYWQQVID MNDYQRRRFA

MusFurUGDP LKASVGFGGS CFQKDVLNLV YLCEALNLPE VARYWQQVID MNDYQRRRFA

MusFur1UGD LKASVGFGGS CFQKDVLNLV YLCEALNLPE VARYWQQVID MNDYQRRRFA

MelUnd1UGD LKASVGFGGS CFQKDVLNLV YLCEALNLPE VARYWQQVID MNDYQRRRFA

FalPer1UGD LKASVGFGGS CFQKDVLNLV YLCEALNLPE VARYWQQVID MNDYQRRRFA

ChrBel2UGD LKASVGFGGS CFQKDVLNLV YLCEALNLPE VARYWQQVID MNDYQRRRFA

FelCat1UGD LKASVGFGGS CFQKDVLNLV YLCEALNLPE VARYWQQVID MNDYQRRRFA

SaiBolUGDP LKASVGFGGS CFQKDVLNLV YLCEALNLPE VARYWQQVID MNDYQRRRFA

DroWilGK1 LQASVGFGGS CFQKDILNLI YICENLNLPE VAAYWQQVID MNDYQKRRFS

CaeRemQV40 LKASVGFGGS CFQKDVLSLV YLCESLNLPQ VAEYWQGVIN VNNWQRRRFA

CaeBriQV40 LKASVGFGGS CFQKDVLSLV YLCESLNLPQ VAEYWQGVIN VNNWQRRRFA

ZeaMayUGDI LNASVGFGGS CFQKDILNLV YICECNGLPE VANYWKQVIK INDYQKSRFV

GalGalUG LKASVGFGGS CFQKDVLNLV YLCEALNLPE VARYWQQVID MNDYQRRRFA

CaeEleUG LQASVGFGGS CFQKDVLSLV YLCESLNLPQ VADYWQGVIN INNWQRRRFA

PseAerGMD MRPGFAFGGS CLPKDVRALT YRASQLDVE. .HPMLGSLMR SNSNQVQKAF

CanCloGM FKPGFAYGGS CLPKDLKALS TLAKDCSLD. .TPLLDSIED SNQNHINYAI

VibCraGMD LQPGPGVGGH CIAVDPWFIV SKTP.....D EARIIHTARK VNDSKPGWVV

PseSyrGMD MRPGFAFGGS CLPKDVRALT YRAGSLDVD. .APLLNSLMR SNTSQVQNAF

AmyAzuGMD LRPGFAFGGS CLPKDLRGLV YAAHRADVA. .VPILSHVLP SNDEHLQRAF

OxaAB1GMD8 MKPGFAFGGS CLPKDVRALT YKARSLDLD. .LPLLNSVLP SNQKQVEKGL

ActGloGDM LKPGFAFGGS CLPKDLRGLV YAARRADVA. .VPLLSHVLP SNEEHLKRAI

AmyAlbGDM7 LRPGFAFGGS CLPKDLRGLV YAAHRADVA. .VPILSHVLP SNDEHLQRAF

AmyBalGDM LKPGFAFGGS CLPKDLRGLV YAAHRADVK. .VPILSHVLA SNDEHLQRAF

PseDC3GMD5 MRPGFAFGGS CLPKDVRALT YRAGSLDVD. .APLLNSLMR SNTSQVQNAF

PseVerGMD1 MRPGFAFGGS CLPKDVRALT YRAGSLDVE. .APLLNSLMR SNESQVQNAF

PseQDAGMD1 MRPGFAFGGS CLPKDVRALT YRAASLDVR. .APLLDSLMR SNESQVQNAF

PseAerGMD1 MRPGFAFGGS CLPKDVRALT YRASQLDVE. .HPMLGSLMR SNSNQVQKAF

PseHYSGMD1 LRPGFAFGGS CLPKDVRALT YRAGQLDVE. .HPLLASIMA SNRNQVKNAF

AmyAlbGMD6 LRPGFAFGGS CLPKDLRGLV YAAHRADVA. .VPILSHVLP SNDEHLQRAF

AmyAzuGMD1 LRPGFAFGGS CLPKDLRGLV YAAHRADVA. .VPILSHVLP SNDEHLQRAF

AlgSagHYPO LKPGFAFGGS CLPKDLRGLL ALANEHSVR. .LPVLDAILR TNLHQIQRAY

AciKBSHYPO LKPGFAFGGS CLPKDLRALA HRASRLDLK. .LPLLEATLP SNQEHLHRAI

AmyBalGMD LKPGFAFGGS CLPKDLRGLV YAAHRADVK. .VPILSHVLA SNDEHLQRAF

StrCanGMD1 LRPGFAFGGS CLPKDLRSLV HAAQRADVS. .VPILSHVLA SNSDHLQRAV

PseAlcGMD1 MKPGFAFGGS CLPKDVRALS YRAGSLDVE. .TPLISSLMR SNAAQVKKAF

ActGloGMD6 LKPGFAFGGS CLPKDLRGLV YAARRADVA. .VPLLSHVLP SNEEHLKRAI

OxaAB1GMD1 MKPGFAFGGS CLPKDVRALT YKARSLDLD. .LPLLNSVLP SNQKQVEKGL

PseAerUNPP MRPGFAFGGS CLPKDVRALT YRASQLDVE. .HPMLGSLMR SNSNQVQKAF

HahGanHYPO MRPGFAFGGS CLPKDVRALT YRASQVDVR. .HPLLASIMD SNAYQVAKAF

DesSalNSD MKPGFAFGGS CLPKDVRALT YKANQLSLD. .LPLLNNVLR SNRRHIERGL

PseTomGMD1 MRPGFAFGGS CLPKDVRALT YRASSLDVE. .APLLNSLMR SNTSQVQNAF

MarAlgGMD MRPGFAFGGS CLPKDVRALT YRANQMDVK. .HPLLSSIMS SNNEQVAHAF

PseSyrGMD3 MRPGFAFGGS CLPKDVRALT YRAGSLDVD. .APLLNSLMR SNTSQVQNAF

NovAroGMD TRPGGPFGGS CLPKDVRALQ HIAADTGAQ. .THLVDSLLR SNDAHKHHQF

AzoSpGMD LKPGFAFGGS CLPKDLRATS YLAKTHDVE. .LPMLNGILQ SNRHHVEEAI

AzoVinGMD MRPGFAFGGS CLPKDVSALS YRAHLWDIE. .APLISSLMR SNAAQVQKAY

BacThuGMD HTPGPGVGGH CLAVDPYFII EKDP.....Q NAKLISDARE INNSMPNFVV

HahGanHYPP MRPGFAFGGS CLPKDVRALT YRASQVDVR. .HPLLASIMD SNAYQVAKAF

AlgSagHYPP LKPGFAFGGS CLPKDLRGLL ALANEHSVR. .LPVLDAILR TNLHQIQRAY

AciKBSHYPP LKPGFAFGGS CLPKDLRALA HRASRLDLK. .LPLLEATLP SNQEHLHRAI

PseSyrGMD2 MRPGFAFGGS CLPKDVRALT YRASSLDVE. .APLLNSLMR SNTSQVQNAF

PseSyrGMD1 MRPGFAFGGS CLPKDVRALT YRASSLDVE. .APLLNSLMR SNTSQVQNAF

StrCanGMD LRPGFAFGGS CLPKDLRSLV HAAQRADVS. .VPILSHVLA SNSDHLQRAV

PseVerGMD MRPGFAFGGS CLPKDVRALT YRAGSLDVE. .APLLNSLMR SNESQVQNAF

PseQDAGMD MRPGFAFGGS CLPKDVRALT YRAASLDVR. .APLLDSLMR SNESQVQNAF

PseHYSGMD LRPGFAFGGS CLPKDVRALT YRAGQLDVE. .HPLLASIMA SNRNQVKNAF

PseAlcGMD MKPGFAFGGS CLPKDVRALS YRAGSLDVE. .TPLISSLMR SNAAQVKKAF

PseAerUPP MRPGFAFGGS CLPKDVRALT YRASQLDVE. .HPMLGSLMR SNSNQVQKAF

StaAurUMD HQPGPGVGGH CLAVDPYFII AKDP.....E NAKLIQTGRE INNSMPAYVV

EscColUMND LQPGPGVGGH CIAVDPWFIV AQNP.....Q QARLIRTARE VNDHKPFWVI

LusFleUGD HTPGPGVGGH CLAVDPYFVV ASAP.....E ESPLIQTARA INVSMPEFVE

SalEntUMD LQPGPGVGGH CIAVDPWFIV AQNP.....Q QARLIRTARE VNDGKPHWVV

ActSerUG LQPGAGVGGH CIAVDPWFIA AQTP.....D QSRLIRTARE VNDSKPQWVI

ErwTasUG LQPGPGVGGH CIAVDPWFIV AQNP.....D LARLIRTARE VNDGKPHWVL

FusUlcUMD LTPGAGVGGH CLAVDPWFIV EKFP.....K EANVIREARL INDFKPRFIV

CorMarUMD LQPGPGVGGH CIAVDPWFIV SAAP.....A EARLIRTARE VNDRKPEWVV

StaVitUMD HLPGPGVGGH CLAVDPYFII AKAP.....E TTTLIQEGRR INRSMPQYVI

SheVioUMD LQPGAGVGGH CIAVDPWFIV NQNP.....D EAKLIHQARK TNDFKPEWVI

SerS4UMD LQPGPGVGGH CIAVDPWFIV SQNP.....Q QARLIHTARL VNDGKPLWVV

PseStuUMD LQPGPGVGGH CIAVDPWFIV SQAE.....G LAKLIRTARE TNDSKPSWVV

PsePseUMD LQPGPGVGGH CIAVDPWFIV SRMP.....E QARLIRTARE VNDSKPEWVL

PseFluUMD LQPGPGVGGH CIAVDPWFIV SKTP.....D IARLIRTARE VNDSKPEWVL

HalSalUMD HSPGPGVGGH CLPVDPQFLT QS.T.....T DSRLISVARD INESMAVHTL

FraPhiUMD LQPGCGVGGH CIAVDPWFIV NAFP.....E DAKIIGTARR VNDSKPHYVI

EdwC07UMD LQPGPGVGGH CIAVDPWFIV AQNP.....Q LARLIHTARL VNDGKPLWVV

AerAquUMD LQPGCGVGGH CIAVDPWFIV NQNP.....D TAKIIHQARL INDYKPHYVV

GeoWSUUMD FYPGPGLGGH CIPIDPFYLT WKAREYN..Y HTRLIELAGE INNAMPEYVV

EscMG1UMD LQPGPGVGGH CIAVDPWFIV AQNP.....Q QARLIRTARE VNDHKPFWVI

DicZeaUMD LQPGPGVGGH CIAVDPWFIV AQHP.....Q QARLIRTARE VNDDKPRWVV

VibOrdUMD LQPGPGVGGH CIAVDPWFIV SKTP.....L EAQIIHTARK VNNSKPHWVV

VidVulUMaD LQPGPGVGGH CIAVDPWFIV SKTP.....E EAQIIHMARK VNDSKPQWVI

KlePneUMaD LQPGPGVGGH CIAVDPWFIV AQNP.....Q QARLIRTARE VNDHKPEWVI

GeoTheUMaD HLPGPGVGGH CLAVDPYFIV EKAK.....E ESQLIQTARR INNSMPHFVV

BurXenUMaD LQPGPGVGGH CIAVDPWFIV DSAP.....E EAKLIRAARG VNDGKPHYVI

HasParUMaD LQPGAGVGGH CIAIDPWFIV AQSE...... HARLIRTARE VNDSKAFWVI

RalSolUMaD LQPGPGVGGH CIAVDPWFIV DAAP.....E SARLIRTARE VNDAKPHYVL

PolIrgUMaD LQPGCGVGGH CIAVDPWFIV SEFP.....E QAQIIKRVRE TNDYKADWCA

RalSolNGD LQPGPGVGGH CIAVDPWFIV DAAP.....E RARLIRTARE VNDAKPHYVL

YerEntUMaD LQPGPGVGGH CIAVDPWFIV SQNP.....Q LARLIHTARL VNDGKPLWVV

EscFerUMaD LQPGPGVGGH CIAVDPWFIV AQNP.....Q QARLIRTARE VNDHKPFWVI

OceGraUMaD LRPGPGVGGH CIAVDPWFIV ASSP.....E NSRLIRAARH VNSSKPSWVL

BacNRRUMaD FYPGPGLGGH CIPLDPYYLS WKAREYG..F HTSMIESSMI VNDRMPEYCV

GeoKauUMaD FYPGPGLGGH CIPIDPFYLT WKAREYN..Y HTRLIELAGE INNAMPEYVV

PsyCNPUMaD LQPGAGVGGH CIAVDPWFIV NQNP.....E EAKLIHQARL TNDAKPLWVI

MorPE3UMaD LQPGPGVGGH CIAVDPWFIV SKTP.....E EARMIHTARK VNDSKPGWVI

SerProUMaD LQPGPGVGGH CIAVDPWFIV SQNP.....Q QARLIHTARL VNDGKPLWVV

451 500

StrPyoUGD KQIINVLKEQ E......... ..SPVKVVGV YRLIMKSNSD NFRESAIKDV

HymAerUGD1 NKVKKHFGSE .......... ..LKGKKMAV WGLSFKPKTD DMREAPSLVI

MetCapUGD1 EKISAYFGGD .......... ..LAGKTIAV WGLAFKPNTD DMREAPSRTL

ActGloUGD6 ERLQDELRI. .......... ..LKGRKIGL LGLAFKPNTD DLRDSPALDI

HalZhaHYPO EKLSRAFNGE .......... ..LEGKKIGV WGLAFKPNTD DMREAPSRVL

MCIThaHYPO ELIKQNIGK. .......... ..IKGKKITV LGVAFKPNTD DIRDSMSIEL

SalPacUGD ALATAALNGE .......... ..VRGRRIAL WGAAFKPGID DVRDSPALDV

NatGarNSD2 ELTIKALNQC HKT....... ..LRESRVLV LGLAYKPDVG DIRSSKIGTV

BacSubUGD2 KKIQSFFET. .......... ..LQGKKAAV LGISFKPNTD DIRNSQAVRL

AzoTolUGD DSILK..... .......... ..RNPKVVGV YRLIMKAGSD NFRASSIQGI

LacCreEPS DQVLKMAGYY DYFNRGDYSA VQEKECIVGI YRLTMKSNSD NFRQSAIQGV

AlaProUG DEIIK..... .......... ..KNPEVVGF YRLVMKEGSD NFRSSAIQGV

StrZooUG EQILDRASSQ KQA....... ..GVPLTIGF YRLIMKSNSD NFRESAIKDI

StrHGBNSD TRAIQSLNRR RKA....... ..VNGSSVLL LGLTYKPNSR DARKSPALAV

NatGarNSD GLLADHVS.. .......... ..LSGARIAV LGLSFKPGTD DVRKSRALDV

TheMelUGD YMIER..... .......... ..RKPKVIGV YRLIMKKDSD NFRQSAILDV

SulDenUGD DSILA..... .......... ..KKPKIVGV YRLVMKSGSD NFRSSAIQGI

SphAlaUGD SEVIK..... .......... ..RNPRVVGI HRLAMKAGSD NFRASSILGV

SheOneUGD DAIIS..... .......... ..KQPKRVGI YRLIMKSESD NFRASAVQGV

SalSerUGD DAILA..... .......... ..RKPEVVGI YRLIMKSGSD NFRASSIQGI

PseHalUGD DSIIK..... .......... ..RNPKVVGI YRLVMKTGSD NFRASAIQGI

ProMirUGD DSIIA..... .......... ..KSPKIVGV YRLIMKSGSD NFRSSSIQGI

ParDisUGD DRVLNKAGYY DYYNRGDFNP NEERKCVIGV YRLTMKSNSD NFRQSSIQGV

ParDisUGD1 DQVLRKAGYY DYYNRGDFNP MNERHCTIGV YRLTMKSNSD NFRQSSIQGV

LacSalUGD DQVLKKAGYY DYSQDMIYNP NEEKQPVVGV YRLTMKSNSD NFRESSIQGV

CloPerUGD DRVLELAGAY EA..NSEWEA SKEKEVVIGV YRLTMKSNSD NFRQSSIQGV

ArcButUGD DSIIR..... .......... ..KNPKIVGI YRLVMKTGSD NFRSSAIQGI

AerHydUGD DSVIR..... .......... ..KNPRRVGV YRLVMKAGSD NFRASAIQGV

LacJohSBP DQVLSKAGYY DYDDNNTYDP SEEKQVTIGV YRLTMKSNSD NFRQSSVQGV

StrUbeUGD SQILQQLSDI NVD....... ..PKDATIGI YRLIMKSNSD NFRESAIKDI

ReiMEDNSD DSIIK..... .......... ..RGPKRVGI YRLVMKSGSD NFRASAIQGI

ExiSibNSD DTIIK..... .......... ..QNPKVVGV YRLTMKTDSD NFRASSIQGI

RumObeHYPO DRVLQKAGYY GYDEENTYNS SMEKSVTVGV YRLTMKSNSD NFRQSSIQGV

RumObeHYP1 DRVLQKAGYY AYGDENTYDA SMEKEVVIGV YRLTMKSNSD NFRQSSIQGV

FaePraHYPO DRVLEIAGAY EA..NEAYDP SKEHNVVVGV YRLTMKSNSD NFRQSSIQGV

EubDolHYPO DRVLEKAGYY SYQDNNEYDS ANEKEITVGV YRLTMKSNSD NFRQSSIQGV

BacPleHYPO EQVLNKAGYY S..ANSQWNV TKEHEVIVGV YRLTMKSNSD NFRQSSIQGV

BacCopHYPO DRVLHKAGYY DYYNRGSYNP AEEKECVIGV YRLTMKSNSD NFRQSSIQGV

StrTheESP DRVLEIAGAY EA..NDSWDE SKEKEVVVGV YRLTMKSNSD NFRQSSIQGV

StrIniCPS EKVLELAGAY E..GSSEFDP NMEKEVVIGV YRLTMKSNSD NFRQSSIQGI

SerProUGD KFIKHHFGED .......... ..LKGKTFAL WGLAFKPNTD DMREASSRVL

GeoMetUGD DKMIAHFSRD NGASP..... ..LAGKTIAI WGLSFKPRTD DMREAPSIVI

GeoUraUGD DKINLRLGDN SLLKP..... ..LTGKTIAI WGLSFKPRTD DMREAPSVVI

AnoFlaUGD EKAKKRFGH. .......... ..LARKKIAL LGLSFKPNTD DMREAASIVI

PorGinNSD DKFSTYYKGN .......... ..VQGRCVAI WGLSFKPGTD DMREAPSLVL

BacSelRPON KKASKRLIT. .......... ..FNGLKVAV LGLTFKPGTD DLREAASLEN

PelPhaNSD RKIRGHFTDG .......... ..IKGSVFAI WGLAFKPNTD DMREAPSRRV

MetPopNSD RKVIAACGGS .......... ..VRGKRVAL LGLTFKPNTD DMRDAPSLSI

MetExtNSD RKVIAACGGS .......... ..VRGKRVAL LGLTFKPNTD DMRDAPSLSI

VibVulPRE ESILK..... .......... ..RNPKVVGI YRLIMKAGSD NFRASSIQGI

GeoKauNSD EKARAHLGV. .......... ..FSGRTAAV LGAAFKPGTD DVRESPALAN

RhiTriEXO RKVISAVGGD .......... ..IRGKKIAI LGLTFKPNTD DMRDSPAIAV

ParDisUGD2 DQVLRMAGYY AYGEENEWSS VREHPVTIGV YRLTMKSNSD NFRQSSIQGI

XanAxoUGD ADVLR..... .......... ..RNPKVVGI YRLIMKAGSD NFRSSSIQGV

XanCamUGD ADVLR..... .......... ..RNPKVVGI YRLIMKAGSD NFRSSSIQGV

EntFaeUGD DRVLELAGAY EA..NSHWDA SKEKKVVIGV YRLTMKSNSD NFRQSSIQGV

StrPneUGD2 QRILEKAGVS E..TDSLDAF KNTQDIVIGI YRLTMKSNSD NFRHSSIQGV

StrPneUGD3 DQVLKMVDSY DYSAHNVFNE RVEKDIIIGI YRLTMKSNSD NFRQSSIQGI

StrPneUG DQVLKMVDYY DYSAHNIFDE RVEKDIIIGI YRLTMKSNSD NFRQSSIQGI

EscAlbUGD DAILS..... .......... ..RKPQVVGI YRLIMKSGSD NFRASSIQGI

BacTheUGD DQVLRKAGYY T..ASCSWDA QKEQKITIGV YRLTMKSNSD NFRQSAIQGI

SacSpiUGD ARATEHLNRR RKS....... ..VNSSAVLL IGMTYKANSG DARNSPSMAV

Aer159PBP TQLVKKMARN KIN....... ..IDEAKVLV MGFTFKGDCP DVRNTKIIDI

ButSynUGD DQVLKMAGYY KS..NSDYDA SREHACTIGV YRLTMKANSD NFRQSSIQGV

BraJapUGD RKVSQALGGS .......... ..LRGKTIAV LGLTFKPDTD DMRDAPSI..

SynCC9UGD RLVVEKLFGT .......... ..VTGKRLAI LGFAFKANTN DTREAPAIRI

StePneUGD GAILA..... .......... ..KQPSVVGI YRLIMKFDSD NFRSSAVKGV

SalAreUGD SVIVDELLGL GRG....... ..RTDSPIAV LGAAFKSDTG DVRNTPVRDV

BurYI2NSD SQLVKTMTKR DIA....... ..ISGARVLI MGLTFKENCP DLRNTRVVDI

BacCerNSD KKASQRLIT. .......... ..FNGLKVAV LGLTFKPGTD DLREAASLEN

PaeSanHYPO SRIKQTMEDS M......... ..LEGKRIMV LGVTFKEDVA DIRESSSIEM

Rhi42MUGD RKVIAACDGN .......... ..VRGKKIAV LGLTFKPNTD DMRDAPSITI

OpiTAV5NSD AKIVRTLFNT .......... ..VAGKKIAV LGFAFKKDTN DTRESPAISV

MarHTCUGD8 DNIVSTLYNT .......... ..VSGKKIVF YGWAFKKDTN DTRESAAIYI

BacSubUGD DKALNRLGG. .......... ..VTGKTIAL LGLSFKPNTD DMREAPSIVI

NocKunUGD QRSQELLNES GLA....... ..LSRSRVLV LGVTYKADIA DQRESPARPV

DesHafUGD NKVIRALNNE NKS....... ..LKDAKVLI LGVAYKKDID DVRESPALKI

BacThuUGD DKIREKADK. .......... ..FKNPIIAC LGLAFKANID DLRESPSVEI

SphJapUGD AKASDMIDA. .......... ..FAGEDIAC LGLAFKANID DFRESPAVKV

RicSibUGD DKIATLLDGD .......... ..LKGKNIAI LGLTYKAGTD DVRASPAIAI

MetCapUGD EKISAYFGGD .......... ..LAGKTIAV WGLAFKPNTD DMREAPSRTL

HymAerUGD NKVKKHFGSE .......... ..LKGKKMAV WGLSFKPKTD DMREAPSLVI

BacSubUGD7 DKALNRLGG. .......... ..VTGKTIAL LGLSFKPNTD DMREAPSIVI

ActGloUDG9 ERLQDELRI. .......... ..LKGRKIGL LGLAFKPNTD DLRDSPALDI

NatGarNSD5 GLLADHVS.. .......... ..LSGARIAV LGLSFKPGTD DVRKSRALDV

UncBacHYPO HLILSHMN.. .......... ..SQNNTLAI LGVAYKANTP VIENSPAVKL

HalZhaHYPP EKLSRAFNGE .......... ..LEGKKIGV WGLAFKPNTD DMREAPSRVL

StrPneUGD1 DQVLKMVDYY DYSAHNVFNE RVEKDIIIGI YRLTMKSNSD NFRQSSIQGI

HoePhoUGD RKIERAIGDE .......... ..LRGKTIGI LGLTFKPNTD DIRESPALTI

CapSpuUGD QKVVAKYGED .......... ..LSGKTFAV WGLSFKPETD DMREAPAIYV

NovAroUGD RKAIEALGGE .......... ..ARGKRVAL LGLTFKPNTD DMRDAPSIAI

ProMarUGD EIVVEKLFGN .......... ..LVDKKIAI LGFSFKENTN DTRESPAISI

HomSapUGD SRIIDSLFNT .......... ..VTDKKIAI LGFAFKKDTG DTRESSSIYI

MusMusUNA SRIIDSLFNT .......... ..VTDKKIAI LGFAFKKDTG DTRESSSIYI

DanRerUGD CRIIDCLFNT .......... ..VTGKKIAL LGFSFKKDTG DTRESSSIYI

XenLaeUGD TRIIDCLFNT .......... ..VTDKKIAL LGFAFKKDTG DTRESSSIYI

RatNorUGD SRIIDSLFNT .......... ..VTDKKIAI LGFAFKKDTG DTRESSSIYI

PonAbeUGD SRIIDSLFNT .......... ..VTDKKIAI LGFAFKKDTG DTRESSSIYI

BosTauUGD3 SRIIDSLFNT .......... ..VTDKKIAI LGFAFKKDTG DTRESSSIYI

BamOldUGD NRVVSSMFNT .......... ..VSGKKIAV LGFAFKKDTG DTRETAAIDV

PedCorUGD EKIIESLFCT .......... ..VTGKKIAI LGFAFKKDTG DTRESAAIFV

ZeaMayUGD8 NRVVSSMFNT .......... ..VAGKKIAV LGFAFKKDTG DTRETPAIDV

StrPurUGDP NKIIACLFNT .......... ..VTDKKIAI LGFAFKKDTG DTRESSSIYL

CioIntUGDP NHIVSSLYNT .......... ..VYGKQITL LGFAFKKDTG DTRESSAIYV

NemVecPPRE NRIINCLFNT .......... ..VSDKKIAI MGFAFKKNTG DTRESASIYV

CaeEleUGD DKIIAELFNT .......... ..VTDKKIAI FGFAFKKNTG DTRESSAIHV

PopTomUGD NRVVSSMFNT .......... ..VSQKKIAI LGFAFKKDTG DTRETPAIDV

DroMelUGD QKIIESLFNT .......... ..VSDKRIAI LGFAFKKNTG DTRETAAITV

AraThaUGD NRVVSSMFNS .......... ..VSNKKIAV LGFAFKKDTG DTRETPAIDV

DroMelUGD1 QKIIESLFNT .......... ..VSDKRIAI LGFAFKKNTG DTRETAAITV

NasVitUDG AKVIESLFNT .......... ..VTDKKISL LGFAFKKNTG DTRESPAIHV

CaeBriHYPO DKIIAELFNT .......... ..VTDKKIAI FGFAFKKNTG DTRESSAIHV

DroWilGK3 QKIIESLFNT .......... ..VSDKRIAI LGFAFKKNTG DTRETAAITV

NasVitUGDP QKIIESLFNT .......... ..VSDKRIAI LGFAFKKNTG DTRETAAITV

DroVirGJ3 QKIIESLFNT .......... ..VSDKRIAI LGFAFKKNTG DTRETAAITV

HomSapUGD2 SRIIDSLFNT .......... ..VTDKKIAI LGFAFKKDTG DTRESSSIYI

XenTroUGD SRIVNCLFNT .......... ..VADKKIAL LGFAFKKDTG DTRESSSIYV

FlaCF1UGD NKIVQTLYNT .......... ..VADKKITF LGWAFKKDTN DTRESAAIYV

ZeaMayUGD NRVVASMFNT .......... ..VAGKKIAV LGFAFKKDTG DTRETPAIDV

MayZebUGD1 CRIIDCLFNT .......... ..VTGKKIAL LGFSFKKDTG DTRESSSIYI

VitvinUGD1 NRVVSSMFNT .......... ..VSGKKIAI LGFAFKKDTG DTRETPAIDV

HomSapUGD1 SRIIDSLFNT .......... ..VTDKKIAI LGFAFKKDTG DTRESSSIYI

OviAriUGD1 SRIIDSLFNT .......... ..VTDKKIAI LGFAFKKDTG DTRESSSIYI

FelCatUGD1 SRIIDSLFNT .......... ..VTDKKIAI LGFAFKKDTG DTRESSSIYI

EquCabUGD1 SRIIDSLFNT .......... ..VTDKKIAI LGFAFKKDTG DTRESSSIYI

CanFamUGD1 SRIIDSLFNT .......... ..VTDKKIAI LGFAFKKDTG DTRESSSIYI

DroMojGI7 QKIIESLFNT .......... ..VSDKRIAI LGFAFKKNTG DTRETAAITV

DroPseGA34 QKIIESLFNT .......... ..VSDKRIAI LGFAFKKNTG DTRETAAITV

HomSapICRA SRIIDSLFNT .......... ..VTDKKIAI LGFAFKKDTG DTRESSSIYI

ColLivUGD SRIIDSLFNT .......... ..VTDKKIAI LGFAFKKDTG DTRESSSIYI

CamFloUGD AKVIESLFNT .......... ..VTDKRIAM LGFAFKKNTG DTRESPAIHV

BosTauUGD SRIIDSLFNT .......... ..VTDKKIAI LGFAFKKDTG DTRESSSIYI

MelGal1UGD SRIIDSLFNT .......... ..VTDKKIAI LGFAFKKDTG DTRESSSIYI

AilMelUGDP SRIIDSLFNT .......... ..VTDKKIAI LGFAFKKDTG DTRESSSIYI

MusFurUGDP SRIIDSLFNT .......... ..VTDKKIAI LGFAFKKDTG DTRESSSIYI

MusFur1UGD SRIIDSLFNT .......... ..VTDKKIAI LGFAFKKDTG DTRESSSIYI

MelUnd1UGD SRIIDSLFNT .......... ..VADKKIAI LGFAFKKDTG DTRESSSIYI

FalPer1UGD SRIIDSLFNT .......... ..VTDKKIAI LGFAFKKDTG DTRESSSIYI

ChrBel2UGD SRIIDSLFNT .......... ..VTDKKIAI LGFAFKKDTG DTRESSSIYI

FelCat1UGD SRIIDSLFNT .......... ..VTDKKIAI LGFAFKKDTG DTRESSSIYI

SaiBolUGDP SRIIDSLFNT .......... ..VTDKKIAI LGFAFKKDTG DTRESSSIYI

DroWilGK1 QKIIESLFNT .......... ..VSDKRIAI LGFAFKKNTG DTRETAAITV

CaeRemQV40 DKIIAELFNT .......... ..VTDKKIAI FGFAFKKNTG DTRESSAIYV

CaeBriQV40 DKIIAELFNT .......... ..VTDKKIAI FGFAFKKNTG DTRESSAIHV

ZeaMayUGDI NRVVSSMFNT .......... ..VAGKKIAV LGFAFKKDTG DTRETPAIDV

GalGalUG SRIIDSLFNT .......... ..VTDKKIAI LGFAFKKDTG DTRESSSIYI

CaeEleUG DKIIAEL.FN T......... ..VTDKKIAI FGFAFKKNTG DTRESSAIHV

PseAerGMD DLITS..... .......... ..HDTRKVGL LGLSFKAGTD DLRESPLVVL

CanCloGM KTIKN..... .......... ..KGKRKIGI FGIAFKEGTD DLRYSPIIKV

VibCraGMD GQTKIAIADF LQDNHEKT.. ..AKDVTIAC YGLAFKPDID DLRESPAMQI

PseSyrGMD DMVAS..... .......... ..YDTRKVAL LGLSFKAGTD DLRESPLVEL

AmyAzuGMD DLVAN..... .......... ..TGKRKVGL FGLSFKPGTD DLRESPLVEL

OxaAB1GMD8 KMIMD..... .......... ..KGARKVGI LGFSFKAGTD DLRESPLVDV

ActGloGDM ELVTA..... .......... ..TGKRKIGL FGLSFKPGTD DLRESPLVEL

AmyAlbGDM7 DLVAH..... .......... ..TGKRKIGL FGLSFKPGTD DLRESPLVEL

AmyBalGDM DLVAR..... .......... ..TGKRKVGL FGLSFKPGTD DLRESPLVEL

PseDC3GMD5 DMVAS..... .......... ..YDTRKVAL LGLSFKAGTD DLRESPLVEL

PseVerGMD1 DIVAG..... .......... ..HDKRKVAL LGLSFKAGTD DLRESPLVEL

PseQDAGMD1 ELIEA..... .......... ..HDKRKVAL LGLSFKAGTD DLRESPLVEL

PseAerGMD1 DLITS..... .......... ..HDTRKVGL LGLSFKAGTD DLRESPLVVL

PseHYSGMD1 ELITR..... .......... ..QDKRRIGL LGLSFKAGSD DLRESPLVEL

AmyAlbGMD6 DLVAH..... .......... ..TGKRKIGL FGLSFKPGTD DLRESPLVEL

AmyAzuGMD1 DLVAN..... .......... ..TGKRKVGL FGLSFKPGTD DLRESPLVEL

AlgSagHYPO NLIVA..... .......... ..HEPKKVAL LGVAFKPSSD DLRESPLLTL

AciKBSHYPO DLLLD..... .......... ..LPARRLGV LGLAFKENTD DLRESPVVAL

AmyBalGMD DLVAR..... .......... ..TGKRKVGL FGLSFKPGTD DLRESPLVEL

StrCanGMD1 DLVER..... .......... ..TGKRRVGL FGLSFKPGTD DLRESPLVEL

PseAlcGMD1 DIVTH..... .......... ..YDKRRIGL LGLSFKAGTD DLRESPLVEL

ActGloGMD6 ELVTA..... .......... ..TGKRKIGL FGLSFKPGTD DLRESPLVEL

OxaAB1GMD1 KMIMD..... .......... ..KGARKVGI LGFSFKAGTD DLRESPLVDV

PseAerUNPP DLITS..... .......... ..HDTRKVGL LGLSFKAGTD DLRESPLVEL

HahGanHYPO DIVHS..... .......... ..YGKRNILM LGLSFKAGTD DLRESPLVDL

DesSalNSD SMIMG..... .......... ..KGNRKVGF LGFSFKAGTD DLRESPLVEV

PseTomGMD1 DMVAS..... .......... ..YDTRKVAL LGLSFKAGTD DLRESPLVEL

MarAlgGMD KILTS..... .......... ..YGCRKVSM LGLSFKSNTD DLRESPLVEL

PseSyrGMD3 DMVAS..... .......... ..YDTRKVAL LGLSFKAGTD DLRESPLVEL

NovAroGMD LQVTKDL... .......... ..QPGARVLL VGLAFKADTD DLRESPAVDM

AzoSpGMD QKVLA..... .......... ..SGCRRVGM LGLSFKTGTD DLRESPLVTL

AzoVinGMD DMIDK..... .......... ..HGSRKVAL LGLSFKAGTD DLRESPQLEL

BacThuGMD ENVGKLLGE. .......... ...GRGKITV MGLTYKGNID DVRESPAMEI

HahGanHYPP DIVHS..... .......... ..YGKRNILM LGLSFKAGTD DLRESPLVDL

AlgSagHYPP NLIVA..... .......... ..HEPKKVAL LGVAFKPSSD DLRESPLLTL

AciKBSHYPP DLLLD..... .......... ..LPARRLGV LGLAFKENTD DLRESPVVAL

PseSyrGMD2 DMVAS..... .......... ..YDTRKVAL LGLSFKAGTD DLRESPLVEL

PseSyrGMD1 DMVAS..... .......... ..YDTRKVAL LGLSFKAGTD DLRESPLVEL

StrCanGMD DLVER..... .......... ..TGKRRVGL FGLSFKPGTD DLRESPLVEL

PseVerGMD DIVAG..... .......... ..HDKRKVAL LGLSFKAGTD DLRESPLVEL

PseQDAGMD ELIEA..... .......... ..HDKRKVAL LGLSFKAGTD DLRESPLVEL

PseHYSGMD ELITR..... .......... ..QDKRRIGL LGLSFKAGSD DLRESPLVEL

PseAlcGMD DIVTH..... .......... ..YDKRRIGL LGLSFKAGTD DLRESPLVEL

PseAerUPP DLITS..... .......... ..HDTRKVGL LGLSFKAGTD DLRESPLVEL

StaAurUMD DTTKQIIKA. .......... ..LSGNKVTV FGLTYKGDVD DIRESPAFDI

EscColUMND DQVKAAVADC LAATDKR... ..ASELKIAC FGLAFKPNID DLRESPAMEI

LusFleUGD AKVDEVMKG. .......... ..APTNKISI LGLTYKGNID DIRESPAMEI

SalEntUMD DQVKAAVADC LAVTDKR... ..ASEVKIAC FGLAFKPNID DLRESPAMGI

ActSerUG EKVKQALADC VNRRNCL... ..ACDVTIAC LGLSFKADID DLRGSPALAI

ErwTasUG EQVQQQVANC LMASDRR... ..GSELKIAC LGLAFKPDID DLRESPAVQV

FusUlcUMD NKVDEILNG. .......... ..NKELTVGV LGLAYKPDID DLRESPAMEI

CorMarUMD DRVADAVKR. .......... ..SGAGQIAA LGLAFKANID DLRESPSLEI

StaVitUMD EESKKILET. .......... ..LNGNKIVV FGLTYKGDVD DVRESPAFDI

SheVioUMD NKIEDAVKS. .......... ..IDNPKIAC LGLAFKPDID DLRESPALEI

SerS4UMD DRVKAAVADC LAATDKR... ..ASEVKIAC FGLAFKPNID DLRESPAVEV

PseStuUMD EKVDAAVADH LSRNHGKS.. ..EKDVVIAC FGLAFKPDID DLRESPSLEI

PsePseUMD GKVDQAIGAY LTANPGRT.. ..ASDMTIAC FGLAFKPDID DLRESPALGI

PseFluUMD EKTKLAVAEF LQLNPHKT.. ..ALDVKIAC LGLAFKPDID DLRESPAVAI

HalSalUMD RHVRRFLDD. .......... ..TAHARVTV LGVAYKGNVD DTRETPALRL

FraPhiUMD SKVKEAIKG. .......... ..IKTPKIAC LGLAFKPDID DLRESPALDI

EdwC07UMD DRVKAALADC LAAEDKR... ..ASEATIAC FGLAFKPDID DLRESPAMEI

AerAquUMD EQVEQAVAG. .......... ..LSNPKIAC LGLAFKPDID DLRESPALEI

GeoWSUUMD NRAMLILNEE GKA....... ..LRGSKVTV LGVAYKKDID DVRESPVLKI

EscMG1UMD DQVKAAVADC LAATDKR... ..ASELKIAC FGLAFKPNID DLRESPAMEI

DicZeaUMD DQVKIRVADA LVQGGKS... ..ARDLCIAC LGLAFKPDVE DLRESPALAI

VibOrdUMD SKTKMLIADF LQDNSDKT.. ..AKEITIAC YGLAFKADID DLRESPAMQI

VidVulUMaD NKVKLAIADF LQANPEKT.. ..AKDMTIAC YGLAFKSDID DLRESPSLEI

KlePneUMaD EQVKAQVADC LNATNKR... ..ASELTIAC FGLAFKPNID DLRESPAMEI

GeoTheUMaD EQVKRMTAE. .......... ..LEAPVIAV FGLTYKGNTD DVRESPAIEI

BurXenUMaD DQTKRLAAR. .......... ..FKAPVIAC LGLAFKANID DLRESPAIDI

HasParUMaD DKIKSCLAEC AVATNRK... ..ISDLTLAC LGITFKANVS DLRESPALAI

RalSolUMaD DRVKQAARR. .......... ..FKEPVIAC FGLSFKANID DLRESPAIEI

PolIrgUMaD NKVIEACQYF ANKE...... ..GKDPVVAC MGLAFKPNID DLRESPAKYI

RalSolNGD DRVKQAARR. .......... ..FKEPVIAC FGLSFKANID DLRESPAIEI

YerEntUMaD DRVKAAVADC LAATDKR... ..ASEVKIAC FGLAFKPNID DLRESPAVEI

EscFerUMaD DQVKAAVADC LAATDKR... ..ASELKIAC FGLAFKPNID DLRESPAMEI

OceGraUMaD GKIDEAVATF LRQYPKLD.. ..EEDVTIAC YGIAFKPDID DMRESPALQI

BacNRRUMaD ERASKILNKV KKA....... ..MNGSRVLV LGVAYKKDID DYRESAALRV

GeoKauUMaD NRAMLILNEE GKA....... ..LRGSKVTV LGVAYKKDID DVRESPVLKI

PsyCNPUMaD NKIEEAISN. .......... ..MVKPKIAC LGLAFKPDID DLRESPALNI

MorPE3UMaD NKVKIAIADF LQENPEKT.. ..AKDVTIAC YGLAFKPDID DLRESPALDI

SerProUMaD DRVKAAVADC LAATDKR... ..ASEVKIAC FGLAFKPNID DLRESPAVEV

501 550

StrPyoUGD IDILKSKD.. IKIIIYEPML NKL....... .......... .........E

HymAerUGD1 IEKLLAEG.. CSVSAYD... .......... .......... ..........

MetCapUGD1 MEALWRAG.. AKVRAFDPVA QEEAHRIYG. .......... .........D

ActGloUGD6 ATLLLARG.. ARVRLHDPIA GERFRREQ.. .......... .........P

HalZhaHYPO MESLWEAG.. AQVYAYDPEA MPETRHIYG. .......... .........D

MCIThaHYPO IRRLIKLD.. AIVTIHDPRA LDNARKI... .......... .........F

SalPacUGD AVRLHAAG.. AQVTAYDPQG LAMARQ.... .......... .........S

NatGarNSD2 IDELQEYD.. IDVAGFDPFA DDEA...... .......... .........A

BacSubUGD2 MERLAELG.. CDVHAYDPEA VLPEH..... .......... .........L

AzoTolUGD MKRIKAKG.. VEVIVYEP.. .......... .......... ..........

LacCreEPS MKKIKAKG.. ATVVIYEPTL ESGS...... .......... .........T

AlaProUG MKRIKAKG.. IKVVVYEPSF NEA....... .......... .........L

StrZooUG IDIINDYG.. VNIVIYEPML G......... .......... .........E

StrHGBNSD ARQLASLG.. AELRAVDPLI APAD...... .......... .........V

NatGarNSD IAELTARG.. ADVVAYD... .......... .......... ..........

TheMelUGD MDILKEKG.. YEIIIYEPVI KEK....... .......... .........E

SulDenUGD MKRIKAKG.. IEVVIYEPVL EES....... .......... .........E

SphAlaUGD MKRVKAKG.. IEVIVYEPLV AED....... .......... .........R

SheOneUGD MKRIKAKG.. IEVVVYEPVL KES....... .......... .........E

SalSerUGD MKRIKAKG.. VEVIIYEPVM EED....... .......... .........T

PseHalUGD MKRIAASN.. VQMVVYEPEL KED....... .......... .........V

ProMirUGD MKRIKAKG.. IEVVIYEPEM KEE....... .......... .........T

ParDisUGD MKRIKAKG.. AEVIIYEPTL EDGS...... .......... .........T

ParDisUGD1 MKRIKAKG.. AEVIIYEPTL EDGS...... .......... .........T

LacSalUGD MKRVRAKG.. AQIIIYEPTL DGNT...... .......... .........T

CloPerUGD MKRLKAKG.. ATVIIYEPTL ENGT...... .......... .........T

ArcButUGD MKRIKAKG.. IEVVVYEPVL NED....... .......... .........T

AerHydUGD MKRIKAKG.. IEVVIYEPTL SEP....... .......... .........E

LacJohSBP MKRVKAKG.. AKIIIFEPTL ENGS...... .......... .........T

StrUbeUGD IDHIKSYQ.. INIVLYEPMM N......... .......... .........E

ReiMEDNSD MKRLKAKG.. IEVVVYEPVL TEH....... .......... .........E

ExiSibNSD MKRIKAKG.. IEVTVFEPVL TED....... .......... .........T

RumObeHYPO MKRIKAKG.. ASVIIYEPTL KDGS...... .......... .........T

RumObeHYP1 MKRIKAKG.. ASVIIYEPTL EDGS...... .......... .........T

FaePraHYPO MKRIKAKG.. ATVIIYEPTL ENGS...... .......... .........T

EubDolHYPO MKRIKAKG.. AKVIIFEPTL KDGE...... .......... .........T

BacPleHYPO MKRIKAKG.. ATVIIYEPTL EDGT...... .......... .........T

BacCopHYPO MKRIKAKG.. ATIIIYEPTL EDGS...... .......... .........T

StrTheESP MKRIKAKG.. ATVIIYEPTL KDGE...... .......... .........K

StrIniCPS MKRIKAKG.. AKVIIFEPSL DDGS...... .......... .........T

SerProUGD MEQLWEAG.. ATVQAYDPEA MNEVQRIYG. .......... .........Q

GeoMetUGD ISKLLEMG.. ATVLAHDPEA VKEAKKIF.. .......... .........G

GeoUraUGD INRLLALG.. AKVCAHDPEA VKEAKKI... .......... .........F

AnoFlaUGD ARELVAEQ.. AIVVAYDPIA MNKAKSV... .......... .........L

PorGinNSD IEKLLEVG.. CRVRVYDPVA MKEAQKR... .......... .........L

BacSelRPON VPLLLEQG.. ADIYAYDPVG ADNFAKVHPE GKN....... .........G

PelPhaNSD IEELLSDG.. ARVRVYDPVA MDEVRRIYG. .......... .........E

MetPopNSD IAGLQDAG.. ARIVAYDPEG MEQARP.... .......... .........L

MetExtNSD IAGLQDAG.. AQIVAYDPEG MEQARP.... .......... .........L

VibVulPRE MKRIKAKG.. IEVVVYEPVL KEA....... .......... .........E

GeoKauNSD IERLIAEG.. ADVRVWDPAA LGHVSRR... .......... .........F

RhiTriEXO IQTLQDNG.. AQVVGYDPEG METTRK.... .......... .........V

ParDisUGD2 MKRVKAKG.. ATVIVYEPAL SDNT...... .......... .........T

XanAxoUGD MKRLKAKG.. VDVIVYEPTL KDP....... .......... .........E

XanCamUGD MKRLKAKG.. VDVIVYEPTL SDP....... .......... .........E

EntFaeUGD MKRVKAKG.. TTVVIYEPSL KDGE...... .......... .........I

StrPneUGD2 MKRLKAKG.. VTVIIYEPTL KDGE...... .......... .........T

StrPneUGD3 MKRIKAKG.. VRVVIYEPTL ESGS...... .......... .........T

StrPneUG MKRIKAKG.. VRVVIYEPTL ESGS...... .......... .........T

EscAlbUGD MKRIKAKG.. VEVIIYEPVM KEE....... .......... .........S

BacTheUGD MKRIKAKG.. ATIVIFEPTM QDGE...... .......... .........T

SacSpiUGD AE........ .......... .......... .......... ..........

Aer159PBP IKELKEFN.. MSVDVYD... .......... .......... ..........

ButSynUGD MKRVKAKG.. ARVIVHEPTL .......... .......... ..........

BraJapUGD .......... .......... .......... .......... ..........

SynCC9UGD CRDLLEEG.. AQLAIHDPKV VARQMTRDLQ QEAAPQADAL S........A

StePneUGD MERLDNYG.. KEIVIYEPTI .......... .......... ..........

SalAreUGD VVALRENG.. FPVRVFDPLA DPAALL.... .......... .........D

BurYI2NSD IADLKEYG.. VQVDVYDPWV SKE....... .......... .........E

BacCerNSD IPLLLEQG.. ADIYAFDPVG ANNFAKVYPE GRN....... .........K

PaeSanHYPO IRLLMAAG.. AQVEYHDPLV PNLQ...... .......... ..........

Rhi42MUGD IQALLDGG.. ANVHAYDPEG MEMAKE.... .......... .........V

OpiTAV5NSD VRDLLEEQ.. ANVVVYDPKV PAEKIRIDVL GSPETGDRKP .........E

MarHTCUGD8 ADALLDEK.. AEIVVYDPKV PAERIYADLD YLDTRSPEE. .........N

BacSubUGD ADRLAALD.. ARMKAYD... .......... .......... ..........

NocKunUGD ARRLA..... .......... .......... .......... ..........

DesHafUGD MELLRKNG.. ANIAYHDPYI PVIEPH.... .......... ..........

BacThuUGD VKYLTDLDV. GEVKVVEPHI NSLPKDL... .......... ..........

SphJapUGD AARLARRYG. RRIKLVEPYA HALPMEFVG. .......... ..........

RicSibUGD VKIL...... .......... .......... .......... ..........

MetCapUGD MEALWRAG.. AKVRAFDPVA QEEAHRIYG. .......... .........D

HymAerUGD IEKLLAEG.. CSVSAYD... .......... .......... ..........

BacSubUGD7 ADRLAALD.. ARMKAYD... .......... .......... ..........

ActGloUDG9 ATLLLARG.. ARVRLHDPIA GERFRRE... .......... .........Q

NatGarNSD5 IAELTARG.. ADVVAYD... .......... .......... ..........

UncBacHYPO IEFFLKHPE. YNVIVYDALA TNNIKDC... .......... .........F

HalZhaHYPP MESLWEAG.. AQVYAYDPEA MPETRHIYG. .......... .........D

StrPneUGD1 MKRIKAKG.. VRVVIYEPTL ESGS...... .......... .........T

HoePhoUGD IQALLDKG.. AIVKAHDPAG MDAARS.... .......... .........A

CapSpuUGD IKELVKRG.. AKVQAYDPKA VHEAKVCYL. .......... .........K

NovAroUGD VQTLLDAG.. AEVVAYDPEG MEAAAA.... .......... .........I

ProMarUGD CRNLINEG.. AFLSINDEKV TEEDIEKSLK KDNFVYGFNK .........N

HomSapUGD SKYLMDEG.. AHLHIYDPKV PREQIVVDLS HPGVSEDDQ. .........V

MusMusUNA SKYLMDEG.. AHLHIYDPKV PREQVVVDLS HPGVSADDQ. .........V

DanRerUGD SKYLMDEG.. AKLHIYDPKV LKEQIIQDLS QPGISGDNPE R........V

XenLaeUGD SKYLMDEG.. AKLHIYDPKV PREQIITDLS QPGVAADDR. .........V

RatNorUGD SKYLMDEG.. AHLHIYDPKV PREQIVVDLS HPGVSADDQ. .........V

PonAbeUGD SKYLMDEG.. AHLHIYDPKV PREQIVVDLS HPGVSEDDQ. .........V

BosTauUGD3 SKYLMDEG.. AHLHIYDPKV PREQIVVDLS HPGVSKDDQ. .........V

BamOldUGD CKGLLGDK.. AKISIYDPQV TEDQIQRDLA MNKFDWDHPI HLQPMSPT.A

PedCorUGD CRTLLAEG.. ARLNIYDPKV ESKQIMEDLK ISLTTQETKN .........E

ZeaMayUGD8 CKGLLGDK.. AQISIYDPQV TEDQIQRDLA MNKFDWDHPM HLQPTSPT.A

StrPurUGDP SKFLMDEG.. AKLSIYDPQV APAQILCELT NPSISADPER .........V

CioIntUGDP AKYLMDEG.. ANLKIYDPKV PSKQIMVELE HPTISECPEK .........A

NemVecPPRE CKYLLDEG.. AKLTIYDPKV EKDQIKLELE HPAITGDAQK .........V

CaeEleUGD IKHLMEEH.. AKLSVYDPKV QKSQMLNDLA SVTSAQD... .........V

PopTomUGD CQGLLGDK.. ALLSIYDPQV QKEHIQRDLI MKKFDWDHPL HLQPKSASSA

DroMelUGD CQTLLEEG.. AALDIYDPKV EPEQIIDDLT HPSVTESPEK .........V

AraThaUGD CKGLLEDK.. ARLSIYDPQV TEDQIQRDLS MNKFDWDHPL HLQPMSPT.T

DroMelUGD1 CQTLLEEG.. AALDIYDPKV EPEQIIDDLT HPSVTESPEK .........V

NasVitUDG AKTL...... .......... .......... .......... ..........

CaeBriHYPO MKHLMEEH.. AKLSVYDPKV QKSQMINDLA AVTSADD... .........V

DroWilGK3 CQTLLEEG.. AKLDIYDPKV EPEQIIDDLT HPSVTESPEN .........V

NasVitUGDP CQTLLEEG.. AALDIYDPKV EPEQIIDDLT HPSVTESPEK .........V

DroVirGJ3 CQTLLEEG.. AKLDIYDPKV EPEQIIDDLT HPSVTESPEN .........V

HomSapUGD2 SKYLMDEG.. AHLHIYDPKV PREQIVVDLS HPGVSEDDQ. .........V

XenTroUGD CKYLMDEG.. AHLAVYDPKV KKEQIIRDLS HPAISGDNPE R........V

FlaCF1UGD ADDLINEQ.. AKISVYDPKV SRNKILSDLD YLETRNSSD. .........N

ZeaMayUGD CKGLLGDK.. AQISIYDPQV TEDQIQRDLA MNKFDWDRPM HLQPTSPT.A

MayZebUGD1 SKYLMDEG.. AKLFIYDPKV LKEQIIHDLS QPSISEDNPE R........V

VitvinUGD1 CKGLLGDK.. AHLSIYDPQV SGEQIQRDLA MKKFDWDHPI HLQPLSPT.S

HomSapUGD1 SKYLMDEG.. AHLHIYDPKV PREQIVVDLS HPGVSEDDQ. .........V

OviAriUGD1 SKYLMDEG.. AHLHIYDPKV PREQIVVDLS HPGVSKDDQ. .........V

FelCatUGD1 SKYLMDEG.. AHLHIYDPKV PREQIVVDLS HPGVSEDDQ. .........V

EquCabUGD1 SKYLMDEG.. AHLHIYDPKV PREQIVVDLS HPGVSEDDQ. .........V

CanFamUGD1 SKYLMDEG.. AHLHIYDPKV PREQIVVDLS HPGVSQDDQ. .........V

DroMojGI7 CQTLLEEG.. AKLDIYDPKV EPEQIIDDLT HPSVTESPEN .........V

DroPseGA34 CQTLLEEG.. AKLDIYDPKV EPEQIIDDLT HPSVTESPEN .........V

HomSapICRA SKYLMDEG.. AHLHIYDPKV PREQIVVDLS HPGVSEDDQ. .........V

ColLivUGD SKYLMDEG.. AKLHIYDPKV PKEQIILDLS HPGVSEDNQ. .........V

CamFloUGD AKTLLDEG.. AVLHIYDPKV EETQIIQDLT HPSVTSNPED .........V

BosTauUGD SKYLMDEG.. AHLHIYDPKV PREQIVVDLS HPGVSKDDQ. .........V

MelGal1UGD SKYLMDEG.. AKLHIYDPKV PKEQIILDLS HPGVSEDNQ. .........V

AilMelUGDP SKYLMDEG.. AHLHIYDPKV PREQIVVDLS HPGVSQDDQ. .........V

MusFurUGDP SKYLMDEG.. AHLHIYDPKV PREQIVVDLS HPGVSQDDQ. .........V

MusFur1UGD SKYLMDEG.. AHLHIYDPKV PREQIVVDLS HPGVSQDDQ. .........V

MelUnd1UGD SKYLMDEG.. AKLHIYDPKV PKEQIILDLS HLGVSEDNQ. .........V

FalPer1UGD SKYLMDEG.. AKLHIYDPKV PKEQIVLDLS HPGVSEDNQ. .........V

ChrBel2UGD SKYLMDEG.. AKLHIYDPKV PREQIILDLS HPGVSEDDQ. .........V

FelCat1UGD SKYLMDEG.. AHLHIYDPKV PREQIVVDLS HPGVSEDDQ. .........V

SaiBolUGDP SKYLMDEG.. AHLHIYDPKV PREQIVVDLS HPGVSEDDQ. .........V

DroWilGK1 CQTLLEEG.. AKLDIYDPKV EPEQIIDDLT HPSVTESPEN .........V

CaeRemQV40 IKHLMEEH.. AKLSIYDPKV QKSQMLNDLA SVTSADD... .........V

CaeBriQV40 MKHLMEEH.. AKLSVYDPKV QKSQMINDLA AVTSADD... .........V

ZeaMayUGDI CKGLLGDK.. AQISIYDPQV TEDQIQRDLA MNKFDWDHPM HLQPTSPT.A

GalGalUG SKYLMDEG.. AKLHIYDPKV PKEQIILDLS HPGVSEDNQ. .........V

CaeEleUG IKHLMEEH.. AKLSVYDPKV QKSQMLNDLA SVTSAQD... .........V

PseAerGMD AEMLIGKG.. YELRIFDRNV EYARVHGANK EYIESK.... .........I

CanCloGM IEDLIRQD.. LEVLVYDNFV SNALQFGANK EYIDKIL... .........P

VibCraGMD VQEIASLH.. .......... .......... .......... ..........

PseSyrGMD AEMLIGKG.. FDLSIFDSNV EYARVHGANK DYIESKI... .........P

AmyAzuGMD AERLLGKG.. YDLRIYDANV SLSRLMGANR EYIEGRL... .........P

OxaAB1GMD8 IEHLLGKG.. YELKLYDKNV NLAALTGANQ DYILNHI... .........P

ActGloGDM AERLLGKG.. YDLKIYDSNV ALSRLVGANR EHIEGRL... .........P

AmyAlbGDM7 AERLLGKG.. YDLRIYDANV SLSRLMGANR EYIEGRL... .........P

AmyBalGDM AEKLLGKG.. YDLKIYDANV SLSRLMGANR EFIEGRL... .........P

PseDC3GMD5 AEMLIGKG.. FDLSIFDSNV EYARVHGANK DYIESKI... .........P

PseVerGMD1 AEMLIGKG.. YDLSIYDSNV EYARVHGANK DYIEGKI... .........P

PseQDAGMD1 AERLIGKG.. YQLDIFDENV QYARMHGANK DYIESKI... .........P

PseAerGMD1 AEMLIGKG.. YELRIFDRNV EYARVHGANK EYIESKI... .........P

PseHYSGMD1 AEMLIGKG.. YELSIYDANV EYARVFGANR EYIESKI... .........P

AmyAlbGMD6 AERLLGKG.. YDLRIYDANV SLSRLMGANR EYIEGRL... .........P

AmyAzuGMD1 AERLLGKG.. YDLRIYDANV SLSRLMGANR EYIEGRL... .........P

AlgSagHYPO AKRLYQSD.. YELAIIDRNV RQSLLKAPDG VIAEQL.... .........G

AciKBSHYPO LEQLIGKG.. REVRVFDPHI QLDAIYGSNR NFILQQI... .........P

AmyBalGMD AEKLLGKG.. YDLKIYDANV SLSRLMGANR EFIEGRL... .........P

StrCanGMD1 AERLHGKG.. YDLRIHDANV SLSRLIGANR EYIETRL... .........P

PseAlcGMD1 AEMLIGKG.. YELRIFDSNV EYARVFGANK EYIESKI... .........P

ActGloGMD6 AERLLGKG.. YDLKIYDSNV ALSRLVGANR EHIEGRL... .........P

OxaAB1GMD1 IEHLLGKG.. YELKLYDKNV NLAALTGANQ DYILNHI... .........P

PseAerUNPP AEMLIGKG.. YEFRIFDRNV EYARVHGANK EYIESKI... .........P

HahGanHYPO AETLIGKG.. YSLQIFDRNV EYARVHGANK EYINSHI... .........P

DesSalNSD IEQLIGKG.. FELKLYDRNV NAAKLLGANR DYIMNRI... .........P

PseTomGMD1 AEMLIGKG.. FELSIFDSNV EYARVHGANK DYIESKI... .........P

MarAlgGMD AEMLIGKG.. YDLQIFDRNV DYARTHGANR EYINQKI... .........P

PseSyrGMD3 AEMLIGKG.. FDLSIFDSNV EYARVHGANK DYIESKI... .........P

NovAroGMD ARKLLDAG.. YALDIYDPQL RPESLVGQNL GYAYAIL... .........P

AzoSpGMD AEQLIGKG.. MQLSIYDPDV QLSRLLGANR RFIETQL... .........P

AzoVinGMD AEMLIGKG.. FKLSIFDSNV EYARDHGANG HYIKNEI... .........P

BacThuGMD VELLRKEG.. YEVAVYDPHV VQ........ .......... ..........

HahGanHYPP AETLIGKG.. YSLQIFDRNV EYARVHGANK EYINSHI... .........P

AlgSagHYPP AKRLYQSD.. YELAIIDRNV RQSLLKAPDG VIAEQL.... .........G

AciKBSHYPP LEQLIGKG.. REVRVFDPHI QLDAIYGSNR NFILQQI... .........P

PseSyrGMD2 AEMLIGKG.. FDLSIFDSNV EYARVHGANK DYIESKI... .........P

PseSyrGMD1 AEMLIGKG.. FDLSIFDSNV EYARVHGANK DYIESKI... .........P

StrCanGMD AERLHGKG.. YDLRIHDANV SLSRLIGANR EYIETRL... .........P

PseVerGMD AEMLIGKG.. YDLSIYDSNV EYARVHGANK DYIEGKI... .........P

PseQDAGMD AERLIGKG.. YQLDIFDENV QYARMHGANK DYIESKI... .........P

PseHYSGMD AEMLIGKG.. YELSIYDANV EYARVFGANR EYIESKI... .........P

PseAlcGMD AEMLIGKG.. YELRIFDSNV EYARVFGANK EYIESKI... .........P

PseAerUPP AEMLIGKG.. YEFRIFDRNV EYARVHGANK EYIESKI... .........P

StaAurUMD YELLNQEPD. IEVCAYDPHV ELDFVEHDMS HAVKDAS... .........L

EscColUMND AELIAQWHS. GETLVVEPNI HQLPKKLTGL WYSGAA.... ..........

LusFleUGD MESLRKKY.. .......... .......... .......... ..........

SalEntUMD AQSIARWHS. GETLVVEPNI RQLPKKLDGL .......... ..........

ActSerUG TQYLADWHR. GVVLAVEPHI SALPP..... .......... .........S

ErwTasUG TGMIAAWHQ. GTTLVVEPNV QQLPA..... .......... .........D

FusUlcUMD AEILRDKG.. YEVIACEPNV NK........ .......... ..........

CorMarUMD AEAIAERFPD VSVLAVEPNA AELPASL... .......... ..........

StaVitUMD YNDLRKEENL IIE....... .......... .......... ..........

SheVioUMD TKALAIKGY. .NILAVEPNI DELPAKFSSL .......... ..........

SerS4UMD AHLIADWHVG .ETLVVEPNV EQLPKSLVG. .......... ..........

PseStuUMD TKHIASTHPG .KVLAIEPNI EYLPVAL... .......... ..........

PsePseUMD ASKLIDKHPG .QVVLVEPNI EKLPKSLEG. .......... ..........

PseFluUMD TQKILAIHPG .PVVTVEPNI ETLPKLLAG. .......... ..........

HalSalUMD LRL....... .......... .......... .......... ..........

FraPhiUMD TKMLSDIDGV .EILAVEPNI KELPAVL... .......... ..........

EdwC07UMD AEMVAQWHS. GTTLVVEPNI HQLPARLAGM .......... ..........

AerAquUMD TKTLANNPA. YQILAVEPNI EALPATL... .......... ..........

GeoWSUUMD VELLEQYG.. AEFAVVDPYV .......... .......... ..........

EscMG1UMD AELIAQWHS. GETLVVEPNI HQLPKKLTGL WYSGAA.... ..........

DicZeaUMD AEQI...... .......... .......... .......... ..........

VibOrdUMD VKELASFHR. GKVMVVEPNI QQLP...... .......... .........E

VidVulUMaD AKKVSEFHR. GKVLVVEPNI AQLP...... .......... .........S

KlePneUMaD AAQIARWHS. GTTQVVEPNI HALPK..... .......... .........K

GeoTheUMaD YEELRRNER. FDVRAYDPHV K......... .......... .........P

BurXenUMaD VNQLADEKV. ADIVVVEPNV SVLPD..... .......... .........A

HasParUMaD TEYFADWHQ. GKLWLVEPHI AQLP...... .......... .........A

RalSolUMaD VQTMVQQQL. GTVLVVEPHI KVLP...... .......... .........A

PolIrgUMaD AARIISEAR. AQVLVVEPNI .......... .......... .........E

RalSolNGD VRTMVQQQL. GTVLVVEPHI KVLP...... .......... .........A

YerEntUMaD AHLIAQWHT. GETLVVEPNV EQLPK..... .......... .........S

EscFerUMaD AELIAQWHS. GETLVVEPNI HQLPN..... .......... .........K

OceGraUMaD AHKLSRTHA. GPLLVVEPNC TEVP...... .......... .........S

BacNRRUMaD IKELEKEG.. AKVTYFDPYI PEYNDH.... .......... .........G

GeoKauUMaD VELLEQYG.. AEFAVVDPYV PSFRA..... .......... .........C

PsyCNPUMaD IKNLVMSG.. FDILAVEPNI DVLPTKL... .......... .........C

MorPE3UMaD TKNIAKMHC. GQVISIEPNI NMLP...... .......... .........S

SerProUMaD AHLIADWHV. GETLVVEPNV EQLPK..... .......... .........S

551 600

StrPyoUGD SEDQSVLVND LENFKKQANI IVTNRYDNEL Q.DVKNKVYS RDIFGRD...

HymAerUGD1 .......... .......... .......... .......... ..........

MetCapUGD1 RADLILCDSP ESALRGADAL AVVTEWNVFR S.PDFDEIKQ SL........

ActGloUGD6 ELAPYLSDTL DGLFDDCDAV VLVTEWAQYL E.LDWAKFVG LMRTPILLDG

HalZhaHYPO RDDLTLCERM EEAIEGVDAL VICTEWKQFR A.VNLHGVSK AMKTPVVIDG

MCIThaHYPO HDNIKYANSI LSSLKNSQCA IIMTKWKEYE G.INNKTTKY MDKKFIIDTR

SalPacUGD APQLSYAATA AEAAIDAELI VVGTGWPEFG A.LDPHLVAD GVARR.VLLD

NatGarNSD2 RESFGIEIQD RLSFEGFDAV VLATPHEEFD H.LDLGTVAD DLADEPALID

BacSubUGD2 RQHITQHSQA FDAIEESDFL FLATEWSEFL A.FDWKKAAD IMKGR.LVID

AzoTolUGD .......... .......... .......... .......... ..........

LacCreEPS FFGSKVIGDL DEFKRVSQAI ITNRYDSCLD D.VTDKVYTR DIFRRD....

AlaProUG FYGSKVIKSL DEFTSVSEII LANRYSEDLI E.VKEKLFSR DIFGEN....

StrZooUG DIGYRVVKDL EQFKNESTII VSNRFEDDLG D.VIDKVYTR DVFGRD....

StrHGBNSD PEGITMVPCD DAQLASADLV VVLTDHD... .......... ..........

NatGarNSD .......... .......... .......... .......... ..........

TheMelUGD FEGIKVEKNL EKFKKISDVI LANRMFKDLE D.VEGKVYTR DLFNRD....

SulDenUGD FFNSRVIKDL DEFKAISDVV VANRLNENIM D.IKEKVYTR DIFNSDS...

SphAlaUGD LFNSRVIRDL DAFKAEADVI IANRITDDLA D.VVDKVYSR DLFGADS...

SheOneUGD FFKSTVIRNL EEFKAMCDVI VTNRMTSELE D.VADKVYTR DLFHHD....

SalSerUGD FFNSRLERDL HCFKQQADVI ISNRMAAELL D.VAEKVYTR DLFGSD....

PseHalUGD FYNSRVIRNL NEFKQISDVV VSNRMVEELS D.ITDKVYTR DLFGSD....

ProMirUGD FFNSKLIHNL DEFKAMSDVI ITNRMASALN D.VEEKVYTR DLFGND....

ParDisUGD FFGSKVVNDL NTFKRQCQAI IANRYDACLD D.VNGKVYSR DVFRRD....

ParDisUGD1 FFGSKVVNDL TAFKAQSQAI IANRYDNCLD D.IKELVYTR DIFKRD....

LacSalUGD FFGSQIINDL ERFKQMSDVI IANRYEKSLD D.VEEKVYTR DIFKRD....

CloPerUGD FFGSKVVNEI DEFKRLSKSI IANRYDSCLD D.IKEKVYTR DLFQRD....

ArcButUGD FFNSRVIKDL SEFKKISDVI VANRLSDILK D.VQDKVYTR DIFGND....

AerHydUGD FFRSRVLSDI VEFKESCDVI LANRMVEELF D.VMDKVYTR DLFGSD....

LacJohSBP FFGSEVVNDL DEFKNKSDAI IANRYNTILD D.VKDKVYTR DIFRKD....

StrUbeUGD DFDLPIIDDL SDFKAMSHII VSNRYDLALE D.VKEKVYTR DIYGVD....

ReiMEDNSD FFRSLVINDL EKFKQVSDVI VANRPSDELA D.VMDKVYTR DLFGSD....

ExiSibNSD FYNSRVIRDL EAFKQESDII LSNRMHSELS D.VEDKVYTR DLYSRD....

RumObeHYPO FFGSKVVNDL DKFKNLSDCI IANRYSQILD D.VKDKVYTR DLFQRD....

RumObeHYP1 FFGSKVVNDL DKFKEQSQAI IANRYDSCLD D.VQDKVYTR DIFKRD....

FaePraHYPO FFGSEVVNDL AEFKKRSQAI IANRYDSCLD D.VQEKVYTR DLFRRD....

EubDolHYPO FFGSEIVNDL DSFKKQSDAI IANRYDECLN D.IEDKVYTR DLFRRD....

BacPleHYPO FFGSRVVNNL EVFKQQANAI IANRYDTCLD D.VKDKVYTR DIFRRD....

BacCopHYPO FFGSKVVNRL EDFKAQAQAI IANRYDACLD D.VKEKVYTR DIFRRD....

StrTheESP FFGSVVVNDL DEFKKKSQAI IANRYNKCLD D.VKEKVYTR DIFQRD....

StrIniCPS FFGSQVVNDL ETFKKTSQSI IANRYDSALD D.VQEKVYTR DLFGRD....

SerProUGD RDDLKLVGTK EAALQGADAL VICTEWQNFR A.PDFDAIKG SLKQPVIFDG

GeoMetUGD DRITYTSTNQ YEILKGADAL AIITEWNEYR N.PDFERISA SLTAPVIFDG

GeoUraUGD GENIVYSNNQ YDILKGADAL AIVTEWNEYR N.PDFDRIKS LLRQPLIFDG

AnoFlaUGD PKEVIYASRV EEALKDADAA MILTEWDEFR Q.LDLSVYVK QMKTPIIFDG

PorGinNSD GDKVEYTTDM YDAVRGAEAL FHVTEWKEFR M.PDWSALSQ TMAASLVIDG

BacSelRPON KGSITYVSNI EDALNGANVC FIFTEWGEVK T.VTPKKYKE LMRTPLVYDG

PelPhaNSD HDDIFYASNP EEAIKGSDAL VVLTEWLVFR S.PDFEMIKR DLAHPVVFDG

MetPopNSD LQGVDYAEDA YACAEGADAL VIVTEWNAFR A.LDLARLKA MMAAPVLVDL

MetExtNSD LHGVAYAEDA YACAEGADAL VIVTEWNAFR A.LDLARLKG LMRAPVLVDL

VibVulPRE FFHSRVIKDL NEFKQTADVI VSNRMVEELT D.VADKVYTR DLFGSD....

GeoKauNSD GDAVVCCETM EEAIRGADVC FIFTEWPAVL Q.FDLHRYKT LMNRPIVLDG

RhiTriEXO IENIEYASGL YEAAAGADAL VIVTEWNQFR A.LDFNRLKQ SMRAP.ILVD

ParDisUGD2 FFGSEVVNDL DLFKKRSAAI IANRYDVELD D.VKNKVYTR DVYRRD....

XanAxoUGD FFRSRVVNDL QAFKREADVI ISNRMAEALQ D.VQDKVYTR DLFGDN....

XanCamUGD FFRSRVVNDL EAFKREADVI ISNRMADALQ D.VQDKVYTR DLFGDN....

EntFaeUGD FFGSKVVNDL DQFKAMCDSI IANRYDTCLD D.IKEKVYTR DIFQRD....

StrPneUGD2 FFGNKVVNNL DKFKEASNVI VANRFEPSLE D.VSNKVYSR DIFKRD....

StrPneUGD3 FFGSSIINDL EEFKRLSNAI VANRYDNSLE D.VKEKVYTR DIFERD....

StrPneUG FFGSSVINDL EEFKRLSNAI VANRYDNSLE D.VKEKVYTR DIFERD....

EscAlbUGD FFNSRLERDL VTFKQQADII ISNRMAEELK D.IADKVYTR DLFGSD....

BacTheUGD FFGSQVINNL VEFKEISQAI IANRYDACLD D.VKEKVYTR DIFQRD....

SacSpiUGD .......... .......... .......... .......... ..........

Aer159PBP .......... .......... .......... .......... ..........

ButSynUGD .......... .......... .......... .......... ..........

BraJapUGD .......... .......... .......... .......... ..........

SynCC9UGD TGSWAEACSV EEAVTGADAV LVLTEWQDYR N.LNWMSLAG RMRKPAWVFD

StePneUGD .......... .......... .......... .......... ..........

SalAreUGD RFGIPPAASL DEAVRGAGCL AFLAGHREFQ R.LDFAALAE LVDVPCLVFD

BurYI2NSD AHHEYGIDPI EPAKGVYDAI VLAVAHKQFA D.EGAEGIHA ..........

BacCerNSD NGNITYVTDI EQALEGANVC FIFTEWGEVK A.LTPEMYKK LMRTP.LIYD

PaeSanHYPO .......... .......... .......... .......... ..........

Rhi42MUGD IGPITYGRDP YEIAEGADAI VIVTEWDEFR A.LDFKRMKS LVKTP.TIVD

OpiTAV5NSD ASRLSVASSA YEASAGAHAV AILTEWDEFK T.LDFGKIHA SMQKPAFLFD

MarHTCUGD8 RKLLTVTYDP IKAVEEAHAI AILTEWDEFK T.YNWKSLYS KMLKPAFVFD

BacSubUGD .......... .......... .......... .......... ..........

NocKunUGD .......... .......... .......... .......... ..........

DesHafUGD .......... .......... .......... .......... ..........

BacThuUGD .......... .......... .......... .......... ..........

SphJapUGD .......... .......... .......... .......... ..........

RicSibUGD .......... .......... .......... .......... ..........

MetCapUGD RADLILCDSP ESALRGADAL AVVTEWNVFR S.PDFDEIKQ SL........

HymAerUGD .......... .......... .......... .......... ..........

BacSubUGD7 .......... .......... .......... .......... ..........

ActGloUDG9 PELAPYLSDT LDGLFDDCDA VVLVTEWAQY LELDWAKFVG LMRTPILLDG

NatGarNSD5 .......... .......... .......... .......... ..........

UncBacHYPO KEQIQYASSI EECFLKANVV LIMTEDPAFK E.IDQRYFSN KSIV......

HalZhaHYPP RDDLTLCERM EEAIEGVDAL VICTEWKQFR A.VNLHGVSK AMKTPVVIDG

StrPneUGD1 FFGSSIINDL EEFKRLSNAI VANRYDNSLE D.VKEKVYTR DIFERD....

HoePhoUGD MPEIEYSDSI QATARDSDAL VIITDWDDFK S.LDFEDLRE VMKSP.VLVD

CapSpuUGD DVEVTYVESK YEALKGADAL ILLTEWKEFR V.PDFDEIAK LLNEK.VIFD

NovAroUGD MPEVTMAPNA YAAIEGADAI VLVTEWDAFR A.LDFARIRR LANAP.VMVD

ProMarUGD EPCWEFQSNL YSAFENAHAV IILTSWDKYK A.IDWERVSL SVKSPFWVFD

HomSapUGD SRLVTISKDP YEACDGAHAV VICTEWDMFK E.LDYERIHK KMLKPAFIFD

MusMusUNA SRLVTISKDP YEACDGAHAL VICTEWDMFK E.LDYERIHK KMLKPAFIFD

DanRerUGD SDLVTVTVDP YEACESAHAL VICTEWDMFK D.LDYEKIYH KMLKPAFIFD

XenLaeUGD SQLVHISTDL YEACENAHAM VICTEWDMFK E.LDFNRIHR MMLKPAFIFD

RatNorUGD SRLVTISKDP YEACDGAHAL VICTEWDMFK E.LDYERIHK RMLKPAFIFD

PonAbeUGD SRLVTISKDP YEACDGAHAV VICTEWDMFK E.LDYERIHK KMLKPAFIFD

BosTauUGD3 ARLVTISKDP YEACDGAHAV VICTEWDMFK E.LDYERIHK KMLKPAFIFD

BamOldUGD VKEVSVTWDA YEATKGAHGV CILTEWDEFK T.LDYKKIYD NMQKPAFLFD

PedCorUGD EKSIMIFDDP YEATAKTHAI VLCTEWDEFI Y.LDYEKIFN EMMKPAHIFD

ZeaMayUGD8 VKQVSCVWDA YEATKGAHGL CILTEWDEFK T.LDYQKIFD NMQKPAFVFD

StrPurUGDP EKLVTIHSDP YEALKGTHAF VVCTEWDEFK D.YDYLRIYK DMLKPAFAFD

CioIntUGDP RELIDVCDDP YVACQGAHAI AVCTEWDMFK T.LDFEKIFN SMLKPAFIFD

NemVecPPRE DRLVTIEHDP YKAVEGAHAI VICTEWDEFK T.YDYQKIHD SMLKPAFVFD

CaeEleUGD ERLITVESDP YAAARGAHAI VVLTEWDEFV E.LNYSQIHN DMQHPAAIFD

PopTomUGD VEQVTVTSDA YEATKEAHGV CILTEWDEFK T.LDYKKIYD NMQKPAFVFD

DroMelUGD KKAVQIHSDP YSAVRATHAL VICTEWDEFV D.LDFKRIYQ SMMKPAYIFD

AraThaUGD VKQVTVTWDA YEATKDAHGI CIMTEWDEFK N.LDFQKIFD NMQKPAFVFD

DroMelUGD1 KKAVQIHSDP YSAVRATHAL VICTEWDEFV D.LDFKRIYQ SMMKPAYIFD

NasVitUDG .......... .......... .......... .......... ..........

CaeBriHYPO TRLVTVETDP YAAARGAHAI VVLTEWDEFV D.LDYNKIHD NMQHPAAIFD

DroWilGK3 KKAVQIHSDP YSAVRSTHAL VVCTEWDEFV D.LDYTRIYQ SMMKPAYIFD

NasVitUGDP KKAVQIHSDP YSAVRATHAL VICTEWDEFV D.LDFKRIYQ SMMKPAYIFD

DroVirGJ3 KKAVQIHSDP YSAVRATHAL VLCTEWDEFV D.LDYKRIYQ SMMKPAYIFD

HomSapUGD2 SRLVTISKDP YEACDGAHAV VICTEWDMFK E.LDYERIHK KMLKPAFIFD

XenTroUGD SELVTITSEP YAACENTHAL VICTEWDLFK D.LDYERIYG KMLKPAFIFD

FlaCF1UGD VDAVQTFQDA YEACKGAHAV AILTEWDEFV K.YDWQKIYD SMHKPAFVFD

ZeaMayUGD IKQVSCVWDA YEATKGAHGV CILTEWDEFK T.LDYQKIFD NMQKPAFVFD

MayZebUGD1 SELVTVTSDP YEACQSAHAL VICTEWDMFK E.LDYDKIYK NMLKPAFMFD

VitvinUGD1 VKQVSVVWDA YTATKDAHGI CILTEWDEFK S.LDYKKIYD NMQKPAFVFD

HomSapUGD1 SRLVTISKDP YEACDGAHAV VICTEWDMFK E.LDYERIHK KMLKPAFIFD

OviAriUGD1 ARLVTISKDP YEACDGAHAV VICTEWDMFK E.LDYERIHK KMLKPAFIFD

FelCatUGD1 SRLVTISKDP YEACDGAHAV VICTEWDMFK E.LDYERIHK KMLKPAFIFD

EquCabUGD1 SRLVTISKDP YEACDGAHAV VICTEWDMFK E.LDYERIHK KMLKPAFIFD

CanFamUGD1 SRLVTISKDP YEACDGAHAV VICTEWDMFK E.LDYERIHK KMLKPAFIFD

DroMojGI7 KKAVQIHSDP YSAVRATHAL VLCTEWDEFV D.LDYKRIYQ SMMKPAYIFD

DroPseGA34 KKAVQIHSDP YSAVRATHAL VICTEWDEFV D.LDFQRIYQ SMMKPAYIFD

HomSapICRA SRLVTISKDP YEACDGAHAV VICTEWDMFK E.LDYERIHK KMLKPAFIFD

ColLivUGD SRLVTISNDP YEACDGAHAL VICTEWDMFK V.KMLSSN.. ..........

CamFloUGD KNRISIYKDA YSATKNTHAI VLCTEWDEFI E.LNYIQIYA GMMKPAYIFD

BosTauUGD ARLVTISKDP YEACDGAHAV VICTEWDMFK E.LDYERIHK KMLKPAFIFD

MelGal1UGD SRLVTISQDP YEACDGAHAL VICTEWDMFK E.LDYERIHK KMLKPAFIFD

AilMelUGDP SRLVTISKDP YEACDGAHAV VICTEWDMFK E.LDYERIHK KMLKPAFIFD

MusFurUGDP SRLVTISKDP YEACDGAHAV VICTEWDMFK E.LDYERIHK KMLKPAFIFD

MusFur1UGD SRLVTISKDP YEACDGAHAV VICTEWDMFK E.LDYERIHK KMLKPAFIFD

MelUnd1UGD SRLVTISKDP YEACDGAHAL VICTEWDMFK E.LDYERIHK KMLKPAFIFD

FalPer1UGD SRLVTISKDP YEACDGAHAL VICTEWDMFK E.LDYERIHK KMLKPAFIFD

ChrBel2UGD SRLVTISKDP YEACDGAHAL VICTEWDMFK E.LDYERIHK KMLKPAFIFD

FelCat1UGD SRLVTISKDP YEACDGAHAV VICTEWDMFK E.LDYERIHK KMLKPAFIFD

SaiBolUGDP SRLVTISKDP YEACDGAHAV VICTEWDMFK E.LDYERIHK KMLKPAFIFD

DroWilGK1 KKAVQIHSDP YSAVRSTHAL VVCTEWDEFV D.LDYTRIYQ SMMKPAYIFD

CaeRemQV40 SRLITVETDP YTAARGAHAI VVLTEWDEFV E.LNYTRIHD DMQHPAAIFD

CaeBriQV40 TRLVTVETDP YAAARGAHAI VVLTEWDEFV D.LDYNKIHD NMQHPAAIFD

ZeaMayUGDI VKQVSCVWDA YEATKGAHGL CILTEWDEFK T.LDYQKIFD NMQKPAFVFD

GalGalUG SRLVTISQDP YEACDGAHAL VICTEWDMFK E.LDYERIHK KMLKPAFIFD

CaeEleUG ERLITVESDP YAAARGAHAI VVLTEWDEFV E.LNYSQIHN DMQHPAAIFD

PseAerGMD PHVSSLLVSD LDEVVASSDV LVLGNGDELF VDLVNKTPSG KKLVDLVGFM

CanCloGM YLQNILVSTE DELIEWAELI IFNHKYPDYQ N.LIKIHTDK IFIDFIHISE

VibCraGMD .......... .......... .......... .......... ..........

PseSyrGMD HVSSLLNSDF DQVINDSDVI ILGNRDERFR S.LANKTPEG KRVIDLVGFM

AmyAzuGMD HLGQLLAGSI EEVADHAEVC LIGTVDPEVL A.ALPAGGGR IDLV......

OxaAB1GMD8 HISKLMVESM DEVLAFADTI VIGNGAAEFK T.VPGQLKPG QNLVDLVRIS

ActGloGDM HLSDLLTNDV AEVFAHAEVC VVGSTEPAVL E.ALAEPGDR DVVDLVRLPD

AmyAlbGDM7 HLGQLLAGSI DEVLDHAEVS LIGCNDPDVL A.AHPVGGGR TIIDLV....

AmyBalGDM HLGQLLAGSV EEVMDHADVV IVGCKDPDVL A.ALPRGGDR VLVDLVRLPD

PseDC3GMD5 HVSSLLNSDF DQVINDSDVI ILGNRDERFR S.LANKTPEG KRVIDLVGFM

PseVerGMD1 HVSSLLNSDF DEVINNCDVI ILGNRDEKFR A.LAHNAPHG KQVVDLVGFM

PseQDAGMD1 HVSSLLNADL QQVIDNADII VLGNRDEQFR T.LAQQAPAG KQVIDLVGFM

PseAerGMD1 HVSSLLVSDL DEVVASSDVL VLGNGDELFV D.LVNKTPSG KKLVDLVGFM

PseHYSGMD1 HVSSLLCNDL QQVIREAEVV VLGNNDDRFA Q.ALDASGGK QIIDLVGFMA

AmyAlbGMD6 HLGQLLAGSI DEVLDHAEVS LIGCNDPDVL A.AHPVGGGR TIIDLV....

AmyAzuGMD1 HLGQLLAGSI EEVADHAEVC LIGTVDPEVL A.ALPAGGGR TIIDLV....

AlgSagHYPO PLVSTLGEDL DEVIQSADVI VIGHAHPDFE S.VIAKVGSH QHIIDLVRIC

AciKBSHYPO HIGRLLDARL EDTLGWADHL VIAQKPDDGM R.AQIESSGL PVRSLIGAAL

AmyBalGMD HLGQLLAGSV EEVMDHADVV IVGCKDPDVL A.ALPRGGDR VLVDLVRLPD

StrCanGMD1 HLAQLLADSV EEVLDHAEVC LVGTKDPAVL A.ALPHGGGP VIVDLIRLPD

PseAlcGMD1 HVSSLLCKEL DEVVSQSDVL IIGNGEQRFA E.VMNSVGDD KQIVDLVGFM

ActGloGMD6 HLSDLLTNDV AEVFAHAEVC VVGSTEPAVL E.ALAEPGDR DVVDLVRLPD

OxaAB1GMD1 HISKLMVESM DEVLAFADTI VIGNGAAEFK T.VPGQLKPG QNLVDLVRIS

PseAerUNPP HVSSLLVSDL DEVVASSDVL VLGNGDELFV D.LVNKTPSG KKLVDLVGFM

HahGanHYPO HVSSLLTSDL NAAMEQADVV IIGNSDEIFE K.ALMNMPSD KKVLDLVGFM

DesSalNSD HISKLMVSDM ESVLDFAETI VVGNNSPEFK D.LQSKIRPG QVVVDLVRFD

PseTomGMD1 HVSSLLNSDF EQVINDSDVI ILGNRDERFR A.LANKTPEG KRVIDLVGFM

MarAlgGMD HLSNLMHSDL RGVIDHADVI VVGNNDELFE T.VISEVPEG KRVIDLVGFM

PseSyrGMD3 HVSSLLNSDF DQVINDSDVI ILGNRDERFR S.LANKTPEG KRVIDLVGFM

NovAroGMD SIDGLLVDKT TA.EARDYGV VIATNRLIRD ..LALPGRRI VDVSAIA...

AzoSpGMD HIGELLKPEL DAVIGEAEVL IVGVSSPAIF DALATHSRPE QKVLDLVRLP

AzoVinGMD HVSALLQSDL DKVVAEADVI VLGNADPRFE K.LAKDVPAG KKVIDLVGFM

BacThuGMD .......... .......... .......... .......... ..........

HahGanHYPP HVSSLLTSDL NAAMEQADVV IIGNSDEIFE K.ALMNMPSD KKVLDLVGFM

AlgSagHYPP PLVSTLGEDL DEVIQSADVI VIGHAHPDFE S.VIAKVGSH QHIIDLVRIC

AciKBSHYPP HIGRLLDARL EDTLGWADHL VIAQKPDDGM R.AQIESSGL PVRSLIGAAL

PseSyrGMD2 HVSSLLNSDF DQVINDSDVI ILGNRDERFR A.LANKTPEG KRVIDLVGFM

PseSyrGMD1 HVSSLLNSDF DQVINDSDVI ILGNRDERFR A.LANKTPEG KRVIDLVGFM

StrCanGMD HLAQLLADSV EEVLDHAEVC LVGTKDPAVL A.ALPHGGGP VIVDLIRLPD

PseVerGMD HVSSLLNSDF DEVINNCDVI ILGNRDEKFR A.LAHNAPHG KQVVDLVGFM

PseQDAGMD HVSSLLNADL QQVIDNADII VLGNRDEQFR T.LAQQAPAG KQVIDLVGFM

PseHYSGMD HVSSLLCNDL QQVIREAEVV VLGNNDDRFA Q.ALDASGGK QIIDLVGFMA

PseAlcGMD HVSSLLCKEL DEVVSQSDVL IIGNGEQRFA E.VMNSVGDD KQIVDLVGFM

PseAerUPP HVSSLLVSDL DEVVASSDVL VLGNGDELFV D.LVNKTPSG KKLVDLVGFM

StaAurUMD VLILSDHSEF KNLSDSHFDK MKHKVIFDTK N.VVKSSFED VLYYNYGNIF

EscColUMND .......... .......... .......... .......... ..........

LusFleUGD .......... .......... .......... .......... ..........

SalEntUMD .......... .......... .......... .......... ..........

ActSerUG LRGKVELVGF EQAIKKADIL LLLVDHTAFK N.LSPDTMSV PWLIDTKGIW

ErwTasUG LQEKVTLVTL EDALQQADVL VMLVDHRQFK A.VDPSVLTQ PWIVDTKGVW

FusUlcUMD .......... .......... .......... .......... ..........

CorMarUMD .......... .......... .......... .......... ..........

StaVitUMD .......... .......... .......... .......... ..........

SheVioUMD .......... .......... .......... .......... ..........

SerS4UMD .......... .......... .......... .......... ..........

PseStuUMD .......... .......... .......... .......... ..........

PsePseUMD .......... .......... .......... .......... ..........

PseFluUMD .......... .......... .......... .......... ..........

HalSalUMD .......... .......... .......... .......... ..........

FraPhiUMD .......... .......... .......... .......... ..........

EdwC07UMD .......... .......... .......... .......... ..........

AerAquUMD .......... .......... .......... .......... ..........

GeoWSUUMD .......... .......... .......... .......... ..........

EscMG1UMD .......... .......... .......... .......... ..........

DicZeaUMD .......... .......... .......... .......... ..........

VibOrdUMD ILNQCELQNF EVAVKGADVH VLLVDHKEF. .......... ..........

VidVulUMaD ELDGNVDLVD ISDTNYADLH VVLVGHKEFG S.NLFDKKNT IFAVNV....

KlePneUMaD LDGLCTLATL EAALASADVL VMLVDHNQFK A.VSGDSVTQ AFIVDTKGVW

GeoTheUMaD SQVPFPLSTK EEALDGAHLV VVLADHNEFK T.LTAEELAS MKTKVIFDTK

BurXenUMaD LRGKVELTSL ASAIEAADIV LLLVDHREFK D.INRRRLQS KLLVDTRGLF

HasParUMaD SLAEKVEWVD FAQGITADVV VFLVDHLAFQ Q.HAKRHLSP TWLVDTRGAW

RalSolUMaD ALQGVELLNA EAALSRADIV VLLVDHQQFR K.LDTDRLQS RVVIDTRGMW

PolIrgUMaD SHSSFNLIPY KKAYYKADIV VWLVSHNEFL E.MPITENKI ELDFCGVRK.

RalSolNGD SLEGVELLNA EAALSRADIV VLLVDHQQFR K.LDTDRLQS RVVIDTRGMW

YerEntUMaD LVGHVTLKDT ATALQQADVL VMLVDHSQFK A.IKPEDVKQ SWIVDTKGVW

EscFerUMaD LNGLCTLAQL DEALATADVL VMLVDHSQFK V.INGDNVHQ QYVVDAKGVW

OceGraUMaD GLGEAELVTM QEAFQRADIH VYLVAHREFQ A.SPRPPRYV IDTVGL....

BacNRRUMaD DIKRGENGLT AELIEKADLV MITTDHTM.. ..VDYSLVQE HAKAIFDTKN

GeoKauUMaD NRVIETVELT PELLAQSDLV LITTDHSN.. ..IDYEMVAR HSRVVFDTRN

PsyCNPUMaD DLGNIKLTDL HSALVDADLI VILVKHQEFI N.LKSGSKIL DFVNC.....

MorPE3UMaD ELSNVQLVCF DIASAEADIH ILLVDHNEFK T.KKPKSGVL IDTKGIWN..

SerProUMaD LVGHVTLKAL PEALQQADVI VMLVDHKQFK A.IKPEEITQ SWIVDTKGVW

601 650

StrPyoUGD .......... .......... .......... .......... ..........

HymAerUGD1 .......... .......... .......... .......... ..........

MetCapUGD1 .......... .......... .......... .......... ..........

ActGloUGD6 RHVLDADRMA RMGYKYLAIS G......... .......... ..........

HalZhaHYPO RNLFSPLAAK EAGITYVSVG R......... .......... ..........

MCIThaHYPO RILSN..... .......... .......... .......... ..........

SalPacUGD TRGVISGPRW RAAGWTVHGL G......... .......... ..........

NatGarNSD2 VTGAVDEDVA ADAGL..... .......... .......... ..........

BacSubUGD2 GRNVLKKELI EACGLICTGV G......... .......... ..........

AzoTolUGD .......... .......... .......... .......... ..........

LacCreEPS .......... .......... .......... .......... ..........

AlaProUG .......... .......... .......... .......... ..........

StrZooUG .......... .......... .......... .......... ..........

StrHGBNSD .......... .......... .......... .......... ..........

NatGarNSD .......... .......... .......... .......... ..........

TheMelUGD .......... .......... .......... .......... ..........

SulDenUGD .......... .......... .......... .......... ..........

SphAlaUGD .......... .......... .......... .......... ..........

SheOneUGD .......... .......... .......... .......... ..........

SalSerUGD .......... .......... .......... .......... ..........

PseHalUGD .......... .......... .......... .......... ..........

ProMirUGD .......... .......... .......... .......... ..........

ParDisUGD .......... .......... .......... .......... ..........

ParDisUGD1 .......... .......... .......... .......... ..........

LacSalUGD .......... .......... .......... .......... ..........

CloPerUGD .......... .......... .......... .......... ..........

ArcButUGD .......... .......... .......... .......... ..........

AerHydUGD .......... .......... .......... .......... ..........

LacJohSBP .......... .......... .......... .......... ..........

StrUbeUGD .......... .......... .......... .......... ..........

ReiMEDNSD .......... .......... .......... .......... ..........

ExiSibNSD .......... .......... .......... .......... ..........

RumObeHYPO .......... .......... .......... .......... ..........

RumObeHYP1 .......... .......... .......... .......... ..........

FaePraHYPO .......... .......... .......... .......... ..........

EubDolHYPO .......... .......... .......... .......... ..........

BacPleHYPO .......... .......... .......... .......... ..........

BacCopHYPO .......... .......... .......... .......... ..........

StrTheESP .......... .......... .......... .......... ..........

StrIniCPS .......... .......... .......... .......... ..........

SerProUGD RNLFDPERLE SRGFTYYAIG RGASIQPVI. .......... ..........

GeoMetUGD RNLYNPRRMK EIGFTYHSIG RNGSAFTG.. .......... ..........

GeoUraUGD RNLYQPSRMK EAGFEYLPIG RNGRHFVDI. .......... ..........

AnoFlaUGD RNCYALDAVK AYGIEYDSIG RKRVK..... .......... ..........

PorGinNSD RNVYELPADS DFTLLNIGNS AIESASSK.. .......... ..........

BacSelRPON RNIYSIDEML EAGVEYHSIG RKAVVKESRD LELQTVRS.. ..........

PelPhaNSD RNIYSPEFME QSGFTYYSIG RPPRGVS... .......... ..........

MetPopNSD RNVYAPDEAR RHGLRHVGVG ALASRD.... .......... ..........

MetExtNSD RNVYAPAETE RHGFAYSGIG VA........ .......... ..........

VibVulPRE .......... .......... .......... .......... ..........

GeoKauNSD RNCYDPKQAD AAGLIYESIG RPVGHRQLKV DRAVDVLQCA SAAFFHKQSE

RhiTriEXO LRNIYRSDEV RKYGFTYTGI GTNLYQDVTN T......... ..........

ParDisUGD2 .......... .......... .......... .......... ..........

XanAxoUGD .......... .......... .......... .......... ..........

XanCamUGD .......... .......... .......... .......... ..........

EntFaeUGD .......... .......... .......... .......... ..........

StrPneUGD2 .......... .......... .......... .......... ..........

StrPneUGD3 .......... .......... .......... .......... ..........

StrPneUG .......... .......... .......... .......... ..........

EscAlbUGD .......... .......... .......... .......... ..........

BacTheUGD .......... .......... .......... .......... ..........

SacSpiUGD .......... .......... .......... .......... ..........

Aer159PBP .......... .......... .......... .......... ..........

ButSynUGD .......... .......... .......... .......... ..........

BraJapUGD .......... .......... .......... .......... ..........

SynCC9UGD ARAITDHGQV RASGLNLWCV GDGEG..... .......... ..........

StePneUGD .......... .......... .......... .......... ..........

SalAreUGD GLLHLPPARV RELHQLGFAY RGIGR..... .......... ..........

BurYI2NSD .......... .......... .......... .......... ..........

BacCerNSD GRNIYDVQAM QEAGIEYHSI GRKSTNRDRM KELNNIELQA SR........

PaeSanHYPO .......... .......... .......... .......... ..........

Rhi42MUGD LRNIYPVAEV TKHGFSYFAI G......... .......... ..........

OpiTAV5NSD GRNIVDLEKL EKIGFRAYGL GK........ .......... ..........

MarHTCUGD8 GRRLMDKSQM EEIGFKYYKI GES....... .......... ..........

BacSubUGD .......... .......... .......... .......... ..........

NocKunUGD .......... .......... .......... .......... ..........

DesHafUGD .......... .......... .......... .......... ..........

BacThuUGD .......... .......... .......... .......... ..........

SphJapUGD .......... .......... .......... .......... ..........

RicSibUGD .......... .......... .......... .......... ..........

MetCapUGD .......... .......... .......... .......... ..........

HymAerUGD .......... .......... .......... .......... ..........

BacSubUGD7 .......... .......... .......... .......... ..........

ActGloUDG9 RHVLDADRMA RMGYKYLAIS G......... .......... ..........

NatGarNSD5 .......... .......... .......... .......... ..........

UncBacHYPO .......... .......... .......... .......... ..........

HalZhaHYPP RNLFSPLAAK EAGITYVSVG R......... .......... ..........

StrPneUGD1 .......... .......... .......... .......... ..........

HoePhoUGD LRNLYNPAQV RELGFTYHSI GRS....... .......... ..........

CapSpuUGD GRNQYNAFEL PQKGWEYIQI GV........ .......... ..........

NovAroUGD LRNVYDPAEV RAAGFEYTSV GRP....... .......... ..........

ProMarUGD TRSILDIKNI QDLGFNFWQL GFGNNN.... .......... ..........

HomSapUGD GRRVLDGLHN ELQTIGFQIE TIGKKVSSKR IPYAPSGEIP KFSLQDPPNK

MusMusUNA GRRVLDGLHS ELQTIGFQIE TIGKKVSSKR IPYTPGEIPK FSLQDPPNKK

DanRerUGD GRRVLDHLHT QLQNVGFQIE TIGKKVTTRI PFTTSGGVPR ITEPPVKKSK

XenLaeUGD GRRVLDELHG ELQNIGFQVE TIGKKVASKR IPFTPTADIP KFGLQDLPHK

RatNorUGD GRRVLDGLHN ELQTIGFQIE TIGKKVSSKR IPYTPGEIPK FSLQDPPNKK

PonAbeUGD GRRVLDGLHN ELQTIGFQIE TIGKKVSSKR IPYAPSGEIP KFSLQDPPNK

BosTauUGD3 GRRVLDGLHN ELQTIGFQIE TIGKKVSSKR IPYAPSGEIP KFSLQDMPNK

BamOldUGD GRNVIDPEKL REIGFIVYSI GKPLDPWLKD MPAMA..... ..........

PedCorUGD GRKILQHDKL IEIGFNVQTI GKSLNSNHLL NNW....... ..........

ZeaMayUGD8 GRNIVDSEKL REIGFIVYSI GKPLDAWLKD MPAVA..... ..........

StrPurUGDP GRRLLDSSLL ENLGFHVEVV GRKSWKNGIL PVTP...... ..........

CioIntUGDP GRGILPYEKL LDIGFEVNVL GKQFSGNSIF QAATSSDILL PSSRVLP...

NemVecPPRE GRMILDHHHL HDVGFQVETI GKVVSSGYVL PLTPPLPPCS D.........

CaeEleUGD GRLILDQKAL REIGFRTFAI GTSPDQAYNL FGTAGY.... ..........

PopTomUGD GRNVVNADKL REIGFIVYSI GKPLDAWLKD MPAIA..... ..........

DroMelUGD GRKILDHERL QQIGFHVQTI GKKYQRTGLL RSWGIVPQL. ..........

AraThaUGD GRNIMNLQKL REIGFIVYSI GKPLDDWLKD MPAVA..... ..........

DroMelUGD1 GRKILDHERL QQIGFHVQTI GKKYQRTGLL RSWGIVPQL. ..........

NasVitUDG .......... .......... .......... .......... ..........

CaeBriHYPO GRLILDQKAL REIGFRTFAI GTAPDQAYNL FGTAGY.... ..........

DroWilGK3 GRKILDHERL QQIGFHVQTI GKKYQRTGLL RSWGIVPQL. ..........

NasVitUGDP GRKILDHERL QQIGFHVQTI GKKYQRTGLL RSWGIVPQL. ..........

DroVirGJ3 GRKILDHERL LQIGFHVQTI GKKYQRAGLL RSWGIVPQL. ..........

HomSapUGD2 GRRVLDGLHN ELQTIGFQIE TIGKKVSSKR IPYAPSGEIP KFSLQDPPNK

XenTroUGD GRRVLDNLHK RLQQIGFQVE TIGKKVNPVS SLVYRAASKP ACQEQDPTPA

FlaCF1UGD GRNILNAKEL KSIGFIYNGI GS........ .......... ..........

ZeaMayUGD GRNIVDPEKL REIGFIVYSI GKPLDAWLKD MPAVA..... ..........

MayZebUGD1 GRRVLDHLHP HLQSLGFQIE TIGKKVTPAR IPYTPAAAGP RITASDVPTS

VitvinUGD1 GRNVVDAEKL REIGFIVYSI GKPLDAWLKD MPAVA..... ..........

HomSapUGD1 GRRVLDGLHN ELQTIGFQIE TIGKKVSSKR IPYAPSGEIP KFSLQDPPNK

OviAriUGD1 GRRVLDGLHN ELQTIGFQIE TIGKKVSSKR IPYAPSGEIP KFSLQDMPNK

FelCatUGD1 GRRVLDGLHN ELQTIGFQIE TIGKKVSSKR IPYAPSGEIP KFSLQDPPNK

EquCabUGD1 GRRVLDGLHN ELQTIGFQIE TIGKKVSSKR IPYAPSGEIP KFSLQDPPNK

CanFamUGD1 GRRVLDGLHN ELQTIGFQIE TIGKKVSSKR IPYAPSGEIP KFSLQDPPNK

DroMojGI7 GRKILDHERL LQIGFHVQTI GKKYQRAGLL RSWGIVPQL. ..........

DroPseGA34 GRKILDHERL HQIGFHVQTI GKKYQRSGLL RSWGIVPQL. ..........

HomSapICRA GRRVLDGLHN ELQTIGFQIE TIGKKVSSKR IPYAPSGEIP KFSLQDPPNK

ColLivUGD .......... .......... .......... .......... ..........

CamFloUGD GRKILDHNRL QRIGFVVQTI GKKLTRSAIS RAWGSQTQV. ..........

BosTauUGD GRRVLDGLHN ELQTIGFQIE TIGKKVSSKR IPYAPSGEIP KFSLQDMPNK

MelGal1UGD GRRVLDDLHN ELQVIGFQVE TIGKKVSAKR IPFASSCEIP KFSLQDPPVK

AilMelUGDP GRRVLDGLHN ELQTIGFQIE TIGKKVSSKR IPYAPSGEIP KFSLQDPPNK

MusFurUGDP GRRVLDGLHN ELQTIGFQIE TIGKKVSSKR IPYAPSGEIP KFSLQDPPNK

MusFur1UGD GRRVLDGLHN ELQTIGFQIE TIGKKVSSKR IPYAPSGEIP KFSLQDPPNK

MelUnd1UGD GRRLLDDLHN ELQVIGFQIE TIGKKVSAKR IPFAPSCEIP KFSLQDPPVK

FalPer1UGD GRRVLDDLHN ELQVIGFQIE TIGKKVSAKR IPFASSCEIP KFSLQDPPVK

ChrBel2UGD GRRVLDDLHN ELQVIGFQIE TIGKKVSAKR IPFAPSCEIP KFSLQDPPVK

FelCat1UGD GRRVLDGLHN ELQTIGFQIE TIGKKVSSKR IPYAPSGEIP KFSLQDPPNK

SaiBolUGDP GRRVLDGLHN ELQTIGFQIE TIGKKVSSKR IPYAPSGEIP KFSLQDPPNK

DroWilGK1 GRKILDHERL QQIGFHVQTI GKKYQRTGLL RSWGIVPQL. ..........

CaeRemQV40 GRLILDQKAL RDIGFRTFAI GTSPDQAYNL FGTAGY.... ..........

CaeBriQV40 GRLILDQKAL REIGFRTFAI GTAPDQAYNL FGTAGY.... ..........

ZeaMayUGDI GRNIVDSEKL REIGFIVYSI GKPLDAWLKD MPAVA..... ..........

GalGalUG GRRVLDDLHN ELQVIGFQIE TIGKKVSAKR IPFASSCEIP KFSLQDPPVK

CaeEleUG GRLILDQKAL REIGFRTFAI GTSPDQAYNL FGTAGY.... ..........

PseAerGMD PHTTTAQAEG ICW....... .......... .......... ..........

CanCloGM SITDNNYEGL CW........ .......... .......... ..........

VibCraGMD .......... .......... .......... .......... ..........

PseSyrGMD TNATTEDGRA EGICW..... .......... .......... ..........

AmyAzuGMD .......... .......... .......... .......... ..........

OxaAB1GMD8 AEQSGGQYDG ICW....... .......... .......... ..........

ActGloGDM AEARRATQGY QGLGW..... .......... .......... ..........

AmyAlbGDM7 .......... .......... .......... .......... ..........

AmyBalGDM AAERRLEEGY AGLAW..... .......... .......... ..........

PseDC3GMD5 TNATTEDGRA EGICW..... .......... .......... ..........

PseVerGMD1 SKATSVSGRT EGICW..... .......... .......... ..........

PseQDAGMD1 SKPSSTTSRT EGICW..... .......... .......... ..........

PseAerGMD1 PHTTTAQAEG ICW....... .......... .......... ..........

PseHYSGMD1 HGSDAQREGI CW........ .......... .......... ..........

AmyAlbGMD6 .......... .......... .......... .......... ..........

AmyAzuGMD1 .......... .......... .......... .......... ..........

AlgSagHYPO DDYQMENYEG IGWS...... .......... .......... ..........

AciKBSHYPO PDADGFLSRR R......... .......... .......... ..........

AmyBalGMD AAERRLEEGY AGLAW..... .......... .......... ..........

StrCanGMD1 AETRRTEPGY MGLAW..... .......... .......... ..........

PseAlcGMD1 AHATQANKEG ICW....... .......... .......... ..........

ActGloGMD6 AEARRATQGY QGLGW..... .......... .......... ..........

OxaAB1GMD1 AEQSGGQYDG ICW....... .......... .......... ..........

PseAerUNPP PHTTTAQAEG ICW....... .......... .......... ..........

HahGanHYPO KTKTVDNLEG ICW....... .......... .......... ..........

DesSalNSD DSSLENEHID GICW...... .......... .......... ..........

PseTomGMD1 ANATSEDGRA EGICW..... .......... .......... ..........

MarAlgGMD KTTSTEVLEG ICW....... .......... .......... ..........

PseSyrGMD3 TNATTEDGRA EGICW..... .......... .......... ..........

NovAroGMD .......... .......... .......... .......... ..........

AzoSpGMD NPQALRAEVE GLCW...... .......... .......... ..........

AzoVinGMD PQRTAGAAEG ICW....... .......... .......... ..........

BacThuGMD .......... .......... .......... .......... ..........

HahGanHYPP KTKTVDNLEG ICW....... .......... .......... ..........

AlgSagHYPP DDYQMENYEG IGWS...... .......... .......... ..........

AciKBSHYPP PDADGFLSRR R......... .......... .......... ..........

PseSyrGMD2 ANATS..... .......... .......... .......... ..........

PseSyrGMD1 ANATS..... .......... .......... .......... ..........

StrCanGMD AETRRTEPGY MGLAW..... .......... .......... ..........

PseVerGMD SKATSVSGRT EGICW..... .......... .......... ..........

PseQDAGMD SKPSSTTSRT EGICW..... .......... .......... ..........

PseHYSGMD HGSDAQREGI CW........ .......... .......... ..........

PseAlcGMD AHATQANKEG ICW....... .......... .......... ..........

PseAerUPP PHTTTAQAEG ICW....... .......... .......... ..........

StaAurUMD NFIDK..... .......... .......... .......... ..........

EscColUMND .......... .......... .......... .......... ..........

LusFleUGD .......... .......... .......... .......... ..........

SalEntUMD .......... .......... .......... .......... ..........

ActSerUG SQL....... .......... .......... .......... ..........

ErwTasUG K......... .......... .......... .......... ..........

FusUlcUMD .......... .......... .......... .......... ..........

CorMarUMD .......... .......... .......... .......... ..........

StaVitUMD .......... .......... .......... .......... ..........

SheVioUMD .......... .......... .......... .......... ..........

SerS4UMD .......... .......... .......... .......... ..........

PseStuUMD .......... .......... .......... .......... ..........

PsePseUMD .......... .......... .......... .......... ..........

PseFluUMD .......... .......... .......... .......... ..........

HalSalUMD .......... .......... .......... .......... ..........

FraPhiUMD .......... .......... .......... .......... ..........

EdwC07UMD .......... .......... .......... .......... ..........

AerAquUMD .......... .......... .......... .......... ..........

GeoWSUUMD .......... .......... .......... .......... ..........

EscMG1UMD .......... .......... .......... .......... ..........

DicZeaUMD .......... .......... .......... .......... ..........

VibOrdUMD .......... .......... .......... .......... ..........

VidVulUMaD .......... .......... .......... .......... ..........

KlePneUMaD R......... .......... .......... .......... ..........

GeoTheUMaD NCIGLDDEDV TVYQIGHLSV VQAIQEQPPE RV........ ..........

BurXenUMaD A......... .......... .......... .......... ..........

HasParUMaD TD........ .......... .......... .......... ..........

RalSolUMaD SAKRIAA... .......... .......... .......... ..........

PolIrgUMaD .......... .......... .......... .......... ..........

RalSolNGD SAKRLAA... .......... .......... .......... ..........

YerEntUMaD R......... .......... .......... .......... ..........

EscFerUMaD R......... .......... .......... .......... ..........

OceGraUMaD .......... .......... .......... .......... ..........

BacNRRUMaD AMKDITNREN IELL...... .......... .......... ..........

GeoKauUMaD AMKDVSKPAK YVKL...... .......... .......... ..........

PsyCNPUMaD .......... .......... .......... .......... ..........

MorPE3UMaD .......... .......... .......... .......... ..........

SerProUMaD R......... .......... .......... .......... ..........

651 655

StrPyoUGD .....

HymAerUGD1 .....

MetCapUGD1 .....

ActGloUGD6 .....

HalZhaHYPO .....

MCIThaHYPO .....

SalPacUGD .....

NatGarNSD2 .....

BacSubUGD2 .....

AzoTolUGD .....

LacCreEPS .....

AlaProUG .....

StrZooUG .....

StrHGBNSD .....

NatGarNSD .....

TheMelUGD .....

SulDenUGD .....

SphAlaUGD .....

SheOneUGD .....

SalSerUGD .....

PseHalUGD .....

ProMirUGD .....

ParDisUGD .....

ParDisUGD1 .....

LacSalUGD .....

CloPerUGD .....

ArcButUGD .....

AerHydUGD .....

LacJohSBP .....

StrUbeUGD .....

ReiMEDNSD .....

ExiSibNSD .....

RumObeHYPO .....

RumObeHYP1 .....

FaePraHYPO .....

EubDolHYPO .....

BacPleHYPO .....

BacCopHYPO .....

StrTheESP .....

StrIniCPS .....

SerProUGD .....

GeoMetUGD .....

GeoUraUGD .....

AnoFlaUGD .....

PorGinNSD .....

BacSelRPON .....

PelPhaNSD .....

MetPopNSD .....

MetExtNSD .....

VibVulPRE .....

GeoKauNSD .....

RhiTriEXO .....

ParDisUGD2 .....

XanAxoUGD .....

XanCamUGD .....

EntFaeUGD .....

StrPneUGD2 .....

StrPneUGD3 .....

StrPneUG .....

EscAlbUGD .....

BacTheUGD .....

SacSpiUGD .....

Aer159PBP .....

ButSynUGD .....

BraJapUGD .....

SynCC9UGD .....

StePneUGD .....

SalAreUGD .....

BurYI2NSD .....

BacCerNSD .....

PaeSanHYPO .....

Rhi42MUGD .....

OpiTAV5NSD .....

MarHTCUGD8 .....

BacSubUGD .....

NocKunUGD .....

DesHafUGD .....

BacThuUGD .....

SphJapUGD .....

RicSibUGD .....

MetCapUGD .....

HymAerUGD .....

BacSubUGD7 .....

ActGloUDG9 .....

NatGarNSD5 .....

UncBacHYPO .....

HalZhaHYPP .....

StrPneUGD1 .....

HoePhoUGD .....

CapSpuUGD .....

NovAroUGD .....

ProMarUGD .....

HomSapUGD KPKV.

MusMusUNA PKV..

DanRerUGD A....

XenLaeUGD KQRV.

RatNorUGD PKV..

PonAbeUGD KPKV.

BosTauUGD3 KPRV.

BamOldUGD .....

PedCorUGD .....

ZeaMayUGD8 .....

StrPurUGDP .....

CioIntUGDP .....

NemVecPPRE .....

CaeEleUGD .....

PopTomUGD .....

DroMelUGD .....

AraThaUGD .....

DroMelUGD1 .....

NasVitUDG .....

CaeBriHYPO .....

DroWilGK3 .....

NasVitUGDP .....

DroVirGJ3 .....

HomSapUGD2 KPKV.

XenTroUGD KKVKI

FlaCF1UGD .....

ZeaMayUGD .....

MayZebUGD1 KAKV.

VitvinUGD1 .....

HomSapUGD1 KPKV.

OviAriUGD1 KPRV.

FelCatUGD1 KPRV.

EquCabUGD1 KPKV.

CanFamUGD1 KPRV.

DroMojGI7 .....

DroPseGA34 .....

HomSapICRA KPKV.

ColLivUGD .....

CamFloUGD .....

BosTauUGD KPRV.

MelGal1UGD KPRV.

AilMelUGDP KPRV.

MusFurUGDP KPRV.

MusFur1UGD KPRV.

MelUnd1UGD KPRV.

FalPer1UGD KPRV.

ChrBel2UGD KPRV.

FelCat1UGD KPRV.

SaiBolUGDP KPKV.

DroWilGK1 .....

CaeRemQV40 .....

CaeBriQV40 .....

ZeaMayUGDI .....

GalGalUG KPRV.

CaeEleUG .....

PseAerGMD .....

CanCloGM .....

VibCraGMD .....

PseSyrGMD .....

AmyAzuGMD .....

OxaAB1GMD8 .....

ActGloGDM .....

AmyAlbGDM7 .....

AmyBalGDM .....

PseDC3GMD5 .....

PseVerGMD1 .....

PseQDAGMD1 .....

PseAerGMD1 .....

PseHYSGMD1 .....

AmyAlbGMD6 .....

AmyAzuGMD1 .....

AlgSagHYPO .....

AciKBSHYPO .....

AmyBalGMD .....

StrCanGMD1 .....

PseAlcGMD1 .....

ActGloGMD6 .....

OxaAB1GMD1 .....

PseAerUNPP .....

HahGanHYPO .....

DesSalNSD .....

PseTomGMD1 .....

MarAlgGMD .....

PseSyrGMD3 .....

NovAroGMD .....

AzoSpGMD .....

AzoVinGMD .....

BacThuGMD .....

HahGanHYPP .....

AlgSagHYPP .....

AciKBSHYPP .....

PseSyrGMD2 .....

PseSyrGMD1 .....

StrCanGMD .....

PseVerGMD .....

PseQDAGMD .....

PseHYSGMD .....

PseAlcGMD .....

PseAerUPP .....

StaAurUMD .....

EscColUMND .....

LusFleUGD .....

SalEntUMD .....

ActSerUG .....

ErwTasUG .....

FusUlcUMD .....

CorMarUMD .....

StaVitUMD .....

SheVioUMD .....

SerS4UMD .....

PseStuUMD .....

PsePseUMD .....

PseFluUMD .....

HalSalUMD .....

FraPhiUMD .....

EdwC07UMD .....

AerAquUMD .....

GeoWSUUMD .....

EscMG1UMD .....

DicZeaUMD .....

VibOrdUMD .....

VidVulUMaD .....

KlePneUMaD .....

GeoTheUMaD .....

BurXenUMaD .....

HasParUMaD .....

RalSolUMaD .....

PolIrgUMaD .....

RalSolNGD .....

YerEntUMaD .....

EscFerUMaD .....

OceGraUMaD .....

BacNRRUMaD .....

GeoKauUMaD .....

PsyCNPUMaD .....

MorPE3UMaD .....

SerProUMaD .....
