# Supplementary material for: Analysis of nucleotide diphosphate sugar dehydrogenases reveals family and group‐specific relationships
Source: FEBS Open Bio. 2016 Jan 11;6(1):77–89. doi: 10.1002/2211-5463.12022 (PMC4794789; doi:10.1002/2211-5463.12022)
Supplement: Supplementary file 3 — Table S1. Complete GEnt results of GDPMDHs. [file FEB4-6-77-s003.docx]

Table S1. Complete GEnt results of GDPMDHs.

| Index | SeqAln | Entropy | SeqGrp | GroupEntropy | PartGroup | SeqNotGroup |
| --- | --- | --- | --- | --- | --- | --- |
| 359 | h | 2.206 | h | 12.231 | 7.235 | l |
| 219 | p | 2.472 | l | 9.762 | 6.126 | p |
| 406 | g | 2.834 | a | 9.594 | 6.07 | g |
| 215 | k | 2.368 | r | 9.453 | 6.011 | k |
| 355 | c | 1.655 | c | 8.764 | 5.348 | a |
| 497 | a | 1.277 | l | 7.814 | 4.947 | s |
| 274 | r | 2.746 | m | 7.744 | 4.545 | r |
| 186 | a | 1.281 | l | 7.678 | 4.733 | c |
| 95 | l | 1.485 | c | 7.445 | 4.506 | g |
| 167 | v | 1.071 | s | 7.428 | 4.066 | v |
| 446 | k | 1.258 | v | 7.234 | 4.21 | r |
| 318 | w | 1.92 | l | 7.127 | 2.677 | w |
| 269 | l | 1.484 | y | 7.094 | 4.283 | n |
| 449 | f | 1.191 | a | 6.888 | 3.318 | f |
| 417 | w | 1.505 | r | 6.72 | 3.857 | l |
| 399 | h | 1.179 | y | 6.629 | 3.318 | k |
| 170 | c | 2.181 | c | 6.58 | 3.753 | s |
| 210 | h | 0.985 | h | 6.559 | 3.901 | k |
| 97 | t | 1.478 | c | 6.485 | 4.006 | l |
| 374 | e | 1.283 | r | 6.449 | 3.42 | e |
| 389 | c | 1.856 | l | 6.381 | 2.857 | g |
| 387 | a | 1.277 | v | 6.3 | 3.319 | g |
| 435 | w | 2.406 | l | 6.266 | 1.944 | w |
| 385 | i | 1.56 | m | 6.254 | 4.042 | a |
| 153 | f | 2.058 | g | 6.191 | 2.754 | f |
| 100 | c | 1.476 | c | 6.188 | 2.733 | v |
| 273 | d | 1.945 | p | 6.138 | 2.97 | d |
| 381 | v | 1.368 | g | 6.113 | 3.201 | i |
| 393 | r | 2.49 | a | 6.03 | 3.225 | r |
| 220 | v | 2.44 | p | 5.901 | 2.488 | v |
| 370 | s | 1.48 | g | 5.896 | 3.325 | d |
| 366 | a | 1.683 | a | 5.717 | 1.706 | f |
| 346 | w | 1.578 | e | 5.493 | 2.053 | w |
| 255 | h | 1.445 | v | 5.48 | 3.154 | w |
| 228 | r | 0.498 | i | 5.428 | 3.305 | r |
| 252 | q | 0.8 | g | 5.378 | 3.001 | q |
| 342 | t | 0.68 | r | 5.343 | 3.013 | f |
| 433 | r | 0.844 | p | 5.298 | 3.092 | n |
| 195 | c | 1.535 | c | 5.296 | 3.72 | i |
| 209 | y | 0.83 | w | 5.272 | 3.252 | d |
| 439 | i | 1.113 | m | 5.268 | 2.954 | i |
| 270 | l | 0.695 | d | 5.228 | 2.823 | l |
| 90 | l | 1.298 | l | 5.164 | 1.681 | a |
| 214 | e | 0.806 | f | 5.098 | 2.647 | e |
| 343 | t | 1.832 | k | 5.084 | 2.946 | m |
| 175 | y | 1.012 | s | 5.065 | 2.485 | y |
| 112 | c | 1.679 | g | 5.048 | 1.563 | c |
| 452 | r | 0.957 | l | 5.048 | 2.651 | r |
| 199 | a | 1.054 | g | 5.033 | 2.535 | m |
| 227 | r | 0.653 | l | 5.008 | 2.245 | r |
| 172 | p | 2.204 | g | 4.936 | 2.091 | n |
| 347 | s | 1.33 | v | 4.903 | 2.607 | s |
| 229 | w | 1.235 | p | 4.852 | 2.522 | i |
| 474 | t | 0.544 | d | 4.838 | 2.694 | t |
| 353 | y | 2.363 | w | 4.809 | 2.164 | l |
| 184 | h | 1.152 | g | 4.769 | 1.875 | r |
| 99 | a | 1.058 | g | 4.754 | 2.674 | v |
| 231 | d | 0.358 | l | 4.729 | 2.183 | d |
| 280 | m | 0.613 | f | 4.646 | 2.406 | e |
| 96 | p | 2.112 | i | 4.61 | 2.106 | s |
| 362 | r | 2.246 | k | 4.597 | 1.984 | r |
| 450 | a | 1.022 | f | 4.579 | 2.291 | a |
| 123 | w | 2.396 | c | 4.512 | 0.914 | w |
| 164 | a | 1.867 | t | 4.496 | 2.335 | a |
| 358 | f | 2.78 | w | 4.486 | 3.047 | m |
| 223 | t | 1.279 | c | 4.46 | 2.356 | a |
| 492 | t | 1.367 | l | 4.417 | 1.645 | t |
| 412 | f | 2.273 | m | 4.345 | 1.512 | f |
| 476 | k | 1.061 | r | 4.301 | 2.397 | l |
| 402 | q | 1.202 | r | 4.26 | 2.243 | y |
| 418 | n | 1.33 | g | 4.256 | 1.878 | q |
| 436 | q | 0.963 | n | 4.236 | 1.978 | q |
| 308 | v | 0.739 | g | 4.179 | 2.413 | i |
| 441 | m | 1.497 | w | 4.103 | 1.6 | m |
| 405 | f | 1.686 | y | 4.058 | 1.601 | v |
| 437 | q | 0.991 | h | 4.034 | 2.067 | q |
| 345 | t | 0.711 | i | 4.022 | 1.956 | t |
| 108 | h | 1.392 | h | 3.964 | 1.49 | i |
| 498 | i | 1.301 | v | 3.883 | 1.957 | q |
| 187 | d | 2.514 | c | 3.871 | 2.514 | d |
| 254 | a | 1.393 | c | 3.846 | 1.596 | l |
| 488 | n | 1.955 | g | 3.837 | 1.93 | d |
| 475 | d | 0.881 | c | 3.813 | 1.981 | d |
| 390 | m | 0.765 | q | 3.743 | 2.187 | y |
| 392 | p | 1.188 | y | 3.666 | 1.857 | q |
| 122 | m | 0.643 | l | 3.664 | 1.69 | a |
| 422 | n | 0.529 | r | 3.664 | 2.103 | n |
| 211 | i | 1.556 | t | 3.64 | 1.768 | i |
| 131 | s | 0.954 | a | 3.605 | 1.571 | l |
| 279 | g | 1.408 | e | 3.574 | 1.804 | g |
| 444 | y | 0.747 | e | 3.55 | 1.381 | y |
| 338 | g | 0.618 | a | 3.526 | 1.726 | i |
| 404 | g | 2.594 | g | 3.524 | 1.01 | s |
| 200 | q | 0.671 | y | 3.451 | 2.194 | q |
| 141 | v | 1.268 | l | 3.436 | 1.405 | y |
| 388 | v | 1.687 | f | 3.435 | 2.291 | a |
| 379 | a | 1.396 | v | 3.385 | 1.559 | a |
| 382 | w | 1.085 | h | 3.37 | 1.673 | e |
| 454 | i | 0.572 | a | 3.359 | 1.622 | i |
| 364 | s | 1.861 | t | 3.351 | 1.889 | s |
| 152 | f | 0.984 | r | 3.325 | 1.179 | f |
| 484 | a | 1.649 | w | 3.311 | 1.658 | i |
| 357 | a | 1.315 | v | 3.292 | 1.985 | w |
| 134 | y | 1.502 | i | 3.266 | 1.371 | y |
| 261 | r | 1.54 | w | 3.24 | 1.19 | a |
| 400 | y | 2.319 | y | 3.156 | 1.412 | f |
| 197 | m | 0.613 | e | 3.149 | 1.451 | m |
| 440 | d | 0.773 | p | 3.114 | 1.622 | d |
| 480 | c | 1.71 | l | 3.099 | 1.586 | c |
| 365 | f | 2.25 | f | 3.064 | 0.968 | s |
| 126 | g | 1.482 | g | 3.061 | 1 | r |
| 401 | l | 1.758 | m | 3.058 | 1.801 | n |
| 224 | e | 1.073 | l | 2.988 | 1.51 | e |
| 201 | v | 0.782 | a | 2.955 | 1.372 | y |
| 271 | n | 0.924 | l | 2.951 | 1.604 | y |
| 386 | r | 0.717 | d | 2.939 | 1.202 | r |
| 317 | h | 0.773 | g | 2.935 | 1.461 | i |
| 398 | n | 0.518 | q | 2.901 | 1.524 | y |
| 118 | h | 0.562 | t | 2.881 | 1.654 | h |
| 361 | q | 1.222 | a | 2.858 | 1 | q |
| 445 | q | 2.102 | h | 2.841 | 1.642 | r |
| 194 | c | 1.896 | l | 2.827 | 1.103 | c |
| 416 | v | 1.385 | l | 2.814 | 1.311 | t |
| 447 | r | 0.655 | q | 2.799 | 1.451 | r |
| 230 | f | 1.326 | i | 2.751 | 1.24 | f |
| 168 | f | 1.732 | m | 2.75 | 1.743 | f |
| 103 | h | 0.562 | g | 2.744 | 1.421 | h |
| 376 | t | 0.703 | v | 2.741 | 1.119 | t |
| 309 | q | 0.904 | d | 2.73 | 1.495 | w |
| 351 | c | 1.235 | m | 2.683 | 1.006 | c |
| 185 | a | 0.602 | d | 2.681 | 1.35 | y |
| 496 | p | 2.33 | p | 2.649 | 0.869 | s |
| 225 | s | 0.653 | n | 2.621 | 1.51 | w |
| 307 | c | 1.473 | p | 2.603 | 1.295 | r |
| 413 | p | 2.152 | p | 2.591 | 0.877 | q |
| 312 | c | 0.875 | q | 2.569 | 1.142 | c |
| 438 | v | 1.527 | l | 2.558 | 1.692 | a |
| 372 | i | 1.681 | t | 2.554 | 0.938 | y |
| 119 | k | 1.863 | i | 2.529 | 0.986 | r |
| 434 | y | 1.826 | i | 2.516 | 0.646 | y |
| 341 | i | 1.25 | f | 2.502 | 1.323 | l |
| 448 | w | 1.363 | n | 2.404 | 1.333 | f |
| 150 | n | 1.145 | a | 2.381 | 1.022 | n |
| 222 | t | 1.707 | k | 2.376 | 0.897 | y |
| 218 | v | 1.747 | m | 2.374 | 1.605 | s |
| 139 | d | 1.131 | g | 2.319 | 1.325 | k |
| 251 | f | 1.159 | f | 2.314 | 1.02 | d |
| 420 | v | 1.255 | t | 2.265 | 1.224 | w |
| 344 | n | 1.344 | p | 2.254 | 1.244 | n |
| 421 | y | 1.522 | h | 2.196 | 0.834 | a |
| 487 | p | 1.36 | v | 2.157 | 0.817 | k |
| 403 | p | 2.793 | p | 2.122 | 0.644 | a |
| 121 | d | 1.42 | d | 2.119 | 0.964 | n |
| 191 | v | 1.999 | m | 2.118 | 1.088 | w |
| 101 | f | 1.634 | l | 2.089 | 0.911 | i |
| 98 | c | 1.893 | l | 2.073 | 0.865 | m |
| 410 | h | 2.458 | t | 2.069 | 0.644 | y |
| 189 | k | 0.745 | d | 2.064 | 1 | k |
| 104 | m | 0.805 | d | 2.027 | 1.031 | f |
| 350 | m | 2.215 | i | 2.022 | 0.886 | l |
| 490 | d | 2.446 | d | 2.014 | 0.607 | g |
| 368 | e | 2.158 | e | 2.006 | 0.593 | s |
| 137 | g | 1.419 | r | 1.964 | 1.22 | y |
| 432 | a | 0.947 | h | 1.943 | 1.13 | f |
| 371 | a | 0.678 | n | 1.84 | 0.88 | m |
| 111 | t | 1.13 | v | 1.816 | 0.775 | t |
| 162 | k | 0.561 | m | 1.798 | 0.953 | k |
| 163 | e | 0.921 | a | 1.741 | 0.849 | e |
| 208 | g | 0.821 | t | 1.715 | 0.816 | p |
| 193 | s | 0.918 | r | 1.708 | 1.016 | a |
| 360 | a | 2.17 | g | 1.672 | 1.034 | h |
| 348 | a | 2.624 | v | 1.668 | 0.493 | s |
| 154 | t | 1.665 | t | 1.631 | 0.661 | s |
| 311 | l | 1.252 | v | 1.62 | 0.708 | f |
| 117 | q | 0.826 | l | 1.612 | 0.696 | e |
| 354 | t | 1.412 | c | 1.573 | 0.569 | f |
| 482 | g | 3.048 | a | 1.545 | 0.31 | r |
| 221 | g | 2.284 | a | 1.538 | 0.54 | m |
| 310 | a | 0.562 | m | 1.518 | 0.804 | q |
| 407 | f | 2.555 | f | 1.499 | 0.651 | w |
| 263 | g | 2.784 | w | 1.488 | 0.834 | g |
| 266 | i | 1.592 | v | 1.474 | 0.631 | w |
| 161 | i | 1.405 | v | 1.466 | 0.509 | y |
| 339 | p | 0.993 | p | 1.458 | 0.632 | r |
| 264 | t | 1.127 | s | 1.45 | 0.631 | q |
| 256 | c | 2.625 | q | 1.444 | 0.617 | s |
| 305 | q | 0.579 | t | 1.402 | 0.626 | q |
| 166 | l | 0.98 | m | 1.353 | 0.571 | f |
| 485 | f | 3.471 | w | 1.345 | 0.322 | m |
| 478 | i | 2.393 | v | 1.273 | 0.659 | f |
| 375 | a | 0.771 | n | 1.266 | 0.538 | m |
| 306 | r | 0.741 | y | 1.261 | 0.659 | w |
| 477 | k | 1.462 | r | 1.233 | 0.444 | v |
| 314 | l | 1.754 | m | 1.225 | 0.458 | v |
| 455 | d | 0.55 | h | 1.218 | 0.579 | e |
| 265 | a | 1.666 | s | 1.216 | 0.751 | v |
| 102 | a | 2.401 | t | 1.212 | 0.676 | a |
| 313 | a | 0.685 | s | 1.209 | 0.539 | w |
| 213 | v | 2.266 | a | 1.201 | 0.5 | t |
| 176 | k | 1.315 | a | 1.173 | 0.568 | d |
| 130 | k | 0.869 | k | 1.149 | 0.508 | i |
| 253 | v | 2.127 | g | 1.128 | 0.437 | i |
| 116 | n | 1.258 | s | 1.125 | 0.486 | i |
| 143 | k | 0.736 | q | 1.114 | 0.53 | f |
| 165 | d | 2.27 | e | 1.079 | 0.612 | l |
| 363 | i | 2.754 | g | 1.072 | 0.55 | i |
| 125 | q | 0.903 | g | 1.069 | 0.503 | s |
| 267 | y | 0.814 | q | 1.066 | 0.486 | f |
| 481 | l | 1.958 | v | 1.025 | 0.393 | w |
| 451 | d | 0.975 | l | 1.015 | 0.442 | c |
| 174 | p | 3.181 | c | 1.001 | 0.268 | n |
| 384 | v | 2.063 | m | 0.966 | 0.443 | i |
| 483 | f | 2.075 | q | 0.954 | 0.377 | w |
| 133 | i | 2.927 | v | 0.938 | 0.553 | f |
| 109 | q | 0.912 | e | 0.902 | 0.446 | t |
| 135 | e | 2.887 | q | 0.869 | 0.287 | d |
| 315 | y | 3.355 | h | 0.845 | 0.275 | l |
| 144 | e | 0.587 | e | 0.823 | 0.351 | r |
| 491 | d | 3.329 | e | 0.823 | 0.256 | n |
| 489 | t | 2.119 | k | 0.818 | 0.365 | i |
| 479 | a | 1.911 | n | 0.793 | 0.394 | a |
| 171 | v | 2.792 | i | 0.782 | 0.245 | t |
| 259 | f | 3.483 | y | 0.761 | 0.316 | r |
| 373 | c | 3.708 | e | 0.757 | 0.393 | c |
| 262 | e | 2.95 | d | 0.754 | 0.332 | p |
| 443 | d | 1.077 | a | 0.75 | 0.386 | d |
| 124 | n | 2.433 | s | 0.746 | 0.461 | r |
| 212 | v | 2.362 | f | 0.712 | 0.277 | m |
| 272 | p | 2.33 | p | 0.692 | 0.295 | t |
| 115 | v | 1.578 | p | 0.656 | 0.294 | k |
| 495 | s | 2.576 | w | 0.653 | 0.232 | t |
| 369 | i | 1.98 | t | 0.65 | 0.316 | m |
| 120 | i | 2.659 | l | 0.597 | 0.339 | v |
| 316 | e | 1.03 | g | 0.593 | 0.279 | k |
| 414 | k | 2.633 | r | 0.588 | 0.236 | i |
| 136 | p | 1.957 | e | 0.579 | 0.339 | f |
| 198 | i | 2.176 | m | 0.562 | 0.253 | v |
| 132 | p | 2.686 | a | 0.553 | 0.205 | y |
| 391 | d | 3.001 | e | 0.547 | 0.229 | h |
| 140 | e | 1.052 | a | 0.517 | 0.238 | r |
| 494 | e | 2.1 | y | 0.503 | 0.239 | s |
| 380 | d | 2.636 | s | 0.478 | 0.211 | n |
| 155 | t | 1.774 | v | 0.477 | 0.202 | l |
| 156 | d | 1.471 | d | 0.472 | 0.212 | m |
| 190 | y | 2.581 | f | 0.459 | 0.184 | s |
| 196 | r | 1.539 | e | 0.45 | 0.242 | q |
| 260 | l | 2.315 | f | 0.448 | 0.188 | v |
| 151 | l | 2.522 | i | 0.393 | 0.173 | f |
| 93 | v | 2.843 | g | 0.385 | 0.169 | i |
| 277 | i | 2.743 | f | 0.384 | 0.188 | v |
| 419 | l | 2.188 | t | 0.381 | 0.17 | i |
| 113 | v | 2.423 | c | 0.369 | 0.144 | f |
| 169 | i | 3.057 | l | 0.369 | 0.217 | v |
| 268 | d | 2.475 | d | 0.356 | 0.156 | e |
| 383 | e | 1.507 | k | 0.353 | 0.168 | m |
| 453 | i | 1.857 | c | 0.308 | 0.149 | a |
| 192 | e | 1.483 | f | 0.286 | 0.145 | y |
| 138 | l | 2.099 | c | 0.279 | 0.134 | d |
| 188 | l | 1.911 | q | 0.188 | 0.097 | i |
| 110 | v | 3.074 | v | 0.186 | 0.07 | i |
| 356 | n | 4.224 | d | 0.153 | 0.092 | n |
| 367 | n | 4.224 | d | 0.153 | 0.092 | n |
| 442 | n | 4.224 | d | 0.153 | 0.092 | n |
| 216 | s | 3.571 | a | 0.144 | 0.087 | s |
| 114 | d | 4.045 | c | 0.135 | 0.081 | d |
| 173 | t | 3.924 | a | 0.135 | 0.081 | t |
| 217 | t | 3.924 | a | 0.135 | 0.081 | t |
| 415 | d | 4.045 | c | 0.135 | 0.081 | d |
| 258 | e | 3.759 | s | 0.132 | 0.08 | e |
| 349 | e | 3.759 | s | 0.132 | 0.08 | e |
| 378 | g | 2.388 | s | 0.115 | 0.05 | q |
| 493 | r | 4.116 | e | 0.111 | 0.067 | r |
| 257 | p | 4.179 | r | 0.103 | 0.062 | p |
| 352 | k | 3.882 | y | 0.099 | 0.06 | k |
| 486 | k | 3.882 | y | 0.099 | 0.06 | k |
| 89 | g | 3.719 | c | 0.091 | 0.055 | g |
| 91 | g | 3.719 | c | 0.091 | 0.055 | g |
| 94 | g | 3.719 | c | 0.091 | 0.055 | g |
| 408 | g | 3.719 | c | 0.091 | 0.055 | g |
| 409 | g | 3.719 | c | 0.091 | 0.055 | g |
| 278 | g | 3.318 | d | 0.059 | 0.024 | s |
| 411 | c | 5.835 | a | 0.048 | 0.029 | c |
| 92 | y | 4.649 | f | 0.031 | 0.016 | h |
