# Supplementary material for: Analysis of nucleotide diphosphate sugar dehydrogenases reveals family and group‐specific relationships
Source: FEBS Open Bio. 2016 Jan 11;6(1):77–89. doi: 10.1002/2211-5463.12022 (PMC4794789; doi:10.1002/2211-5463.12022)
Supplement: Supplementary file 4 — Table S2. Complete GEnt results of UDPNAMDHs. [file FEB4-6-77-s004.docx]

Table S2. Complete GEnt results of UDPNAMDHs.

| Index | SeqAln | Entropy | SeqGrp | GroupEntropy | PartGroup | SeqNotGroup |
| --- | --- | --- | --- | --- | --- | --- |
| 259 | f | 3.483 | k | 12.777 | 4.016 | f |
| 262 | e | 2.95 | p | 11.128 | 4.039 | e |
| 359 | h | 2.206 | r | 10.593 | 4.024 | l |
| 414 | k | 2.633 | v | 10.251 | 4.073 | k |
| 407 | f | 2.555 | v | 10.061 | 3.629 | f |
| 410 | h | 2.458 | h | 9.943 | 3.979 | s |
| 215 | k | 2.368 | e | 9.924 | 3.964 | r |
| 391 | d | 3.001 | h | 9.609 | 4.03 | d |
| 418 | n | 1.33 | f | 9.595 | 4.264 | q |
| 256 | c | 2.625 | c | 9.4 | 3.899 | n |
| 416 | v | 1.385 | p | 9.17 | 3.909 | i |
| 388 | v | 1.687 | a | 8.842 | 3.499 | i |
| 400 | y | 2.319 | i | 8.768 | 3.846 | y |
| 261 | r | 1.54 | l | 8.75 | 3.858 | r |
| 272 | p | 2.33 | n | 8.474 | 3.545 | p |
| 355 | c | 1.655 | e | 8.417 | 3.675 | c |
| 389 | c | 1.856 | n | 8.379 | 3.739 | c |
| 362 | r | 2.246 | n | 8.05 | 3.344 | r |
| 260 | l | 2.315 | v | 7.854 | 3.613 | l |
| 255 | h | 1.445 | h | 7.816 | 3.625 | s |
| 445 | q | 2.102 | k | 7.765 | 3.389 | q |
| 417 | w | 1.505 | w | 7.723 | 3.842 | k |
| 311 | l | 1.252 | a | 7.692 | 3.339 | l |
| 360 | a | 2.17 | d | 7.691 | 3.486 | a |
| 446 | k | 1.258 | p | 7.574 | 3.44 | k |
| 405 | f | 1.686 | a | 7.33 | 2.548 | f |
| 439 | i | 1.113 | r | 7.036 | 3.186 | i |
| 435 | w | 2.406 | i | 6.992 | 1.771 | w |
| 489 | t | 2.119 | v | 6.987 | 3.372 | s |
| 268 | d | 2.475 | e | 6.661 | 3.062 | d |
| 448 | w | 1.363 | w | 6.627 | 3.696 | r |
| 132 | p | 2.686 | h | 6.579 | 3.058 | p |
| 398 | n | 0.518 | v | 6.393 | 3.19 | n |
| 175 | y | 1.012 | f | 6.356 | 3.127 | t |
| 167 | v | 1.071 | w | 6.302 | 3.61 | s |
| 371 | a | 0.678 | m | 6.281 | 3.413 | n |
| 412 | f | 2.273 | i | 6.276 | 3.04 | f |
| 382 | w | 1.085 | w | 6.261 | 3.168 | e |
| 161 | i | 1.405 | p | 6.239 | 3.274 | y |
| 317 | h | 0.773 | i | 6.205 | 3.267 | h |
| 351 | c | 1.235 | c | 6.113 | 3.836 | i |
| 480 | c | 1.71 | c | 6.082 | 3.268 | l |
| 209 | y | 0.83 | g | 6.059 | 2.652 | y |
| 413 | p | 2.152 | a | 6.024 | 3.045 | q |
| 166 | l | 0.98 | c | 5.943 | 2.789 | f |
| 265 | a | 1.666 | v | 5.879 | 3.02 | a |
| 93 | v | 2.843 | i | 5.86 | 3.269 | v |
| 172 | p | 2.204 | p | 5.786 | 1.786 | n |
| 438 | v | 1.527 | g | 5.747 | 2.665 | i |
| 229 | w | 1.235 | w | 5.6 | 3.364 | i |
| 214 | e | 0.806 | l | 5.449 | 2.573 | e |
| 318 | w | 1.92 | y | 5.408 | 2.014 | w |
| 119 | k | 1.863 | a | 5.383 | 2.89 | k |
| 218 | v | 1.747 | s | 5.267 | 2.79 | m |
| 112 | c | 1.679 | g | 5.216 | 1.617 | c |
| 419 | l | 2.188 | i | 5.186 | 2.935 | l |
| 379 | a | 1.396 | i | 5.173 | 2.37 | a |
| 387 | a | 1.277 | m | 5.128 | 2.684 | g |
| 449 | f | 1.191 | c | 5.084 | 2.314 | f |
| 280 | m | 0.613 | m | 5.06 | 2.704 | d |
| 476 | k | 1.061 | l | 5.011 | 2.123 | k |
| 421 | y | 1.522 | s | 4.959 | 1.692 | y |
| 95 | l | 1.485 | l | 4.839 | 1.364 | c |
| 366 | a | 1.683 | g | 4.82 | 1.522 | f |
| 307 | c | 1.473 | c | 4.781 | 2.857 | r |
| 186 | a | 1.281 | c | 4.768 | 2.151 | f |
| 194 | c | 1.896 | s | 4.57 | 1.577 | c |
| 251 | f | 1.159 | v | 4.559 | 2.298 | y |
| 392 | p | 1.188 | p | 4.54 | 1.792 | q |
| 123 | w | 2.396 | c | 4.532 | 0.881 | w |
| 385 | i | 1.56 | i | 4.513 | 1.545 | a |
| 90 | l | 1.298 | l | 4.484 | 1.542 | a |
| 361 | q | 1.222 | i | 4.451 | 1.945 | q |
| 153 | f | 2.058 | v | 4.423 | 1.21 | f |
| 150 | n | 1.145 | y | 4.378 | 2.44 | n |
| 364 | s | 1.861 | a | 4.34 | 1.683 | s |
| 227 | r | 0.653 | a | 4.319 | 1.99 | r |
| 338 | g | 0.618 | g | 4.291 | 2.002 | i |
| 108 | h | 1.392 | k | 4.288 | 2.044 | h |
| 345 | t | 0.711 | a | 4.235 | 1.848 | i |
| 134 | y | 1.502 | g | 4.181 | 1.257 | y |
| 384 | v | 2.063 | l | 4.112 | 2.397 | i |
| 184 | h | 1.152 | y | 4.11 | 2.095 | g |
| 210 | h | 0.985 | m | 4.003 | 2.064 | h |
| 200 | q | 0.671 | p | 3.964 | 2.122 | y |
| 422 | n | 0.529 | q | 3.963 | 1.915 | i |
| 441 | m | 1.497 | t | 3.933 | 1.39 | m |
| 264 | t | 1.127 | q | 3.909 | 1.769 | t |
| 254 | a | 1.393 | a | 3.793 | 1.246 | l |
| 211 | i | 1.556 | l | 3.774 | 1.89 | i |
| 399 | h | 1.179 | n | 3.755 | 1.841 | y |
| 346 | w | 1.578 | r | 3.743 | 1.394 | w |
| 208 | g | 0.821 | k | 3.643 | 2.04 | g |
| 404 | g | 2.594 | g | 3.634 | 1.036 | s |
| 347 | s | 1.33 | t | 3.6 | 1.747 | s |
| 96 | p | 2.112 | p | 3.585 | 1.082 | v |
| 454 | i | 0.572 | k | 3.501 | 1.731 | i |
| 390 | m | 0.765 | r | 3.479 | 1.831 | m |
| 197 | m | 0.613 | s | 3.376 | 1.674 | r |
| 156 | d | 1.471 | m | 3.346 | 1.668 | d |
| 450 | a | 1.022 | l | 3.34 | 1.487 | f |
| 131 | s | 0.954 | i | 3.316 | 1.705 | s |
| 223 | t | 1.279 | m | 3.196 | 1.364 | c |
| 126 | g | 1.482 | g | 3.171 | 1.032 | r |
| 98 | c | 1.893 | r | 3.113 | 0.997 | c |
| 339 | p | 0.993 | e | 3.036 | 1.35 | p |
| 376 | t | 0.703 | q | 3.008 | 1.539 | t |
| 122 | m | 0.643 | c | 2.992 | 1.501 | l |
| 168 | f | 1.732 | a | 2.959 | 1.264 | f |
| 100 | c | 1.476 | f | 2.927 | 1.148 | c |
| 103 | h | 0.562 | s | 2.907 | 1.354 | q |
| 308 | v | 0.739 | s | 2.894 | 1.605 | v |
| 101 | f | 1.634 | w | 2.878 | 1.537 | i |
| 185 | a | 0.602 | e | 2.878 | 1.462 | y |
| 375 | a | 0.771 | d | 2.856 | 1.324 | a |
| 269 | l | 1.484 | m | 2.831 | 0.899 | n |
| 402 | q | 1.202 | l | 2.828 | 1.244 | n |
| 252 | q | 0.8 | y | 2.764 | 1.378 | g |
| 365 | f | 2.25 | l | 2.76 | 0.921 | s |
| 374 | e | 1.283 | d | 2.736 | 1.45 | r |
| 353 | y | 2.363 | i | 2.731 | 0.731 | y |
| 369 | i | 1.98 | f | 2.715 | 0.882 | i |
| 342 | t | 0.68 | i | 2.663 | 1.222 | f |
| 497 | a | 1.277 | a | 2.647 | 1.004 | i |
| 225 | s | 0.653 | q | 2.614 | 1.205 | w |
| 498 | i | 1.301 | f | 2.595 | 1.295 | q |
| 270 | l | 0.695 | i | 2.58 | 1.446 | d |
| 437 | q | 0.991 | m | 2.534 | 1.389 | h |
| 162 | k | 0.561 | v | 2.483 | 1.102 | r |
| 434 | y | 1.826 | q | 2.472 | 0.656 | y |
| 279 | g | 1.408 | g | 2.462 | 0.871 | e |
| 354 | t | 1.412 | m | 2.435 | 1.1 | a |
| 341 | i | 1.25 | h | 2.419 | 1.183 | l |
| 170 | c | 2.181 | v | 2.407 | 0.937 | s |
| 492 | t | 1.367 | y | 2.342 | 0.943 | t |
| 118 | h | 0.562 | h | 2.21 | 1.3 | v |
| 403 | p | 2.793 | p | 2.198 | 0.663 | a |
| 271 | n | 0.924 | q | 2.161 | 0.88 | y |
| 231 | d | 0.358 | a | 2.146 | 1.111 | r |
| 350 | m | 2.215 | f | 2.121 | 0.903 | a |
| 447 | r | 0.655 | f | 2.089 | 1.175 | r |
| 483 | f | 2.075 | v | 2.087 | 0.663 | w |
| 267 | y | 0.814 | v | 2.086 | 1.063 | k |
| 368 | e | 2.158 | e | 2.082 | 0.614 | s |
| 444 | y | 0.747 | g | 2.031 | 0.951 | w |
| 137 | g | 1.419 | v | 2.024 | 1.01 | y |
| 372 | i | 1.681 | i | 2.024 | 0.755 | y |
| 266 | i | 1.592 | w | 1.986 | 1.098 | i |
| 436 | q | 0.963 | r | 1.981 | 1.132 | n |
| 120 | i | 2.659 | g | 1.952 | 0.848 | i |
| 496 | p | 2.33 | h | 1.93 | 0.684 | s |
| 104 | m | 0.805 | c | 1.902 | 0.85 | m |
| 380 | d | 2.636 | n | 1.898 | 0.958 | d |
| 195 | c | 1.535 | l | 1.874 | 0.748 | c |
| 401 | l | 1.758 | h | 1.805 | 0.681 | n |
| 163 | e | 0.921 | p | 1.801 | 1.086 | d |
| 485 | f | 3.471 | y | 1.8 | 0.583 | m |
| 213 | v | 2.266 | i | 1.788 | 0.858 | t |
| 343 | t | 1.832 | s | 1.749 | 0.597 | m |
| 348 | a | 2.624 | v | 1.721 | 0.503 | s |
| 130 | k | 0.869 | e | 1.717 | 0.903 | t |
| 370 | s | 1.48 | v | 1.698 | 0.704 | d |
| 154 | t | 1.665 | h | 1.693 | 1.031 | s |
| 358 | f | 2.78 | c | 1.675 | 0.649 | w |
| 111 | t | 1.13 | f | 1.661 | 0.726 | t |
| 357 | a | 1.315 | s | 1.596 | 0.829 | v |
| 490 | d | 2.446 | e | 1.594 | 0.571 | g |
| 228 | r | 0.498 | q | 1.582 | 0.662 | i |
| 482 | g | 3.048 | a | 1.58 | 0.313 | r |
| 433 | r | 0.844 | k | 1.575 | 0.72 | p |
| 305 | q | 0.579 | p | 1.503 | 0.717 | h |
| 97 | t | 1.478 | t | 1.481 | 0.525 | c |
| 140 | e | 1.052 | m | 1.478 | 0.816 | e |
| 316 | e | 1.03 | k | 1.465 | 0.72 | e |
| 310 | a | 0.562 | y | 1.451 | 0.826 | i |
| 230 | f | 1.326 | p | 1.427 | 0.565 | f |
| 432 | a | 0.947 | s | 1.406 | 0.566 | f |
| 125 | q | 0.903 | r | 1.404 | 0.641 | s |
| 201 | v | 0.782 | q | 1.403 | 0.597 | n |
| 488 | n | 1.955 | d | 1.398 | 0.45 | g |
| 143 | k | 0.736 | h | 1.392 | 0.764 | f |
| 199 | a | 1.054 | p | 1.381 | 0.612 | m |
| 152 | f | 0.984 | q | 1.373 | 0.528 | f |
| 440 | d | 0.773 | l | 1.366 | 0.736 | d |
| 196 | r | 1.539 | l | 1.346 | 0.703 | r |
| 487 | p | 1.36 | g | 1.341 | 0.659 | k |
| 475 | d | 0.881 | e | 1.325 | 0.735 | p |
| 141 | v | 1.268 | q | 1.32 | 0.551 | y |
| 188 | l | 1.911 | m | 1.313 | 0.754 | t |
| 138 | l | 2.099 | n | 1.252 | 0.435 | i |
| 315 | y | 3.355 | y | 1.235 | 0.34 | l |
| 222 | t | 1.707 | s | 1.213 | 0.396 | y |
| 221 | g | 2.284 | m | 1.204 | 0.671 | r |
| 363 | i | 2.754 | f | 1.186 | 0.444 | v |
| 219 | p | 2.472 | a | 1.165 | 0.397 | l |
| 373 | c | 3.708 | g | 1.156 | 0.433 | a |
| 453 | i | 1.857 | t | 1.153 | 0.529 | i |
| 312 | c | 0.875 | i | 1.106 | 0.537 | c |
| 381 | v | 1.368 | v | 1.092 | 0.44 | g |
| 455 | d | 0.55 | m | 1.087 | 0.598 | d |
| 273 | d | 1.945 | n | 1.069 | 0.498 | s |
| 479 | a | 1.911 | i | 1.068 | 0.478 | g |
| 224 | e | 1.073 | e | 1.058 | 0.453 | l |
| 174 | p | 3.181 | c | 1.032 | 0.274 | n |
| 117 | q | 0.826 | q | 1.01 | 0.535 | y |
| 253 | v | 2.127 | y | 1.003 | 0.591 | v |
| 474 | t | 0.544 | c | 0.985 | 0.467 | t |
| 481 | l | 1.958 | f | 0.973 | 0.448 | w |
| 116 | n | 1.258 | q | 0.97 | 0.484 | i |
| 99 | a | 1.058 | i | 0.963 | 0.403 | s |
| 139 | d | 1.131 | a | 0.959 | 0.448 | k |
| 386 | r | 0.717 | s | 0.933 | 0.417 | y |
| 452 | r | 0.957 | q | 0.93 | 0.374 | m |
| 406 | g | 2.834 | n | 0.908 | 0.283 | a |
| 393 | r | 2.49 | y | 0.907 | 0.51 | a |
| 190 | y | 2.581 | l | 0.849 | 0.379 | s |
| 491 | d | 3.329 | e | 0.848 | 0.26 | n |
| 144 | e | 0.587 | i | 0.819 | 0.423 | s |
| 484 | a | 1.649 | e | 0.793 | 0.329 | i |
| 212 | v | 2.362 | y | 0.748 | 0.298 | m |
| 274 | r | 2.746 | w | 0.739 | 0.193 | m |
| 176 | k | 1.315 | c | 0.734 | 0.414 | g |
| 420 | v | 1.255 | a | 0.734 | 0.315 | w |
| 378 | g | 2.388 | q | 0.692 | 0.39 | g |
| 192 | e | 1.483 | m | 0.674 | 0.388 | l |
| 309 | q | 0.904 | a | 0.665 | 0.334 | w |
| 191 | v | 1.999 | c | 0.651 | 0.169 | w |
| 313 | a | 0.685 | v | 0.621 | 0.252 | w |
| 443 | d | 1.077 | v | 0.618 | 0.285 | t |
| 306 | r | 0.741 | v | 0.61 | 0.284 | q |
| 109 | q | 0.912 | s | 0.576 | 0.29 | v |
| 263 | g | 2.784 | d | 0.574 | 0.22 | s |
| 136 | p | 1.957 | t | 0.544 | 0.221 | k |
| 115 | v | 1.578 | c | 0.54 | 0.232 | k |
| 164 | a | 1.867 | v | 0.54 | 0.234 | c |
| 189 | k | 0.745 | c | 0.51 | 0.245 | k |
| 135 | e | 2.887 | k | 0.496 | 0.29 | d |
| 113 | v | 2.423 | f | 0.494 | 0.288 | l |
| 494 | e | 2.1 | g | 0.471 | 0.199 | y |
| 344 | n | 1.344 | k | 0.46 | 0.209 | p |
| 121 | d | 1.42 | t | 0.452 | 0.198 | n |
| 451 | d | 0.975 | g | 0.45 | 0.214 | c |
| 383 | e | 1.507 | r | 0.422 | 0.181 | m |
| 314 | l | 1.754 | q | 0.42 | 0.205 | i |
| 171 | v | 2.792 | i | 0.379 | 0.162 | t |
| 133 | i | 2.927 | f | 0.368 | 0.203 | v |
| 495 | s | 2.576 | w | 0.356 | 0.157 | a |
| 477 | k | 1.462 | c | 0.353 | 0.175 | i |
| 198 | i | 2.176 | l | 0.335 | 0.19 | c |
| 155 | t | 1.774 | q | 0.308 | 0.182 | i |
| 165 | d | 2.27 | n | 0.28 | 0.123 | l |
| 110 | v | 3.074 | t | 0.254 | 0.153 | i |
| 187 | d | 2.514 | h | 0.253 | 0.1 | c |
| 193 | s | 0.918 | n | 0.224 | 0.116 | r |
| 102 | a | 2.401 | v | 0.205 | 0.09 | c |
| 151 | l | 2.522 | w | 0.158 | 0.085 | i |
| 478 | i | 2.393 | l | 0.142 | 0.071 | m |
| 356 | n | 4.224 | e | 0.136 | 0.081 | n |
| 367 | n | 4.224 | e | 0.136 | 0.081 | n |
| 442 | n | 4.224 | e | 0.136 | 0.081 | n |
| 216 | s | 3.571 | a | 0.128 | 0.076 | s |
| 114 | d | 4.045 | a | 0.12 | 0.071 | d |
| 173 | t | 3.924 | s | 0.12 | 0.072 | t |
| 217 | t | 3.924 | s | 0.12 | 0.072 | t |
| 415 | d | 4.045 | a | 0.12 | 0.071 | d |
| 258 | e | 3.759 | a | 0.118 | 0.07 | e |
| 349 | e | 3.759 | a | 0.118 | 0.07 | e |
| 277 | i | 2.743 | f | 0.116 | 0.06 | l |
| 493 | r | 4.116 | v | 0.099 | 0.059 | r |
| 257 | p | 4.179 | a | 0.092 | 0.055 | p |
| 352 | k | 3.882 | g | 0.088 | 0.053 | k |
| 486 | k | 3.882 | g | 0.088 | 0.053 | k |
| 124 | n | 2.433 | q | 0.086 | 0.044 | r |
| 169 | i | 3.057 | f | 0.082 | 0.044 | l |
| 89 | g | 3.719 | c | 0.081 | 0.048 | g |
| 91 | g | 3.719 | c | 0.081 | 0.048 | g |
| 94 | g | 3.719 | c | 0.081 | 0.048 | g |
| 408 | g | 3.719 | c | 0.081 | 0.048 | g |
| 409 | g | 3.719 | c | 0.081 | 0.048 | g |
| 220 | v | 2.44 | c | 0.073 | 0.04 | e |
| 278 | g | 3.318 | d | 0.063 | 0.025 | s |
| 411 | c | 5.835 | g | 0.043 | 0.026 | c |
| 92 | y | 4.649 | f | 0.027 | 0.014 | h |
