# Supplementary material for: Analysis of nucleotide diphosphate sugar dehydrogenases reveals family and group‐specific relationships
Source: FEBS Open Bio. 2016 Jan 11;6(1):77–89. doi: 10.1002/2211-5463.12022 (PMC4794789; doi:10.1002/2211-5463.12022)
Supplement: Supplementary file 5 — Table S3. Complete GEnt results of UDPGDHs. [file FEB4-6-77-s005.docx]

Table S3. Complete GEnt results of UDPGDHs.

| Index | SeqAln | Entropy | SeqGrp | GroupEntropy | PartGroup | SeqNotGroup |
| --- | --- | --- | --- | --- | --- | --- |
| 359 | h | 2.206 | l | 12.577 | 7.208 | h |
| 366 | a | 1.683 | f | 10.13 | 5.844 | a |
| 112 | c | 1.679 | c | 10.031 | 6.2 | g |
| 215 | k | 2.368 | k | 9.458 | 5.275 | r |
| 90 | l | 1.298 | a | 9.421 | 5.297 | l |
| 355 | c | 1.655 | a | 8.211 | 4.345 | c |
| 387 | a | 1.277 | g | 7.83 | 3.789 | m |
| 123 | w | 2.396 | w | 7.217 | 5.579 | i |
| 446 | k | 1.258 | r | 7.086 | 3.822 | v |
| 417 | w | 1.505 | l | 7.018 | 3.578 | r |
| 153 | f | 2.058 | f | 6.911 | 4.76 | g |
| 318 | w | 1.92 | w | 6.674 | 4.732 | v |
| 435 | w | 2.406 | w | 6.448 | 5.309 | l |
| 167 | v | 1.071 | i | 6.41 | 2.985 | w |
| 347 | s | 1.33 | s | 6.408 | 3.148 | v |
| 389 | c | 1.856 | g | 6.372 | 3.773 | l |
| 404 | g | 2.594 | s | 6.26 | 4.455 | g |
| 229 | w | 1.235 | k | 6.251 | 2.882 | w |
| 439 | i | 1.113 | i | 6.238 | 3.437 | m |
| 362 | r | 2.246 | r | 6.167 | 4.076 | q |
| 255 | h | 1.445 | s | 6.115 | 3.182 | h |
| 379 | a | 1.396 | a | 6.077 | 3.381 | i |
| 227 | r | 0.653 | r | 6.021 | 3.501 | v |
| 214 | e | 0.806 | e | 5.989 | 3.452 | f |
| 449 | f | 1.191 | f | 5.988 | 3.631 | c |
| 254 | a | 1.393 | l | 5.944 | 3.764 | c |
| 492 | t | 1.367 | t | 5.941 | 2.987 | l |
| 280 | m | 0.613 | d | 5.929 | 2.814 | f |
| 418 | n | 1.33 | q | 5.912 | 3.468 | f |
| 412 | f | 2.273 | f | 5.911 | 4.017 | i |
| 134 | y | 1.502 | y | 5.641 | 3.275 | v |
| 126 | g | 1.482 | r | 5.617 | 3.776 | g |
| 385 | i | 1.56 | a | 5.469 | 2.91 | m |
| 338 | g | 0.618 | i | 5.366 | 2.788 | g |
| 186 | a | 1.281 | f | 5.319 | 2.552 | l |
| 210 | h | 0.985 | k | 5.031 | 2.427 | h |
| 382 | w | 1.085 | e | 4.828 | 2.688 | f |
| 346 | w | 1.578 | w | 4.824 | 3.376 | e |
| 150 | n | 1.145 | n | 4.766 | 2.353 | y |
| 365 | f | 2.25 | s | 4.657 | 3.164 | l |
| 422 | n | 0.529 | n | 4.626 | 2.261 | r |
| 372 | i | 1.681 | y | 4.624 | 2.565 | i |
| 413 | p | 2.152 | q | 4.523 | 2.797 | a |
| 175 | y | 1.012 | t | 4.426 | 2.449 | f |
| 261 | r | 1.54 | a | 4.394 | 2.435 | l |
| 399 | h | 1.179 | k | 4.338 | 2.397 | y |
| 168 | f | 1.732 | f | 4.322 | 1.841 | m |
| 361 | q | 1.222 | q | 4.321 | 2.838 | v |
| 400 | y | 2.319 | f | 4.317 | 2.436 | i |
| 231 | d | 0.358 | r | 4.209 | 2.235 | i |
| 95 | l | 1.485 | g | 4.196 | 2.267 | c |
| 209 | y | 0.83 | y | 4.111 | 1.954 | w |
| 450 | a | 1.022 | a | 4.094 | 2.323 | l |
| 454 | i | 0.572 | i | 4.084 | 2.28 | d |
| 317 | h | 0.773 | h | 4.046 | 2.102 | i |
| 416 | v | 1.385 | t | 4.014 | 2.237 | p |
| 434 | y | 1.826 | y | 3.922 | 2.929 | l |
| 374 | e | 1.283 | e | 3.918 | 1.926 | r |
| 403 | p | 2.793 | a | 3.893 | 2.77 | p |
| 496 | p | 2.33 | s | 3.886 | 2.499 | p |
| 342 | t | 0.68 | f | 3.827 | 1.955 | r |
| 364 | s | 1.861 | s | 3.814 | 2.201 | g |
| 350 | m | 2.215 | l | 3.78 | 1.941 | m |
| 307 | c | 1.473 | r | 3.738 | 1.811 | c |
| 170 | c | 2.181 | s | 3.737 | 2.074 | c |
| 368 | e | 2.158 | s | 3.722 | 2.641 | e |
| 498 | i | 1.301 | q | 3.708 | 2.027 | m |
| 388 | v | 1.687 | m | 3.667 | 1.69 | f |
| 98 | c | 1.893 | m | 3.654 | 2.278 | r |
| 497 | a | 1.277 | i | 3.646 | 1.556 | l |
| 103 | h | 0.562 | h | 3.643 | 1.899 | g |
| 161 | i | 1.405 | y | 3.624 | 1.776 | p |
| 476 | k | 1.061 | k | 3.589 | 2.071 | l |
| 308 | v | 0.739 | v | 3.57 | 1.699 | g |
| 172 | p | 2.204 | n | 3.563 | 2.642 | g |
| 251 | f | 1.159 | d | 3.558 | 2.056 | v |
| 405 | f | 1.686 | v | 3.544 | 2.403 | a |
| 448 | w | 1.363 | v | 3.526 | 1.221 | w |
| 444 | y | 0.747 | w | 3.506 | 1.913 | s |
| 410 | h | 2.458 | y | 3.473 | 1.95 | h |
| 376 | t | 0.703 | t | 3.384 | 1.986 | q |
| 200 | q | 0.671 | q | 3.37 | 1.546 | y |
| 398 | n | 0.518 | n | 3.369 | 1.726 | v |
| 445 | q | 2.102 | r | 3.34 | 1.617 | h |
| 270 | l | 0.695 | m | 3.264 | 1.532 | d |
| 480 | c | 1.71 | i | 3.256 | 1.523 | c |
| 211 | i | 1.556 | i | 3.252 | 1.911 | l |
| 390 | m | 0.765 | y | 3.242 | 1.6 | q |
| 195 | c | 1.535 | i | 3.233 | 1.356 | c |
| 219 | p | 2.472 | p | 3.22 | 0.913 | l |
| 185 | a | 0.602 | y | 3.214 | 1.734 | d |
| 406 | g | 2.834 | g | 3.209 | 0.869 | a |
| 197 | m | 0.613 | r | 3.197 | 1.834 | e |
| 252 | q | 0.8 | q | 3.196 | 1.58 | y |
| 104 | m | 0.805 | f | 3.188 | 1.41 | r |
| 223 | t | 1.279 | a | 3.046 | 1.719 | m |
| 311 | l | 1.252 | f | 3.019 | 1.593 | a |
| 354 | t | 1.412 | f | 3.01 | 1.417 | m |
| 360 | a | 2.17 | a | 3.01 | 1.214 | d |
| 119 | k | 1.863 | r | 2.99 | 1.545 | a |
| 348 | a | 2.624 | s | 2.961 | 2.173 | a |
| 152 | f | 0.984 | f | 2.921 | 1.739 | r |
| 437 | q | 0.991 | q | 2.919 | 1.53 | h |
| 351 | c | 1.235 | s | 2.916 | 1.121 | c |
| 269 | l | 1.484 | n | 2.898 | 1.386 | y |
| 407 | f | 2.555 | w | 2.898 | 1.541 | v |
| 265 | a | 1.666 | a | 2.886 | 1.148 | v |
| 438 | v | 1.527 | v | 2.856 | 1.282 | l |
| 208 | g | 0.821 | p | 2.819 | 1.362 | k |
| 371 | a | 0.678 | t | 2.801 | 1.255 | m |
| 228 | r | 0.498 | r | 2.796 | 1.268 | i |
| 131 | s | 0.954 | l | 2.788 | 1.551 | a |
| 225 | s | 0.653 | w | 2.758 | 1.358 | q |
| 345 | t | 0.711 | t | 2.728 | 1.605 | a |
| 447 | r | 0.655 | r | 2.723 | 1.338 | q |
| 341 | i | 1.25 | l | 2.718 | 1.534 | h |
| 357 | a | 1.315 | w | 2.689 | 1.202 | v |
| 490 | d | 2.446 | g | 2.677 | 1.849 | e |
| 402 | q | 1.202 | n | 2.657 | 1.423 | r |
| 218 | v | 1.747 | v | 2.649 | 0.968 | m |
| 273 | d | 1.945 | s | 2.639 | 1.193 | p |
| 166 | l | 0.98 | f | 2.606 | 1.405 | a |
| 184 | h | 1.152 | m | 2.584 | 1.357 | y |
| 274 | r | 2.746 | r | 2.573 | 0.755 | m |
| 256 | c | 2.625 | s | 2.524 | 0.953 | c |
| 370 | s | 1.48 | d | 2.521 | 1.242 | g |
| 401 | l | 1.758 | n | 2.515 | 1.385 | m |
| 414 | k | 2.633 | k | 2.477 | 0.856 | v |
| 108 | h | 1.392 | n | 2.46 | 1.237 | k |
| 433 | r | 0.844 | e | 2.442 | 1.134 | p |
| 482 | g | 3.048 | r | 2.427 | 1.994 | g |
| 474 | t | 0.544 | t | 2.419 | 1.139 | d |
| 391 | d | 3.001 | l | 2.403 | 0.8 | h |
| 358 | f | 2.78 | m | 2.368 | 0.942 | w |
| 441 | m | 1.497 | m | 2.349 | 1.704 | t |
| 97 | t | 1.478 | i | 2.325 | 0.864 | c |
| 271 | n | 0.924 | y | 2.305 | 1.282 | l |
| 111 | t | 1.13 | t | 2.298 | 1.339 | i |
| 141 | v | 1.268 | y | 2.29 | 1.367 | q |
| 487 | p | 1.36 | k | 2.279 | 1.303 | g |
| 230 | f | 1.326 | f | 2.25 | 1.424 | p |
| 343 | t | 1.832 | m | 2.231 | 1.179 | k |
| 272 | p | 2.33 | a | 2.224 | 0.787 | n |
| 489 | t | 2.119 | s | 2.222 | 0.969 | v |
| 339 | p | 0.993 | k | 2.22 | 0.969 | e |
| 220 | v | 2.44 | l | 2.188 | 0.868 | p |
| 393 | r | 2.49 | f | 2.187 | 0.833 | y |
| 485 | f | 3.471 | m | 2.152 | 1.683 | y |
| 436 | q | 0.963 | q | 2.15 | 1.116 | n |
| 162 | k | 0.561 | k | 2.148 | 1.146 | m |
| 99 | a | 1.058 | s | 2.147 | 1.037 | g |
| 122 | m | 0.643 | a | 2.144 | 1.081 | t |
| 262 | e | 2.95 | e | 2.143 | 0.807 | p |
| 260 | l | 2.315 | l | 2.124 | 0.793 | v |
| 93 | v | 2.843 | v | 2.111 | 0.728 | i |
| 392 | p | 1.188 | q | 2.085 | 1.256 | y |
| 475 | d | 0.881 | p | 2.085 | 0.993 | c |
| 483 | f | 2.075 | w | 2.051 | 1.32 | i |
| 259 | f | 3.483 | f | 2.044 | 0.787 | r |
| 384 | v | 2.063 | i | 2.04 | 0.971 | l |
| 194 | c | 1.896 | c | 1.988 | 1.453 | g |
| 419 | l | 2.188 | m | 1.988 | 0.695 | i |
| 201 | v | 0.782 | n | 1.96 | 1.163 | a |
| 305 | q | 0.579 | q | 1.956 | 1.102 | k |
| 222 | t | 1.707 | y | 1.922 | 1.355 | t |
| 118 | h | 0.562 | s | 1.91 | 0.833 | t |
| 132 | p | 2.686 | w | 1.88 | 0.698 | h |
| 121 | d | 1.42 | n | 1.878 | 0.938 | d |
| 100 | c | 1.476 | l | 1.862 | 0.976 | c |
| 137 | g | 1.419 | y | 1.805 | 0.879 | r |
| 440 | d | 0.773 | d | 1.802 | 0.909 | p |
| 420 | v | 1.255 | w | 1.79 | 0.957 | t |
| 174 | p | 3.181 | n | 1.764 | 1.337 | p |
| 189 | k | 0.745 | k | 1.714 | 0.825 | d |
| 481 | l | 1.958 | w | 1.71 | 0.911 | f |
| 139 | d | 1.131 | k | 1.708 | 0.869 | g |
| 268 | d | 2.475 | n | 1.669 | 0.645 | e |
| 199 | a | 1.054 | m | 1.666 | 0.873 | g |
| 381 | v | 1.368 | t | 1.637 | 0.649 | g |
| 312 | c | 0.875 | c | 1.617 | 0.875 | q |
| 154 | t | 1.665 | s | 1.606 | 0.721 | v |
| 221 | g | 2.284 | r | 1.597 | 0.925 | m |
| 101 | f | 1.634 | i | 1.583 | 0.885 | f |
| 163 | e | 0.921 | h | 1.524 | 0.626 | p |
| 96 | p | 2.112 | s | 1.492 | 0.948 | v |
| 491 | d | 3.329 | n | 1.486 | 1.09 | d |
| 315 | y | 3.355 | l | 1.466 | 1.061 | y |
| 187 | d | 2.514 | n | 1.462 | 0.503 | c |
| 432 | a | 0.947 | f | 1.426 | 0.811 | h |
| 125 | q | 0.903 | s | 1.403 | 0.82 | r |
| 452 | r | 0.957 | s | 1.377 | 0.575 | m |
| 484 | a | 1.649 | i | 1.244 | 0.692 | s |
| 156 | d | 1.471 | n | 1.229 | 0.537 | m |
| 310 | a | 0.562 | t | 1.229 | 0.589 | y |
| 213 | v | 2.266 | t | 1.212 | 0.82 | a |
| 267 | y | 0.814 | w | 1.209 | 0.585 | v |
| 309 | q | 0.904 | w | 1.209 | 0.573 | d |
| 164 | a | 1.867 | f | 1.198 | 0.48 | t |
| 279 | g | 1.408 | y | 1.146 | 0.619 | e |
| 117 | q | 0.826 | y | 1.138 | 0.591 | l |
| 386 | r | 0.717 | y | 1.136 | 0.652 | d |
| 116 | n | 1.258 | i | 1.047 | 0.637 | q |
| 130 | k | 0.869 | t | 1.026 | 0.583 | e |
| 191 | v | 1.999 | w | 0.998 | 0.636 | m |
| 176 | k | 1.315 | d | 0.985 | 0.475 | c |
| 314 | l | 1.754 | v | 0.961 | 0.484 | m |
| 421 | y | 1.522 | r | 0.955 | 0.436 | s |
| 264 | t | 1.127 | m | 0.945 | 0.39 | h |
| 190 | y | 2.581 | s | 0.931 | 0.569 | l |
| 453 | i | 1.857 | i | 0.93 | 0.473 | t |
| 115 | v | 1.578 | k | 0.928 | 0.566 | c |
| 375 | a | 0.771 | m | 0.911 | 0.504 | d |
| 344 | n | 1.344 | g | 0.91 | 0.399 | p |
| 494 | e | 2.1 | s | 0.899 | 0.531 | y |
| 193 | s | 0.918 | d | 0.852 | 0.333 | r |
| 171 | v | 2.792 | t | 0.836 | 0.54 | v |
| 212 | v | 2.362 | m | 0.822 | 0.582 | y |
| 313 | a | 0.685 | w | 0.822 | 0.521 | s |
| 451 | d | 0.975 | c | 0.822 | 0.458 | l |
| 495 | s | 2.576 | a | 0.82 | 0.52 | s |
| 455 | d | 0.55 | d | 0.818 | 0.415 | i |
| 144 | e | 0.587 | r | 0.802 | 0.438 | i |
| 266 | i | 1.592 | r | 0.777 | 0.329 | w |
| 306 | r | 0.741 | w | 0.664 | 0.312 | y |
| 143 | k | 0.736 | f | 0.659 | 0.294 | h |
| 353 | y | 2.363 | a | 0.657 | 0.299 | i |
| 488 | n | 1.955 | r | 0.653 | 0.306 | g |
| 135 | e | 2.887 | d | 0.643 | 0.376 | k |
| 196 | r | 1.539 | q | 0.623 | 0.308 | l |
| 140 | e | 1.052 | y | 0.56 | 0.219 | m |
| 383 | e | 1.507 | m | 0.532 | 0.318 | k |
| 113 | v | 2.423 | l | 0.527 | 0.312 | f |
| 109 | q | 0.912 | v | 0.508 | 0.283 | e |
| 224 | e | 1.073 | r | 0.488 | 0.18 | l |
| 188 | l | 1.911 | t | 0.472 | 0.188 | m |
| 380 | d | 2.636 | r | 0.439 | 0.207 | s |
| 198 | i | 2.176 | c | 0.43 | 0.234 | m |
| 192 | e | 1.483 | l | 0.401 | 0.187 | m |
| 369 | i | 1.98 | m | 0.385 | 0.196 | f |
| 120 | i | 2.659 | i | 0.384 | 0.19 | l |
| 253 | v | 2.127 | i | 0.382 | 0.17 | y |
| 136 | p | 1.957 | f | 0.371 | 0.203 | e |
| 478 | i | 2.393 | m | 0.369 | 0.183 | v |
| 316 | e | 1.03 | l | 0.353 | 0.18 | g |
| 165 | d | 2.27 | l | 0.351 | 0.193 | e |
| 277 | i | 2.743 | v | 0.298 | 0.163 | f |
| 477 | k | 1.462 | i | 0.272 | 0.161 | c |
| 124 | n | 2.433 | i | 0.261 | 0.116 | s |
| 155 | t | 1.774 | l | 0.255 | 0.13 | q |
| 102 | a | 2.401 | c | 0.249 | 0.115 | t |
| 133 | i | 2.927 | i | 0.228 | 0.103 | v |
| 378 | g | 2.388 | r | 0.204 | 0.094 | d |
| 263 | g | 2.784 | g | 0.197 | 0.09 | a |
| 278 | g | 3.318 | s | 0.195 | 0.125 | g |
| 443 | d | 1.077 | y | 0.174 | 0.09 | a |
| 110 | v | 3.074 | i | 0.149 | 0.082 | t |
| 169 | i | 3.057 | v | 0.118 | 0.062 | l |
| 479 | a | 1.911 | a | 0.107 | 0.049 | t |
| 138 | l | 2.099 | p | 0.1 | 0.048 | n |
| 151 | l | 2.522 | y | 0.079 | 0.035 | v |
| 373 | c | 3.708 | a | 0.067 | 0.029 | g |
| 92 | y | 4.649 | h | 0.027 | 0.017 | y |
| 363 | i | 2.754 | i | 0.027 | 0.013 | g |
| 216 | s | 3.571 | s | 0.01 | 0.005 | a |
| 356 | n | 4.224 | n | 0.01 | 0.005 | a |
| 367 | n | 4.224 | n | 0.01 | 0.005 | a |
| 442 | n | 4.224 | n | 0.01 | 0.005 | a |
| 114 | d | 4.045 | d | 0.009 | 0.004 | r |
| 173 | t | 3.924 | t | 0.009 | 0.004 | r |
| 217 | t | 3.924 | t | 0.009 | 0.004 | r |
| 258 | e | 3.759 | e | 0.009 | 0.004 | a |
| 349 | e | 3.759 | e | 0.009 | 0.004 | a |
| 415 | d | 4.045 | d | 0.009 | 0.004 | r |
| 257 | p | 4.179 | p | 0.007 | 0.003 | d |
| 352 | k | 3.882 | k | 0.007 | 0.003 | a |
| 486 | k | 3.882 | k | 0.007 | 0.003 | a |
| 493 | r | 4.116 | r | 0.007 | 0.003 | a |
| 89 | g | 3.719 | g | 0.006 | 0.003 | r |
| 91 | g | 3.719 | g | 0.006 | 0.003 | r |
| 94 | g | 3.719 | g | 0.006 | 0.003 | r |
| 408 | g | 3.719 | g | 0.006 | 0.003 | r |
| 409 | g | 3.719 | g | 0.006 | 0.003 | r |
| 411 | c | 5.835 | c | 0.003 | 0.002 | a |
